# Supplementary material for: Systematic, comprehensive, evidence-based approach to identify neuroprotective interventions for motor neuron disease: using systematic reviews to inform expert consensus
Source: BMJ Open. 2023 Feb 1;13(2):e064169. doi: 10.1136/bmjopen-2022-064169 (PMC9896226; doi:10.1136/bmjopen-2022-064169)
Supplement: Author's manuscript [file bmjopen-2022-064169.draft_revisions.pdf]

BMJ Open

BMJ Open is committed to open peer review. As part of this commitment we make the peer review history of every article we publish publicly available.

When an article is published we post the peer reviewers' comments and the authors' responses online. We also post the versions of the paper that were used during peer review. These are the versions that the peer review comments apply to.

The versions of the paper that follow are the versions that were submitted during the peer review process. They are not the versions of record or the final published versions. They should not be cited or distributed as the published version of this manuscript.

BMJ Open is an open access journal and the full, final, typeset and author-corrected version of record of the manuscript is available on our site with no access controls, subscription charges or pay-per-view fees (http://bmjopen.bmj.com).

If you have any questions on BMJ Open's open peer review process please email info.bmjopen@bmj.com

BMJ Open

A Systematic Approach to Identify Neuroprotective Interventions for Motor Neuron Disease

Journal:	*BMJ Open*
Manuscript ID	bmjopen-2022-064169
Article Type:	Original research
Date Submitted by the Author:	25-Apr-2022
Complete List of Authors:	Wong, Charis; The University of Edinburgh Centre for Clinical Brain Sciences, Anne Rowling Regenerative Neurology Clinic; The University of Edinburgh Centre for Clinical Brain Sciences, Euan MacDonald Centre for Motor Neuron Disease Research<br>Gregory, Jenna M.; The University of Edinburgh Centre for Clinical Brain Sciences; University of Aberdeen Institute of Medical Sciences<br>Liao, Jing; The University of Edinburgh Centre for Clinical Brain Sciences<br>Egan, Kieren ; University of Strathclyde, Computer and Information Science; The University of Edinburgh Centre for Clinical Brain Sciences<br>Vesterinen, Hanna M.; The University of Edinburgh Centre for Clinical Brain Sciences<br>Ahmad Khan, Aimal; The University of Edinburgh Edinburgh Medical School<br>Anwar, Maarij; The University of Edinburgh Edinburgh Medical School<br>Beagan, Caitlin; The University of Edinburgh Edinburgh Medical School<br>Brown, Fraser; The University of Edinburgh Centre for Clinical Brain Sciences; The University of Edinburgh Centre for Clinical Brain Sciences, Anne Rowling Regenerative Neurology Clinic<br>Cafferkey, John; NHS Lothian, Royal Infirmary of Edinburgh, NHS Lothian, Edinburgh, UK<br>Cardinali, Alessandra; The University of Edinburgh Centre for Clinical Brain Sciences, Euan MacDonald Centre for Motor Neuron Disease Research; UK Dementia Research Institute, University of Edinburgh<br>Chiam, Jane Yi; The University of Edinburgh Edinburgh Medical School<br>Chiang, Claire; The University of Edinburgh Edinburgh Medical School<br>Collins, Victoria; The University of Edinburgh Edinburgh Medical School<br>Dormido, Joyce; NHS Borders<br>Elliott, Elizabeth; The University of Edinburgh Centre for Clinical Brain Sciences, Anne Rowling Regenerative Neurology Clinic; The University of Edinburgh Centre for Clinical Brain Sciences, Euan MacDonald Centre for Motor Neuron Disease Research<br>Foley, Peter; The University of Edinburgh Centre for Clinical Brain Sciences, Anne Rowling Regenerative Neurology Clinic<br>Foo, Yu Cheng; The University of Edinburgh Edinburgh Medical School<br>Fulton-Humble, Lily; University of Aberdeen, Institute of Medical Sciences<br>Gane, Angus; The University of Edinburgh College of Medicine and Veterinary Medicine<br>Glasmacher, Stella; The University of Edinburgh Centre for Clinical Brain Sciences, Anne Rowling Regenerative Neurology Clinic; The University of Edinburgh Centre for Clinical Brain Sciences, Euan MacDonald Centre for

Motor Neuron Disease Research
Heffernan, Áine; UK Dementia Research Institute, University of Edinburgh
Jayaprakash, Kiran; The University of Edinburgh Centre for Clinical Brain Sciences, Anne Rowling Regenerative Neurology Clinic; The University of Edinburgh Centre for Clinical Brain Sciences
Jayasuriya, Nimesh; The University of Edinburgh College of Medicine and Veterinary Medicine; NHS Lothian
Kaddouri, Amina; The University of Edinburgh Edinburgh Medical School
Kiernan, Jamie; The University of Edinburgh Edinburgh Medical School
Langlands, Gavin; NHS Greater Glasgow and Clyde
Leighton, D; The University of Edinburgh Centre for Clinical Brain Sciences; University of Glasgow, School of Psychology and Neuroscience
Liu, Jiaming; The University of Edinburgh Edinburgh Medical School
Lyon, James; NHS Lothian
Mehta, Arpan; The University of Edinburgh Centre for Clinical Brain Sciences, Anne Rowling Regenerative Neurology Clinic; The University of Edinburgh Centre for Clinical Brain Sciences, Euan MacDonald Centre for Motor Neuron Disease Research
Meng, Alyssa; The University of Edinburgh Centre for Discovery Brain Sciences
Nguyen, Vivienne; The University of Edinburgh Edinburgh Medical School
Park, Na Hyun; NHS Lothian
Quigley, Suzanne; The University of Edinburgh Centre for Clinical Brain Sciences, Anne Rowling Regenerative Neurology Clinic; The University of Edinburgh Centre for Clinical Brain Sciences
Rashid, Yousuf; The University of Edinburgh Edinburgh Medical School
Salzinger, Andrea; The University of Edinburgh Centre for Clinical Brain Sciences; UK Dementia Research Institute, University of Edinburgh
Shiell, Bethany; The University of Edinburgh College of Medicine and Veterinary Medicine
Singh, Ankur; The University of Edinburgh College of Medicine and Veterinary Medicine
Soane, Tim; NHS Forth Valley
Thompson, Alexandra; The University of Edinburgh College of Medicine and Veterinary Medicine
Tomala, Olaf; The University of Edinburgh Edinburgh Medical School
Waldron, Fergal; University of Aberdeen, Institute of Medical Sciences; The University of Edinburgh Institute of Evolutionary Biology
Selvaraj, Bhuvaneish; The University of Edinburgh Centre for Clinical Brain Sciences, Anne Rowling Regenerative Neurology Clinic; The University of Edinburgh Centre for Clinical Brain Sciences, Euan MacDonald Centre for Motor Neuron Disease Research
Chataway, Jeremy; UCL Queen Square Institute of Neurology, Queen Square Multiple Sclerosis Centre, Department of Neuroinflammation,; National Institute for Health Research, University College London Hospitals, Biomedical Research Centre
Swingler, Robert; The University of Edinburgh Centre for Clinical Brain Sciences, Euan MacDonald Centre for Motor Neuron Disease Research
Connick, Peter; The University of Edinburgh Centre for Clinical Brain Sciences; The University of Edinburgh Centre for Clinical Brain Sciences, Anne Rowling Regenerative Neurology Clinic
Pal, Suvankar; The University of Edinburgh Centre for Clinical Brain Sciences, Anne Rowling Regenerative Neurology Clinic; The University of Edinburgh Centre for Clinical Brain Sciences, Euan MacDonald Centre for Motor Neuron Disease Research
Chandran, Siddharthan ; The University of Edinburgh Centre for Clinical Brain Sciences, Anne Rowling Regenerative Neurology Clinic; UK Dementia Research Institute, University of Edinburgh
Macleod, Malcolm; The University of Edinburgh Centre for Clinical Brain Sciences

Keywords:	Clinical trials < THERAPEUTICS, Motor neurone disease < NEUROLOGY, Adult neurology < NEUROLOGY, NEUROLOGY, THERAPEUTICS

SCHOLARONE™
Manuscripts

I, the Submitting Author has the right to grant and does grant on behalf of all authors of the Work (as defined in the below author licence), an exclusive licence and/or a non-exclusive licence for contributions from authors who are: i) UK Crown employees; ii) where BMJ has agreed a CC-BY licence shall apply, and/or iii) in accordance with the terms applicable for US Federal Government officers or employees acting as part of their official duties; on a worldwide, perpetual, irrevocable, royalty-free basis to BMJ Publishing Group Ltd ("BMJ") its licensees and where the relevant Journal is co-owned by BMJ to the co-owners of the Journal, to publish the Work in this journal and any other BMJ products and to exploit all rights, as set out in our licence.

The Submitting Author accepts and understands that any supply made under these terms is made by BMJ to the Submitting Author unless you are acting as an employee on behalf of your employer or a postgraduate student of an affiliated institution which is paying any applicable article publishing charge ("APC") for Open Access articles. Where the Submitting Author wishes to make the Work available on an Open Access basis (and intends to pay the relevant APC), the terms of reuse of such Open Access shall be governed by a Creative Commons licence – details of these licences and which Creative Commons licence will apply to this Work are set out in our licence referred to above.

Other than as permitted in any relevant BMJ Author's Self Archiving Policies, I confirm this Work has not been accepted for publication elsewhere, is not being considered for publication elsewhere and does not duplicate material already published. I confirm all authors consent to publication of this Work and authorise the granting of this licence.

For peer review only

TITLE

A Systematic Approach to Identify Neuroprotective Interventions for Motor Neuron Disease

AUTHORS

The Writing Committee for Repurposing Living Systematic Review – Motor Neuron Disease (ReLiSyR-MND)

Charis Wong[1,2,3], Jenna M. Gregory[1,3,4], Jing Liao[1], Kieren Egan[1], Hanna M. Vesterinen[1], Aimal Ahmad Khan[5], Maarij Anwar[5], Caitlin Beagan[6], Fraser Brown[1,2], John Cafferkey[7], Alessandra Cardinali[1,3,8], Jane Yi Chiam[5], Claire Chiang[6], Victoria Collins[6], Joyce Dormido[9], Elizabeth Elliott[1,2,3], Peter Foley[1,2], Yu Cheng Foo[5], Lily Fulton-Humble[4], Angus B. Gane[6], Stella A. Glasmacher[1,2], Áine Heffernan[8], Kiran Jayaprakash[1,2], Nimesh Jayasuriya[6,10], Amina Kaddouri[5], Jamie Kiernan[5], Gavin Langlands[11], Danielle Leighton[1,3,12], Jiaming Liu[5], James Lyon[10], Arpan R. Mehta[1,2,3,8], Alyssa Meng[13], Vivienne Nguyen[5], Na Hyun Park[10], Suzanne Quigley[1,2], Yousuf Rashid[5], Andrea Salzinger[1,8], Bethany Shiell[6], Ankur Singh[6], Tim Soane[14], Alexandra Thompson[5], Olaf Tomala[5], Fergal M. Waldron[4,15], Bhuvaneish T. Selvaraj[1,2,3,8], Jeremy Chataway[16,17,18], Robert Swingler[3], Peter Connick[1,2], Suvankar Pal[1,2,3], Siddharthan Chandran[1,2,3,8], Malcolm R. Macleod[1]

[1]Centre for Clinical Brain Sciences, University of Edinburgh, Edinburgh, UK, [2]Anne Rowling Regenerative Neurology Clinic, University of Edinburgh, UK, [3]Euan MacDonald Centre for Motor Neuron Disease Research, University of Edinburgh, Edinburgh, UK, [4]Institute of Medical Sciences, University of Aberdeen, Aberdeen, UK, [5]Edinburgh Medical School, University of Edinburgh, Edinburgh, UK, [6]University of Edinburgh, Edinburgh, UK, [7]Royal Infirmary of Edinburgh, NHS Lothian, Edinburgh, UK, [8]UK Dementia Research Institute at University of Edinburgh, University of Edinburgh, Edinburgh bioQuarter, Chancellor's Building, 49 Little France Crescent, Edinburgh, UK, [9]NHS Borders and Midlothian, UK, [10]NHS Lothian, Edinburgh, UK, [11]NHS Greater Glasgow and Clyde, Glasgow, UK, [12]School of Psychology and Neuroscience, University of Glasgow, Glasgow, UK, [13]Centre for Discovery Brain Sciences, University of Edinburgh, Edinburgh, UK, [14]NHS Forth Valley, Larbert, UK, [15]Institute of Evolutionary Biology, University of Edinburgh, Edinburgh, UK, [16]Queen Square Multiple Sclerosis Centre, Department of Neuroinflammation, UCL Queen Square Institute of Neurology, Faculty of Brain Sciences, University College London, London, UK, [17]National Institute for Health Research, University College London Hospitals, Biomedical Research Centre, London, UK, [18]Medical Research Council Clinical Trials Unit at UCL, Institute of Clinical Trials and Methodology, University College London, London, UK

Corresponding Author:

Malcolm R. Macleod

Address: Centre for Clinical Brain Sciences, University of Edinburgh, Edinburgh, UK.

Email: malcolm.macleod@ed.ac.uk

Word count: 3435 words

ORCID iD

Charis Wong 0000-0002-8488-037X

Jenna M. Gregory 0000-0003-3337-4079

Jing Liao 0000-0002-9591-8070

Kieren Egan 0000-0002-1639-4281

Fraser Brown 0000-0002-8314-4936

John Cafferkey 0000-0001-6926-9508

Peter Foley 0000-0001-7039-2976

Nimesh Jayasuriya 0000-0002-0688-3161

Gavin Langlands 0000-0002-8972-4798

Danielle Leighton 0000-0003-2015-7198

Arpan R. Mehta 0000-0002-5715-5025

Na Hyun Park 0000-0003-4674-6767

Fergal M. Waldron 0000-0003-2622-0776

Bhuvaneish T. Selvaraj 0000-0001-7299-0910

Jeremy Chataway 0000-0001-7286-6901

Peter Connick 0000-0002-3892-8037

Suvankar Pal 0000-0003-4276-639X

Siddharthan Chandran 0000-0001-6827-1593

Malcolm R. Macleod 0000-0001-9187-9839

For peer review only

ABSTRACT

Objectives:

Motor neuron disease (MND) is an incurable progressive neurodegenerative disease with limited treatment options. There is a pressing need for innovation in identifying therapies to take to clinical trial. Here we detail a systematic, structured, and unbiased evidence-based approach to guide selection of drugs for clinical evaluation in the Motor Neuron Disease – Systematic Multi-arm Adaptive Randomised Trial (MND-SMART, clinicaltrials.gov registration number: NCT04302870), an adaptive platform trial.

Methods:

We conducted a two-stage systematic review and meta-analysis to identify potential neuroprotective interventions. In stage one, we identified drugs from the clinical literature tested in at least one study in MND or in two or more cognate diseases with potential shared pivotal pathways (Alzheimer's disease, Huntington's disease, Parkinson's disease, or multiple sclerosis). We scored and ranked 66 drugs thus identified using a predefined framework evaluating safety, efficacy, study size and quality of studies. In stage two, we conducted a systematic review of the MND preclinical literature describing efficacy of these drugs in animal models, multicellular eukaryotic models and human induced pluripotent stem cell studies; 17 of these drugs were reported to improve survival in at least one preclinical study. An expert panel then shortlisted and ranked 22 drugs considering stage one and stage two findings, mechanistic plausibility, safety and tolerability, findings from previous clinical trials in MND, and feasibility for use in clinical trials.

Results:

Based on this process, the panel selected memantine and trazodone for testing in MND-SMART.

Discussion:

For future drug selection, we will incorporate automation tools, text-mining and machine learning techniques to the systematic reviews and consider data generated from other domains, including high-throughput phenotypic screening of human induced pluripotent stem cells.

Keywords

Motor neuron disease, amyotrophic lateral sclerosis, systematic review, evidence synthesis, clinical trials

For peer review only

STRENGTHS AND LIMITATIONS OF THIS STUDY:

- We described a systematic, evidence-based approach towards drug repurposing in motor neuron disease (MND), specifically for Motor Neuron Disease – Systematic Multi-arm Adaptive Randomised Trial (MND-SMART), a phase III multi-arm multi-stage clinical trial in MND.
- Systematic reviews of clinical studies in neurodegenerative diseases and MND preclinical studies provided a robust evidence base to inform expert panel decisions on drug selection for clinical trials.
- Providing a contemporary evidence base using traditional systematic reviews is challenging given their time-consuming and labour-intensive nature.
- Incorporation of machine learning and automation tools for systematic reviews, and data from experimental drug screening can be helpful for future drug selection.

For peer review only

INTRODUCTION

Motor neuron disease (MND), also known as amyotrophic lateral sclerosis (ALS), is a progressive neurodegenerative disease with a median survival of 2-3 years.[1] Despite many promising preclinical studies and 125 phase II and phase III trials reported between 2008 and 2019, riluzole remains the only globally approved disease-modifying treatment, prolonging survival by an average of two to three months.[2] Edavarone and masitinib have both emerged as potentially promising candidates in clinical trials, but treatment effects are modest and neither drug has received approval in Europe.[3 ,4] In a long-term multi-centre prospective cohort study, edaravone showed no significant disease-modifying effect.[5] Previously, decisions to evaluate drugs in MND have been informed by preclinical studies, typically using mouse models, such as the SOD1^{G93A} mouse, despite known limitations in the extent to which such models recapitulate human pathology,[6] and concerns of the reproducibility of findings from such models.[7] Clinical trials in MND are further complicated by the challenges of designing and delivering trials in a rapidly progressive, heterogeneous, disabling and fatal disease with a lack of reliable and sensitive outcome measures or biomarkers.[2]

Over the same period there have, however, been rapid technical advances in MND genomics, human induced pluripotent stem cells and gene-editing, which have enabled better understanding of underlying pathophysiology (including potential shared pathways across neurodegenerative diseases), and the development of more sophisticated disease models. In parallel, drug repurposing (testing a drug already used or tested for other indications) has been successfully adopted in many diseases and can significantly reduce development time and cost, with the added benefit of the availability of prior safety data to guide selection.[8] In relapsing-remitting multiple sclerosis for instance, dimethyl fumarate, cladribine,[9], alemtuzumab,[10 ,11] and rituximab[12] provide examples of successful repurposing as disease-modifying treatments.

Systematic review has been recommended to have a key role in planning new research studies.[13] We previously used a strategy based on systematic review to identify repurposed interventions for secondary progressive multiple sclerosis (MS). This involved a two-stage systematic review and meta-analysis assessing clinical and preclinical data to identify putative therapeutic interventions[14] and led to the Multiple Sclerosis – Secondary Progressive Multi-Arm Randomisation Trial (MS-SMART), a phase IIb multi-arm randomised controlled trial.[15 ,16] The three drugs selected for MS-SMART were based in part on their availability for investigator-led clinical trials and did not show efficacy, but two of the top seven drugs thus identified, ibudilast (ranked first), and lipoic acid, have since shown promise in phase II studies in secondary progressive MS.[17 ,18]

Noting similarities between MS and MND as neurodegenerative diseases with limited treatment options, in 2014 we embarked on a similar strategy to identify candidate oral neuroprotective agents in MND. In parallel, we developed the multi-arm multi-stage Motor Neuron Disease – Systematic Multi-Arm Adaptive Randomised Trial (MND-SMART, clinicaltrials.gov registration number: NCT04302870) to provide a more efficient pipeline to evaluate drugs in MND than conventional

For peer review only

standalone two-arm trials.[19 ,20] Here we describe the development and implementation of a systematic, structured, and unbiased evidence-based approach to inform the selection of potential oral neuroprotective agents for clinical evaluation in MND-SMART. Specifically, the purpose here is not to provide a contemporary summary of existing evidence, but to describe the process through which clinical trial drugs were selected.

METHODS

The work was guided by a systematic review protocol. Over the duration of the project and given the novelty of this approach, this protocol was updated in the light of accumulating experience, and the complete record of the protocol, including the changes made, is available at Open Science Framework.[21]

Overview

The overall drug selection strategy is characterised in Figure 1. We used systematic review to identify publications describing clinical trials or reports of the clinical use of drugs in MND and in four other neurodegenerative diseases which we considered might share pivotal pathways: Alzheimer's disease (AD), Parkinson's disease (PD), Huntington's disease (HD) and MS. For MS, we excluded studies of relapsing-remitting disease since we were interested in drugs tested in the progressive phase where neurodegeneration is a major feature. We annotated publications for the drugs tested and diseases studied, taking forward drugs described in at least one MND publication or in publications in at least two other diseases. We scored each drug using a predefined framework evaluating efficacy, safety, study size and quality. In parallel, we performed a systematic review and meta-analysis of the preclinical MND and frontotemporal dementia (FTD; because of pathological overlap with MND) literature for these drugs. We summarised evidence from both reviews for each drug and presented these to an expert panel consisting of clinical and academic neurologists with expertise in MND, clinical trials, pharmacology/drug selection, and preclinical models of MND.

Systematic review of clinical evidence

The MS-SMART drug selection process used the same strategy, except we selected drugs tested at least once in MS or in at least two other conditions. The protocol for and results from this search, conducted in September 2011, has been published.[14] That search involved three online databases (PubMed, ISI Web of Knowledge and EMBASE) using the terms "multiple sclerosis" OR "Alzheimer's disease" OR "Huntington's disease" OR "Parkinson's disease" OR "motor neuron disease" OR "amyotrophic lateral sclerosis". On 13 December 2013, we updated the search using the same terms but with limitations for PubMed to clinical trials, and date of record creation after 01/07/2011; for ISI Web of Knowledge to Document type "Clinical trial" and publication years: 2011, 2012 and 2013; for EMBASE: previous search string AND ("case series" or "case report" or "cohort study"), with limits: human studies, full text studies from 2011; and we also contacted the Cochrane Neuromuscular review group to obtain a list of interventions tested in MND/ALS. The protocol of this update was

For peer review only

stored locally; in the light of increasing recognition of the importance of making systematic review protocols available, the protocol was published without amendment in September 2019.[22]

Two reviewers (MM and KE) independently screened title and abstracts of publications identified in the new search against the inclusion and exclusion criteria (Table 1) with discrepancies resolved by discussion. We included case reports, uncontrolled case series, non-randomised parallel group studies, crossover studies, and randomised controlled trials with any report of safety or efficacy. We extracted basic information from each publication including author, year of publication, intervention tested and disease.

Table 1: Eligibility criteria for clinical systematic review.

**Inclusion criteria**
- Publications reporting qualitative or quantitative data provided on either safety or efficacy of an orally delivered intervention in people with motor neuron disease / amyotrophic lateral sclerosis, Alzheimer's disease, Parkinson's disease, Huntington's disease or multiple sclerosis<br><br>- Studies reporting change in clinical status (including death, tracheostomy free survival, relapse frequency, disability progression, behavioural symptoms), or changes in biomarkers (including magnetic resonance imaging, blood, cerebrospinal fluid and muscle strength)
**Exclusion criteria**
- Isolated reporting of non-pharmacological interventions such as acupuncture, aromatherapy, physiotherapy or exercise<br>- Articles reporting the use of interventions already licensed for clinical use in MND such as riluzole<br>- Articles on levodopa treatment for Parkinson's disease<br>- Studies reporting different modes of intervention delivery other than oral administration<br>- Publications reporting secondary analysis of previously published clinical trial data<br>- Protocols for clinical trials<br>- Preventative studies<br>- Reviews<br>- Studies on healthy volunteers<br>- Studies in patients with relapsing-remitting MS<br>- Studies reporting combination treatments including where an oral and a non-oral intervention are administered<br>- Publications where disease type is not specified to be in keeping with the included diseases (studies of vascular dementia, mild cognitive impairment and dementias other than Alzheimer's disease are excluded<br>- Studies on patients with parkinsonism are excluded as this do not imply Parkinson's disease exclusively)<br>- Publications describing studies where multiple drugs were tested in a cohort without any data on individual drugs

For peer review only

For all candidate interventions which had not been excluded based on feasibility or plausibility, we extracted further information on safety, efficacy, quality of study and study size from publications to a Microsoft Access database and scored these against a predefined metric (Table 2 and Table 3). For each drug, we calculated an overall drug score by taking the product of the mean score in each domain for safety, efficacy, quality, study size and multiplying this by $\log_{10}$(1+number of publications). We then ranked drugs according to these scores.

Table 2: Scoring metric for clinical review

**Safety Score [S]**<br>"not described": 1 point<br>"SUSARs (Suspected Unexpected Serious Adverse Reactions) or mortality observed": 1 point<br>"SAEs (Serious Adverse Events) only": 2 points<br>"AEs (Adverse events) only": 3 points<br>"No adverse effects reported": 4 points
**Efficacy Score [E]**<br>***Efficacy score is assigned based on primary outcome measure, and where this is not identified, on the mean efficacy score for all outcomes reported in each publication.***<br>"not presented": 1 point<br>"definite (i.e. statistically significant) worsening": 1 point<br>"neutral": 2 points<br>"non-significant improvement": 3 points<br>"significant improvement": 4 points
**Quality Score [Q]**<br>Study quality was assessed using a combination of criteria taken from a risk of bias tool developed through a Delphi process, GRADE and CAMARADES methods as shown in Table 3. Once each publication has been scored they are sorted in quartiles of study quality based on the total number of checklist items scored, with the lowest quartile scoring 1 point and the highest quartile scoring 4 points.
**Study size score [SS]**<br>"1-10 patients": 1 point<br>"11-100 patients": 2 points<br>"101-1000 patients": 3 points<br>">1000 patients": 4 points

For peer review only

Table 3: Scoring method for evaluation of study quality in clinical systematic review.

	CAMARADES	Delphi	GRADE
**Binary response items :** *Yes (1 point); No (0 points)*			
Peer reviewed publication	X		
Statement of potential conflicts of interest	X		
Sample size calculation	X	X	
Random allocation to group	X	X	X
Allocation concealment	X		X
Blinded assessment of outcome		X	
**Tertiary response items:** *Yes (1 point); No (0 points); Not clear (0.5 points)*			
Were the groups similar at baseline regarding the most important prognostic indicators?		X	
Were the eligibility criteria specified?		X	
Were point estimates and measures of variability presented for the primary outcome measures?		X	
Was there intention to treat analysis?		X	
Complete accounting of patient and outcome events			X
Non-selective outcome reporting		X	
No other limitations			X
Can we be confident in the assessment of outcome?			X
**Quinary response items:** *N/A; Definitely yes (1 point); Probably yes (0.75 points); Probably no (0.25 points); Definitely No (0 points)*			
Was selection of treatment and control groups drawn from the same population?			X
Can we be confident that patients received the allocated treatment?			X
Can we be confident that the outcome of interest was not present at start of the study?			X
Did the study stratify on variables associated with the outcome of interest or did the analysis take this into account?			X
Can we be confident in the assessment of the presence or absence of prognostic factors?			X
Was the follow up of cohorts adequate?			X
Were co-interventions similar between groups?			X

Systematic review of preclinical evidence

In parallel, we performed a systematic review of the preclinical literature (date of search 6th April 2016), focussing on publications describing the candidate interventions which had not been excluded on the basis of feasibility or plausibility, and using our previously published systematic review protocol.[23] We evaluated data from all *in vivo* models of MND and FTD including (i) mammalian models (mouse and rat), (ii) organisms with a central nervous system (*Drosophila, Caenorhabditis elegans* and Zebrafish) and (iii) multicellular eukaryotic models such as yeast. We also include data from studies using human induced pluripotent stem cells (iPSCs) derived from people with MND.

Patient and public involvement

The MND-SMART group has consulted people with MND, their families and carers via a patient and public involvement advisory group throughout the development of the trial. The group recommended using liquid investigational medicinal product formulations to enable patients with swallowing

For peer review only

difficulties including those using gastrostomy to participate and remain in the trial. This was taken into consideration by the expert panel during the drug selection process.

Expert panel review

A panel of six independent experts consisting of neurologists with expertise in translational neurology, clinical MND research, meta-analysis and clinical trials reviewed all data. The panel rated drugs as "green" (most favourable), "amber" (less favourable) and "red" (least favourable) based on biological plausibility; safety profile; and ranking by drug score from clinical literature. Drugs rated "red" for any criteria were excluded, along with drugs which had been tested in more than three previous trials in MND. Next, the panel ranked shortlisted drugs considering also preclinical evidence and practical issues such as availability in oral liquid formulation, and restricted availability without prescription (to discourage off-protocol self-medication from study participants). As this approach might not cover novel drugs or pathways that have yet to be tested clinically in neurodegenerative diseases, the panel were given flexibility to consider emerging evidence for hitherto unconsidered drugs.

RESULTS

Clinical systematic review and initial screening of candidate interventions

The PRISMA diagram for the clinical review is shown in Figure 2. Literature search in August 2011 of PubMed, ISI Web of knowledge and EMBASE, and Cochrane list of clinical trials in MS for MS-SMART identified 29500 publications. 12893 duplicates were removed, and 15232 publications did not meet the inclusion criteria. 1375 publications were included from this initial search.

In the updated search in December 2013 a further 3124 publications were identified from PubMed, ISI Web of Knowledge, EMBASE and Cochrane databases. 541 duplicates were removed, and 2322 publications did not meet the inclusion criteria. 261 publications were included.

Based on information contained in the Title and Abstract of these 1636 included publications we identified 595 interventions, of which 139 met our criteria of being described in at least one MND publication or in publications in two other diseases, in a total of 884 publications. On full text screening, 266 of these 884 publications did not meet our inclusion criteria. A further 50 interventions described in 90 publications were were excluded because it became apparent at full text screening that the intervention has not been tested either in MND or in at least two of the other diseases. The remaining 66 interventions (528 publications) were scored against our predefined criteria and ranked (Table 4). During preparation of this manuscript, we discovered that a publication describing the effect of N-acetyl cysteine in MND had been included in error, as no data were available for N-acetyl cysteine monotherapy.[24]

For peer review only

Table 4: Longlisted interventions ranked by drug score from clinical review

Intervention	Number of Publications	Quality Score	Efficacy Score	Safety Score	Study Size Score	Drug score
Rivastigmine	29	3.34	3.33	2.1	2.62	90.62
Memantine	51	3.02	2.87	2.2	2.47	80.7
Vitamin D3	11	3.27	3.01	3.36	2.18	78.08
Donepezil	41	3.1	2.72	2.51	2.24	76.99
Pramipexole	14	3.07	3.04	2.43	2.64	70.41
Galantamine	12	2.75	2.85	2.67	2.5	58.29
Amantadine	59	2.37	3	2.36	1.9	56.53
Dextromethorphan /quinidine	3	4	3.33	2.33	3	56.19
Selegiline	21	3	2.59	2.1	2.38	51.98
4-Aminopyridine	10	3.2	2.76	2.5	2.1	48.28
Acetyl-L-Carnitine	10	2.9	2.64	2.5	2.3	45.83
Simvastatin	5	3.6	2.27	2.8	2.4	42.73
Lamotrigine	6	4	2.25	2.33	2.33	41.41
Bromocriptine	13	2.92	2.4	2.54	1.92	39.21
Clozapine	6	3	2.9	2.5	2	36.8
Gabapentin	9	2.67	2.4	2.44	2.33	36.46
Creatine	12	2.67	2.14	2.33	2.42	35.87
Ginkgo biloba	3	3.67	2.61	3.67	1.67	35.21
Minocycline	11	2.45	2.27	2.64	2.18	34.54
Vitamin E	9	3.33	2.1	2	2.44	34.25
Levetiracetam	6	3	3.33	2.17	1.83	33.57
Atomoxetine	4	3.25	2.5	3.25	1.75	32.3
Coenzyme Q10	9	3.33	2.16	1.89	2.33	31.67
Tacrine	10	3.3	2.81	1.8	1.8	31.26
Olanzapine	5	2.4	3.47	2.6	1.6	26.93
Estrogen	9	3	2.66	1.67	2	26.57
Nimodipine	5	3	2.35	2.2	2.2	26.55
Riluzole	17	2.41	2.35	1.76	2.06	25.88
Ciclosporin	9	2.78	1.93	2.11	2.22	25.15
Dextromethorphan	7	2.29	2.4	2.71	1.86	24.97
Naltrexone	8	2.5	2.22	2.62	1.75	24.29
Theophylline	2	4	3.25	2.5	1.5	23.26
Valproate	9	2.56	2.33	2	1.89	22.53
Fluoxetine	6	2.67	2.42	2.33	1.67	21.18
Levamisole	3	2.67	2.78	2.33	2	20.81
Melatonin	8	2	2.11	2.75	1.88	20.81
Celecoxib	2	4	2	1.5	3	17.18
3,4-Diaminopyridine	6	2.83	2.4	2.17	1.33	16.62
Milacemide	4	3	1.75	2.25	2	16.51

N-acetyl cystine	1	3	3	3	2	16.26
Tranylcypromine	2	2.5	2.5	3.5	1.5	15.66
Aspirin	4	2.75	2	1.75	2.25	15.14
Ursodeoxycholic acid	1	4	2	3	2	14.45
Tolbutamide	2	3	2.5	2	2	14.31
Imipramine	2	3.5	2	2	2	13.36
Lithium	12	2.42	2.19	1.5	1.5	13.29
Modafinil	2	4	3.33	1	2	12.72
Omega 3 Fatty Acid	2	2.5	1.75	3	2	12.52
Octacosanol	2	2.5	2	3.5	1.5	12.52
Indinavir	2	3.5	1.8	1.5	2.5	11.27
Sodium phenylbutyrate	1	4	2	2	2	9.63
Tilorone	1	4	2	2	2	9.63
lipoic acid	2	2.5	2	2	2	9.54
Isoprinosine	4	3	1.75	1.25	2	9.17
Tetrahydrocannab inol	2	3.5	1.5	1.5	2	7.51
Topiramate	1	4	1	2	3	7.22
Haloperidol	2	3	2.33	1	1.5	5.01
Amino acid mixture	5	1.6	2	1	2	4.98
Rolipram	2	1.5	2	3	1	4.29
Alsamin	1	2	3.5	1	2	4.21
Pentoxifylline	3	2	1.61	1	2	3.88
Verapamil	1	2	2	1	2	2.41
IGF-1	1	2	1	1	3	1.81
Propranolol	1	1	3	1	2	1.81
Fluvoxamine	2	1	3	1	1	1.43
Amitriptyline	1	1	1	3	1	0.9

Preclinical systematic review

We identified 14195 publications. After removing duplicates, two independent researchers screened Title and Abstract of 7586 unique publications, with differences reconciled by a third reviewer. 396 studies were included. 330 studies reported effects on survival of the model organism and were included in the meta-analysis. Of the 66 longlisted interventions from the clinical review, 20 had been tested in in vivo models reporting an endpoint of survival (Table 5), and of these 17 had at least one report of improved survival. Because survival was reported in a single paper for 11 of these 20 interventions, we did not conduct a meta-analysis.

Table 5: Summary of preclinical studies evaluating the effect of interventions longlisted from the clinical review on survival outcomes. All listed studies used mouse models. LogMSR = log(median survival in treatment group/median survival in control group)

Publication	Drug	Total number of animals	Median survival in treatment group	Median survival in control group	logMSR
Kira 2006	Acetyl-L-Carnitine	20	270	240	0.1178
Barneoud 1999	Aspirin	38	150	155	-0.0328
Tanaka 2011	Bromocriptine	69	40	35	0.1335
Drachman 2002	Celecoxib	55	139	119	0.1554
Karlsson 2004	Ciclosporin	13	144	130	0.1023
Keep 2001	Ciclosporin	11	24	12	0.6931
Turner 2003	Clozapine	16	140	132	0.0588
Andreassen 2001	Creatine	24	155	135	0.1382
Kaddurah-Daouk 2000	Creatine	13	169	144	0.1601
Klivenyi 2004	Creatine	22	150	125	0.1823
Choi 2008	Estrogen	70	135	127	0.0611
Koschnitzky 2014	Fluoxetine	34	139	132	0.0517
Gurney 1996	Gabapentin	17	140	139	0.0072
Gurney 1996	Gabapentin	38	175	165	0.0588
Ferrante 2001	Ginkgo Biloba	20	136	125	0.0843
Fornai 2008	Lithium	20	146	117	0.2214
Gill 2009	Lithium	55	124	127	-0.0239
Pizzasegola 2009	Lithium	20	119	129	-0.0807
Dardiotis 2013	Melatonin	28	143	143	0.0000
Weishaupt 2006	Melatonin	50	137	131	0.0448
Zhang 2013	Melatonin	30	145	137	0.0568
Wang 2005	Memantine	21	130	122	0.0635
Keller 2011	Minocycline	32	147	138	0.0632
Kriz 2002	Minocycline	29	364	336	0.0800
Van Den Bosch 2002	Minocycline	14	155	130	0.1759
Zhang 2003	Minocycline	20	140	130	0.0741
Zhu 2002	Minocycline	20	135	127	0.0611
Andreassen 2000	N-acetyl cysteine	30	134	129	0.0380
Jaarsma 1998	N-acetyl cysteine	28	251	239	0.0490
Yip 2013	Omega 3	32	182	182	0.0000
Petri 2006	Sodium Phenylbutyrate	26	139	127	0.0903
Ryu 2005	Sodium Phenylbutyrate	40	145	127	0.1325
Crochemore 2009	Valproate	11	140	140	0.0000
Rouaux 2007	Valproate	36	115	110	0.0445
Sugai 2004	Valproate	17	295	265	0.1072

For peer review only

Gianfocaro 2013	Vitamin D	100	126	124	0.0160

Shortlisted candidate drugs for clinical trial

The expert panel met in 2017 and considered the clinical and preclinical research summaries for each of these 66 drugs, shortlisting 22 for further consideration. An evidence summary was compiled for each of these drugs including the following information: (1) If they had been tested in 3 or more *in vivo* MND studies, (2) the number of clinical trials in people with MND, (3) the putative target pathway, (4) feasibility for delivery via enteral tube (noting that swallowing is commonly affected in MND), (5) detailed safety information including common side effects, rare but serious side effects and requirements for monitoring, (6) published clinical studies in MND, and (7) clinical trials registered on clinicaltrials.gov. The expert panel met on 9th January 2017 and discussed the evidence for each drug. 11 drugs were excluded in the first round. Following a second round of discussions, four other drugs were excluded. Reasons for exclusion are detailed in Table 6.

Table 6: Drugs excluded following expert panel review and reasons for exclusion.

	**Drug**	**Reason for exclusion**
Excluded after Round 1	Bromocriptine	Unfavourable safety profile
	Gabapentin	>3 previous clinical trials in MND
	Creatine	>3 previous clinical trials in MND
	Clozapine	Unfavourable safety profile
	Minocycline	>3 previous clinical trials in MND
	Valproate	>3 previous clinical trials in MND
	Celecoxib	Unfavourable safety profile
	Aspirin	Poor biological plausibility
	Ginkgo biloba	Poor biological plausibility
	Lithium	>3 previous clinical trials in MND
	Amino acid mixture	>3 previous clinical trials in MND
Excluded after Round 2	Estrogen	Aggregate judgment of data presented
	Vitamin D3	
	Omega 3	
	Sodium phenylbutyrate	

For peer review only

For peer review only - http://bmjopen.bmj.com/site/about/guidelines.xhtml

The seven candidate drugs remaining were memantine, acetyl-l-carnitine, simvastatin, ciclosporin, melatonin, fluoxetine and N-acetyl cysteine. The evidence for each final shortlisted drug is summarised in Table 7 and Table 8. The panel also considered emerging evidence relating to trazodone, and following due consideration recommended memantine and trazodone as the first two investigational medicinal products for MND-SMART.

Table 7: Number of publications of clinical studies and participants according to type of disease for the final seven shortlisted drugs. AD: Alzheimer's disease; HD: Huntington's disease; MND: Motor neuron disease; MS: Multiple sclerosis; PD: Parkinson's disease.

**Drug**	**Number of publications**						**Number of participants**					
	**MND**	**AD**	**HD**	**MS**	**PD**	**Total**	**MND**	**AD**	**HD**	**MS**	**PD**	**Total**
**Acetyl-L-carnitine**	0	9	1	0	0	**10**	0	1224	10	0	0	**1234**
**Ciclosporin**	2	0	0	7	0	**9**	110	0	0	1092	0	**1202**
**Fluoxetine**	0	0	1	2	3	**6**	0	0	30	51	32	**113**
**Melatonin**	1	4	0	0	3	**8**	3	273	0	0	64	**340**
**Memantine**	1	32	2	1	15	**51**	63	11912	39	116	809	**12939**
**N-acetyl cysteine**	0	1	0	0	0	**1**	0	47	0	0	0	**47**
**Simvastatin**	0	3	0	1	1	**5**	0	469	0	307	12	**788**

Table 8: Summary of previous MND clinical trials for final shortlisted drugs. RCT: Randomised controlled trial; N: number of participants. AE: Adverse events; AR: Adverse reactions; ALC: Acetyl-l-carnitine; ALSFRS: Amyotrophic Lateral Sclerosis Functional Rating Scale; MMT: Manual Muscle Testing; MN: Motor neuron; MRSI: Magnetic Resonance Spectroscopy Imaging; MUNE: Motor unit number estimate; NAC: N-acetyl cysteine; SD: Standard deviation; SUSAR: Suspected Unexpected Severe Adverse Reaction.

**Drug**	**Publication**	**RCT**	**Number of participants (active arm: placebo arm)**	**Duration**	**Primary Outcome Measure**	**Efficacy results**	**Safety results**
**Memantine**	*De Carvalho 2010*	Y	63 (32:31)	12 months	ALSFRS and safety	Equivocal (underpowered)	No increase in AEs
	Levine 2010	N	20 (20:0)	18 months	Safety and tolerability	ALSFRS decline of -0.73 points/month (pre-treatment rate - 1.07/month)	AE: Nausea in 1 participant.
	Chan 2011	N	24 (24:0)	5 months	MUNE, MRSI, ALSFRS-R and MMT	MUNE: Significant slowing of MN loss (-12.4 ± 3.7/month in run-in phase to -5.3 ± 2.2/month in treatment phase; mean ± SD, p=0.03).	AE: similar between run-in and treatment phases

For peer review only

						Other outcomes equivocal.	
**Acetyl-l-carnitine (ALC)**	*Beghi 2013*	Y	82 (42:40)	12 months	Proportion of patients no longer self-sufficient	Significantly less treated participants loss self-sufficiency (80.9% ALC vs 97.5% placebo, p=0.0296)	No significant difference in AEs.
**Simvastatin**	Nil						
**Ciclosporin**	*Appel 1988*	Y	74 (36:38)	48 weeks	Appel ALS rating scale	Progression to 22 points equivocal in treated and untreated patients (relative risk of ciclosporin 0.991, p=0.485)<br><br>In subgroup of male patients with symptoms ≤18 months, relative risk of progression was 0.302, p=0.0205.	Significant number of expected ARs: hirsutism, headache, flushing, nausea and vomiting, tremor, anorexia and gum hyperplasia. No SUSARs.
**Melatonin**	*Weishaupt 2006*	N	31 (31:0)	12 months	Safety	ALSFRS presented without any quantitative analysis	No AEs reported or observed.
**Fluoxetine**	Nil						
**N-acetyl cysteine**	*Louwerse 1995*	Y	110 (54:56)	12 months	Survival	Non-significant trend towards improvement in survival (hazard ratio 0.74 in NAC group compared to placebo, 95% confidence interval, 0.41 to 1.33; log rank test, P=0.31)	No safety data reported

DISCUSSION

Since drugs have undergone rigorous safety and pharmacokinetic testing, drug repurposing — the use of an established drug in a novel therapeutic indication — reduces costs and barriers to clinical development. Our experience of the successful application of a systematic approach to selecting neuroprotective drugs for repurposing in MS clinical trials[14] encouraged us to use a similar approach in MND. The first part of the review assessed clinical data in MND and in other neurodegenerative diseases with potential shared pathophysiological pathways. This allowed for the identification of drugs with good central nervous system penetrance and the potential for efficacy and

For peer review only

safety in patients with neurodegenerative diseases. However, drug selection based on clinical data alone is biased towards those tested in conditions where large well-designed randomised controlled trials have been performed and where the mechanism of action may be particular to that condition. Notably, two of our top five ranked drugs were cholinesterase inhibitors licensed for Alzheimer's disease, a mechanism less relevant to MND. It was therefore important that we augment this approach with expert opinion and with preclinical data in MND and frontotemporal dementia models to provide mechanistic relevance. Taken together we have compiled evidence from clinical and preclinical data and used this to inform the selection of potential oral neuroprotective agents for clinical evaluation in people with MND. Through sequential systematic review and meta-analysis, we identified a short list of 22 candidate interventions selected from an initial set of 595 drugs.

While some identified drugs demonstrate a good safety profile and have a relevant putative target pathway in MND, others have less favourable side effects profiles or a requirement for close therapeutic monitoring (e.g. clozapine) which necessitates a higher threshold of evidence before testing in clinical trial. This highlights another advantage of our approach, in that it allows the identification of interventions that warrant further rigorous preclinical testing ("cislation"[25]) in *in vivo* or *in vitro* models of ALS, with a view to providing more robust information for efficacy to support their inclusion in future clinical trials.

Following rounds of discussion, the expert panel identified memantine as a drug to be tested in MND-SMART. Memantine is a non-competitive N-methyl-D-aspartate (NMDA) receptor antagonist used in the treatment of moderate to severe Alzheimer's disease. It was shown to significantly delay disease progression and improve survival in mouse models carrying a high copy number of $SOD1^{G93A}$. [26] Memantine has been previously tested in three MND clinical trials. A phase II double-blind placebo-controlled study of 63 patients powered to evaluate safety and tolerability did not identify any increase in adverse events.[27] There was a trend towards improvement in patients treated with memantine 20 mg/day, but no significant difference in ALSFRS-R. In a 5-month randomised double-blind study of 24 participants with ALS, there was significant slowing of spinal motor neuron loss as demonstrated on motor unit estimation testing in the high dose group (10 mg twice daily) compared to low dose (5 mg twice daily).[28] Adverse events were not reported. In a single-arm pilot study of 19 ALS patients, patients treated with riluzole and memantine had reduction in rate of ALSFRS decline and reduced CSF tau levels without any increase in adverse events.[29]

We also asked the expert panel to consider other drugs for which relevant data had only become available after the searches described here had been performed. Trazodone was nominated for consideration through this route. Trazodone is an atypical serotonin antagonist and reuptake inhibitor antidepressant. An unbiased drug screen found that trazodone inhibited Protein Kinase RNA-like endoplasmic reticulum kinase (PERK), which is pivotal to stress granule formation, a common feature of neurodegenerative diseases.[30] Inhibition of PERK was found to be beneficial in a fly model of ALS as well as in an *in vitro* neuronal assay of TDP-43 injury.[31] Furthermore, trazodone has been

For peer review only

shown to modulate the ER-stress response resulting in an improvement in survival in animal models of prion disease and FTD.[30] and to be involved in mitochondrial energy metabolism and fatty acid synthesis in animal models of Huntington's disease, and may prevent mitochondrial dysfunction in MND.[32] In a randomized double-blind placebo-controlled crossover phase II trial in 31 patients with FTD, trazodone was found to improve cognition as assessed by the neuropsychiatric inventory.[33] In trials of trazodone in Parkinson's and Alzheimer's disease, although there was no improvement in cognition, symptoms of sleep disturbance and depression were alleviated and adverse events were not increased.[34 ,35]

Limitations of this approach

The main challenge in this approach to drug selection is the ambition to base drug choice on the most contemporary evidence. Systematic reviews are time consuming, as evidenced by the interval between our updated search (2013) and expert committee consideration (2017). Furthermore, drugs with promising data in some domains would be excluded if they have been tested in only one disease other than MND; or if they have not been tested clinically despite overwhelming preclinical evidence. We excluded combination therapies, but it may be – as in the treatment of various cancers[36] and infections[37 ,38] – that engagement with multiple targets is required to achieve a substantial disease-modifying effect.

Finally, some have claimed that the literature-based systematic review approach to drug selection is intrinsically flawed because it does not take into account disease specific pathophysiology (which may be largely unknown).[39] While the three drugs tested in MS-SMART were not effective,[15] we note that two other drugs on the final MS-SMART shortlist - ibudilast[17] and lipoic acid[18] - have since shown promise in independent phase II trials. Lipoic acid has been identified again as a favourable candidate drug in a further, independent review in 2020.[40] We sought to address this issue here by considering, in addition to clinical information, data from in vivo and in vitro research. Although much successful drug repurposing has been opportunistic and serendipitous, we recognise that future efforts should include consideration of our mechanistic understanding of neurodegenerative diseases and should systemically incorporate additional target and pathway-based information.[8]

Future approaches to drug selection in MND-SMART

Ongoing rounds of drug selection for MND-SMART exploit innovations in automating literature searches, screening, and annotation, with these algorithms trained using the human efforts in the work reported here. These techniques show substantial improvements in efficiency in other fields.[41] Using the Systematic Review Facility (SyRF) (https://syrf.org.uk)[42] we have enabled a 'living' systematic review with automatic search, citation screening, identification of disease and drug, and selection of drugs meeting our criteria for the range of diseases in which studies have been performed. Because of similarities between MND and frontotemporal dementia we have included this as an additional disease of interest. Further details are extracted from full text publications of shortlisted drugs by a combination of machine and human work enabled through the SyRF platform,

For peer review only

with human monitoring of machine decisions. The incorporation of machine learning and text mining techniques substantially reduces the human effort required and makes this approach feasible in the context of timely drug selection for adaptive clinical trials.

Complementing our literature-based approach, our current platform incorporates data from additional domains, including in house *in vitro* high throughput screening using human induced pluripotent stem cell culture; pathway and network analysis; and mining of drug and trial databases. We have also sought a broader range of inputs to our expert committee such that it now includes those with experience and expertise in managing people with MND and their symptoms, and of clinical trials, translational and clinical neurology, systematic reviews, experimental drug screening, pharmacology, chemistry, and drug discovery.

CONCLUSIONS

We describe our experience in conducting a systematic, structured, unbiased and evidence-based approach to the selection of candidate drugs for evaluation in a clinical trial in MND by combining review of clinical and preclinical literature, and expert panel input. The first two drugs selected are memantine and trazodone. For future selection, we will incorporate machine learning and text mining to our systematic reviews and data from our drug discovery platform.

FUNDING

For the purpose of open access, the authors have applied a Creative Commons Attribution (CC BY) licence to any Author Accepted Manuscript version arising from this submission. MND-SMART is funded by grants from MND Scotland, My Name'5 Doddie Foundation (DOD/14/15) and specific donations to the Euan MacDonald Centre. The Chandran lab is supported by the UK Dementia Research Institute, which receives its funding from UK DRI Ltd, funded by the UK Medical Research Council, Alzheimer's Society and Alzheimer's Research UK. E.E is a clinical academic fellow jointly funded by MND Scotland (MNDS) and the Chief Scientist Office (CSO) (217ARF R45951). A.R.M. was a Lady Edith Wolfson Clinical Fellow, jointly funded by the Medical Research Council (MRC) and the Motor Neurone Disease Association (MR/R001162/1). A.Salzinger is funded by Marie Sklodowska-Curie actions Innovative Training Network (ITN). B.T.S is funded by Rowling fellowship.

CONFLICTS OF INTEREST

In the last 3 years, J. Chataway has received support from the Efficacy and Evaluation (EME) Programme, a Medical Research Council (MRC) and National Institute for Health Research (NIHR) partnership and the Health Technology Assessment (HTA) Programme (NIHR), the UK MS Society, the US National MS Society and the Rosetrees Trust. He is supported in part by the NIHR University College London Hospitals (UCLH) Biomedical Research Centre, London, UK. He has been a local principal investigator for a trial in MS funded by the Canadian MS society. A local principal investigator for commercial trials funded by: Actelion, Novartis and Roche; and has taken part in advisory boards/consultancy for Azadyne, Janssen, Merck, NervGen, Novartis and Roche.

For peer review only

CONTRIBUTIONS

Project management: Suvankar Pal, Siddharthan Chandran, and Malcolm R. Macleod.

Project administration: Charis Wong, Jenna M. Gregory, Jing Liao, Kieren Egan, Hanna M. Vesterinen, and Malcolm R. Macleod.

Conceptualisation: Jenna M. Gregory, Kieren Egan, Hanna M. Vesterinen, Siddharthan Chandran, and Malcolm R. Macleod.

Systematic searching, screening, annotation and data extraction: Charis Wong, Jenna M. Gregory, Jing Liao, Kieren Egan, Hanna M. Vesterinen, Aimal Ahmad Khan, Maarij Anwar, Caitlin Beagan, Fraser Brown, John Cafferkey, Alessandra Cardinali, Jane Yi Chiam, Claire Chiang, Victoria Collins, Joyce Dormido, Elizabeth Elliott, Peter Foley, Yu Cheng Foo, Lily Fulton-Humble, Angus B. Gane, Stella A. Glasmacher, Áine Heffernan, Kiran Jayaprakash, Nimesh Jayasuriya, Amina Kaddouri, Jamie Kiernan, Gavin Langlands, Danielle Leighton, Jiaming Liu, James Lyon, Arpan R. Mehta, Alyssa Meng, Vivienne Nguyen, Na Hyun Park, Suzanne Quigley, Yousuf Rashid, Andrea Salzinger, Bethany Shiell, Ankur Singh, Tim Soane, Alexandra Thompson, Olaf Tomala, Fergal M. Waldron, Suvankar Pal, and Malcolm R. Macleod.

Data curation: Charis Wong, Jenna M. Gregory, Jing Liao, Kieren Egan, Hanna M. Vesterinen, and Malcolm R. Macleod.

Data analysis: Charis Wong, Jenna M. Gregory, Jing Liao, Kieren Egan, Hanna M. Vesterinen, and Malcolm R. Macleod.

Methodology: Charis Wong, Jenna M. Gregory, Jing Liao, Kieren Egan, Hanna M. Vesterinen, and Malcolm R. Macleod.

Software and programming: Jing Liao.

Visualisation: Charis Wong.

In vitro drug screening: Alessandra Cardinali, and Bhuvaneish T. Selvaraj.

Expert panel: Jeremy Chataway, Robert Swingler, Peter Connick, Suvankar Pal, Siddharthan Chandran, and Malcolm R. Macleod.

Writing - original draft: Charis Wong and Jenna M. Gregory.

Writing - review & editing: Charis Wong, Jenna M. Gregory, Jing Liao, John Cafferkey, Arpan R. Mehta, Jeremy Chataway, Robert Swingler, Suvankar Pal, Siddharthan Chandran, and Malcolm R. Macleod.

All authors have read and approved the manuscript.

DATA AVAILABILITY

Supplemental data are available at https://osf.io/rzcsw/.

ETHICS APPROVAL STATEMENT

This study does not involve human participants or animal subjects. Ethical approval was not required.

For peer review only

REFERENCES

1. Westeneng HJ, Debray TPA, Visser AE, et al. Prognosis for patients with amyotrophic lateral sclerosis: development and validation of a personalised prediction model. *Lancet Neurol* 2018;**17**(5):423-33.
2. Wong C, Stavrou M, Elliott E, et al. Clinical trials in amyotrophic lateral sclerosis: a systematic review and perspective. *Brain Commun* 2021;**3**(4):fcab242.
3. European Medicines Agency. Refusal of the marketing authorisation for Alsitek (masitinib). In: Agency EM, ed., 2018.
4. European Medicines Agency. Withdrawal assessment report Radicava (International non-proprietary name: edaravone). Procedure No. EMEA/H/C/004938/0000. In: Use EMACfMPfH, ed. Amsterdam, The Netherlands, 2019.
5. Witzel S, Maier A, Steinbach R, et al. Safety and Effectiveness of Long-term Intravenous Administration of Edaravone for Treatment of Patients With Amyotrophic Lateral Sclerosis. *JAMA Neurol* 2022.
6. van den Berg LH, Sorenson E, Gronseth G, et al. Revised Airlie House consensus guidelines for design and implementation of ALS clinical trials. *Neurology* 2019;**92**(14):e1610-e23.
7. Perrin S. Preclinical research: Make mouse studies work. *Nature* 2014;**507**(7493):423-5.
8. Pushpakom S, Iorio F, Eyers PA, et al. Drug repurposing: progress, challenges and recommendations. *Nat Rev Drug Discov* 2018.
9. Giovannoni G. Cladribine to Treat Relapsing Forms of Multiple Sclerosis. *Neurotherapeutics* 2017;**14**(4):874-87.
10. The Lancet. Alemtuzumab for multiple sclerosis. *The Lancet* 2012;**380**(9856):1792.
11. Coles AJ, Twyman CL, Arnold DL, et al. Alemtuzumab for patients with relapsing multiple sclerosis after disease-modifying therapy: a randomised controlled phase 3 trial. *The Lancet* 2012;**380**(9856):1829-39.
12. Salzer J, Svenningsson R, Alping P, et al. Rituximab in multiple sclerosis: A retrospective observational study on safety and efficacy. *Neurology* 2016;**87**(20):2074-81.
13. Chalmers I, Bracken MB, Djulbegovic B, et al. How to increase value and reduce waste when research priorities are set. *Lancet* 2014;**383**(9912):156-65.
14. Vesterinen HM, Connick P, Irvine CM, et al. Drug repurposing: a systematic approach to evaluate candidate oral neuroprotective interventions for secondary progressive multiple sclerosis. *PloS one* 2015;**10**(4):e0117705.
15. Chataway J, De Angelis F, Connick P, et al. Efficacy of three neuroprotective drugs in secondary progressive multiple sclerosis (MS-SMART): a phase 2b, multiarm, double-blind, randomised placebo-controlled trial. *The Lancet Neurology*.
16. Connick P, De Angelis F, Parker RA, et al. Multiple Sclerosis-Secondary Progressive Multi-Arm Randomisation Trial (MS-SMART): a multiarm phase IIb randomised, double-blind, placebo-controlled clinical trial comparing the efficacy of three neuroprotective drugs in secondary progressive multiple sclerosis. *BMJ open* 2018;**8**(8):e021944.
17. Fox RJ, Coffey CS, Conwit R, et al. Phase 2 Trial of Ibudilast in Progressive Multiple Sclerosis. *New England Journal of Medicine* 2018;**379**(9):846-55.
18. Spain R, Powers K, Murchison C, et al. Lipoic acid in secondary progressive MS: A randomized controlled pilot trial. *Neurol Neuroimmunol Neuroinflamm* 2017;**4**(5):e374.
19. MND-SMART. MND-SMART Clinical trials for MND. Secondary MND-SMART Clinical trials for MND. www.mnd-smart.org.
20. Mehta AR, Chataway J, Pal S, et al. Trials for neurodegenerative diseases: time to innovate. *Lancet Neurol* 2021;**20**(12):984.
21. Macleod M, Wong C. ReLiSyR MND. Secondary ReLiSyR MND 2019. https://osf.io/UBMHE/.
22. Egan K VH, Macleod MR. A novel strategy to identify candidate drugs for clinical trial in Motor Neuron disease. 2019.
23. Gregory JM, Waldron FM, Soane T, et al. Protocol for a systematic review and meta-analysis of experimental models of amyotrophic lateral sclerosis. *Evidence-based Preclinical Medicine* 2016;**3**(2):e00023.
24. Vyth A, Timmer JG, Bossuyt PM, et al. Survival in patients with amyotrophic lateral sclerosis, treated with an array of antioxidants. *J Neurol Sci* 1996;**139 Suppl**:99-103.
25. Macleod M, Mohan S. Reproducibility and Rigor in Animal-Based Research. *ILAR Journal* 2019;**60**(1):17-23.
26. Wang R, Zhang D. Memantine prolongs survival in an amyotrophic lateral sclerosis mouse model. *European Journal of Neuroscience* 2005;**22**(9):2376-80.

27. de Carvalho M, Pinto S, Costa J, et al. A randomized, placebo-controlled trial of memantine for functional disability in amyotrophic lateral sclerosis. *Amyotrophic lateral sclerosis : official publication of the World Federation of Neurology Research Group on Motor Neuron Diseases* 2010;**11**(5):456-60.
28. Chan K, R T, G B, et al. C82 a randomized, double-blind, dose-ranging study of memantine in patients with amyotrophic lateral sclerosis. 22nd International Symposium on ALS/MND. *Amyotrophic Lateral Sclerosis* 2011;**12**(supp1):48.
29. Levine TD, Bowser R, Hank N, et al. A pilot trial of memantine and riluzole in ALS: correlation to CSF biomarkers. *Amyotrophic lateral sclerosis : official publication of the World Federation of Neurology Research Group on Motor Neuron Diseases* 2010;**11**(6):514-9.
30. Halliday M, Radford H, Zents KAM, et al. Repurposed drugs targeting eIF2α-P-mediated translational repression prevent neurodegeneration in mice. *Brain* 2017;**140**(6):1768-83.
31. Kim HJ, Raphael AR, LaDow ES, et al. Therapeutic modulation of eIF2alpha phosphorylation rescues TDP-43 toxicity in amyotrophic lateral sclerosis disease models. *Nat Genet* 2014;**46**(2):152-60.
32. Lauterbach EC. Neuroprotective effects of psychotropic drugs in Huntington's disease. *Int J Mol Sci* 2013;**14**(11):22558-603.
33. Lebert F, Stekke W, Hasenbroekx C, et al. Frontotemporal dementia: a randomised, controlled trial with trazodone. *Dement Geriatr Cogn Disord* 2004;**17**(4):355-9.
34. Werneck AL, Rosso AL, Vincent MB. The use of an antagonist 5-HT2a/c for depression and motor function in Parkinson' disease. *Arq Neuropsiquiatr* 2009;**67**(2B):407-12.
35. Camargos EF, Quintas JL, Louzada LL, et al. Trazodone and cognitive performance in Alzheimer disease. *J Clin Psychopharmacol* 2015;**35**(1):88-9.
36. Bayat Mokhtari R, Homayouni TS, Baluch N, et al. Combination therapy in combating cancer. *Oncotarget* 2017;**8**(23):38022-43.
37. Schmid A, Wolfensberger A, Nemeth J, et al. Monotherapy versus combination therapy for multidrug-resistant Gram-negative infections: Systematic Review and Meta-Analysis. *Scientific Reports* 2019;**9**(1):15290.
38. Arts EJ, Hazuda DJ. HIV-1 antiretroviral drug therapy. *Cold Spring Harb Perspect Med* 2012;**2**(4):a007161-a61.
39. Fox RJ. Feast or famine in multiple sclerosis therapeutics. *The Lancet Neurology*.
40. Cunniffe N, Vuong KA, Ainslie D, et al. Systematic approach to selecting licensed drugs for repurposing in the treatment of progressive multiple sclerosis. *J Neurol Neurosurg Psychiatry* 2021;**92**(3):295-302.
41. Bannach-Brown A, Przybyla P, Thomas J, et al. Machine learning algorithms for systematic review: reducing workload in a preclinical review of animal studies and reducing human screening error. *Systematic reviews* 2019;**8**(1):23.
42. Bahor Z, Liao J, Currie G, et al. Development and uptake of an online systematic review platform: the early years of the CAMARADES Systematic Review Facility (SyRF). *BMJ Open Sci* 2021;**5**(1):e100103.

For peer review only

Figure legends

Figure 1: Diagram illustrating two-stage systematic review approach to inform identification and selection of putative treatments to take forward to clinical trial.

Figure 2: PRISMA diagram for clinical systematic review. Ti/Ab: Title/Abstract

For peer review only

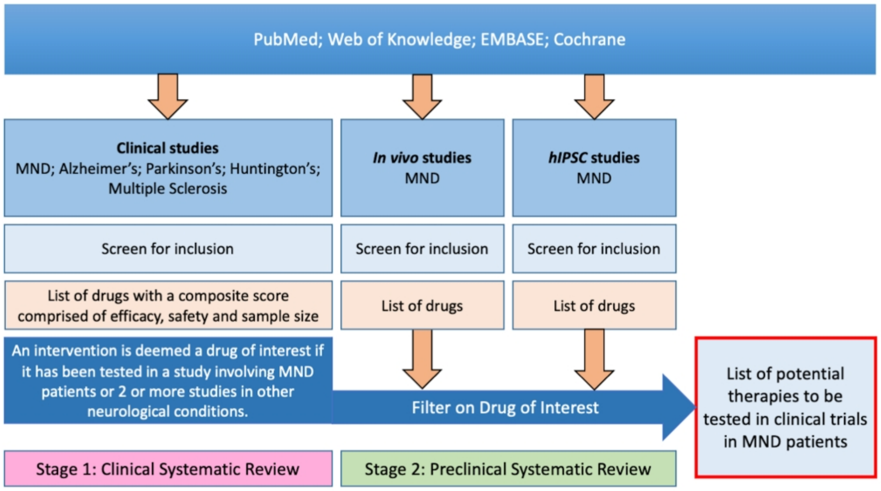

Diagram illustrating two-stage systematic review approach to inform identification and selection of putative treatments to take forward to clinical trial.

337x190mm (300 x 300 DPI)

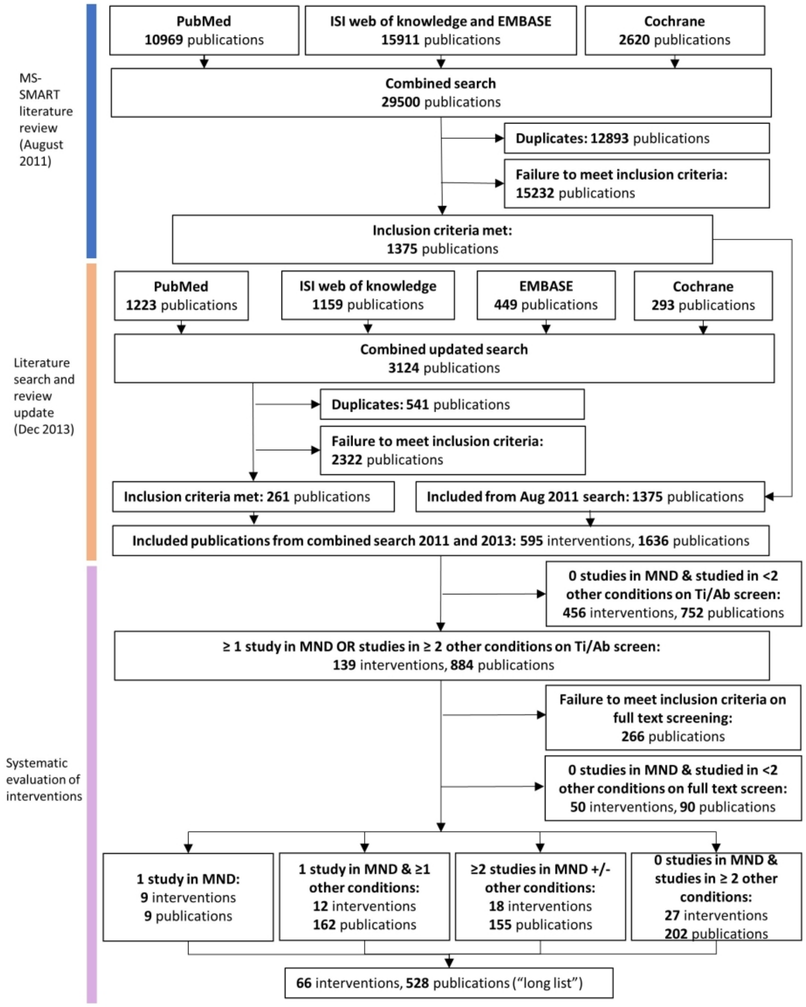

PRISMA diagram for clinical systematic review. Ti/Ab: Title/Abstract

190x275mm (300 x 300 DPI)

PRISMA 2020 Checklist

Section and Topic	Item #	Checklist item	Location where item is reported
**TITLE**			
Title	1	Identify the report as a systematic review.	NA - the paper describes a process of selecting drugs for which systematic reviews are a part of rather than the only process involved.
**ABSTRACT**			
Abstract	2	See the PRISMA 2020 for Abstracts checklist.	3<br>The abstract does not include all items in the PRISMA 2020 Abstract checklist as the paper describes a process of selecting drugs for which systematic reviews are a part of rather than the only process
**INTRODUCTION**			
Rationale	3	Describe the rationale for the review in the context of existing knowledge.	5
Objectives	4	Provide an explicit statement of the objective(s) or question(s) the review addresses.	5, 6
**METHODS**			
Eligibility criteria	5	Specify the inclusion and exclusion criteria for the review and how studies were grouped for the syntheses.	6, 7
Information sources	6	Specify all databases, registers, websites, organisations, reference lists and other sources searched or consulted to identify studies. Specify the date when each source was last searched or consulted.	6, 7
Search strategy	7	Present the full search strategies for all databases, registers and websites, including any filters and limits used.	6
Selection process	8	Specify the methods used to decide whether a study met the inclusion criteria of the review, including how many reviewers screened each record and each report retrieved, whether they worked independently, and if applicable, details of automation tools used in the process.	7
Data collection process	9	Specify the methods used to collect data from reports, including how many reviewers collected data from each report, whether they worked independently, any processes for obtaining or confirming data from study investigators, and if applicable, details of automation tools used in the process.	7
Data items	10a	List and define all outcomes for which data were sought. Specify whether all results that were compatible with each outcome domain in	7, 8

For peer review only

PRISMA 2020 Checklist

Section and Topic	Item #	Checklist item	Location where item is reported
		each study were sought (e.g. for all measures, time points, analyses), and if not, the methods used to decide which results to collect.	
	10b	List and define all other variables for which data were sought (e.g. participant and intervention characteristics, funding sources). Describe any assumptions made about any missing or unclear information.	7
Study risk of bias assessment	11	Specify the methods used to assess risk of bias in the included studies, including details of the tool(s) used, how many reviewers assessed each study and whether they worked independently, and if applicable, details of automation tools used in the process.	8, 9
Effect measures	12	Specify for each outcome the effect measure(s) (e.g. risk ratio, mean difference) used in the synthesis or presentation of results.	8
Synthesis methods	13a	Describe the processes used to decide which studies were eligible for each synthesis (e.g. tabulating the study intervention characteristics and comparing against the planned groups for each synthesis (item #5)).	6-8
	13b	Describe any methods required to prepare the data for presentation or synthesis, such as handling of missing summary statistics, or data conversions.	6-9
	13c	Describe any methods used to tabulate or visually display results of individual studies and syntheses.	6-9
	13d	Describe any methods used to synthesize results and provide a rationale for the choice(s). If meta-analysis was performed, describe the model(s), method(s) to identify the presence and extent of statistical heterogeneity, and software package(s) used.	6-9
	13e	Describe any methods used to explore possible causes of heterogeneity among study results (e.g. subgroup analysis, meta-regression).	NA
	13f	Describe any sensitivity analyses conducted to assess robustness of the synthesized results.	NA
Reporting bias assessment	14	Describe any methods used to assess risk of bias due to missing results in a synthesis (arising from reporting biases).	NA
Certainty assessment	15	Describe any methods used to assess certainty (or confidence) in the body of evidence for an outcome.	9
**RESULTS**			
Study selection	16a	Describe the results of the search and selection process, from the number of records identified in the search to the number of studies included in the review, ideally using a flow diagram.	10
	16b	Cite studies that might appear to meet the inclusion criteria, but which were excluded, and explain why they were excluded.	10
Study characteristics	17	Cite each included study and present its characteristics.	Supplemental data; We did not cite all studies in main manuscript due to volume of studies.
Risk of bias in studies	18	Present assessments of risk of bias for each included study.	Supplemental data
Results of individual studies	19	For all outcomes, present, for each study: (a) summary statistics for each group (where appropriate) and (b) an effect estimate and its precision (e.g. confidence/credible interval), ideally using structured tables or plots.	Supplemental data for study-level results. We present the drug-level results in page 10-11
Results of	20a	For each synthesis, briefly summarise the characteristics and risk of bias among contributing studies.	Supplemental data for study-

For peer review only

PRISMA 2020 Checklist

Section and Topic	Item #	Checklist item	Location where item is reported
syntheses			level results. We present the drug-level results in page 10-11
	20b	Present results of all statistical syntheses conducted. If meta-analysis was done, present for each the summary estimate and its precision (e.g. confidence/credible interval) and measures of statistical heterogeneity. If comparing groups, describe the direction of the effect.	Supplemental data for study-level results. We present the drug-level results in page 10-11
	20c	Present results of all investigations of possible causes of heterogeneity among study results.	Not applicable here – the nature of our systematic review where we include publications on different neurodegenrative diseases, using different interventions and outcomes means that we expect results to be heterogenous.
	20d	Present results of all sensitivity analyses conducted to assess the robustness of the synthesized results.	NA – see above
Reporting biases	21	Present assessments of risk of bias due to missing results (arising from reporting biases) for each synthesis assessed.	NA – see above
Certainty of evidence	22	Present assessments of certainty (or confidence) in the body of evidence for each outcome assessed.	NA – see above
**DISCUSSION**			
Discussion	23a	Provide a general interpretation of the results in the context of other evidence.	16-19
	23b	Discuss any limitations of the evidence included in the review.	16-17
	23c	Discuss any limitations of the review processes used.	18
	23d	Discuss implications of the results for practice, policy, and future research.	16-19
**OTHER INFORMATION**			
Registration and protocol	24a	Provide registration information for the review, including register name and registration number, or state that the review was not registered.	6
	24b	Indicate where the review protocol can be accessed, or state that a protocol was not prepared.	6
	24c	Describe and explain any amendments to information provided at registration or in the protocol.	6
Support	25	Describe sources of financial or non-financial support for the review, and the role of the funders or sponsors in the review.	19
Competing	26	Declare any competing interests of review authors.	19

For peer review only

PRISMA 2020 Checklist

Section and Topic	Item #	Checklist item	Location where item is reported
interests			
Availability of data, code and other materials	27	Report which of the following are publicly available and where they can be found: template data collection forms; data extracted from included studies; data used for all analyses; analytic code; any other materials used in the review.	20

From: Page MJ, McKenzie JE, Bossuyt PM, Boutron I, Hoffmann TC, Mulrow CD, et al. The PRISMA 2020 statement: an updated guideline for reporting systematic reviews. BMJ 2021;372:n71. doi: 10.1136/bmj.n71
For more information, visit: http://www.prisma-statement.org/

For peer review only

PRISMA 2020 Checklist

Section and Topic	Item #	Checklist item	Location where item is reported
**TITLE**			
Title	1	Identify the report as a systematic review.	NA - the paper describes a process of selecting drugs for which the preclinical systematic review forms a part of rather than being the only process involved.
**ABSTRACT**			
Abstract	2	See the PRISMA 2020 for Abstracts checklist.	3<br>The abstract does not include all items in the PRISMA 2020 Abstract checklist as the paper describes a process of selecting drugs for which the preclinical systematic review forms a part of rather than being the only process involved.
**INTRODUCTION**			
Rationale	3	Describe the rationale for the review in the context of existing knowledge.	5, 6
Objectives	4	Provide an explicit statement of the objective(s) or question(s) the review addresses.	5, 6

For peer review only

PRISMA 2020 Checklist

Section and Topic	Item #	Checklist item	Location where item is reported
**METHODS**			
Eligibility criteria	5	Specify the inclusion and exclusion criteria for the review and how studies were grouped for the syntheses.	9; protocol in ref(23)
Information sources	6	Specify all databases, registers, websites, organisations, reference lists and other sources searched or consulted to identify studies. Specify the date when each source was last searched or consulted.	9; protocol in ref(23)
Search strategy	7	Present the full search strategies for all databases, registers and websites, including any filters and limits used.	9; protocol in ref(23)
Selection process	8	Specify the methods used to decide whether a study met the inclusion criteria of the review, including how many reviewers screened each record and each report retrieved, whether they worked independently, and if applicable, details of automation tools used in the process.	9; protocol in ref(23)
Data collection process	9	Specify the methods used to collect data from reports, including how many reviewers collected data from each report, whether they worked independently, any processes for obtaining or confirming data from study investigators, and if applicable, details of automation tools used in the process.	9; protocol in ref(23)
Data items	10a	List and define all outcomes for which data were sought. Specify whether all results that were compatible with each outcome domain in each study were sought (e.g. for all measures, time points, analyses), and if not, the methods used to decide which results to collect.	9; protocol in ref(23)
	10b	List and define all other variables for which data were sought (e.g. participant and intervention characteristics, funding sources). Describe any assumptions made about any missing or unclear information.	9; protocol in ref(23)
Study risk of bias assessment	11	Specify the methods used to assess risk of bias in the included studies, including details of the tool(s) used, how many reviewers assessed each study and whether they worked independently, and if applicable, details of automation tools used in the process.	9; protocol in ref(23)
Effect measures	12	Specify for each outcome the effect measure(s) (e.g. risk ratio, mean difference) used in the synthesis or presentation of results.	9; protocol in ref(23)
Synthesis methods	13a	Describe the processes used to decide which studies were eligible for each synthesis (e.g. tabulating the study intervention characteristics and comparing against the planned groups for each synthesis (item #5)).	9; protocol in ref(23)
	13b	Describe any methods required to prepare the data for presentation or synthesis, such as handling of missing summary statistics, or data conversions.	9; protocol in ref(23)
	13c	Describe any methods used to tabulate or visually display results of individual studies and syntheses.	9; protocol in ref(23)
	13d	Describe any methods used to synthesize results and provide a rationale for the choice(s). If meta-analysis was performed, describe the model(s), method(s) to identify the presence and extent of statistical heterogeneity, and software package(s) used.	9; protocol in ref(23)
	13e	Describe any methods used to explore possible causes of heterogeneity among study results (e.g. subgroup analysis, meta-regression).	9; protocol in ref(23)
	13f	Describe any sensitivity analyses conducted to assess robustness of the synthesized results.	9; protocol in ref(23)
Reporting bias assessment	14	Describe any methods used to assess risk of bias due to missing results in a synthesis (arising from reporting biases).	9; protocol in ref(23)
Certainty assessment	15	Describe any methods used to assess certainty (or confidence) in the body of evidence for an outcome.	9; protocol in ref(23)
**RESULTS**			
Study selection	16a	Describe the results of the search and selection process, from the number of records identified in the search to the number of studies included	12

For peer review only - http://bmjopen.bmj.com/site/about/guidelines.xhtml

PRISMA 2020 Checklist

Section and Topic	Item #	Checklist item	Location where item is reported
		in the review, ideally using a flow diagram.	
	16b	Cite studies that might appear to meet the inclusion criteria, but which were excluded, and explain why they were excluded.	Full results not reported here as the aim of this paper is to describe a process of selecting drug, which includes use of data from the preclinical systematic reviews, rather than the describing the full preclinical systematic review.
Study characteristics	17	Cite each included study and present its characteristics.	Supplemental data
Risk of bias in studies	18	Present assessments of risk of bias for each included study.	Supplemental data
Results of individual studies	19	For all outcomes, present, for each study: (a) summary statistics for each group (where appropriate) and (b) an effect estimate and its precision (e.g. confidence/credible interval), ideally using structured tables or plots.	13, Supplemental data
Results of syntheses	20a	For each synthesis, briefly summarise the characteristics and risk of bias among contributing studies.	Supplemental data
	20b	Present results of all statistical syntheses conducted. If meta-analysis was done, present for each the summary estimate and its precision (e.g. confidence/credible interval) and measures of statistical heterogeneity. If comparing groups, describe the direction of the effect.	Page 13; Supplemental data
	20c	Present results of all investigations of possible causes of heterogeneity among study results.	Because survival was reported in a single paper for 11 of these 20 interventions, we did not conduct a

For peer review only

PRISMA 2020 Checklist

Section and Topic	Item #	Checklist item	Location where item is reported
			meta-analysis.
	20d	Present results of all sensitivity analyses conducted to assess the robustness of the synthesized results.	Because survival was reported in a single paper for 11 of these 20 interventions, we did not conduct a meta-analysis.
Reporting biases	21	Present assessments of risk of bias due to missing results (arising from reporting biases) for each synthesis assessed.	Because survival was reported in a single paper for 11 of these 20 interventions, we did not conduct a meta-analysis.
Certainty of evidence	22	Present assessments of certainty (or confidence) in the body of evidence for each outcome assessed.	Because survival was reported in a single paper for 11 of these 20 interventions, we did not conduct a meta-analysis.
**DISCUSSION**			
Discussion	23a	Provide a general interpretation of the results in the context of other evidence.	16-18
	23b	Discuss any limitations of the evidence included in the review.	16, 17
	23c	Discuss any limitations of the review processes used.	18
	23d	Discuss implications of the results for practice, policy, and future research.	17-18
**OTHER INFORMATION**			
Registration and	24a	Provide registration information for the review, including register name and registration number, or state that the review was not registered.	9, ref 23

For peer review only

For peer review only - http://bmjopen.bmj.com/site/about/guidelines.xhtml

PRISMA 2020 Checklist

Section and Topic	Item #	Checklist item	Location where item is reported
protocol	24b	Indicate where the review protocol can be accessed, or state that a protocol was not prepared.	6, 9
	24c	Describe and explain any amendments to information provided at registration or in the protocol.	6
Support	25	Describe sources of financial or non-financial support for the review, and the role of the funders or sponsors in the review.	19
Competing interests	26	Declare any competing interests of review authors.	19
Availability of data, code and other materials	27	Report which of the following are publicly available and where they can be found: template data collection forms; data extracted from included studies; data used for all analyses; analytic code; any other materials used in the review.	20

From: Page MJ, McKenzie JE, Bossuyt PM, Boutron I, Hoffmann TC, Mulrow CD, et al. The PRISMA 2020 statement: an updated guideline for reporting systematic reviews. BMJ 2021;372:n71. doi: 10.1136/bmj.n71

For more information, visit: http://www.prisma-statement.org/

For peer review only

BMJ Open

A systematic, comprehensive, evidence-based approach to identify neuroprotective interventions for motor neuron disease: using systematic reviews to inform expert consensus

Journal:	*BMJ Open*
Manuscript ID	bmjopen-2022-064169.R1
Article Type:	Original research
Date Submitted by the Author:	19-Dec-2022
Complete List of Authors:	Wong, Charis; The University of Edinburgh Centre for Clinical Brain Sciences, Anne Rowling Regenerative Neurology Clinic; The University of Edinburgh Centre for Clinical Brain Sciences, Euan MacDonald Centre for Motor Neuron Disease Research<br>Gregory, Jenna M.; The University of Edinburgh Centre for Clinical Brain Sciences; University of Aberdeen Institute of Medical Sciences<br>Liao, Jing; The University of Edinburgh Centre for Clinical Brain Sciences<br>Egan, Kieren ; University of Strathclyde, Computer and Information Science; The University of Edinburgh Centre for Clinical Brain Sciences<br>Vesterinen, Hanna M.; The University of Edinburgh Centre for Clinical Brain Sciences<br>Ahmad Khan, Aimal; The University of Edinburgh Edinburgh Medical School<br>Anwar, Maarij; The University of Edinburgh Edinburgh Medical School<br>Beagan, Caitlin; The University of Edinburgh Edinburgh Medical School<br>Brown, Fraser; The University of Edinburgh Centre for Clinical Brain Sciences; The University of Edinburgh Centre for Clinical Brain Sciences, Anne Rowling Regenerative Neurology Clinic<br>Cafferkey, John; NHS Lothian, Royal Infirmary of Edinburgh, NHS Lothian, Edinburgh, UK<br>Cardinali, Alessandra; The University of Edinburgh Centre for Clinical Brain Sciences, Euan MacDonald Centre for Motor Neuron Disease Research; UK Dementia Research Institute, University of Edinburgh<br>Chiam, Jane Yi; The University of Edinburgh Edinburgh Medical School<br>Chiang, Claire; The University of Edinburgh Edinburgh Medical School<br>Collins, Victoria; The University of Edinburgh Edinburgh Medical School<br>Dormido, Joyce; NHS Borders<br>Elliott, Elizabeth; The University of Edinburgh Centre for Clinical Brain Sciences, Anne Rowling Regenerative Neurology Clinic; The University of Edinburgh Centre for Clinical Brain Sciences, Euan MacDonald Centre for Motor Neuron Disease Research<br>Foley, Peter; The University of Edinburgh Centre for Clinical Brain Sciences, Anne Rowling Regenerative Neurology Clinic<br>Foo, Yu Cheng; The University of Edinburgh Edinburgh Medical School<br>Fulton-Humble, Lily; University of Aberdeen, Institute of Medical Sciences<br>Gane, Angus; The University of Edinburgh College of Medicine and Veterinary Medicine

	Glasmacher, Stella; The University of Edinburgh Centre for Clinical Brain Sciences, Anne Rowling Regenerative Neurology Clinic; The University of Edinburgh Centre for Clinical Brain Sciences, Euan MacDonald Centre for Motor Neuron Disease Research<br>Heffernan, Áine; UK Dementia Research Institute, University of Edinburgh<br>Jayaprakash, Kiran; The University of Edinburgh Centre for Clinical Brain Sciences, Anne Rowling Regenerative Neurology Clinic; The University of Edinburgh Centre for Clinical Brain Sciences<br>Jayasuriya, Nimesh; The University of Edinburgh College of Medicine and Veterinary Medicine; NHS Lothian<br>Kaddouri, Amina; The University of Edinburgh Edinburgh Medical School<br>Kiernan, Jamie; The University of Edinburgh Edinburgh Medical School<br>Langlands, Gavin; NHS Greater Glasgow and Clyde<br>Leighton, D; The University of Edinburgh Centre for Clinical Brain Sciences; University of Glasgow, School of Psychology and Neuroscience<br>Liu, Jiaming; The University of Edinburgh Edinburgh Medical School<br>Lyon, James; NHS Lothian<br>Mehta, Arpan; The University of Edinburgh Centre for Clinical Brain Sciences, Anne Rowling Regenerative Neurology Clinic; The University of Edinburgh Centre for Clinical Brain Sciences, Euan MacDonald Centre for Motor Neuron Disease Research<br>Meng, Alyssa; The University of Edinburgh Centre for Discovery Brain Sciences<br>Nguyen, Vivienne; The University of Edinburgh Edinburgh Medical School<br>Park, Na Hyun; NHS Lothian<br>Quigley, Suzanne; The University of Edinburgh Centre for Clinical Brain Sciences, Anne Rowling Regenerative Neurology Clinic; The University of Edinburgh Centre for Clinical Brain Sciences<br>Rashid, Yousuf; The University of Edinburgh Edinburgh Medical School<br>Salzinger, Andrea; The University of Edinburgh Centre for Clinical Brain Sciences; UK Dementia Research Institute, University of Edinburgh<br>Shiell, Bethany; The University of Edinburgh College of Medicine and Veterinary Medicine<br>Singh, Ankur; The University of Edinburgh College of Medicine and Veterinary Medicine<br>Soane, Tim; NHS Forth Valley<br>Thompson, Alexandra; The University of Edinburgh College of Medicine and Veterinary Medicine<br>Tomala, Olaf; The University of Edinburgh Edinburgh Medical School<br>Waldron, Fergal; University of Aberdeen, Institute of Medical Sciences; The University of Edinburgh Institute of Evolutionary Biology<br>Selvaraj, Bhuvaneish; The University of Edinburgh Centre for Clinical Brain Sciences, Anne Rowling Regenerative Neurology Clinic; The University of Edinburgh Centre for Clinical Brain Sciences, Euan MacDonald Centre for Motor Neuron Disease Research<br>Chataway, Jeremy; UCL Queen Square Institute of Neurology, Queen Square Multiple Sclerosis Centre, Department of Neuroinflammation,; National Institute for Health Research, University College London Hospitals, Biomedical Research Centre<br>Swingler, Robert; The University of Edinburgh Centre for Clinical Brain Sciences, Euan MacDonald Centre for Motor Neuron Disease Research<br>Connick, Peter; The University of Edinburgh Centre for Clinical Brain Sciences; The University of Edinburgh Centre for Clinical Brain Sciences, Anne Rowling Regenerative Neurology Clinic<br>Pal, Suvankar; The University of Edinburgh Centre for Clinical Brain Sciences, Anne Rowling Regenerative Neurology Clinic; The University of Edinburgh Centre for Clinical Brain Sciences, Euan MacDonald Centre for Motor Neuron Disease Research<br>Chandran, Siddharthan ; The University of Edinburgh Centre for Clinical Brain Sciences, Anne Rowling Regenerative Neurology Clinic; UK

	Dementia Research Institute, University of Edinburgh<br>Macleod, Malcolm; The University of Edinburgh Centre for Clinical Brain Sciences
**Primary Subject Heading**:	Neurology
Secondary Subject Heading:	Research methods, Evidence based practice
Keywords:	Clinical trials < THERAPEUTICS, Motor neurone disease < NEUROLOGY, Adult neurology < NEUROLOGY, NEUROLOGY, THERAPEUTICS

SCHOLARONE™
Manuscripts

I, the Submitting Author has the right to grant and does grant on behalf of all authors of the Work (as defined in the below author licence), an exclusive licence and/or a non-exclusive licence for contributions from authors who are: i) UK Crown employees; ii) where BMJ has agreed a CC-BY licence shall apply, and/or iii) in accordance with the terms applicable for US Federal Government officers or employees acting as part of their official duties; on a worldwide, perpetual, irrevocable, royalty-free basis to BMJ Publishing Group Ltd ("BMJ") its licensees and where the relevant Journal is co-owned by BMJ to the co-owners of the Journal, to publish the Work in this journal and any other BMJ products and to exploit all rights, as set out in our licence.

The Submitting Author accepts and understands that any supply made under these terms is made by BMJ to the Submitting Author unless you are acting as an employee on behalf of your employer or a postgraduate student of an affiliated institution which is paying any applicable article publishing charge ("APC") for Open Access articles. Where the Submitting Author wishes to make the Work available on an Open Access basis (and intends to pay the relevant APC), the terms of reuse of such Open Access shall be governed by a Creative Commons licence – details of these licences and which Creative Commons licence will apply to this Work are set out in our licence referred to above.

Other than as permitted in any relevant BMJ Author's Self Archiving Policies, I confirm this Work has not been accepted for publication elsewhere, is not being considered for publication elsewhere and does not duplicate material already published. I confirm all authors consent to publication of this Work and authorise the granting of this licence.

For peer review only

TITLE

A systematic, comprehensive, evidence-based approach to identify neuroprotective interventions for motor neuron disease: using systematic reviews to inform expert consensus

AUTHORS

The Writing Committee for Repurposing Living Systematic Review – Motor Neuron Disease (ReLiSyR-MND)

Charis Wong[1,2,3], Jenna M. Gregory[1,3,4], Jing Liao[1], Kieren Egan[1], Hanna M. Vesterinen[1], Aimal Ahmad Khan[5], Mohammed Maarij Anwar[5], Caitlin Beagan[6], Fraser Brown[1,2], John Cafferkey[7], Alessandra Cardinali[1,3,8], Jane Yi Chiam[5], Claire Chiang[6], Victoria Collins[6], Joyce Dormido[9], Elizabeth Elliott[1,2,3], Peter Foley[1,2], Yu Cheng Foo[5], Lily Fulton-Humble[4], Angus B. Gane[6], Stella A. Glasmacher[1,2], Áine Heffernan[8], Kiran Jayaprakash[1,2], Nimesh Jayasuriya[6,10], Amina Kaddouri[5], Jamie Kiernan[5], Gavin Langlands[11], Danielle Leighton[1,3,12], Jiaming Liu[5], James Lyon[10], Arpan R. Mehta[1,2,3,8], Alyssa Meng[13], Vivienne Nguyen[5], Na Hyun Park[10], Suzanne Quigley[1,2], Yousuf Rashid[5], Andrea Salzinger[1,8], Bethany Shiell[6], Ankur Singh[6], Tim Soane[14], Alexandra Thompson[5], Olaf Tomala[5], Fergal M. Waldron[4,15], Bhuvaneish T. Selvaraj[1,2,3,8], Jeremy Chataway[16,17,18], Robert Swingler[3], Peter Connick[1,2], Suvankar Pal[1,2,3], Siddharthan Chandran[1,2,3,8], Malcolm R. Macleod[1]

[1]Centre for Clinical Brain Sciences, University of Edinburgh, Edinburgh, UK, [2]Anne Rowling Regenerative Neurology Clinic, University of Edinburgh, UK, [3]Euan MacDonald Centre for Motor Neuron Disease Research, University of Edinburgh, Edinburgh, UK, [4]Institute of Medical Sciences, University of Aberdeen, Aberdeen, UK, [5]Edinburgh Medical School, University of Edinburgh, Edinburgh, UK, [6]University of Edinburgh, Edinburgh, UK, [7]Royal Infirmary of Edinburgh, NHS Lothian, Edinburgh, UK, [8]UK Dementia Research Institute at University of Edinburgh, University of Edinburgh, Edinburgh bioQuarter, Chancellor's Building, 49 Little France Crescent, Edinburgh, UK, [9]NHS Borders and Midlothian, UK, [10]NHS Lothian, Edinburgh, UK, [11]NHS Greater Glasgow and Clyde, Glasgow, UK, [12]School of Psychology and Neuroscience, University of Glasgow, Glasgow, UK, [13]Centre for Discovery Brain Sciences, University of Edinburgh, Edinburgh, UK, [14]NHS Forth Valley, Larbert, UK, [15]Institute of Evolutionary Biology, University of Edinburgh, Edinburgh, UK, [16]Queen Square Multiple Sclerosis Centre, Department of Neuroinflammation, UCL Queen Square Institute of Neurology, Faculty of Brain Sciences, University College London, London, UK, [17]National Institute for Health Research, University College London Hospitals, Biomedical Research Centre, London, UK, [18]Medical Research Council Clinical Trials Unit at UCL, Institute of Clinical Trials and Methodology, University College London, London, UK

Corresponding Author:

Malcolm R. Macleod

Address: Centre for Clinical Brain Sciences, University of Edinburgh, Edinburgh, UK.

Email: malcolm.macleod@ed.ac.uk

Word count: 3956 words

ORCID iD

Charis Wong 0000-0002-8488-037X
Jenna M. Gregory 0000-0003-3337-4079
Jing Liao 0000-0002-9591-8070
Kieren Egan 0000-0002-1639-4281
Mohammed Maarij Anwar 0000-0002-7136-8342
Fraser Brown 0000-0002-8314-4936
John Cafferkey 0000-0001-6926-9508
Elizabeth Elliott 0000-0002-8207-741X
Peter Foley 0000-0001-7039-2976
Nimesh Jayasuriya 0000-0002-0688-3161
Gavin Langlands 0000-0002-8972-4798
Danielle Leighton 0000-0003-2015-7198
Arpan R. Mehta 0000-0002-5715-5025
Na Hyun Park 0000-0003-4674-6767
Fergal M. Waldron 0000-0003-2622-0776
Bhuvaneish T. Selvaraj 0000-0001-7299-0910
Jeremy Chataway 0000-0001-7286-6901
Peter Connick 0000-0002-3892-8037
Suvankar Pal 0000-0003-4276-639X
Siddharthan Chandran 0000-0001-6827-1593
Malcolm R. Macleod 0000-0001-9187-9839

For peer review only

ABSTRACT

Objectives:

Motor neuron disease (MND) is an incurable progressive neurodegenerative disease with limited treatment options. There is a pressing need for innovation in identifying therapies to take to clinical trial. Here we detail a systematic and structured evidence-based approach to inform consensus decision making to select the first two drugs for evaluation in Motor Neuron Disease – Systematic Multi-arm Adaptive Randomised Trial (MND-SMART: NCT04302870), an adaptive platform trial. We aim to identify and prioritise candidate drugs which have the best available evidence for efficacy, acceptable safety profiles and are feasible for evaluation within the trial protocol.

Methods:

We conducted a two-stage systematic review to identify potential neuroprotective interventions. First, we reviewed clinical studies in MND, Alzheimer's disease, Huntington's disease, Parkinson's disease, and multiple sclerosis, identifying drugs described in at least one MND publication or publications in two or more other diseases. We scored and ranked drugs using a metric evaluating safety, efficacy, study size and study quality. In stage two, we reviewed efficacy of drugs in MND animal models, multicellular eukaryotic models and human induced pluripotent stem cell studies. An expert panel reviewed candidate drugs over two shortlisting rounds and a final selection round, considering the systematic review findings, late breaking evidence, mechanistic plausibility, safety, tolerability, and feasibility of evaluation in MND-SMART.

Results:

From the clinical review, we identified 595 interventions. 66 drugs met our drug/disease logic. Of these, 22 drugs with supportive clinical and preclinical evidence were shortlisted at round 1. Seven drugs proceeded to round 2. The panel reached a consensus to evaluate memantine and trazodone as the first two arms of MND-SMART.

Discussion:

For future drug selection, we will incorporate automation tools, text-mining and machine learning techniques to the systematic reviews and consider data generated from other domains, including high-throughput phenotypic screening of human induced pluripotent stem cells.

Keywords

Motor neuron disease, amyotrophic lateral sclerosis, systematic review, evidence synthesis, clinical trials

For peer review only

STRENGTHS AND LIMITATIONS OF THIS STUDY:

- We describe a systematic, structured, and evidence-based, consensus approach for drug repurposing in motor neuron disease (MND), specifically for Motor Neuron Disease – Systematic Multi-arm Adaptive Randomised Trial (MND-SMART), a phase III multi-arm multi-stage adaptive clinical trial in MND.
- Systematic reviews of clinical studies in neurodegenerative diseases and MND preclinical studies provide a robust evidence base to inform expert panel decisions on drug selection for clinical trial.
- Providing a contemporary evidence base using traditional systematic reviews is challenging given their time-consuming and labour-intensive nature.
- Incorporation of machine learning and automation tools for systematic reviews, and data from experimental drug screening can be helpful for future drug selection.

For peer review only

INTRODUCTION

Motor neuron disease (MND), also known as amyotrophic lateral sclerosis (ALS), is a progressive neurodegenerative disease with a median survival of 2-3 years.[1] Despite many promising preclinical studies and 125 phase II and phase III trials reported between 2008 and 2019, riluzole remains the only globally approved disease-modifying treatment, prolonging survival by an average of two to three months.[2] Edavarone, masitinib, AMX0035 (sodium phenylbutyrate and taursodeoxycholic acid) and tofersen have emerged as potentially promising candidates in clinical trials, but treatment effects are modest and none of these drugs have received approval in Europe.[3-7] In a long-term multi-centre prospective cohort study, edaravone showed no significant disease-modifying effect.[8] Previously, decisions to evaluate drugs in MND have been informed by preclinical studies, typically using mouse models, such as the $SOD1^{G93A}$ mouse, despite known limitations in the extent to which such models recapitulate human pathology,[9] and concerns of the reproducibility of findings from such models.[10] Clinical trials in MND are further complicated by the challenges of designing and delivering trials in a rapidly progressive, heterogeneous, disabling and fatal disease with a lack of reliable and sensitive outcome measures or biomarkers.[2]

Over the same period there have, however, been rapid technical advances in MND genomics, human induced pluripotent stem cells and gene-editing, which have enabled better understanding of underlying pathophysiology (including potential shared pathways across neurodegenerative diseases), and the development of more sophisticated disease models. In parallel, drug repurposing (testing a drug already used or tested for other indications) has been successfully adopted in many diseases and can significantly reduce development time and cost, with the added benefit of the availability of prior safety data to guide selection.[11] In relapsing-remitting multiple sclerosis for instance, dimethyl fumarate, cladribine,[12], alemtuzumab,[13 ,14] and rituximab[15] provide examples of successful repurposing as disease-modifying treatments.

Systematic review has been recommended to have a key role in planning new research studies.[16] We previously used a strategy based on systematic review to identify repurposed interventions for secondary progressive multiple sclerosis (MS). This involved a two-stage systematic review and meta-analysis assessing clinical and preclinical data to identify putative therapeutic interventions[17] and led to the Multiple Sclerosis – Secondary Progressive Multi-Arm Randomisation Trial (MS-SMART), a phase IIb multi-arm randomised controlled trial.[18 ,19] The three drugs selected for MS-SMART were based in part on their availability for investigator-led clinical trials and did not show efficacy, but two of the top seven drugs thus identified, ibudilast (ranked first), and lipoic acid, have since shown promise in phase II studies in secondary progressive MS.[20 ,21]

Noting similarities between MS and MND as neurodegenerative diseases with limited treatment options, in 2014 we embarked on a similar strategy to identify candidate oral neuroprotective agents in MND. In parallel, we developed the multi-arm multi-stage Motor Neuron Disease – Systematic Multi-Arm Adaptive Randomised Trial (MND-SMART, clinicaltrials.gov registration number:

For peer review only

NCT04302870) to provide a more efficient pipeline to evaluate drugs in MND than conventional standalone two-arm trials.[22-24] Here we describe the development and implementation of a systematic, structured, and unbiased evidence-based approach to inform expert consensus in the selection of potential oral neuroprotective agents for clinical evaluation in MND-SMART. Specifically, the purpose here is not to provide a contemporary summary of existing evidence, but to describe the process through which clinical trial drugs were selected.

METHODS

The work was guided by a systematic review protocol. Over the duration of the project and given the novelty of this approach, this protocol was updated in the light of accumulating experience, and the complete record of the protocol, including the changes made, is available at Open Science Framework.[25]

Overview

The overall drug selection strategy is characterised in Figure 1. We used systematic review to identify publications describing clinical trials or reports of the clinical use of drugs in MND and in four other neurodegenerative diseases which we considered might share pivotal pathways: Alzheimer's disease (AD), Parkinson's disease (PD), Huntington's disease (HD) and MS. For MS, we excluded studies of relapsing-remitting disease since we were interested in drugs tested in the progressive phase where neurodegeneration is a major feature. We also excluded studies of other diseases of motor neurons including Kennedy's disease and spinal muscle atrophy. We annotated publications for the drugs tested and diseases studied, taking forward drugs described in at least one MND publication or in publications in at least two other diseases. We scored each drug using a predefined framework evaluating efficacy, safety, study size and quality. In parallel, we performed a systematic review of the preclinical MND and frontotemporal dementia (FTD; because of pathological overlap with MND) literature for these drugs. We summarised evidence from both reviews for each drug and presented these to an expert panel consisting of clinical and academic neurologists with expertise in MND, clinical trials, pharmacology, and preclinical models of MND.

Systematic review of clinical evidence

The MS-SMART drug selection process used the same strategy, except we selected drugs tested at least once in MS or in at least two other conditions. The protocol for and results from this search, conducted in September 2011, has been published.[17] That search involved three online databases (PubMed, ISI Web of Knowledge and EMBASE) using the terms “multiple sclerosis” OR “Alzheimer's disease” OR “Huntington's disease” OR “Parkinson's disease” OR “motor neuron disease” OR “amyotrophic lateral sclerosis”. On 13 December 2013, we updated the search using the same terms but with limitations for PubMed to clinical trials, and date of record creation after 01/07/2011; for ISI Web of Knowledge to Document type “Clinical trial” and publication years: 2011, 2012 and 2013; for EMBASE: previous search string AND (“case series” or “case report” or “cohort study”), with limits: human studies, full text studies from 2011; and we also contacted the Cochrane Neuromuscular

For peer review only

review group to obtain a list of interventions tested in MND/ALS. The protocol of this update was stored locally; in the light of increasing recognition of the importance of making systematic review protocols available, the protocol was published without amendment in September 2019.[26]

Two reviewers (MM and KE) independently screened title and abstracts of publications identified in the new search against the inclusion and exclusion criteria (Table 1) with discrepancies resolved by discussion. We included case reports, uncontrolled case series, non-randomised parallel group studies, crossover studies, and randomised controlled trials with any report of safety or efficacy. We extracted basic information from each publication including author, year of publication, intervention tested and disease.

Table 1: Eligibility criteria for clinical systematic review.

**Inclusion criteria**
- Publications reporting qualitative or quantitative data provided on either safety or efficacy of an orally delivered intervention in people with motor neuron disease / amyotrophic lateral sclerosis, Alzheimer's disease, Parkinson's disease, Huntington's disease or multiple sclerosis<br><br>- Studies reporting change in clinical status (including death, tracheostomy free survival, relapse frequency, disability progression, behavioural symptoms), or changes in biomarkers (including magnetic resonance imaging, blood, cerebrospinal fluid and muscle strength)
**Exclusion criteria**
- Isolated reporting of non-pharmacological interventions such as acupuncture, aromatherapy, physiotherapy or exercise<br>- Articles reporting the use of interventions already licensed for clinical use in MND such as riluzole<br>- Articles on levodopa treatment for Parkinson's disease<br>- Studies reporting different modes of intervention delivery other than oral administration<br>- Publications reporting secondary analysis of previously published clinical trial data<br>- Protocols for clinical trials<br>- Preventative studies<br>- Reviews<br>- Studies on healthy volunteers<br>- Studies in patients with relapsing-remitting MS<br>- Studies reporting combination treatments including where an oral and a non-oral intervention are administered<br>- Publications where disease type is not specified to be in keeping with the included diseases (studies of vascular dementia, mild cognitive impairment and dementias other than Alzheimer's disease are excluded<br>- Studies on patients with parkinsonism are excluded as this do not imply Parkinson's disease exclusively)<br>- Publications describing studies where multiple drugs were tested in a cohort without any data on individual drugs

For peer review only

For all candidate interventions which had not been excluded based on feasibility or plausibility, we extracted further information on safety, efficacy, quality of study and study size from publications to a Microsoft Access database and scored these against a predefined metric (Table 2 and Table 3). For each drug, we calculated an overall drug score by taking the product of the mean score in each domain for safety, efficacy, quality, study size and multiplying this by $\log_{10}$(1+number of publications). We then ranked drugs according to these scores.

Table 2: Scoring metric for clinical review

**Safety Score [S]**<br>“not described”: 1 point<br>“SUSARs (Suspected Unexpected Serious Adverse Reactions) or mortality observed”: 1 point<br>“SAEs (Serious Adverse Events) only”: 2 points<br>“AEs (Adverse events) only”: 3 points<br>“No adverse effects reported”: 4 points
**Efficacy Score [E]**<br>***Efficacy score is assigned based on primary outcome measure, and where this is not identified, on the mean efficacy score for all outcomes reported in each publication.***<br>“not presented”: 1 point<br>“definite (i.e. statistically significant) worsening”: 1 point<br>“neutral”: 2 points<br>“non-significant improvement”: 3 points<br>“significant improvement”: 4 points
**Quality Score [Q]**<br>Study quality was assessed using a combination of criteria taken from a risk of bias tool developed through a Delphi process, GRADE and CAMARADES methods as shown in Table 3. Once each publication has been scored they are sorted in quartiles of study quality based on the total number of checklist items scored, with the lowest quartile scoring 1 point and the highest quartile scoring 4 points.
**Study size score [SS]**<br>“1-10 patients”: 1 point<br>“11-100 patients”: 2 points<br>“101-1000 patients”: 3 points<br>“>1000 patients”: 4 points

For peer review only

Table 3: Scoring method for evaluation of study quality in clinical systematic review.

	CAMARADES	Delphi	GRADE
**Binary response items :** *Yes (1 point); No (0 points)*			
Peer reviewed publication	X		
Statement of potential conflicts of interest	X		
Sample size calculation	X	X	
Random allocation to group	X	X	X
Allocation concealment	X		X
Blinded assessment of outcome		X	
**Tertiary response items:** *Yes (1 point); No (0 points); Not clear (0.5 points)*			
Were the groups similar at baseline regarding the most important prognostic indicators?		X	
Were the eligibility criteria specified?		X	
Were point estimates and measures of variability presented for the primary outcome measures?		X	
Was there intention to treat analysis?		X	
Complete accounting of patient and outcome events			X
Non-selective outcome reporting		X	
No other limitations			X
Can we be confident in the assessment of outcome?			X
**Quinary response items:** *N/A; Definitely yes (1 point); Probably yes (0.75 points); Probably no (0.25 points); Definitely No (0 points)*			
Was selection of treatment and control groups drawn from the same population?			X
Can we be confident that patients received the allocated treatment?			X
Can we be confident that the outcome of interest was not present at start of the study?			X
Did the study stratify on variables associated with the outcome of interest or did the analysis take this into account?			X
Can we be confident in the assessment of the presence or absence of prognostic factors?			X
Was the follow up of cohorts adequate?			X
Were co-interventions similar between groups?			X

Systematic review of preclinical evidence

In parallel, we performed a systematic review of the preclinical literature (date of search 6th April 2016), focussing on publications describing the candidate interventions which had not been excluded on the basis of feasibility or plausibility, and using our previously published systematic review protocol.[27] We evaluated data from all *in vivo* models of MND and FTD including (i) mammalian models (mouse and rat), (ii) organisms with a central nervous system (*Drosophila, Caenorhabditis elegans* and Zebrafish) and (iii) multicellular eukaryotic models such as yeast. We also include data from studies using human induced pluripotent stem cells (iPSCs) derived from people with MND.

Patient and public involvement

The MND-SMART group has consulted people with MND, their families and carers via a patient and public involvement advisory group throughout the development of the trial. They expressed enthusiasm for a study design that enables definitive testing of drugs with promising efficacy, broad

For peer review only

inclusion criteria, and design features which minimise participant burden including remote study assessments, non-invasive outcome measures, liquid medication that can be administered in more advanced stages of disease, and drugs with favourable safety and tolerability profiles. This was taken into consideration by the expert panel during the drug selection process.

Shortlisting of drugs

Drugs with supportive evidence from both clinical and preclinical literature were shortlisted for review by an expert panel over two shortlisting rounds and a final selection round. Over the two shortlisting rounds, the panel rated drugs as “green” (most favourable), “amber” (less favourable) and “red” (least favourable) based on biological plausibility; safety profile; and data from the clinical and preclinical reviews, and logistical considerations relating to factors including drug manufacturing, storage, dosing schedule, and route of administration. Drugs rated “red” for any criteria were excluded, along with drugs which had been tested in more than three previous trials in MND. Remaining drugs after the second shortlisting round entered a final selection round. We hand searched literature to identify and summarise all MND clinical trials for shortlisted drugs, including trials which may have been missed in the original search, trials which were annotated using drug synonyms in the original review (e.g. acetylcysteine/acetylcystine/N-acetyl cystine/N-acetylcysteine for N-acetyl cysteine), and trials which have been excluded in the clinical review but contain relevant data for expert panel discussions, such as drugs given in combination with other treatments or drugs given in non-oral formulations. We presented the expert panel with clinical, preclinical and clinical trial summaries for the final shortlisted drugs. Members of the expert panel independently ranked shortlisted drugs. The expert panel then met to finalise selection of drugs for clinical trial. As this approach might not cover novel drugs or pathways that had yet to be tested clinically in neurodegenerative diseases, the panel were given flexibility to consider emerging evidence for hitherto unconsidered drugs.

RESULTS

Clinical systematic review and initial screening of candidate interventions

The PRISMA diagram for the clinical review is shown in Figure 2. Further data are available in Supplementary File 1. Literature search in August 2011 of PubMed, ISI Web of knowledge and EMBASE, and Cochrane list of clinical trials in MS for MS-SMART identified 29500 publications. 12893 duplicates were removed, and 15232 publications did not meet the inclusion criteria. 1375 publications were included from this initial search.

In the updated search in December 2013 a further 3124 publications were identified from PubMed, ISI Web of Knowledge, EMBASE and Cochrane databases. 541 duplicates were removed, and 2322 publications did not meet the inclusion criteria. 261 publications were included.

Based on information contained in the Title and Abstract of these 1636 included publications we identified 595 interventions, of which 139 met our criteria of being described in at least one MND publication or in publications in two other diseases, in a total of 884 publications. On full text

screening, 266 of these 884 publications did not meet our inclusion criteria. A further 50 interventions described in 90 publications were excluded because more detailed review of the primary literature at full text screening showed that the intervention has not been tested either in MND or in at least two of the other diseases. The remaining 66 interventions (528 publications) were scored against our predefined criteria and ranked (Table 4). During preparation of this manuscript, we discovered that a publication describing the effect of N-acetyl cysteine in MND had been included in error, as no data were available for N-acetyl cysteine monotherapy.[28]

For peer review only

Table 4: Longlisted interventions ranked by drug score from clinical review

Intervention	Number of Publications	Quality Score	Efficacy Score	Safety Score	Study Size Score	Drug score
Rivastigmine	29	3.34	3.33	2.1	2.62	90.62
Memantine	51	3.02	2.87	2.2	2.47	80.7
Vitamin D3	11	3.27	3.01	3.36	2.18	78.08
Donepezil	41	3.1	2.72	2.51	2.24	76.99
Pramipexole	14	3.07	3.04	2.43	2.64	70.41
Galantamine	12	2.75	2.85	2.67	2.5	58.29
Amantadine	59	2.37	3	2.36	1.9	56.53
Dextromethorphan /quinidine	3	4	3.33	2.33	3	56.19
Selegiline	21	3	2.59	2.1	2.38	51.98
4-Aminopyridine	10	3.2	2.76	2.5	2.1	48.28
Acetyl-L-Carnitine	10	2.9	2.64	2.5	2.3	45.83
Simvastatin	5	3.6	2.27	2.8	2.4	42.73
Lamotrigine	6	4	2.25	2.33	2.33	41.41
Bromocriptine	13	2.92	2.4	2.54	1.92	39.21
Clozapine	6	3	2.9	2.5	2	36.8
Gabapentin	9	2.67	2.4	2.44	2.33	36.46
Creatine	12	2.67	2.14	2.33	2.42	35.87
Ginkgo biloba	3	3.67	2.61	3.67	1.67	35.21
Minocycline	11	2.45	2.27	2.64	2.18	34.54
Vitamin E	9	3.33	2.1	2	2.44	34.25
Levetiracetam	6	3	3.33	2.17	1.83	33.57
Atomoxetine	4	3.25	2.5	3.25	1.75	32.3
Coenzyme Q10	9	3.33	2.16	1.89	2.33	31.67
Tacrine	10	3.3	2.81	1.8	1.8	31.26
Olanzapine	5	2.4	3.47	2.6	1.6	26.93
Estrogen	9	3	2.66	1.67	2	26.57
Nimodipine	5	3	2.35	2.2	2.2	26.55
Riluzole	17	2.41	2.35	1.76	2.06	25.88
Ciclosporin	9	2.78	1.93	2.11	2.22	25.15
Dextromethorphan	7	2.29	2.4	2.71	1.86	24.97
Naltrexone	8	2.5	2.22	2.62	1.75	24.29
Theophylline	2	4	3.25	2.5	1.5	23.26
Valproate	9	2.56	2.33	2	1.89	22.53
Fluoxetine	6	2.67	2.42	2.33	1.67	21.18
Levamisole	3	2.67	2.78	2.33	2	20.81
Melatonin	8	2	2.11	2.75	1.88	20.81
Celecoxib	2	4	2	1.5	3	17.18
3,4-Diaminopyridine	6	2.83	2.4	2.17	1.33	16.62
Milacemide	4	3	1.75	2.25	2	16.51

N-acetyl cystine	1	3	3	3	2	16.26
Tranylcypromine	2	2.5	2.5	3.5	1.5	15.66
Aspirin	4	2.75	2	1.75	2.25	15.14
Ursodeoxycholic acid	1	4	2	3	2	14.45
Tolbutamide	2	3	2.5	2	2	14.31
Imipramine	2	3.5	2	2	2	13.36
Lithium	12	2.42	2.19	1.5	1.5	13.29
Modafinil	2	4	3.33	1	2	12.72
Omega 3 Fatty Acid	2	2.5	1.75	3	2	12.52
Octacosanol	2	2.5	2	3.5	1.5	12.52
Indinavir	2	3.5	1.8	1.5	2.5	11.27
Sodium phenylbutyrate	1	4	2	2	2	9.63
Tilorone	1	4	2	2	2	9.63
lipoic acid	2	2.5	2	2	2	9.54
Isoprinosine	4	3	1.75	1.25	2	9.17
Tetrahydrocannab inol	2	3.5	1.5	1.5	2	7.51
Topiramate	1	4	1	2	3	7.22
Haloperidol	2	3	2.33	1	1.5	5.01
Amino acid mixture	5	1.6	2	1	2	4.98
Rolipram	2	1.5	2	3	1	4.29
Alsamin	1	2	3.5	1	2	4.21
Pentoxifylline	3	2	1.61	1	2	3.88
Verapamil	1	2	2	1	2	2.41
IGF-1	1	2	1	1	3	1.81
Propranolol	1	1	3	1	2	1.81
Fluvoxamine	2	1	3	1	1	1.43
Amitriptyline	1	1	1	3	1	0.9

Preclinical systematic review

We identified 14195 publications. After removing duplicates, two independent researchers screened Title and Abstract of 7586 unique publications, with differences reconciled by a third reviewer. 396 studies were included. 330 studies reported survival outcomes. 313 studies reported behavioural outcomes. Of the 66 longlisted interventions from the clinical review, there were preclinical survival data for 20 drugs (Table 5) and behavioural outcome data for 12 drugs.[29] Further data are available in Supplementary File 2.

Table 5: Summary of preclinical studies evaluating the effect of interventions longlisted from the clinical review on survival outcomes. All listed studies used mouse models. LogMSR = log(median survival in treatment group/median survival in control group)

Publication	Drug	Total number of animals	Median survival in treatment group	Median survival in control group	logMSR
Kira 2006	Acetyl-L-Carnitine	20	270	240	0.1178
Barneoud 1999	Aspirin	38	150	155	-0.0328
Tanaka 2011	Bromocriptine	69	40	35	0.1335
Drachman 2002	Celecoxib	55	139	119	0.1554
Karlsson 2004	Ciclosporin	13	144	130	0.1023
Keep 2001	Ciclosporin	11	24	12	0.6931
Turner 2003	Clozapine	16	140	132	0.0588
Andreassen 2001	Creatine	24	155	135	0.1382
Kaddurah-Daouk 2000	Creatine	13	169	144	0.1601
Klivenyi 2004	Creatine	22	150	125	0.1823
Choi 2008	Estrogen	70	135	127	0.0611
Koschnitzky 2014	Fluoxetine	34	139	132	0.0517
Gurney 1996	Gabapentin	17	140	139	0.0072
Gurney 1996	Gabapentin	38	175	165	0.0588
Ferrante 2001	Ginkgo Biloba	20	136	125	0.0843
Fornai 2008	Lithium	20	146	117	0.2214
Gill 2009	Lithium	55	124	127	-0.0239
Pizzasegola 2009	Lithium	20	119	129	-0.0807
Dardiotis 2013	Melatonin	28	143	143	0.0000
Weishaupt 2006	Melatonin	50	137	131	0.0448
Zhang 2013	Melatonin	30	145	137	0.0568
Wang 2005	Memantine	21	130	122	0.0635
Keller 2011	Minocycline	32	147	138	0.0632
Kriz 2002	Minocycline	29	364	336	0.0800
Van Den Bosch 2002	Minocycline	14	155	130	0.1759
Zhang 2003	Minocycline	20	140	130	0.0741
Zhu 2002	Minocycline	20	135	127	0.0611
Andreassen 2000	N-acetyl cysteine	30	134	129	0.0380
Jaarsma 1998	N-acetyl cysteine	28	251	239	0.0490
Yip 2013	Omega 3	32	182	182	0.0000
Petri 2006	Sodium Phenylbutyrate	26	139	127	0.0903
Ryu 2005	Sodium Phenylbutyrate	40	145	127	0.1325
Crochemore 2009	Valproate	11	140	140	0.0000
Rouaux 2007	Valproate	36	115	110	0.0445
Sugai 2004	Valproate	17	295	265	0.1072

For peer review only

Gianfocaro 2013	Vitamin D	100	126	124	0.0160

Shortlisted candidate drugs for clinical trial

21 drugs with supportive evidence in both clinical and preclinical systematic reviews were shortlisted for further evaluation. Simvastatin was added to the shortlist based on data from the clinical review and emerging data on its potential role in pathways of interest. Nuclear factor erythroid 2- related factor 2 (NRF2) is a transcription factor which controls expression and regulation of antioxidant proteins.[30] Modulating NRF2 may therefore protect against oxidative stress, a common feature across neurodegenerative diseases including MND.[30] A separate systematic review of interventions modulating NRF2 pathway in animal *in vivo* models of neurodegeneration and neuronal injury highlighted statins as a candidate drug targeting NRF2.[29 ,31] A clinical trial in Alzheimer's disease demonstrated reduction of cerebrospinal fluid cholesterol level by simvastatin, thus demonstrating blood brain barrier penetrance, while providing supportive evidence that simvastatin may play a role in altering lipid biosynthesis, which may in turn inhibit protein misfolding and stress response mechanisms in MND.[32]

An evidence summary was compiled for each of the 22 shortlisted drugs including the following information: (1) If they had been tested in 3 or more *in vivo* MND studies, (2) the number of clinical trials in people with MND, (3) the putative target pathway, (4) feasibility for delivery via enteral tube (noting that swallowing is commonly affected in MND), (5) detailed safety information including common side effects, rare but serious side effects and requirements for monitoring, (6) published clinical studies in MND, and (7) clinical trials registered on clinicaltrials.gov. The expert panel met on 9th January 2017 and discussed the evidence for each drug. 11 drugs were excluded in the first round. Following a second round of discussions, four other drugs were excluded based on aggregate judgement of data presented. Reasons for exclusion are detailed in Table 6.

Table 6: Drugs excluded following expert panel review and reasons for exclusion.

	**Drug**	**Reason for exclusion**
Excluded after Round 1	Bromocriptine	Unfavourable safety profile
	Gabapentin	>3 previous clinical trials in MND
	Creatine	>3 previous clinical trials in MND
	Clozapine	Unfavourable safety profile
	Minocycline	>3 previous clinical trials in MND
	Valproate	>3 previous clinical trials in MND
	Celecoxib	Unfavourable safety profile

For peer review only

	Aspirin	Poor biological plausibility
	Ginkgo biloba	Poor biological plausibility
	Lithium	>3 previous clinical trials in MND
	Amino acid mixture	>3 previous clinical trials in MND
Excluded after Round 2	Estrogen	Aggregate judgment of data presented
	Vitamin D3	
	Omega 3	
	Sodium phenylbutyrate	

The seven candidate drugs remaining were memantine, acetyl-l-carnitine, simvastatin, ciclosporin, melatonin, fluoxetine and N-acetyl cysteine. The clinical review data for each final shortlisted drug are summarised in Table 7. MND clinical trials for shortlisted drugs including additional trials identified on handsearching are summarised in Table 8.

On 30th January 2017, members of the expert panel independently ranked shortlisted drugs. On 2nd February 2017, the panel reached a consensus to take the two top ranked drugs acetyl-l-carnitine and memantine forward to clinical trial. However, there were subsequent concerns regarding the availability of acetyl-l-carnitine without prescription and the resulting potential that self-medication with a known trial drug by trial participants, in addition to their randomised treatment allocation, might affect trial integrity.

Subsequently, the panel considered the other final shortlisted drugs and also considered emerging and compelling *in vivo* and *in vitro* evidence of the prevention of neurodegeneration by trazodone, through the targeting of eIF2a-P-mediated translational repression.[33] Following detailed consideration, the panel recommended memantine and trazodone as the first two investigational medicinal products for MND-SMART.

For peer review only

Table 7: Clinical systematic review data for the final shortlisted drugs: number of publications (including interventional and observational studies) and participants according to type of disease. AD: Alzheimer's disease; HD: Huntington's disease; MND: Motor neuron disease; MS: Multiple sclerosis; PD: Parkinson's disease.

**Drug**	**Number of publications**						**Number of participants**					
	**MND**	**AD**	**HD**	**MS**	**PD**	**Total**	**MND**	**AD**	**HD**	**MS**	**PD**	**Total**
**Acetyl-L-carnitine**	0	9	1	0	0	**10**	0	1224	10	0	0	**1234**
**Ciclosporin**	2	0	0	7	0	**9**	110	0	0	1092	0	**1202**
**Fluoxetine**	0	0	1	2	3	**6**	0	0	30	51	32	**113**
**Melatonin**	1	4	0	0	3	**8**	3	273	0	0	64	**340**
**Memantine**	1	32	2	1	15	**51**	63	11912	39	116	809	**12939**
**N-acetyl cysteine**	0	1	0	0	0	**1**	0	47	0	0	0	**47**
**Simvastatin**	0	3	0	1	1	**5**	0	469	0	307	12	**788**

For peer review only

Table 8: Summary of previous MND clinical trials for final shortlisted drugs. RCT: Randomised controlled trial; N: number of participants. AE: Adverse events; AR: Adverse reactions; ALC: Acetyl-l-carnitine; ALSFRS: Amyotrophic Lateral Sclerosis Functional Rating Scale; MMT: Manual Muscle Testing; MN: Motor neuron; MRSI: Magnetic Resonance Spectroscopy Imaging; MUNE: Motor unit number estimate; SD: Standard deviation; SUSAR: Suspected Unexpected Severe Adverse Reaction.

**Drug**	**Publication**	**RCT**	**Number of participants (active arm: placebo arm)**	**Duration**	**Primary Outcome Measure**	**Efficacy results**	**Safety results**
**Memantine**	*De Carvalho 2010*	Y	63 (32:31)	12 months	ALSFRS and safety	Equivocal (underpowered)	No increase in AEs
	Levine 2010 (with riluzole)	N	20 (20:0)	18 months	Safety and tolerability	ALSFRS decline of -0.73 points/month (pre-treatment rate -1.07/month)	AE: Nausea in 1 participant.
	Chan 2011	N	24 (24:0)	5 months	MUNE, MRSI, ALSFRS-R and MMT	MUNE: Significant slowing of MN loss (-12.4 ± 3.7/month in run-in phase to -5.3 ± 2.2/month in treatment phase; mean ± SD, p=0.03). Other outcomes equivocal.	AE: similar between run-in and treatment phases
**Acetyl-l-carnitine (ALC)**	*Beghi 2013 (with riluzole)*	Y	82 (42:40)	12 months	Proportion of patients no longer self-sufficient	Significantly less treated participants loss self-sufficiency (80.9% ALC vs 97.5% placebo, p=0.0296)	No significant difference in AEs.
**Simvastatin**	Nil						
**Ciclosporin**	*Appel 1988*	Y	74 (36:38)	48 weeks	Appel ALS rating scale	Progression to 22 points equivocal in treated and untreated patients (relative risk of ciclosporin 0.991, p=0.485)<br><br>In subgroup of male patients with symptoms ≤18 months, relative risk of progression was 0.302, p=0.0205.	Significant number of expected Ars: hirsutism, headache, flushing, nausea and vomiting, tremor, anorexia and gum hyperplasia. No SUSARs.
**Melatonin**	*Weishaupt 2006 (rectal melatonin)*	N	31 (31:0)	12 months	Safety	ALSFRS presented without any quantitative analysis	No AEs reported or observed.
**Fluoxetine**	Nil						
**N-acetyl cysteine**	*Louwerse 1995 (acetylcysteine)*	Y	110 (54:56)	12 months	Survival	Non-significant trend towards improvement in survival (hazard ratio 0.74 in acetylcysteine	No safety data reported

						group compared to placebo, 95% confidence interval, 0.41 to 1.33; log rank test, P=0.31)	

DISCUSSION

Since drugs have undergone rigorous safety and pharmacokinetic testing, drug repurposing — the use of an established drug in a novel therapeutic indication — reduces costs and barriers to clinical development. Our experience of the successful application of a systematic approach to selecting neuroprotective drugs for repurposing in MS clinical trials[17] encouraged us to use a similar approach in MND. The first part of the review assessed clinical data in MND and in other neurodegenerative diseases with potential shared pathophysiological pathways. This allowed for the identification of drugs with good central nervous system penetrance and the potential for efficacy and safety in people with neurodegenerative diseases. However, drug selection based on clinical data alone is biased towards those tested in conditions where large well-designed randomised controlled trials have been performed and where the mechanism of action may be particular to that condition. Notably, two of our top five ranked drugs were cholinesterase inhibitors licensed for Alzheimer's disease, a mechanism less relevant to MND. It was therefore important that we augment this approach with expert opinion and with preclinical data in MND and frontotemporal dementia models to provide mechanistic relevance. Taken together we have compiled evidence from clinical and preclinical data and used this to inform the selection of potential oral neuroprotective agents for clinical evaluation in people with MND. Through sequential systematic review, we identified a short list of 22 candidate interventions selected from an initial set of 595 drugs.

While some identified drugs demonstrate a good safety profile and have a relevant putative target pathway in MND, others have less favourable side effects profiles or a requirement for close therapeutic monitoring (e.g. clozapine) which necessitates a higher threshold of evidence before testing in clinical trial. This highlights another advantage of our approach, in that it allows the identification of interventions that warrant further rigorous preclinical testing ("cislation"[34]) in *in vivo* or *in vitro* models of ALS, with a view to providing more robust information for efficacy to support their inclusion in future clinical trials.

Following rounds of discussion, the expert panel identified memantine as a drug to be tested in MND-SMART. Memantine is a non-competitive N-methyl-D-aspartate (NMDA) receptor antagonist used in the treatment of moderate to severe Alzheimer's disease. It was shown to significantly delay disease progression and improve survival in mouse models carrying a high copy number of $SOD1^{G93A}$. [35] Memantine has been previously tested in three MND clinical trials. A phase II double-blind placebo-controlled study of 63 patients powered to evaluate safety and tolerability did not identify any increase in adverse events.[36] There was a trend towards improvement in patients treated with memantine 20 mg/day, but no significant difference in ALSFRS-R. In a 5-month randomised double-blind study of 24

For peer review only

participants with ALS, there was significant slowing of spinal motor neuron loss as demonstrated on motor unit estimation testing in the high dose group (10 mg twice daily) compared to low dose (5 mg twice daily).[37] Adverse events were not reported. In a single-arm pilot study of 19 ALS patients, patients treated with riluzole and memantine had reduction in rate of ALSFRS decline and reduced CSF tau levels without any increase in adverse events.[38]

We also asked the expert panel to consider other drugs for which relevant data had only become available after the searches described here had been performed. Trazodone was nominated for consideration through this route. Trazodone is an atypical serotonin antagonist and reuptake inhibitor antidepressant. An unbiased drug screen found that trazodone inhibited Protein Kinase RNA-like endoplasmic reticulum kinase (PERK), which is pivotal to stress granule formation, a common feature of neurodegenerative diseases.[33] Inhibition of PERK was found to be beneficial in a fly model of ALS as well as in an *in vitro* neuronal assay of TDP-43 injury.[39] Furthermore, trazodone has been shown to modulate the ER-stress response resulting in an improvement in survival in animal models of prion disease and FTD.[33] Trazodone also modulated mitochondrial energy metabolism and fatty acid synthesis in animal models of Huntington's disease, and may prevent mitochondrial dysfunction in MND.[40] In a randomized double-blind placebo-controlled crossover phase II trial in 31 patients with FTD, trazodone was found to improve cognition as assessed by the neuropsychiatric inventory.[41] In trials of trazodone in Parkinson's and Alzheimer's disease, although there was no improvement in cognition, symptoms of sleep disturbance and depression were alleviated and adverse events were not increased.[42 ,43]

Limitations of this approach

The main challenge in this approach to drug selection is the ambition to base drug choice on the most contemporary evidence. Systematic reviews are time consuming, as evidenced by the interval between our updated search (2013) and expert committee consideration (2017). Furthermore, drugs with promising data in some domains would be excluded if they have been tested in only one disease other than MND; or if they have not been tested clinically despite overwhelming preclinical evidence. We excluded combination therapies, but it may be – as in the treatment of various cancers[44] and infections[45 ,46] – that engagement with multiple targets is required to achieve a substantial disease-modifying effect.

The inclusion of trazodone may be seen as a weakness of this approach, but in our view this demonstrates a strength in the flexibility of our approach. We do not believe that systematic review should be used as part of a rigid selection process with little need for input from experts; but rather that that expert input is informed by a detailed and robust systematic review process. The expert committee selected trazodone in the full knowledge that it had not been selected through the systematic review process, but were convinced that the emerging evidence of potential efficacy, coupled with long standing clinical experience in its use, made it an attractive candidate for testing in MND-SMART.

For peer review only

Finally, some have suggested that the literature-based systematic review approach to drug selection is intrinsically flawed because it does not take into account disease specific pathophysiology (which may be largely unknown).[47] While the three drugs tested in MS-SMART were not effective,[18] we note that two other drugs on the final MS-SMART shortlist - ibudilast[20] and lipoic acid[21] - have since shown promise in independent phase II trials. Lipoic acid has been identified again as a favourable candidate drug in a further, independent review in 2020.[48] We sought to address this issue here by considering, in addition to clinical information, data from in vivo and in vitro research. Although much successful drug repurposing has been opportunistic and serendipitous, we recognise that future efforts should include consideration of our mechanistic understanding of neurodegenerative diseases and should systemically incorporate additional target and pathway-based information.[11]

Future approaches to drug selection in MND-SMART

Ongoing rounds of drug selection for MND-SMART exploit innovations in automating literature searches, screening, and annotation, with these algorithms trained using the human efforts in the work reported here. These techniques show substantial improvements in efficiency in other fields.[49] Using the Systematic Review Facility (SyRF) (https://syrf.org.uk)[50] we have enabled a 'living' systematic review with automatic search, citation screening, identification of disease and drug, and selection of drugs meeting our criteria for the range of diseases in which studies have been performed. Because of similarities between MND and frontotemporal dementia we have included this as an additional disease of interest. Further details are extracted from full text publications of shortlisted drugs by a combination of machine and human work enabled through the SyRF platform, with human monitoring of machine decisions. The incorporation of machine learning and text mining techniques substantially reduces the human effort required and makes this approach feasible in the context of timely drug selection for adaptive clinical trials.

Complementing our literature-based approach, our current platform incorporates data from additional domains, including in house *in vitro* high throughput screening using human induced pluripotent stem cell culture; pathway and network analysis; and mining of drug and trial databases. We have also sought a broader range of inputs to our expert committee such that it now includes those with experience and expertise in managing people with MND and their symptoms, and of clinical trials, translational and clinical neurology, systematic reviews, experimental drug screening, pharmacology, chemistry, and drug discovery.

CONCLUSIONS

We describe our experience in conducting a systematic, structured, unbiased and evidence-based approach to the selection of candidate drugs for evaluation in a clinical trial in MND by combining review of clinical and preclinical literature, and expert panel input. The first two drugs selected are

For peer review only

memantine and trazodone. For future selection, we will incorporate machine learning and text mining to our systematic reviews and data from our drug discovery platform.

FUNDING

For the purpose of open access, the authors have applied a Creative Commons Attribution (CC BY) licence to any Author Accepted Manuscript version arising from this submission. MND-SMART is funded by grants from MND Scotland, My Name'5 Doddie Foundation (DOD/14/15) and specific donations to the Euan MacDonald Centre. The Chandran lab is supported by the UK Dementia Research Institute, which receives its funding from UK DRI Ltd, funded by the UK Medical Research Council, Alzheimer's Society and Alzheimer's Research UK. E.E is a clinical academic fellow jointly funded by MND Scotland (MNDS) and the Chief Scientist Office (CSO) (217ARF R45951). A.R.M. was a Lady Edith Wolfson Clinical Fellow, jointly funded by the Medical Research Council (MRC) and the Motor Neurone Disease Association (MR/R001162/1). A.Salzinger is funded by Marie Sklodowska-Curie actions Innovative Training Network (ITN). B.T.S is funded by Rowling fellowship.

CONFLICTS OF INTEREST

In the last 3 years, J. Chataway has received support from the Efficacy and Evaluation (EME) Programme, a Medical Research Council (MRC) and National Institute for Health Research (NIHR) partnership and the Health Technology Assessment (HTA) Programme (NIHR), the UK MS Society, the US National MS Society and the Rosetrees Trust. He is supported in part by the NIHR University College London Hospitals (UCLH) Biomedical Research Centre, London, UK. He has been a local principal investigator for a trial in MS funded by the Canadian MS society. A local principal investigator for commercial trials funded by: Actelion, Novartis and Roche; and has taken part in advisory boards/consultancy for Azadyne, Janssen, Merck, NervGen, Novartis and Roche.

CONTRIBUTIONS

Suvankar Pal, Siddharthan Chandran, and Malcolm R. Macleod managed the project. Charis Wong, Jenna M. Gregory, Jing Liao, Kieren Egan, Hanna M. Vesterinen, and Malcolm R. Macleod were project administrators. Jenna M. Gregory, Kieren Egan, Hanna M. Vesterinen, Siddharthan Chandran, and Malcolm R. Macleod conceptualised the project. Charis Wong, Jenna M. Gregory, Jing Liao, Kieren Egan, Hanna M. Vesterinen, Aimal Ahmad Khan, Mohammed Maarij Anwar, Caitlin Beagan, Fraser Brown, John Cafferkey, Alessandra Cardinali, Jane Yi Chiam, Claire Chiang, Victoria Collins, Joyce Dormido, Elizabeth Elliott, Peter Foley, Yu Cheng Foo, Lily Fulton-Humble, Angus B. Gane, Stella A. Glasmacher, Áine Heffernan, Kiran Jayaprakash, Nimesh Jayasuriya, Amina Kaddouri, Jamie Kiernan, Gavin Langlands, Danielle Leighton, Jiaming Liu, James Lyon, Arpan R. Mehta, Alyssa Meng, Vivienne Nguyen, Na Hyun Park, Suzanne Quigley, Yousuf Rashid, Andrea Salzinger, Bethany Shiell, Ankur Singh, Tim Soane, Alexandra Thompson, Olaf Tomala, Fergal M. Waldron, Suvankar Pal, and Malcolm R. Macleod performed systematic searching, screening, annotation and data extraction. Charis Wong, Jenna M. Gregory, Jing Liao, Kieren Egan, Hanna M. Vesterinen, and Malcolm R. Macleod developed the project methodology, curated and analysed data. Jing Liao

For peer review only

handled software and programming. Charis Wong developed data visualisation. Alessandra Cardinali and Bhuvaneish T. Selvaraj performed in vitro drug screening. Jeremy Chataway, Robert Swingler, Peter Connick, Suvankar Pal, Siddharthan Chandran, and Malcolm R. Macleod were members of the expert panel. Charis Wong and Jenna M. Gregory wrote the original draft. Charis Wong, Jenna M. Gregory, Jing Liao, John Cafferkey, Arpan R. Mehta, Jeremy Chataway, Robert Swingler, Suvankar Pal, Siddharthan Chandran, and Malcolm R. Macleod reviewed and edited the manuscript. All authors have read and approved the manuscript.

DATA AVAILABILITY

All data relevant to the study are included in the article or uploaded as supplementary information.

ETHICS APPROVAL STATEMENT

This study does not involve human participants or animal subjects. Ethical approval was not required.

For peer review only

REFERENCES

1. Westeneng HJ, Debray TPA, Visser AE, et al. Prognosis for patients with amyotrophic lateral sclerosis: development and validation of a personalised prediction model. *Lancet Neurol* 2018;**17**(5):423-33.
2. Wong C, Stavrou M, Elliott E, et al. Clinical trials in amyotrophic lateral sclerosis: a systematic review and perspective. *Brain Commun* 2021;**3**(4):fcab242.
3. European Medicines Agency. Refusal of the marketing authorisation for Alsitek (masitinib). In: Agency EM, ed., 2018.
4. European Medicines Agency. Withdrawal assessment report Radicava (International non-proprietary name: edaravone). Procedure No. EMEA/H/C/004938/0000. In: Use EMACfMPfH, ed. Amsterdam, The Netherlands, 2019.
5. Paganoni S, Hendrix S, Dickson SP, et al. Long-term survival of participants in the CENTAUR trial of sodium phenylbutyrate-taurursodiol in amyotrophic lateral sclerosis. *Muscle Nerve* 2021;**63**(1):31-39.
6. Paganoni S, Macklin EA, Hendrix S, et al. Trial of Sodium Phenylbutyrate-Taurursodiol for Amyotrophic Lateral Sclerosis. *N Engl J Med* 2020;**383**(10):919-30.
7. Miller TM, Cudkowicz ME, Genge A, et al. Trial of Antisense Oligonucleotide Tofersen for SOD1 ALS. *N Engl J Med* 2022;**387**(12):1099-110.
8. Witzel S, Maier A, Steinbach R, et al. Safety and Effectiveness of Long-term Intravenous Administration of Edaravone for Treatment of Patients With Amyotrophic Lateral Sclerosis. *JAMA Neurol* 2022.
9. van den Berg LH, Sorenson E, Gronseth G, et al. Revised Airlie House consensus guidelines for design and implementation of ALS clinical trials. *Neurology* 2019;**92**(14):e1610-e23.
10. Perrin S. Preclinical research: Make mouse studies work. *Nature* 2014;**507**(7493):423-5.
11. Pushpakom S, Iorio F, Eyers PA, et al. Drug repurposing: progress, challenges and recommendations. *Nat Rev Drug Discov* 2018.
12. Giovannoni G. Cladribine to Treat Relapsing Forms of Multiple Sclerosis. *Neurotherapeutics* 2017;**14**(4):874-87.
13. The Lancet. Alemtuzumab for multiple sclerosis. *The Lancet* 2012;**380**(9856):1792.
14. Coles AJ, Twyman CL, Arnold DL, et al. Alemtuzumab for patients with relapsing multiple sclerosis after disease-modifying therapy: a randomised controlled phase 3 trial. *The Lancet* 2012;**380**(9856):1829-39.
15. Salzer J, Svenningsson R, Alping P, et al. Rituximab in multiple sclerosis: A retrospective observational study on safety and efficacy. *Neurology* 2016;**87**(20):2074-81.
16. Chalmers I, Bracken MB, Djulbegovic B, et al. How to increase value and reduce waste when research priorities are set. *Lancet* 2014;**383**(9912):156-65.
17. Vesterinen HM, Connick P, Irvine CM, et al. Drug repurposing: a systematic approach to evaluate candidate oral neuroprotective interventions for secondary progressive multiple sclerosis. *PloS one* 2015;**10**(4):e0117705.
18. Chataway J, De Angelis F, Connick P, et al. Efficacy of three neuroprotective drugs in secondary progressive multiple sclerosis (MS-SMART): a phase 2b, multiarm, double-blind, randomised placebo-controlled trial. *The Lancet Neurology*.
19. Connick P, De Angelis F, Parker RA, et al. Multiple Sclerosis-Secondary Progressive Multi-Arm Randomisation Trial (MS-SMART): a multiarm phase IIb randomised, double-blind, placebo-controlled clinical trial comparing the efficacy of three neuroprotective drugs in secondary progressive multiple sclerosis. *BMJ open* 2018;**8**(8):e021944.
20. Fox RJ, Coffey CS, Conwit R, et al. Phase 2 Trial of Ibudilast in Progressive Multiple Sclerosis. *New England Journal of Medicine* 2018;**379**(9):846-55.
21. Spain R, Powers K, Murchison C, et al. Lipoic acid in secondary progressive MS: A randomized controlled pilot trial. *Neurol Neuroimmunol Neuroinflamm* 2017;**4**(5):e374.
22. Wong C, Dakin RS, Williamson J, et al. Motor Neuron Disease Systematic Multi-Arm Adaptive Randomised Trial (MND-SMART): a multi-arm, multi-stage, adaptive, platform, phase III randomised, double-blind, placebo-controlled trial of repurposed drugs in motor neuron disease. *BMJ open* 2022;**12**(7):e064173.
23. Mehta AR, Chataway J, Pal S, et al. Trials for neurodegenerative diseases: time to innovate. *Lancet Neurol* 2021;**20**(12):984.
24. Mehta AR, Pal S, Chataway J, et al. Smarter adaptive platform clinical trials in neurology: a showcase for UK innovation. *Brain* 2022;**145**(8):e64-e65.
25. Macleod M, Wong C. ReLiSyR MND. Secondary ReLiSyR MND 2019. https://osf.io/UBMHE/.

For peer review only

26. Egan K VH, Macleod MR. A novel strategy to identify candidate drugs for clinical trial in Motor Neuron disease. 2019.
27. Gregory JM, Waldron FM, Soane T, et al. Protocol for a systematic review and meta-analysis of experimental models of amyotrophic lateral sclerosis. *Evidence-based Preclinical Medicine* 2016;**3**(2):e00023.
28. Vyth A, Timmer JG, Bossuyt PM, et al. Survival in patients with amyotrophic lateral sclerosis, treated with an array of antioxidants. *J Neurol Sci* 1996;**139 Suppl**:99-103.
29. Gregory JM. A systematic approach to identify oral neuroprotective interventions for motor neuron disease. University of Edinburgh, 2016.
30. Johnson DA, Johnson JA. Nrf2--a therapeutic target for the treatment of neurodegenerative diseases. *Free Radic Biol Med* 2015;**88**(Pt B):253-67.
31. Iwamoto K, Yoshii Y, Ikeda K. Atorvastatin treatment attenuates motor neuron degeneration in wobbler mice. *Amyotroph Lateral Scler* 2009;**10**(5-6):405-9.
32. Kim HE, Grant AR, Simic MS, et al. Lipid Biosynthesis Coordinates a Mitochondrial-to-Cytosolic Stress Response. *Cell* 2016;**166**(6):1539-52.e16.
33. Halliday M, Radford H, Zents KAM, et al. Repurposed drugs targeting eIF2α-P-mediated translational repression prevent neurodegeneration in mice. *Brain* 2017;**140**(6):1768-83.
34. Macleod M, Mohan S. Reproducibility and Rigor in Animal-Based Research. *ILAR Journal* 2019;**60**(1):17-23.
35. Wang R, Zhang D. Memantine prolongs survival in an amyotrophic lateral sclerosis mouse model. *European Journal of Neuroscience* 2005;**22**(9):2376-80.
36. de Carvalho M, Pinto S, Costa J, et al. A randomized, placebo-controlled trial of memantine for functional disability in amyotrophic lateral sclerosis. *Amyotrophic lateral sclerosis : official publication of the World Federation of Neurology Research Group on Motor Neuron Diseases* 2010;**11**(5):456-60.
37. Chan K, R T, G B, et al. C82 a randomized, double-blind, dose-ranging study of memantine in patients with amyotrophic lateral sclerosis. 22nd International Symposium on ALS/MND. *Amyotrophic Lateral Sclerosis* 2011;**12**(supp1):48.
38. Levine TD, Bowser R, Hank N, et al. A pilot trial of memantine and riluzole in ALS: correlation to CSF biomarkers. *Amyotrophic lateral sclerosis : official publication of the World Federation of Neurology Research Group on Motor Neuron Diseases* 2010;**11**(6):514-9.
39. Kim HJ, Raphael AR, LaDow ES, et al. Therapeutic modulation of eIF2alpha phosphorylation rescues TDP-43 toxicity in amyotrophic lateral sclerosis disease models. *Nat Genet* 2014;**46**(2):152-60.
40. Lauterbach EC. Neuroprotective effects of psychotropic drugs in Huntington's disease. *Int J Mol Sci* 2013;**14**(11):22558-603.
41. Lebert F, Stekke W, Hasenbroekx C, et al. Frontotemporal dementia: a randomised, controlled trial with trazodone. *Dement Geriatr Cogn Disord* 2004;**17**(4):355-9.
42. Werneck AL, Rosso AL, Vincent MB. The use of an antagonist 5-HT2a/c for depression and motor function in Parkinson' disease. *Arq Neuropsiquiatr* 2009;**67**(2B):407-12.
43. Camargos EF, Quintas JL, Louzada LL, et al. Trazodone and cognitive performance in Alzheimer disease. *J Clin Psychopharmacol* 2015;**35**(1):88-9.
44. Bayat Mokhtari R, Homayouni TS, Baluch N, et al. Combination therapy in combating cancer. *Oncotarget* 2017;**8**(23):38022-43.
45. Schmid A, Wolfensberger A, Nemeth J, et al. Monotherapy versus combination therapy for multidrug-resistant Gram-negative infections: Systematic Review and Meta-Analysis. *Scientific Reports* 2019;**9**(1):15290.
46. Arts EJ, Hazuda DJ. HIV-1 antiretroviral drug therapy. *Cold Spring Harb Perspect Med* 2012;**2**(4):a007161-a61.
47. Fox RJ. Feast or famine in multiple sclerosis therapeutics. *The Lancet Neurology*.
48. Cunniffe N, Vuong KA, Ainslie D, et al. Systematic approach to selecting licensed drugs for repurposing in the treatment of progressive multiple sclerosis. *J Neurol Neurosurg Psychiatry* 2021;**92**(3):295-302.
49. Bannach-Brown A, Przybyla P, Thomas J, et al. Machine learning algorithms for systematic review: reducing workload in a preclinical review of animal studies and reducing human screening error. *Systematic reviews* 2019;**8**(1):23.
50. Bahor Z, Liao J, Currie G, et al. Development and uptake of an online systematic review platform: the early years of the CAMARADES Systematic Review Facility (SyRF). *BMJ Open Sci* 2021;**5**(1):e100103.

For peer review only

Figure legends

Figure 1: Diagram illustrating two-stage systematic review approach to inform identification and selection of putative treatments to take forward to clinical trial.

Figure 2: PRISMA diagram for clinical systematic review. Ti/Ab: Title/Abstract

For peer review only

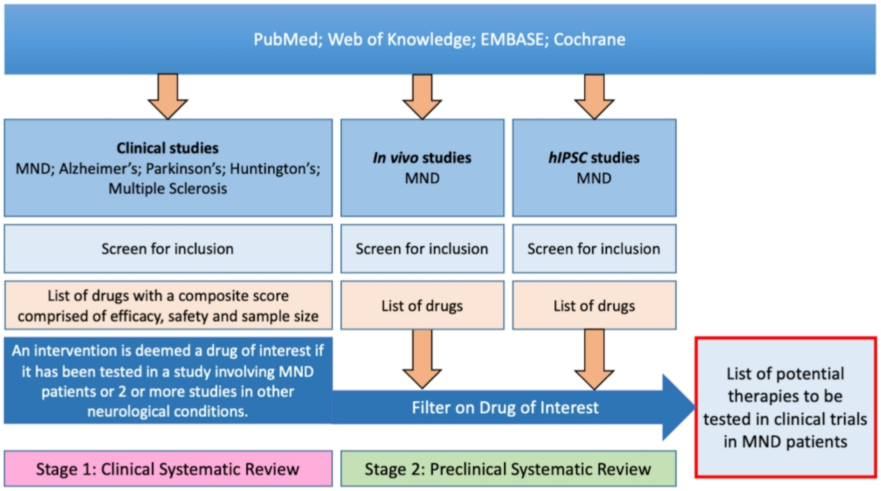

Diagram illustrating two-stage systematic review approach to inform identification and selection of putative treatments to take forward to clinical trial.

337x190mm (300 x 300 DPI)

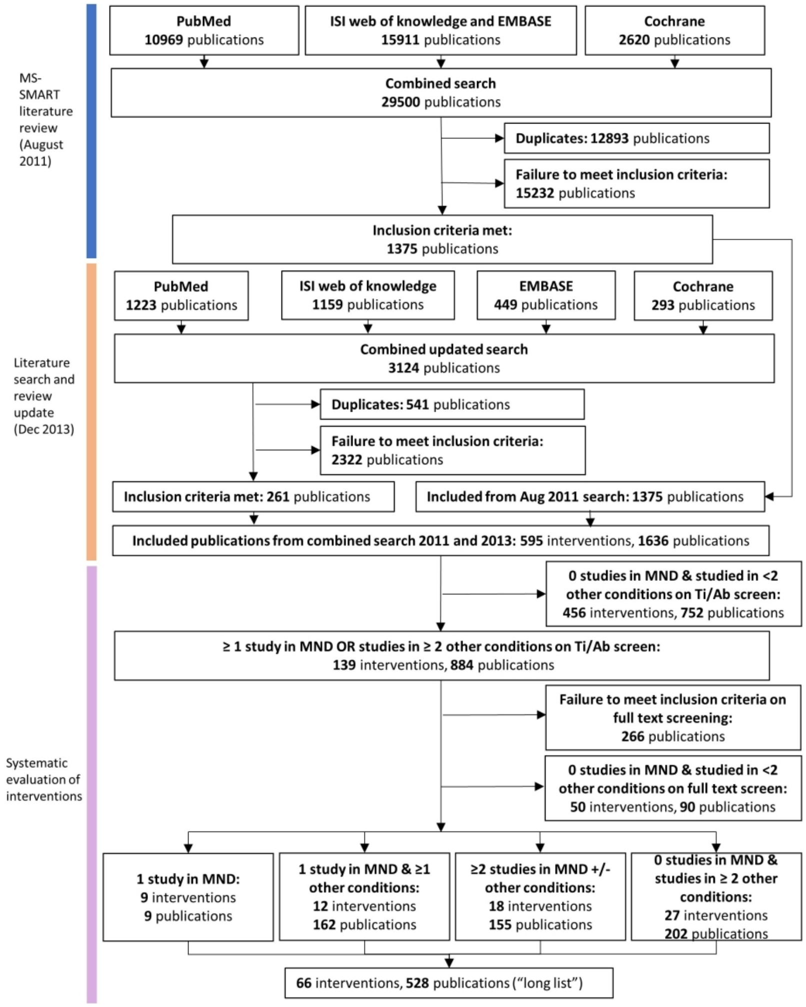

PRISMA diagram for clinical systematic review. Ti/Ab: Title/Abstract

190x275mm (300 x 300 DPI)

Surname	Year	Publication	Pub ID	Drug	Type of Disease
Aarestrup	1989	16946	6290	theophyllin	MND
Aarsland	1998	13086	20586	olanzapine	Parkinsons Disease
Aarsland	2003	12035	1562	galantamin	Parkinsons Disease
Aarsland	2002	12047	1791	donepezil	Parkinsons Disease
Aarsland	2009	11096	261	memantine	Parkinsons Disease
Acierno	1983	12743	7181	acetyl-l-car	Alzheimers Disease
Adair	2001	12088	5778	n-acetyl cys	Alzheimers Disease
Addy	2009	12409	271	mk-0657	Parkinsons Disease
Addy	2009	12410	271	levodopa/c	Parkinsons Disease
Agashivala	2013	16920	1170	fingolimod	Multiple Sclerosis
Aggarwal	2010	11798	7712	lithium	MND
Aggarwal	2010	11960	7712	riluzole	MND
Agnoli	1992	12137	6913	selegiline	Alzheimers Disease
Aguera-Ort	2008	13243	18411	rivastigmin	Alzheimers Disease
Aguera-Ort	2010	12734	3821	memantine	Alzheimers Disease
Ahlin	1991	12881	10975	tetrahydro	Alzheimers Disease
Ahlskog	1991	12436	2982	mk-458 (hp	Parkinsons Disease
Ahmed	1968	13380	3775	chlorpropa	Parkinsons Disease
Ahrens	2001	12466	9784	mmf (myco	Multiple Sclerosis
Aimard	1978	13289	10857	azathioprin	Multiple Sclerosis
Aisen	1996	11884	8138	3,4-diamin	MND
Aisen	2006	12148	4644	3-amino-1-	Alzheimers Disease
Aisen	1996	13259	19755	colchicine	Alzheimers Disease
Aisen	1996	16925	1041	3,4-diamin	MND
Aisen	2000	12091	6046	prednisolor	Alzheimers Disease
Aisen	2001	12094	5823	hydroxychl	Alzheimers Disease
Aisen	2001	12096	5823	hydroxychl	Alzheimers Disease
Aisen	2002	12098	5707	nimesulide	Alzheimers Disease
Aisen	2003	12138	5488	rofecoxib	Alzheimers Disease
Aisen	2003	12139	5488	naproxen	Alzheimers Disease
Aisen	2003	12354	5535	folic acid /	Alzheimers Disease
Aisen	1996	12633	6561	prednisolor	Alzheimers Disease
Aisen	2008	12179	4223	vitamin b a	Alzheimers Disease
Aisen	2011	12850	26657	tramiprosa	Alzheimers Disease
Akhondzad	2003	12293	5475	melissa offi	Alzheimers Disease
Akhondzad	2003	12294	5540	salvia offici	Alzheimers Disease
Akhondzad	2010	12216	3834	saffron	Alzheimers Disease
Akhondzad	2010	12224	4001	saffron	Alzheimers Disease
Akhondzad	2010	12225	4001	crocus sati	Alzheimers Disease
Akuffo	2008	12299	4236	rosiglitazon	Alzheimers Disease
Alarcon	1998	12861	20360	bromocript	Parkinsons Disease
Alarcon	1998	12862	20360	l-dopa/bror	Parkinsons Disease
Albayrak	2012	16820	130	fluvoxamin	Huntingtons Disease
Al-Chalabi	2009	16975	4055	lithium	MND
Al-Chalabi	2011	16911	6337	lithium carl	MND
Alikhani	2009	19018	72	calcitriol	Multiple Sclerosis
Allain	1988	13353	3152	exifone	Parkinsons Disease
Allain	1997	12619	6515	s12024	Alzheimers Disease
Alva	2011	16662	981	rivastigmin	Alzheimers Disease

For peer review only

Alvarez	1999	12213	6048	citicoline	Alzheimers Disease
Alvarez	2000	12583	5872	anopsis	Alzheimers Disease
Alvarez	2011	13157	26641	cerebrolysi	Alzheimers Disease
Alvarez	2011	13158	26641	donepezil	Alzheimers Disease
Alvarez	2011	13159	26641	donepezil/c	Alzheimers Disease
Amaducci	1988	12723	7096	phosphatid	Alzheimers Disease
Amanullah	2011	16933	267	nabilone	Alzheimers Disease
Amato	1993	13135	26846	azathioprin	Multiple Sclerosis
Amenta	2011	13132	26653	donepezil/c	Alzheimers Disease
Aminoff	1974	11085	7677	lithium	Huntingtons Disease
Aminoff	1974	11941	7673	lithium	Huntingtons Disease
Anand	2000	13088	20795	rivastigmin	Alzheimers Disease
Andersen	2012	16663	532	donepezil	Alzheimers Disease
Androsova	2000	12741	5914	memantine	Alzheimers Disease
Androsova	2000	12800	5914	memantine	Alzheimers Disease
Anonymous	1996	16972	2740	riluzole	MND
Anonymous	1999	16958	1324	bdnf	MND
Apostolski	1998	12720	8078	nimodipine	MND
Apostolski	1998	12724	8078	alsamin	MND
Apostolski	1998	12725	8078	se/vitamin	MND
Apostolski	1998	12726	8078	l-arg/l-met/	MND
Appel	1988	11810	8241	ciclosporin	MND
Appleton	1970	13901	3711	amantadine	Parkinsons Disease
Appleton	1971	13895	3658	amantadine	Parkinsons Disease
Aragona	2009	12568	8625	cannabis ex	Multiple Sclerosis
Arai	2009	12355	4121	donepezil/c	Alzheimers Disease
Arnulf	2001	12057	1926	sr27897b	Parkinsons Disease
Arun	2013	16589	23365093	amiloride	Multiple Sclerosis
Asayama	2003	12130	5424	melatonin	Alzheimers Disease
Asher	1981	12585	7628	tetrabenazi	Huntingtons Disease
Ashlskog	1996	13054	19865	levodopa/c	Parkinsons Disease
Ashlskog	1996	13055	19865	levodopa/c	Parkinsons Disease
Askmark	1993	11844	8213	dextrometh	MND
Atri	2008	12348	4297	cholinester	Alzheimers Disease
Atri	2008	12349	4297	memantine	Alzheimers Disease
Auriel	2006	12419	1020	methylphen	Parkinsons Disease
Avila	2003	12036	1564	fluoxetine	Parkinsons Disease
Avila	2003	12037	1564	nefazodone	Parkinsons Disease
babiloni	2009	12197	4118	ibuprofen	Alzheimers Disease
Bachurin	2001	12198	5813	dimebon	Alzheimers Disease
Bakchine	2007	11116	4491	memantine	Alzheimers Disease
Bakchine	2008	12812	4361	memantine	Alzheimers Disease
Bakheit	1990	13072	15363	levodopa/b	Parkinsons Disease
Ballantyne	1989	16893	451	ciclosporin	MND
Bandini	2002	12049	1852	brasofensin	Parkinsons Disease
Bandini	2002	12053	1852	amantadine	Parkinsons Disease
Banerjee	2011	12887	19036	sertraline	Alzheimers Disease
Banerjee	2011	12888	19036	mirtazapine	Alzheimers Disease
Banerjee	2013	16664	151	sertraline	Alzheimers Disease
Banerjee	2013	16665	151	mirtazapine	Alzheimers Disease

For peer review only

Bara-Jimen	2003	12039	1587	istradefyllir	Parkinsons Disease
Bara-Jimen	2006	11987	944	riluzole	Parkinsons Disease
Barak	1996	12178	6582	inositol	Alzheimers Disease
Barak	1999	12472	10064	moclobemi	Multiple Sclerosis
Barak	2001	12847	21145	donepezil	Alzheimers Disease
Barbato	1996	13052	19828	melperone	Parkinsons Disease
Barbeau	1971	12022	3668	amantadine	Parkinsons Disease
Barbeau	1971	13898	3687	levodopa/r	Parkinsons Disease
Barbeau	1982	12516	10817	cyprohepta	Multiple Sclerosis
Bares	2001	13096	21238	pergolide	Parkinsons Disease
Barkhof	2010	12471	8396	ibudilast	Multiple Sclerosis
Barrie	1989	12864	19169	madopar	Parkinsons Disease
Barrie	1989	12866	19169	madopar cr	Parkinsons Disease
Bass	1990	12948	26928	pemoline	Multiple Sclerosis
Bassi	1986	12626	7609	transdihydr	Huntingtons Disease
Bastard	1976	13210	15610	5-hydroxy-l	Parkinsons Disease
Bastone	1995	16941	958	amino acid	MND
Bates	1977	12531	10863	linoleic acic	Multiple Sclerosis
Bates	1978	12528	10848	linoleic acic	Multiple Sclerosis
Bates	1988	13165	26932	fatty acids	Multiple Sclerosis
Bates	1989	12523	10642	maxepa oil	Multiple Sclerosis
Batysheva	2010	13245	18866	tizanidine	Multiple Sclerosis
Bauer	1974	12707	3527	amantadine	Parkinsons Disease
Bauer	2012	16939	7128	tch346	MND
Baum	2007	12296	4450	cucumin	Alzheimers Disease
Baum	2008	12442	4403	curcumin	Alzheimers Disease
Baum	2008	12777	4403	curcumin	Alzheimers Disease
Becker	1974	13166	26956	4 ammoniu	Multiple Sclerosis
Bedard	1977	12018	3423	nomifensin	Parkinsons Disease
Bedard	1998	12426	2372	naphthoxaz	Parkinsons Disease
Bedard	1999	12081	2190	trihexypher	Parkinsons Disease
Bedlack	2009	11951	7756	levetiraceta	MND
Beglinger	2009	11789	7244	atomoxetin	Huntingtons Disease
Beister	2004	11491	7397	memantine	Huntingtons Disease
Belfiore	1998	12868	20385	flunarizine	Huntingtons Disease
Bellelli	2005	12716	5034	cholinester	Alzheimers Disease
Bender	2005	11817	7386	creatine	Huntingtons Disease
Bensimon	1994	11854	8199	riluzole	MND
Bensimon	1994	12935	19451	riluzole	MND
Bensimon	2002	12715	7958	riluzole	MND
Bentham	1990	12123	6998	l-tryptopha	Alzheimers Disease
bentham	2008	12181	4427	aspirin	Alzheimers Disease
Berg	2011	16873	913	afq056	Parkinsons Disease
Bergamasc	1994	12649	6800	idebenone	Alzheimers Disease
Bertorini	2011	13037	19034	3,4-diamino	MND
Bever	1994	12664	10479	4-aminopyr	Multiple Sclerosis
Bever	1996	12486	10299	3,4-diamino	Multiple Sclerosis
Bianchetti	2003	12578	5460	acetyl-l-car	Alzheimers Disease
Bidzan	2005	12737	4936	ginkgo bilol	Alzheimers Disease
Bielekova	2009	12476	8489	rolipram	Multiple Sclerosis

For peer review only

Bierer	1993	12150	6868	physostigm	Alzheimers Disease
Bierer	1994	12644	6781	physostigm	Alzheimers Disease
Bierer	1994	12645	6781	physostigm	Alzheimers Disease
Bilikiewicz	2004	12114	5294	colostrinin	Alzheimers Disease
Birkmayer	1996	12643	6628	coenzyme r	Alzheimers Disease
Bisaga	2011	16952	1152	cytoflavin	Multiple Sclerosis
Bissay	1994	12509	10456	bromocript	Multiple Sclerosis
Bittencourt	1988	12811	10645	oxcarbazep	Multiple Sclerosis
Black	2010	11508	2	syn115	Parkinsons Disease
Black	2010	12398	3882	bapineuzur	Alzheimers Disease
Blackwell	2008	11794	7288	modafinil	Huntingtons Disease
Blass	1988	12106	7087	thiamine	Alzheimers Disease
Blesa	2006	12898	11314	galantamin	Alzheimers Disease
Blin	1989	12745	8236	l-threonine	MND
Blin	1991	12744	8227	l-threonine	MND
Blin	1992	11966	8219	l-threonine	MND
Blin	1996	11972	8144	dextrometh	MND
Blonsky	1974	12668	3557	elantrine	Parkinsons Disease
Blonsky	1974	12671	3557	trihexypher	Parkinsons Disease
Blonsky	1976	13373	3461	elantrine	Parkinsons Disease
Bohuslavizk	1993	13058	27004	ruta gravec	Multiple Sclerosis
Boiko	2007	13167	13655	levodopa/c	Parkinsons Disease
Boman	1970	12694	3755	amantadine	Parkinsons Disease
Bonelli	2002	11833	7447	olanzapine	Huntingtons Disease
Bonelli	2002	11837	7460	olanzapine	Huntingtons Disease
Bonelli	2003	11829	7437	minocycline	Huntingtons Disease
Bonelli	2004	11818	7394	minocycline	Huntingtons Disease
Bonnefont-	2000	12270	8015	riluzole	MND
Bonuccelli	1992	12433	2923	dextrometh	Parkinsons Disease
Bonuccelli	2004	13118	22521	rivastigmine	Parkinsons Disease
Bonuccelli	2012	16823	280	pridopidine	Parkinsons Disease
Boomsma	1995	12893	19620	madopar	Parkinsons Disease
Boomsma	1995	12894	19620	sinemet	Parkinsons Disease
Borghamm	2008	11589	617	memantine	Parkinsons Disease
Bossuyt	1995	12540	8173	n-acetyl cys	MND
Boston	2004	12104	5172	ethyl-epa	Alzheimers Disease
Bottiglieri	1990	12975	12882	s-adenosylr	Alzheimers Disease
Bottini	1992	13138	6901	oxiracetam	Alzheimers Disease
Bowen	2003	12507	9560	pirfenidone	Multiple Sclerosis
Bowes	1991	12379	2990	levodopa/c	Parkinsons Disease
Bozek	1984	13059	27030	isoniazid	Multiple Sclerosis
Bozek	1984	13078	27604	isoniazid	Multiple Sclerosis
Brannan	1995	12842	15156	levodopa/c	Parkinsons Disease
Brannan	1995	12843	15156	selegine \ l	Parkinsons Disease
Braun	1989	12449	11212	skf 38393	Huntingtons Disease
Breitner	2011	16981	1725	celecoxib	Alzheimers Disease
Breitner	2011	16982	1725	naproxen	Alzheimers Disease
Breuer	2007	11120	8794	lamotrigine	Multiple Sclerosis
Breuer	2007	12493	8794	lamotrigine	Multiple Sclerosis
Brichta	2006	11907	7840	valproate	MND

For peer review only

Brinkman	1982	12069	7192	lecithin	Alzheimers Disease
Brioschi	2009	12872	25792	modafinil	Multiple Sclerosis
Brody	1974	11811	8280	inosiplex	MND
Bromberg	1994	16971	2268	amino acid	MND
Brooks	2001	16976	2190	xaliproden	MND
Brooks	2004	11915	7895	dextrometh	MND
Brooks	1998	12232	6278	acetyl-l-car	Alzheimers Disease
Bruce	2012	16842	1729	armodafinil	Multiple Sclerosis
Brusa	2009	11790	7267	aripiprazole	Huntingtons Disease
Brusa	2009	11791	7267	tetrabenazi	Huntingtons Disease
Brusco	1999	12404	6183	melatonin	Alzheimers Disease
Bukatina	1993	12154	6862	amiridin	Alzheimers Disease
Burn	2006	11758	879	rivastigmin	Parkinsons Disease
Burns	2011	13924	26655	donepezil	Alzheimers Disease
Burton	1985	12994	15516	mesulergin	Parkinsons Disease
Burton	2010	12464	8374	vitamin d3	Multiple Sclerosis
Burton	1985	12992	15516	mesulergin	Parkinsons Disease
Butzer	1975	12713	3490	amantadine	Parkinsons Disease
Cacabelos	1996	12844	19792	citicoline	Alzheimers Disease
Caine	1979	12374	7637	clozapine	Huntingtons Disease
Cakir	2008	13127	24614	mirtazapine	Alzheimers Disease
calabrese	2007	11113	4498	memantine	Alzheimers Disease
Callaghan	1974	13337	3541	levodopa/a	Parkinsons Disease
Callaghan	1975	12662	3479	piribedil	Parkinsons Disease
Callaghan	1975	12663	3479	piribedil / a	Parkinsons Disease
Callaghan	1974	13336	3541	amantadine	Parkinsons Disease
Campanella	1972	13391	3610	amantadine	Parkinsons Disease
Campbell	1972	13900	3644	amantadine	Parkinsons Disease
Campi	1990	12673	7004	selegiline	Alzheimers Disease
Campi	1990	12674	7004	l-acetylcarr	Alzheimers Disease
Candalino	1977	13392	7654	lithium carl	Huntingtons Disease
Candelise	1973	12608	7679	amantadine	Huntingtons Disease
Candelise	1973	12609	7679	diazepam/l	Huntingtons Disease
Candelise	1973	12610	7679	diazepam/r	Huntingtons Disease
Candelise	1973	12611	7679	l-dopa/ro 4	Huntingtons Disease
Cantillon	1996	12838	19779	haloperidol	Alzheimers Disease
Cantillon	1996	12839	19779	buspirone	Alzheimers Disease
Caparros-Le	2005	13121	23160	cyproteron	Alzheimers Disease
Caraceni	1978	11891	7638	deanol	Huntingtons Disease
Caraceni	1980	12289	7633	bromocript	Huntingtons Disease
Caraceni	2001	13097	21265	selegiline	Parkinsons Disease
Caraceni	2001	13099	21265	bromocript	Parkinsons Disease
Caramia	2000	11902	8039	diazepam	MND
Caramia	2000	11904	8039	gabapentin	MND
Caramia	2000	11905	8039	riluzole	MND
Carman	1974	11919	7674	lithium	Huntingtons Disease
Caro	1978	11934	7651	vitamin e	Huntingtons Disease
Carolei	1976	13390	3441	melanocyte	Parkinsons Disease
Carroll	2004	12026	1344	cannador	Parkinsons Disease
Carter	2005	11954	7882	modafinil	MND

For peer review only

Carter	1993	13060	27100	3,4-diamino	Multiple Sclerosis
Casacchia	1983	12450	3291	rolipram	Parkinsons Disease
Castrop	2012	16892	2870	fingolimod	Multiple Sclerosis
Cendrowsk	1971	13369	10929	azathioprin	Multiple Sclerosis
Chacko	1995	13048	19616	clozapine	Parkinsons Disease
Chan	2009	12441	4193	vitamin/nu	Alzheimers Disease
Chan	2013	16852	1758	amantadine	Parkinsons Disease
Chana	1997	12331	2461	gabapentin	Parkinsons Disease
Chandra	2001	12545	5792	nutritional	Alzheimers Disease
Chang	2008	12765	426	madopar	Parkinsons Disease
Chappel	2007	12297	4544	ly451395	Alzheimers Disease
Chen	2010	13130	25914	polygonum	Alzheimers Disease
Chen	2010	11947	7692	hydroxyure	MND
Chen	2007	12766	634	madopar	Parkinsons Disease
Chen	2010	13129	25914	piracetam	Alzheimers Disease
Chio	2010	12326	7702	lithium	MND
Chipilsky	1997	13168	27134	anastal	Multiple Sclerosis
Chiu	2009	12954	25277	rivastigmin	Alzheimers Disease
Chouza	1982	13370	3303	tiapride	Huntingtons Disease
Clarke	2001	12058	1938	remacemid	Parkinsons Disease
Claus	1991	12665	6961	piracetam	Alzheimers Disease
Clemens	2008	11869	7772	morphine	MND
Clemens	1995	12344	2778	nicotine	Parkinsons Disease
Cohen	1989	12519	10620	amantadine	Multiple Sclerosis
Comi	2010	11566	8310	laquinomo	Multiple Sclerosis
Comi	2013	16856	89	cladribine	Multiple Sclerosis
Comi	2012	16671	483	laquinomo	Multiple Sclerosis
Como	1997	11856	7535	fluoxetine	Huntingtons Disease
Confavreux	2012	16672	329	teriflunomi	Multiple Sclerosis
Connelly	2008	12350	4505	cholinester	Alzheimers Disease
Consroe	1991	11864	7584	cannabidiol	Huntingtons Disease
Cook	2011	13061	27202	cladribine	Multiple Sclerosis
Corey-Bloo	1998	13267	20283	rivastigmin	Alzheimers Disease
Cornelli	2010	12391	3917	donepezil/a	Alzheimers Disease
Cornelli	2010	12392	3917	donepezil/a	Alzheimers Disease
Corvol	2011	12841	13072	entacapone	Parkinsons Disease
Cotzias	1976	13887	3450	levodopa/a	Parkinsons Disease
Couto	1976	13889	3460	levodopa/g	Parkinsons Disease
Couto	1976	13890	3460	levodopa/a	Parkinsons Disease
Cree	2010	12457	8325	naltrexone	Multiple Sclerosis
Cretu	2008	12748	4294	donepezil/r	Alzheimers Disease
Cretu	2008	12749	4294	donepezil/r	Alzheimers Disease
Cretu	2008	13148	24581	galantamin	Alzheimers Disease
Croisile	1993	13361	6856	piracetam	Alzheimers Disease
Crook	1992	12651	6921	phosphatid	Alzheimers Disease
Cubo	2006	11801	7337	donepezil	Huntingtons Disease
Cucinotta	1985	13371	7147	naftidrofur	Alzheimers Disease
Cucinotta	1996	12177	6588	dihydroerg	Alzheimers Disease
Cudkowicz	2003	11928	7925	topiramate	MND
Cudkowicz	2006	11903	7829	celecoxib	MND

For peer review only

Cudkowicz	2008	11863	7764	arimoclom	MND
Cudkowicz	2009	11950	7758	sodium phe	MND
Cudkowicz	2011	16860	1822	dexpramip	MND
Cumbo	2005	13284	22867	rivastigmin	Alzheimers Disease
Cumbo	2005	13285	22867	galantamin	Alzheimers Disease
Cumbo	2005	13286	22867	donepezil	Alzheimers Disease
Currier	1993	12511	10513	methotrexa	Multiple Sclerosis
Currier	1993	13062	27231	methotrexa	Multiple Sclerosis
Curtis	2009	11788	7240	nabilone	Huntingtons Disease
Cutler	1985	12089	7154	zimeldine	Alzheimers Disease
Cutler	1985	12625	7158	l363,568	Alzheimers Disease
Cutler	1993	12414	6848	milacemide	Alzheimers Disease
da Silva-Jur	2005	11994	1123	levodopa/c	Parkinsons Disease
da Silva-Jur	2005	11995	1123	levodopa/c	Parkinsons Disease
Dallocchio	1999	12596	7513	risperidone	Huntingtons Disease
Dallos	1970	12024	3712	amantadine	Parkinsons Disease
Dallos	1972	12003	3621	amantadine	Parkinsons Disease
Danielczyk	1981	13202	15552	clopenthixo	Parkinsons Disease
Datieva	2013	16955	1836	melatonin	Parkinsons Disease
Dautzenbe	2006	11110	4747	memantine	Alzheimers Disease
Davidson	1988	12103	7091	4-aminopyr	Alzheimers Disease
Davidson	1989	12108	7067	physostigm	Alzheimers Disease
Davis	1990	12558	10596	4-aminopyr	Multiple Sclerosis
de	2008	12182	4402	indometha	Alzheimers Disease
De Carvalh	2010	11092	7705	memantine	MND
De Carvalh	2010	16909	4353	memantine	MND
De Deyn	2012	16676	580	quetiapine	Alzheimers Disease
de Tommas	2004	11819	7396	rivastigmin	Huntingtons Disease
de Tommas	2005	11808	7358	levetiraceta	Huntingtons Disease
de Tommas	2007	11796	7328	rivastigmin	Huntingtons Disease
Dean	1969	12534	10946	imipramine	Multiple Sclerosis
Debove	2001	11977	7966	riluzole	MND
Del	1995	13258	19606	levodopa/c	Parkinsons Disease
del Ser	2013	16867	228	tideglusib	Alzheimers Disease
Delwaide	1986	13137	7134	phosphatid	Alzheimers Disease
Demarin	2004	12577	5256	nicotinamic	Alzheimers Disease
Denber	1969	12696	3770	mefexamid	Parkinsons Disease
Denber	1969	12697	3770	procyclidin	Parkinsons Disease
Dennler	2001	13274	21143	rivastigmin	Alzheimers Disease
Derakshanc	2013	19019	561	vitamin d3	Multiple Sclerosis
Deroover	1984	12427	7616	tiapride	Huntingtons Disease
Derouesne	1997	13082	19992	s12024-2	Alzheimers Disease
Desai	1998	12542	8072	riluzole	MND
Desnulle	2001	11979	7996	vitamin e	MND
Deuschl	2007	12916	13542	entacapone	Parkinsons Disease
Deuschl	2007	12917	13542	entacapone	Parkinsons Disease
Devanand	2012	16881	807	risperidone	Alzheimers Disease
Devanand	2012	16934	286	risperidone	Alzheimers Disease
Devonshire	2012	16980	449	fingolimod	Multiple Sclerosis
Diamond	2013	16675	468	ginkgo bilol	Multiple Sclerosis

For peer review only

Diamond	2013	16857	122	ginkgo bilol	Multiple Sclerosis
DiFrancisco	2012	16874	1864	vitamin b a	Parkinsons Disease
Dissing	1989	13140	3098	tetrahydrol	Parkinsons Disease
Dobato	1994	13171	27283	amantadine	MND
Dobato	1994	13172	27283	amantadine	Parkinsons Disease
Dobato	1994	13173	27283	amantadine	Multiple Sclerosis
Doody	2008	12183	4271	dimebon	Alzheimers Disease
Doody	2012	16677	1875	donepezil	Alzheimers Disease
Doody	2013	16682	654	semagacest	Alzheimers Disease
Doraiswam	1996	12360	6583	nsaids	Alzheimers Disease
Dowling	2003	13278	22030	melatonin	Parkinsons Disease
Dowling	2005	11996	1144	melatonin	Parkinsons Disease
Dowzenko	1976	13396	3465	oxprenolol	Parkinsons Disease
Drory	2002	11984	7953	creatine	MND
Drymiotis	1976	13372	11279	mesoridazi	Huntingtons Disease
Dubinsky	2006	11814	7362	cysteamine	Huntingtons Disease
Dubios	2012	16918	663	v0191	Alzheimers Disease
Dubois	1998	12564	10168	d-penicillar	Multiple Sclerosis
Dubois	2012	16678	313	donepezil	Parkinsons Disease
Duffy	1987	12639	7105	lecithin	Alzheimers Disease
Dujardin	2006	11967	867	rivastigmin	Parkinsons Disease
Dupont	1997	12062	2419	tolcapone	Parkinsons Disease
Dupont	1997	12327	2419	tolcapone	Parkinsons Disease
Dursun	2000	12543	7488	antipsychot	Huntingtons Disease
Dworkin	1984	12672	10777	linoleic acic	Multiple Sclerosis
Dysken	1982	12614	7190	lecithin	Alzheimers Disease
Dysken	1992	12650	6907	milacemide	Alzheimers Disease
Dysken	2002	12356	5714	ondansetro	Alzheimers Disease
Dzudza	1974	12759	3530	amantadine	Parkinsons Disease
Dzudza	1974	12760	3530	parkopan (t	Parkinsons Disease
Eagger	1991	13043	19192	tacrine	Alzheimers Disease
Eagger	1994	12886	11013	tacrine	Alzheimers Disease
Eberhardt	1990	12076	3018	citicoline	Parkinsons Disease
Ebersbach	2010	12845	13083	tolcapone	Parkinsons Disease
Eckford	1995	12855	16377	desmopres	Multiple Sclerosis
Edwards	2005	13119	22941	rivastigmin	Alzheimers Disease
Eggert	2010	11999	85	perampane	Parkinsons Disease
Eisen	1993	11968	8209	lamotrigine	MND
Elfont	2009	13110	27337	tolperisone	Multiple Sclerosis
Ellis	1997	16943	1536	co-enz q/vi	MND
Ellison	1986	13064	27340	azathioprin	Multiple Sclerosis
Emre	2010	11097	23	memantine	Parkinsons Disease
Engel	1992	12143	6903	phosphatid	Alzheimers Disease
Esmaeilzad	2011	16824	941	pridopidine	Huntingtons Disease
Espay	2011	13160	13046	methylpher	Parkinsons Disease
Espir	1970	13065	27352	carbamaze	Multiple Sclerosis
Estadieu	1998	12974	12410	tacrine	Alzheimers Disease
Etienne	1981	12605	7197	lecithin	Alzheimers Disease
Evans	2000	12911	20830	donepezil	Alzheimers Disease
Evers	2003	13114	22300	misoprosto	Multiple Sclerosis

For peer review only

evidente	1999	12425	2258	rimantadin	Parkinsons Disease
Fabbrini	2002	12044	1773	donepezil	Parkinsons Disease
Factor	1988	13907	15440	pergolide	Parkinsons Disease
Factor	1989	13041	19173	levodopa/c	Parkinsons Disease
Factor	1989	13042	19173	levodopa/c	Parkinsons Disease
Factor	1990	12589	3055	vitamin e	Parkinsons Disease
Factor	1994	13117	15217	clozapine	Parkinsons Disease
Faglioni	1975	12757	3491	l-dopa/ ro4	Parkinsons Disease
Fahn	1972	12646	7685	perphenazi	Huntingtons Disease
Fahn	1975	12714	3486	amantadine	Parkinsons Disease
Fahn	1991	12015	3002	tocopherol	Parkinsons Disease
Fakouhi	1995	12405	6658	cycloserine	Alzheimers Disease
Falah	2012	16680	372	levetiraceta	Multiple Sclerosis
Fareed	1971	12567	8282	isoprinosin	MND
Farlow	2011	16686	1028	donepezil	Alzheimers Disease
Fedorova	2003	13182	14287	piribedil	Parkinsons Disease
Fedorova	2005	13388	13992	tolcapone	Parkinsons Disease
Fedorova	2006	13176	13830	levodopa/c	Parkinsons Disease
Fehling	1972	12690	3649	amantadine	Parkinsons Disease
Fehling	1973	12683	3595	amantadine	Parkinsons Disease
Feldman	2006	11118	4625	memantine	Alzheimers Disease
Feldman	2010	12220	3922	atorvastati	Alzheimers Disease
Ferel	1972	13222	15683	levodopa/r	Parkinsons Disease
Fernandez	2010	11587	243	istradefyllir	Parkinsons Disease
Fernandez	2000	11843	7500	donepezil	Huntingtons Disease
Fernández-	2011	16910	4382	dutasteride	MND
Ferrante	2005	11911	7855	coenzyme (	MND
Ferreira	2010	12878	13200	nebicapone	Parkinsons Disease
Field	1983	13066	27385	linoleic acic	Multiple Sclerosis
Field	1983	13067	27385	arachidonic	Multiple Sclerosis
Fieschi	1970	12279	3725	amantadine	Parkinsons Disease
Fieschi	1970	12761	3741	amantadine	Parkinsons Disease
Fieschi	1970	13227	15716	levodopa/a	Parkinsons Disease
Figiel	2008	12949	24652	rivastigmin	Alzheimers Disease
Figiel	2008	13128	24619	rivastigmin	Alzheimers Disease
Finsterer	1999	11898	8053	riluzole	MND
Fischer	1984	12997	15527	levodopa/b	Parkinsons Disease
Fischer	1974	13375	3549	melanocyte	Parkinsons Disease
Fischer	1984	12995	15527	levodopa/b	Parkinsons Disease
Fischhof	1992	12659	6929	pyritinol	Alzheimers Disease
Fischhof	1996	12635	6616	vincamine	Alzheimers Disease
Fisher	1974	12306	7676	gaba	Huntingtons Disease
Fisman	1981	12366	7199	2-dimethyla	Alzheimers Disease
Fisman	1981	12598	7198	lecithin	Alzheimers Disease
Fitten	1990	12363	7017	tha + lecith	Alzheimers Disease
Fleischhack	1986	11597	7141	memantine	Alzheimers Disease
Fleisher	2008	12184	4257	ly450139	Alzheimers Disease
Fleisher	2011	16690	1933	divalproex	Multiple Sclerosis
Fleming	2000	19078	733	19-nor-1,25	Multiple Sclerosis
Fleming	2011	16684	904	trichuris su	Multiple Sclerosis

For peer review only

Flet	2010	13899	8431	3,4-diamino	Multiple Sclerosis
Flowers	2013	16936	2901	temozolom	Multiple Sclerosis
Fogelson	2003	12315	1655	rivastigmin	Parkinsons Disease
Forbes	1979	16900	2474	baclofen	MND
Foster	1983	12581	7621	4,5,6,7-tetr	Huntingtons Disease
Foster	1996	12630	6551	tacrine	Alzheimers Disease
Foster	1996	12631	6551	lecithin	Alzheimers Disease
Foster	1996	12632	6551	tacrine & le	Alzheimers Disease
Foster	1961	13068	27426	tolbutamid	Multiple Sclerosis
Fox	2004	13174	27433	cannabis oi	Multiple Sclerosis
Fox	2012	16679	310	bg-12	Multiple Sclerosis
Fox	2012	16691	1942	memantine	Alzheimers Disease
Frackiewicz	2002	13332	1840	levodopa/c	Parkinsons Disease
Frakey	2012	16681	400	modafinil	Alzheimers Disease
Frank	2008	12418	7287	tetrabenazi	Huntingtons Disease
Frank	2009	11786	7233	tetrabenazi	Huntingtons Disease
Freedman	1971	12361	3678	amantadine	Parkinsons Disease
Freund-Lev	2006	12547	4660	omega 3 fa	Alzheimers Disease
Freund-Lev	2008	12185	4508	omega 3 fa	Alzheimers Disease
Freund-Lev	2009	12199	4095	omega 3 fa	Alzheimers Disease
Friedman	1999	12944	20625	clozapine	Parkinsons Disease
Frolich	2011	13038	19054	azd3480	Alzheimers Disease
Frolich	2011	13039	19054	donepezil	Alzheimers Disease
Funfgeld	1970	13379	3717	amantadine	Parkinsons Disease
Funfgeld	1989	13923	7066	phosphatid	Alzheimers Disease
Fuschillo	2001	12848	21150	donepezil	Alzheimers Disease
Fuschillo	2001	12849	21150	rivastigmin	Alzheimers Disease
Gambi	1983	13249	19097	dantrolene	Multiple Sclerosis
Garcia de Y	2011	16683	721	pridopidine	Huntingtons Disease
Garren	2009	13111	27482	bht-3009	Multiple Sclerosis
Gasparani	1997	12006	2518	tolcapone	Parkinsons Disease
Gauthier	1990	12115	7010	lecithin/tet	Alzheimers Disease
Gauthier	2009	12353	4072	tramiprosat	Alzheimers Disease
Gauthier	2004	13282	22439	donepezil	Alzheimers Disease
Gavrilova	2009	13149	25210	galantamin	Alzheimers Disease
Gehrman	2009	12205	4165	melatonin	Alzheimers Disease
Geisler	1996	12497	10366	amantadine	Multiple Sclerosis
Geisler	1996	12498	10366	pemoline	Multiple Sclerosis
Geldmache	2011	12370	3832	pioglitazon	Alzheimers Disease
Gelfin	2012	16848	1972	d-serine	Parkinsons Disease
Geminiani	1996	12937	19834	cabergoline	Parkinsons Disease
Gerlach	1976	12652	3453	benserazide	Parkinsons Disease
Gerlach	1976	12658	3453	cb 154 (2-b	Parkinsons Disease
Ghezzi	1989	13287	4112	azathioprin	Multiple Sclerosis
Gil	1992	16945	2047	amino acid	MND
Giladi	2003	12034	1539	rivastigmin	Parkinsons Disease
Gilligan	1970	13358	3710	amantadine	Parkinsons Disease
Gilligan	1972	13905	7683	tetrabenazi	Huntingtons Disease
Gimenez-Ro	1989	12750	7595	tetrabenazi	Huntingtons Disease
Gimenez-Ro	1989	12751	7595	haloperidol	Huntingtons Disease

For peer review only

Gimenez-R	2003	13180	14253	quetiapine	Parkinsons Disease
Giovannoni	2010	12927	18839	cladribine	Multiple Sclerosis
Gironi	2008	12484	8666	naltrexone	Multiple Sclerosis
Giuffra	1992	11862	7583	milacemide	Huntingtons Disease
Giuffra	1993	12340	2898	milacemide	Parkinsons Disease
Godwin-Au	1970	12025	3722	amantadine	Parkinsons Disease
Goebel	1995	13342	6680	estrogen	Alzheimers Disease
Goety	1990	12373	7593	acetyl-l-car	Huntingtons Disease
Goetz	2007	11738	822	levodopa/s	Parkinsons Disease
Golan	2013	19031	819	vitamin d3	Multiple Sclerosis
Gold	2010	12221	3839	rosiglitazon	Alzheimers Disease
Goldberg	1986	12922	16015	vitamin d	Multiple Sclerosis
Gonce	1985	13250	19113	pergolide	Parkinsons Disease
Gonsette	1993	13070	27536	isoprinosin	Multiple Sclerosis
Goodkin	1992	13365	10540	methotrexa	Multiple Sclerosis
Goodkin	1995	12575	10441	mitoxantro	Multiple Sclerosis
Goodkin	1996	12488	10305	methotrexa	Multiple Sclerosis
Goodman	2007	12505	8927	4-aminopyr	Multiple Sclerosis
Goodman	2008	12485	8674	4-aminopyr	Multiple Sclerosis
Goodman	2009	12479	8582	4-aminopyr	Multiple Sclerosis
Goodman	2010	12453	8298	4-aminopyr	Multiple Sclerosis
Goonetillek	1995	11865	8179	rx77368	MND
Gordon	2004	11914	7906	minocycline	MND
Gordon	2007	11875	7783	minocycline	MND
Gordon	2008	12254	7761	minocycline	MND
Gordon	2008	12255	7761	celecoxib /	MND
Gordon	2006	16978	3432	minocycline	MND
Gordon	2007	16903	3452	minocycline	MND
Gorlich	1972	13020	15675	levodopa/r	Parkinsons Disease
Gottschalk	1965	12538	10969	imipramine	Multiple Sclerosis
Goulley	1993	13045	19256	levodopa/b	Parkinsons Disease
Grace	2009	11642	409	galantamin	Parkinsons Disease
Graf	2005	11910	7891	vitamin e	MND
Gredal	1997	11893	8107	dextrometh	MND
Green	1992	12146	6890	oxiracetam	Alzheimers Disease
Green	2009	12206	3970	tarenflurbil	Alzheimers Disease
Griffiths	1971	12691	3657	amantadine	Parkinsons Disease
Groeneveld	2003	11917	7933	creatine	MND
Groeneveld	2003	11918	7915	riluzole	MND
Groeneveld	2005	12552	7879	creatine	MND
Grossberg	2011	16693	1057	rivastigmin	Alzheimers Disease
Grossmann	1990	12667	6995	naftidrofur	Alzheimers Disease
Groudon	1978	13904	7642	choline	Huntingtons Disease
Growdon	1986	12095	7128	piracetam 8	Alzheimers Disease
Grundman	2003	12141	5492	leteprinim (	Alzheimers Disease
Guan	2012	16694	696	vitamin e	Alzheimers Disease
Gutzmann	1998	12231	6221	idebenone	Alzheimers Disease
Gutzmann	2002	12190	5743	idebenone	Alzheimers Disease
Gutzmann	2002	12191	5743	idebenone	Alzheimers Disease
Haan	2004	12352	5103	egb- 761	Alzheimers Disease

For peer review only

Haas	1994	13074	27598	ciclosporin	Multiple Sclerosis
Haas	1994	13077	27598	azathioprin	Multiple Sclerosis
Hager	2007	12576	4443	lipoic acid	Alzheimers Disease
Hampel	2009	12330	4060	lithium	Alzheimers Disease
Hanagasi	2011	16695	830	rasagiline	Parkinsons Disease
Hansen	1965	12679	3806	tolbutamid	Parkinsons Disease
Hanyu	2009	12210	4163	pioglitazon	Alzheimers Disease
Haque	1994	11852	8201	tolperisone	MND
Harkins	1996	12930	12624	tacrine	Alzheimers Disease
Harrington	2011	13155	26640	rosiglitazon	Alzheimers Disease
Harrington	2011	13156	26640	rosiglitazon	Alzheimers Disease
Harrington	2011	16866	838	rosiglitazon	Alzheimers Disease
Haslam	1967	11926	7690	penicillamin	Huntingtons Disease
Hass	2007	12343	756	creatine m	Parkinsons Disease
Hassan	1980	13003	27624	ds103-282	Multiple Sclerosis
Hassan	1980	13002	27624	baclofen	Multiple Sclerosis
Hauser	2003	12038	1586	istradefyllir	Parkinsons Disease
Hauser	2008	11650	422	istradefyllir	Parkinsons Disease
Hauser	2010	12910	13082	pramipexol	Parkinsons Disease
Hauser	2011	12933	26724	ropinirole	Parkinsons Disease
Hauser	2011	16697	1047	preladenan	Parkinsons Disease
Hauser	2011	16698	777	ipx066	Parkinsons Disease
Hauser	2011	16699	777	levodopa	Parkinsons Disease
Hayashi	2010	12223	3927	yokukansar	Alzheimers Disease
Hayton	2012	16702	2049	lamotrigine	Multiple Sclerosis
Heckmann	2004	11821	7400	amantadine	Huntingtons Disease
Henderson	1994	12851	19292	estrogen	Alzheimers Disease
Henderson	1996	12248	6581	estrogen	Alzheimers Disease
Henderson	2000	12215	6050	estrogen	Alzheimers Disease
Henderson	2009	12955	25304	ac-1202	Alzheimers Disease
Heo	2008	12718	4260	ginseng	Alzheimers Disease
Herrmann	2004	12105	5204	pindolol	Alzheimers Disease
Hersch	2006	11807	7354	creatine	Huntingtons Disease
Herskovits	1973	12803	3596	fusaric acid	Parkinsons Disease
Heyman	1987	13363	7115	lecithin	Alzheimers Disease
Hoagland	1997	16942	1154	cntf	MND
Hoglund	2005	12100	5000	simvastatin	Alzheimers Disease
Hohol	1996	12495	10359	myelin	Multiple Sclerosis
Hohol	1997	13004	27676	linomide	Multiple Sclerosis
Holl	2010	11787	7235	venlafaxine	Huntingtons Disease
Hollander	1994	11858	8183	dextrometh	MND
Hollander	1987	12101	7104	rs 86	Alzheimers Disease
Holthoff	2013	16704	170	memantine	Alzheimers Disease
Hong	2006	13122	23474	galantamin	Alzheimers Disease
Horton	2013	16705	57	4-aminopyr	Multiple Sclerosis
Hoverd	1998	13175	27688	desmopres	Multiple Sclerosis
Howard	2012	16707	2073	donepezil	Alzheimers Disease
Howard	2012	16706	2073	donepezil/r	Alzheimers Disease
Hu	2006	13325	4737	donepezil	Alzheimers Disease
Hu	2003	13331	1524	nuzhen yan	Parkinsons Disease

For peer review only

Hueber	1996	13262	19863	amantadin	Parkinsons Disease
Hughes	1988	13006	27700	azathioprin	Multiple Sclerosis
Hugon	1996	16938	1065	riluzole	MND
Hunter	1970	12282	3716	amantadin	Parkinsons Disease
Huntington	2001	11839	7474	coenzyme (	Huntingtons Disease
Huntington	2001	11841	7474	remacemid	Huntingtons Disease
Huntington	2003	11823	7416	riluzole	Huntingtons Disease
Huntington	2004	11822	7401	minocyclin	Huntingtons Disease
Huntington	2006	11804	7352	tetrabenazi	Huntingtons Disease
Huntington	2010	11783	7214	minocyclin	Huntingtons Disease
Huntington	2008	11792	7270	ethyl-epa	Huntingtons Disease
Huolman	2011	16708	759	rivastigmin	Multiple Sclerosis
Hutchinson	1996	12008	2564	tacrine	Parkinsons Disease
Hutton	1993	13186	15252	cabergoline	Parkinsons Disease
Hyman	1985	12092	7148	naltrexone	Alzheimers Disease
Iavarone	2000	13272	20763	donepezil	Alzheimers Disease
Irving	2009	13032	18710	docosahexa	Alzheimers Disease
Isik	2012	16710	2091	donepezil	Alzheimers Disease
Iwasaki	2012	16932	238	yokukansar	Huntingtons Disease
Jack	2003	12251	5557	ci-979 (mila	Alzheimers Disease
Jacob	2002	13104	21712	melatonin	MND
Janik	1996	16944	1555	selegiline	MND
Jankovic	1982	12308	7627	tetrabenazi	Huntingtons Disease
Jankovic	1983	16979	1516	pergolide	Parkinsons Disease
Jankovic	2002	12046	1788	riluzole	Parkinsons Disease
Jankovic	2009	11607	299	atomoxetin	Parkinsons Disease
Jansen	1994	13080	19384	clozapine	Parkinsons Disease
Jelic	2008	12909	24616	donepezil	Alzheimers Disease
Jenike	1986	12637	7132	lecithin anc	Alzheimers Disease
Jennekens-	1985	12084	7163	desglycinar	Alzheimers Disease
Jerusalem	1968	13232	15751	baclofen	Multiple Sclerosis
Joerger	2012	16865	401	thc	MND
Johansson	2011	16712	1059	memantine	Parkinsons Disease
Johnson	1995	13007	27764	d1	Multiple Sclerosis
Jolkkonen	1985	12417	7152	org 2766	Alzheimers Disease
Jones	2007	11105	4594	memantine	Alzheimers Disease
Jones	2008	12333	4278	donepezil/a	Alzheimers Disease
Jones	2008	12334	4278	donepezil	Alzheimers Disease
Jones	1983	13040	19099	4-aminopyr	Multiple Sclerosis
Jorga	2000	12873	20904	tolcapone	Parkinsons Disease
Jorgensen	1971	13897	3677	amantadin	Parkinsons Disease
Jori	1990	13190	15370	cabergoline	Parkinsons Disease
Jossan	1994	12566	8206	selegiline	MND
Kakuta	2012	16713	2941	riluzole	MND
Kalkers	2002	12510	9637	riluzole	Multiple Sclerosis
Kalra	1998	12271	8081	riluzole	MND
Kamphius	2011	16714	771	souvenaid	Alzheimers Disease
Kampman	2012	16846	351	vitamin d3	Multiple Sclerosis
Kano	2008	12951	24822	pramipexol	Parkinsons Disease
Kanowski	1990	12675	7011	xantinolnic	Alzheimers Disease

For peer review only

Kanowski	1996	12634	6593	ginkgo bilol	Alzheimers Disease
Kanowski	2003	13359	5358	egb- 761	Alzheimers Disease
Kapoor	2010	11083	8338	lamotrigine	Multiple Sclerosis
Kapoor	2010	12459	8338	lamotrigine	Multiple Sclerosis
Kappos	1987	13012	27801	ciclosporin	Multiple Sclerosis
Kappos	1987	13013	27801	azathioprin	Multiple Sclerosis
Kappos	1988	13014	27802	azathioprin	Multiple Sclerosis
Kappos	1988	13015	27802	ciclosporin	Multiple Sclerosis
Kappos	2008	12482	8630	fumarate (t	Multiple Sclerosis
Kappos	2012	16715	2128	bg-12	Multiple Sclerosis
Kappos	2012	16917	570	bg-12	Multiple Sclerosis
Kartzinel	1976	11901	7663	bromocript	Huntingtons Disease
Karussis	1995	13018	27822	linomide	Multiple Sclerosis
Karussis	1996	12492	10320	linomide	Multiple Sclerosis
Kastrukoff	1978	13179	27828	antithymoc	Multiple Sclerosis
Katunina	2008	11670	485	himantane	Parkinsons Disease
Katzenschla	2004	12323	1329	levodopa/c	Parkinsons Disease
Katzenschla	2004	12324	1329	mucuna pru	Parkinsons Disease
Kaufer	1996	13260	19803	tacrine	Alzheimers Disease
Kaufmann	2009	11949	7729	coenzyme q	MND
Kavia	2006	12941	27838	cannabidiol	Multiple Sclerosis
Keller	2011	16716	1127	galantamin	Alzheimers Disease
Kennelly	2011	16717	785	nilvadipine	Alzheimers Disease
Kenney	2007	11799	7333	tetrabenazi	Huntingtons Disease
Khatri	1983	13019	27855	cyclophosp	Multiple Sclerosis
Khatri	2011	16882	908	fingolimod	Multiple Sclerosis
Kieburtz	1994	13919	2806	deprenyl	Parkinsons Disease
Kieburtz	1996	11859	7547	remacemid	Huntingtons Disease
Kieburtz	1997	12840	4177	entacapone	Parkinsons Disease
Kieburtz	2008	12411	504	creatine	Parkinsons Disease
Kieburtz	2008	12412	504	minocycline	Parkinsons Disease
Kieburtz	2010	11785	7230	latrepirdine	Huntingtons Disease
Kieburtz	2011	12902	13065	pramipexol	Parkinsons Disease
Killestein	2000	12945	27869	tetrahydroc	Multiple Sclerosis
Killestein	2000	12946	27869	cannabis ex	Multiple Sclerosis
Killestein	2005	12462	9253	riluzole	Multiple Sclerosis
Kim	2011	16719	2148	ginseng	Multiple Sclerosis
Kim	2009	13113	27874	ginseng	Multiple Sclerosis
Kimball	2007	19021	1200	vitamin d3	Multiple Sclerosis
Kinali	2002	11942	7945	albuterol	MND
Kinn	1990	12924	16193	desmopres	Multiple Sclerosis
Kiseleva	1973	12754	3591	amantadine	Parkinsons Disease
Kline	1974	13374	3529	biperiden	Parkinsons Disease
Knezevic	1989	12107	7068	pyritinol	Alzheimers Disease
Knopman	2001	12196	5797	trimethylgl	Alzheimers Disease
Koetsier	1988	12860	27895	ciclosporin	Multiple Sclerosis
Kojima	2013	16922	2955	bupropion	Multiple Sclerosis
Koller	1982	13386	11235	estrogen	Huntingtons Disease
Koller	1991	12435	2972	mk-458 (hp	Parkinsons Disease
Kondo	2002	13275	21572	selegiline	Parkinsons Disease

For peer review only

Kontush	2001	12200	5814	vitamin e	Alzheimers Disease
Kontush	2001	12201	5814	vitamin c /	Alzheimers Disease
Kopff	1996	13263	19927	adrenocort	Multiple Sclerosis
Korchounov	2012	16837	2169	rasagiline	Parkinsons Disease
Korten	1973	12676	3580	cyclobenza	Parkinsons Disease
Korwin-Piot	1992	12931	19232	padma 28	Multiple Sclerosis
Kostic	2012	16720	234	fluoxetine	Parkinsons Disease
Kravitz	1974	12280	3517	amantadine	Parkinsons Disease
Kremer	1999	11079	7506	lamotrigine	Huntingtons Disease
Krupp	1993	13028	27931	pemoline	Multiple Sclerosis
Krupp	1995	12500	10393	amantadine	Multiple Sclerosis
Krupp	1995	12502	10393	pemoline	Multiple Sclerosis
Krupp	2004	13181	27933	donepezil	Multiple Sclerosis
Krupp	2011	13246	18994	donepezil	Multiple Sclerosis
Kulisevsky	2002	12265	1837	theophyllin	Parkinsons Disease
Kulua	2011	16983	1153	entacapone	Parkinsons Disease
Kume	2012	16871	2185	telmisartan	Alzheimers Disease
Kume	2012	16872	2185	amlodipine	Alzheimers Disease
Kuroiwa	1967	12959	27944	carbamazep	Multiple Sclerosis
Kurth	1997	12070	2541	tolcapone	Parkinsons Disease
Kwak	2012	16723	2187	l-dopa	Parkinsons Disease
Kwak	2012	16724	2187	carbidopa	Parkinsons Disease
Kwiecinski	2001	11981	7971	selegiline	MND
Kyomen	2007	12402	4494	levetiraceta	Alzheimers Disease
La Mantia	1997	12960	27953	cyclophosp	Multiple Sclerosis
Lacomblez	1989	16940	479	gangloiside	MND
Lacomblez	1996	11872	8140	riluzole	MND
Lacomblez	1996	11889	8126	riluzole	MND
Lacomblez	1989	16963	1871	gangloiside	MND
Lacomblez	2002	11985	7956	riluzole	MND
Lacomblez	2002	12740	7948	riluzole	MND
Lacomblez	2004	11957	7904	xaliproden	MND
Laitinen	1971	13896	3667	amantadine	Parkinsons Disease
Landwehrm	2007	11795	7315	riluzole	Huntingtons Disease
Lang	1995	12869	19668	remoxiprid	Parkinsons Disease
Lang	2002	13387	21470	galantamin	Alzheimers Disease
Lange	1998	11896	8094	selegiline	MND
Lange	1998	16895	1230	selegiline	MND
Lange	2009	12477	8558	modafinil	Multiple Sclerosis
Lannaccone	1996	11973	8127	fluvoxamin	MND
Lannfelt	2008	12186	4263	pbt2	Alzheimers Disease
Larsen	2003	12870	22018	entacapone	Parkinsons Disease
Larsson	2010	12891	13101	memantine	Parkinsons Disease
Larsson	2011	16884	2202	memantine	Parkinsons Disease
Lawlor	1991	12132	6950	meta-chlor	Alzheimers Disease
Lawlor	1994	12903	19334	trazodone	Alzheimers Disease
Le Bars	1997	12237	6405	egb- 761	Alzheimers Disease
Le Bars	2000	12290	5972	gingko bilol	Alzheimers Disease
Le Bars	2003	12129	5405	egb- 761	Alzheimers Disease
Le Page	2011	16726	2206	mitoxantro	Multiple Sclerosis

For peer review only

Lee	2008	12400	4298	panax ginse	Alzheimers Disease
Lees	2007	12982	13526	tolcapone	Parkinsons Disease
Lees	2012	16727	2212	perampane	Parkinsons Disease
Lehmann	1997	12859	27980	linomide	Multiple Sclerosis
Lehr	2007	12778	4573	tesofensine	Alzheimers Disease
Lenz	2009	12837	25228	pozanicline	Alzheimers Disease
Leonard	1974	13025	15818	haloperidol	Huntingtons Disease
Leonard	1974	11939	7672	haloperidol	Huntingtons Disease
Leonard	1974	13023	15818	lithium	Huntingtons Disease
Leonard	1975	12376	7669	haloperidol	Huntingtons Disease
Leonard	1975	12377	7669	lithium	Huntingtons Disease
Leroi	2009	11093	293	memantine	Parkinsons Disease
Leroi	2004	12031	1508	donepezil	Parkinsons Disease
Leszek	1999	12599	6063	colostrinin	Alzheimers Disease
Leszek	1999	12600	6063	selenium	Alzheimers Disease
Leszek	2002	12443	5603	colostrinin	Alzheimers Disease
Levin	2008	12729	344	memantine	Parkinsons Disease
Levin	2006	13169	13828	tianeptine	Parkinsons Disease
Levin	2010	13164	13220	pramipexol	Parkinsons Disease
Levine	1969	12533	10941	diazepam	Multiple Sclerosis
Levinson	1985	12627	7151	buflomedil	Alzheimers Disease
Levitsky	2005	12738	7877	amitriptylir	MND
Levy	2006	11899	7847	coenzyme (	MND
Levy	1983	12074	7182	lecithin	Alzheimers Disease
LeWitt	2008	11696	557	istradefyllir	Parkinsons Disease
Leyhe	2009	12335	4133	lithium	Alzheimers Disease
Li	2011	16951	1055	memantine	Parkinsons Disease
Li	2012	16868	648	hirudin	Alzheimers Disease
Li	2011	16956	2220	bushenhuo	Parkinsons Disease
Li	2012	16947	496	bushenhuo	Parkinsons Disease
Li	2012	16950	928	bushenhuo	Parkinsons Disease
Liang	2008	11860	7779	hydroxyure	MND
Lieberman	1977	13886	3405	lergotrile	Parkinsons Disease
Lieberman	1983	12998	15538	lisuride	Parkinsons Disease
Lin	2003	13326	5400	tiaoxin reci	Alzheimers Disease
Lin	2003	13327	5400	bushen rec	Alzheimers Disease
Lin	2003	13323	5400	donepezil	Alzheimers Disease
Linazasoro	2005	11997	1151	donepezil	Parkinsons Disease
Linazasoro	2005	12420	1111	donepezil	Parkinsons Disease
Little	1985	12090	7153	lecithin	Alzheimers Disease
Litvinenko	2007	11693	525	galantamin	Parkinsons Disease
Litvinenko	2009	13151	25211	rivastigmin	Alzheimers Disease
Litvinenko	2010	11094	150	memantine	Parkinsons Disease
Litvinenko	2008	11640	397	galantamin	Parkinsons Disease
Litvinenko	2012	16948	617	clonazepan	Parkinsons Disease
Litvinenko	2012	16949	617	melatonin	Parkinsons Disease
Liversedge	1956	16897	1630	glycocyami	MND
Loeb	2004	12116	5302	doxycycline	Alzheimers Disease
Logothetis	2005	12518	9139	hydroxyzin	Multiple Sclerosis
Lomen-Hoe	2007	16930	3526	hydroxyure	MND

For peer review only

Lopez-Pous	2008	12950	24667	trazodone	Alzheimers Disease
Lorenzi	2011	16729	1102	memantine	Alzheimers Disease
Lorenzi	2011	16829	1102	memantine	Alzheimers Disease
Lou	2009	12408	242	modafinil	Parkinsons Disease
Lovera	2010	11090	8365	memantine	Multiple Sclerosis
Lovera	2012	16730	319	gingko bilol	Multiple Sclerosis
Lucetti	2002	11830	7441	amantadine	Huntingtons Disease
Lucetti	2003	11933	7429	amantadine	Huntingtons Disease
Lugaresi	2001	12463	9779	methotrexa	Multiple Sclerosis
Luginger	2000	12060	2024	amantadine	Parkinsons Disease
Lundin	2010	11784	7219	pridopidine	Huntingtons Disease
Lyons	2009	12912	13243	pramipexol	Parkinsons Disease
Lyytinen	2000	13091	20903	selegiline	Parkinsons Disease
Macdonald	2008	12187	4412	lithium	Alzheimers Disease
Mace	2010	12407	239	amino acid	Parkinsons Disease
Mace	2010	12981	13103	5-ht deplet	Parkinsons Disease
Maciejek	1989	13241	16161	amantidine	Multiple Sclerosis
Maciejek	1997	12962	28055	isoprinosin	Multiple Sclerosis
Mackenzie	1978	13204	15580	bromocript	Parkinsons Disease
MacManus	2011	16731	1046	bg-12	Multiple Sclerosis
maher-edw	2010	12562	3964	sb-742457	Alzheimers Disease
maher-edw	2011	16733	955	sb-742457	Alzheimers Disease
maher-edw	2011	16734	955	donepezil	Alzheimers Disease
Mainero	2002	13188	28066	3,4-diamino	Multiple Sclerosis
Malin	2008	12964	28069	creatine	Multiple Sclerosis
Mally	1995	13047	19610	selegiline	Parkinsons Disease
Malsch	2001	12983	14501	amantadine	Parkinsons Disease
Malsch	2001	12985	14501	budipine	Parkinsons Disease
Maltby	1994	12161	6805	lecithin	Alzheimers Disease
Maltby	1994	12167	6805	tacrine & le	Alzheimers Disease
Mannen	1991	12988	15310	bromocript	Parkinsons Disease
Mannen	1991	12989	15310	bromocript	Parkinsons Disease
Mannie	1971	12692	3665	amantadine	Parkinsons Disease
Manson	2000	12066	2096	idazoxan	Parkinsons Disease
Manyam	1973	11087	7681	lithium	Huntingtons Disease
Manyam	1980	12429	7634	isoniazid / p	Huntingtons Disease
Manyam	1981	11883	7629	isoniazid	Huntingtons Disease
Manyam	1987	12291	7605	isoniazid / p	Huntingtons Disease
Manyam	1990	11879	7590	isoniazid	Huntingtons Disease
Marcus	2012	19173	712	vitamin d3	Multiple Sclerosis
Marcusson	1997	12616	6411	propentofy	Alzheimers Disease
Marder	2002	12261	1762	donepezil	Parkinsons Disease
Marforio	1984	13189	28081	adrenocort	Multiple Sclerosis
Marquet	2012	16735	298	safinamide	Parkinsons Disease
Marquet	2012	16736	298	phenelzine	Parkinsons Disease
Marquet	2012	16737	298	safinamide	Parkinsons Disease
Marsden	1973	13017	15659	levodopa/c	Parkinsons Disease
Marsden	1974	13016	15646	propranolo	Parkinsons Disease
Marsh	2009	11623	375	atomoxetin	Parkinsons Disease
Marti Mass	1986	12756	3229	cinnarizine	Parkinsons Disease

For peer review only

Martin	1974	12712	3524	trihexypher	Parkinsons Disease
Martin	1975	13010	15630	levodopa/c	Parkinsons Disease
Maruyama	2006	12156	4755	donepezil	Alzheimers Disease
Maruyama	2006	12372	4755	donepezil/l	Alzheimers Disease
Massaro	1990	12517	10580	levamisole	Multiple Sclerosis
Mattila	1970	13378	3700	prazitone (a	Parkinsons Disease
Mattsson	1973	11086	7680	lithium	Huntingtons Disease
Maurer	1997	12236	6377	egb- 761	Alzheimers Disease
Mawdsley	1972	13893	3620	amantadine	Parkinsons Disease
Mazza	2006	12273	4687	ginkgo bilol	Alzheimers Disease
Mazzini	1994	11845	8204	selegiline	MND
Mazzini	1998	11897	8073	gabapentin	MND
Mazzini	2001	12550	7974	creatine	MND
McCaffrey	1987	12099	7114	sulcotidil	Alzheimers Disease
McCarten	1995	12173	6657	triazolam	Alzheimers Disease
McCaul	1974	13011	15643	lithium	Parkinsons Disease
McGeer	1972	12367	3635	folic acid	Parkinsons Disease
McGuinnes	2002	12965	28111	cranberry ju	Multiple Sclerosis
McLean	1982	12719	7623	isoniazid	Huntingtons Disease
McLellan	1974	11921	7678	tetrabenazi	Huntingtons Disease
McLellan	1974	11922	7678	thiopropaza	Huntingtons Disease
Meador	1993	12434	15328	thiamine	Alzheimers Disease
Meara	1996	13261	19848	sertraline	Parkinsons Disease
Meco	1995	12884	19679	risperidone	Huntingtons Disease
Medaer	1999	13191	28118	prucaloprid	Multiple Sclerosis
Medeiros	2007	11722	732	melatonin	Parkinsons Disease
Mehta	2010	11232	8436	memantine	Multiple Sclerosis
Meininger	2004	11956	7903	xaliproden	MND
Meininger	2006	11881	7853	pentoxifylli	MND
Mejer	1983	12082	3289	tiapride	Parkinsons Disease
Mendis	1994	12932	19402	remoxiprid	Parkinsons Disease
Mendonca	2007	11713	657	methylpher	Parkinsons Disease
Mercuri	2004	11913	7909	phenylbuty	MND
Mercuri	2007	11874	7812	phenylbuty	MND
Merello	1999	11089	2160	memantine	Parkinsons Disease
Merlini	2002	16961	2146	gabapentin	MND
Merlini	2002	16962	2146	salbutamol	MND
Merlini	2003	11958	7922	gabapentin	MND
Merrick	1973	13377	3567	amantadine	Parkinsons Disease
Mertin	1999	13922	28130	bromelain/	Multiple Sclerosis
Mesnage	2004	12422	1427	sr141716	Parkinsons Disease
Mesnage	2004	12423	1427	sr 142801	Parkinsons Disease
Mesnage	2004	12424	1427	sr 48692	Parkinsons Disease
Metman	1999	12068	2145	amantadine	Parkinsons Disease
Mets	2007	12895	24005	rivastigmin	Alzheimers Disease
Meyer	2008	11855	7769	thalidomide	MND
Micheli	1986	12623	7611	lisuride	Huntingtons Disease
Mielke	1998	12234	6302	propentofy	Alzheimers Disease
Mielke	2012	16968	2312	memantine	Alzheimers Disease
Milanese	1988	13075	16111	azathioprin	Multiple Sclerosis

For peer review only

Milanese	1993	12514	10527	azathioprin	Multiple Sclerosis
Milanov	2001	12322	1917	levodopa/b	Parkinsons Disease
Millac	1967	12535	10961	atromid	Multiple Sclerosis
Millac	1969	12555	10943	cyclophosp	Multiple Sclerosis
Millac	1970	12283	3713	amantadine	Parkinsons Disease
Millar	1973	12532	10914	linoleic acic	Multiple Sclerosis
Millar	1984	13192	28148	linoleic acic	Multiple Sclerosis
Miller	2012	16739	2315	firategrast	Multiple Sclerosis
Miller	1996	11890	8125	gabapentin	MND
Miller	1996	11867	8148	nimodipine	MND
Miller	1996	11871	8145	verapamil	MND
Miller	1996	12274	8131	riluzole	MND
Miller	1996	16898	2053	nimodipine	MND
Miller	1998	16973	1641	riluzole	MND
Miller	2000	16926	1902	gabapentin	MND
Miller	2001	11983	8006	gabapentin	MND
Miller	2001	12549	7976	gabapentin	MND
Miller	2011	16740	816	lithium	MND
Miller	2007	11873	7792	tch346	MND
Miller	2007	16902	3413	tch346	MND
Miller	1993	12157	6855	org 2766	Alzheimers Disease
Miller	1961	12968	28169	prednisolor	Multiple Sclerosis
Miller	1961	12972	28169	aspirin	Multiple Sclerosis
Miller	1963	12857	28172	aspirin	Multiple Sclerosis
Miller	2012	16738	282	teriflunomi	Multiple Sclerosis
Mimori	1996	12175	6592	thiamine	Alzheimers Disease
Minett	2003	12033	1536	donepezil	Parkinsons Disease
Miret	2010	13194	28184	cladribine	Multiple Sclerosis
Missonnier	1999	12217	6092	s12024-2	Alzheimers Disease
Mitchell	1993	16894	758	selegiline	MND
Mitchell	1994	16965	3373	selegiline	MND
Mitchell	1995	12276	8163	selegiline	MND
mizukami	2006	12158	4779	milnaciprar	Alzheimers Disease
Mizukami	2009	12212	4184	tj-54	Alzheimers Disease
Mizuno	2010	11519	44	istradefyllir	Parkinsons Disease
Modrego	2010	11106	3995	memantine	Alzheimers Disease
Mohr	1989	12112	7053	clonidine	Alzheimers Disease
Mohr	1995	12171	6714	cycloserine	Alzheimers Disease
Mohr	1997	12239	6420	sabeluzole	Alzheimers Disease
Mohs	2009	12351	4022	atomoxetin	Alzheimers Disease
Molinari	1995	13334	2766	famotidine	Parkinsons Disease
Molinuevo	2011	12846	19059	donepezil	Alzheimers Disease
Moller	2011	16742	867	modafinil	Multiple Sclerosis
Molloy	2013	16744	74	doxycycline	Alzheimers Disease
Molloy	2013	16745	74	rifampin	Alzheimers Disease
Monge	1997	12856	20080	madopar	Parkinsons Disease
Montalban	2011	16746	2342	fingolimod	Multiple Sclerosis
Montastruc	1989	12991	15410	bromocript	Parkinsons Disease
Montastruc	1990	13195	15387	bromocript	Parkinsons Disease
Montastruc	1994	12986	15188	bromocript	Parkinsons Disease

For peer review only

Montastruc	1994	13196	15433	bromocript	Parkinsons Disease
Moore	1987	12017	3197	tetrahydrol	Parkinsons Disease
Moreau	2013	16747	75	memantine	Parkinsons Disease
Morgan	2005	12317	1170	rivastigmin	Parkinsons Disease
Morich	1996	13051	19796	nimodipine	Alzheimers Disease
Morillas-Ru	2010	13033	18891	polyphenol	Alzheimers Disease
Morita	2011	16886	2351	selegiline	Parkinsons Disease
Morita	2011	16887	2351	trihexypher	Parkinsons Disease
Morita	2011	16888	2351	amantadine	Parkinsons Disease
Morita	2011	16889	2351	entacapone	Parkinsons Disease
Morita	2011	16885	2351	droxidopa	Parkinsons Disease
Morrison	2013	16748	103	lithium	MND
Morrison	2013	16913	6987	lithium	MND
Morrow	2009	12478	8580	l-amphetan	Multiple Sclerosis
Morrow	2013	16749	2358	lisdexamfet	Multiple Sclerosis
Morselli	2007	12560	7782	riluzole	MND
Mosayebi	2011	19032	1575	vitamin d3	Multiple Sclerosis
Moss	1999	12229	6177	msf	Alzheimers Disease
Moss	2013	16750	76	msf	Alzheimers Disease
Mostert	2006	12546	9079	fluoxetine	Multiple Sclerosis
Mostert	2008	12489	8730	fluoxetine	Multiple Sclerosis
Mueller	1997	13308	10246	gabapentin	Multiple Sclerosis
Muller	2003	12040	1646	coenzyme c	Parkinsons Disease
Muller	2003	13109	22061	levodopa/c	Parkinsons Disease
Muller	2005	12023	1316	budipine	Parkinsons Disease
Muller	2006	11720	720	donepezil	Parkinsons Disease
Muller	2010	12314	81	benserazide	Parkinsons Disease
Muller-Vah	1999	11846	7504	nabilone	Huntingtons Disease
Mulnard	2000	12211	6039	estrogen	Alzheimers Disease
Munro	2006	13143	23411	sertraline	Alzheimers Disease
Munro	2012	16751	248	sertraline	Alzheimers Disease
Munsat	1981	12556	8273	amantadine	MND
Munsat	1997	16896	1478	riluzole	MND
Murialdo	2000	12593	6003	dexametha	Alzheimers Disease
Murphy	1995	16960	1589	igf-1	MND
Murray	1985	13344	10759	amantadine	Multiple Sclerosis
Myers	1992	12978	28254	chlorambuc	Multiple Sclerosis
Myers	1998	12473	10113	pentoxifylli	Multiple Sclerosis
Myllyla	1997	12858	20084	tolcapone	Parkinsons Disease
Na	2011	12879	19057	donepezil	Alzheimers Disease
Nabavi	2012	19080	1626	vitamin d3	Multiple Sclerosis
Nagels	2004	13197	28257	oxybutynin	Multiple Sclerosis
Nagels	2004	13199	28257	tolterodine	Multiple Sclerosis
Nakanishi	1992	13069	15296	bromocript	Parkinsons Disease
Napreyeyei	2007	12298	4569	ginkgo bilol	Alzheimers Disease
Narabayash	1995	13049	19644	lazabemide	Parkinsons Disease
Naseri	2009	12943	25731	ms14	Multiple Sclerosis
Nathan	2013	16916	117	gsk239512	Alzheimers Disease
Neu	1994	13257	19506	sulfasalazin	Multiple Sclerosis
Newall	1996	11885	8136	metoprolol	MND

For peer review only

Newall	1996	11886	8136	propranolo	MND
Newman	1985	12451	7613	emd 23,448	Huntingtons Disease
Nicoletti	2007	12413	621	estrogen/m	Parkinsons Disease
Niebroj-Dol	2002	11937	7950	riluzole	MND
Niepel	2013	16753	230	modafinil	Multiple Sclerosis
Nikolova	2001	13273	21112	donepezil	Alzheimers Disease
NINDS NET-	2007	11730	788	coenzyme q	Parkinsons Disease
NINDS NET-	2007	11731	788	gpi-1485	Parkinsons Disease
NINDS NET-	2006	11990	1028	creatine	Parkinsons Disease
NINDS NET-	2006	11991	1028	minocycline	Parkinsons Disease
Nolan	1991	12127	6969	thiamine	Alzheimers Disease
Nordberg	1993	12967	11076	tacrine	Alzheimers Disease
Norris	1974	12709	8279	guanidine	MND
Norris	1974	12711	8279	guanidine	MND
Norris	1986	11963	8249	octacosano	MND
Norris	1993	11969	8207	physostigm	MND
Norris	1987	13201	28511	ciclosporin	Multiple Sclerosis
Norris	1967	13381	3788	methixene	Parkinsons Disease
Noseworth	1997	12979	28528	sulfasalazin	Multiple Sclerosis
Noseworth	2000	12653	15367	linomide	Multiple Sclerosis
Nunes	2013	16819	188	lithium	Alzheimers Disease
Nutt	1978	11894	7639	naltrexone	Huntingtons Disease
Nutt	1979	12604	3360	valproate	Parkinsons Disease
O'Connor	2011	16754	2400	teriflunomi	Multiple Sclerosis
Ogawa	1985	12591	3257	l-threo-3,4-	Parkinsons Disease
Okahara	2010	12226	3930	yokukansar	Alzheimers Disease
Okahara	2010	12227	3930	yokukansar	Alzheimers Disease
Okereke	2004	12311	1342	donepezil	Parkinsons Disease
Olanow	1989	12438	3070	cv 205-502	Parkinsons Disease
Olanow	2004	12421	1405	sarizotan	Parkinsons Disease
Olanow	2006	11735	810	tch346	Parkinsons Disease
Olanow	2008	12914	13386	rasagiline	Parkinsons Disease
Olarte	1982	12574	8271	levamisole	MND
Olarte	1985	12571	8259	levamisole	MND
Olin	2010	11493	230	memantine	Alzheimers Disease
Olson	1978	11961	8277	tilorone	MND
Olson	1997	13203	28572	gabapentin	Multiple Sclerosis
Ondo	2002	11832	7442	tetrabenazi	Huntingtons Disease
Ondo	2007	11088	7334	memantine	Huntingtons Disease
Ondo	2010	12906	13078	parcopa	Parkinsons Disease
Ondo	2010	12908	13078	levodopa/c	Parkinsons Disease
Ondo	2011	12900	13054	memantine	Parkinsons Disease
Ondo	2011	16878	1056	selegiline	Parkinsons Disease
O'Riordan	1994	12984	28559	indoramin	Multiple Sclerosis
O'Suilleabh	2003	11826	7426	amantadine	Huntingtons Disease
Ott	2007	11111	4566	memantine	Alzheimers Disease
Overs	2012	16839	279	dalfampridi	Multiple Sclerosis
Paci	2001	12312	1906	amantadine	Parkinsons Disease
Paillisse	2005	12264	7868	riluzole	MND
Pakkenberg	1976	13206	15600	levodopa/b	Parkinsons Disease

For peer review only

Pakkenberg	1976	13211	15600	levodopa/c	Parkinsons Disease
Paleacu	2002	11835	7456	olanzapine	Huntingtons Disease
Palma	2000	16977	2563	gabapentin	MND
Pan	2011	16759	1036	tcm	Parkinsons Disease
Pane	2008	11850	7762	salbutamol	MND
Panitch	2006	12513	9083	dextrometh	Multiple Sclerosis
Papavasilio	1973	12002	3578	oxybate so	Parkinsons Disease
Park	1977	12019	3424	nomifensin	Parkinsons Disease
Parkes	1970	12027	3730	amantadine	Parkinsons Disease
Parkes	1970	12269	3747	amantadine	Parkinsons Disease
Parkes	1971	13021	15696	amantadine	Parkinsons Disease
Parkes	1975	13008	15620	levoamphe	Parkinsons Disease
Parkes	1975	13009	15620	dextroampl	Parkinsons Disease
Parkinson S	1993	12013	2892	selegiline	Parkinsons Disease
Parkinson S	1993	12014	2892	vitamin e	Parkinsons Disease
Parkinson S	1993	12075	2881	lazabemide	Parkinsons Disease
Parkinson S	1994	12011	2834	lazabemide	Parkinsons Disease
Parkinson S	1995	12072	2692	hp-200	Parkinsons Disease
Parkinson S	1995	12395	2962	hp-200	Parkinsons Disease
Parkinson S	1996	12338	2671	selegiline	Parkinsons Disease
Parkinson S	1996	12432	2575	lazabemide	Parkinsons Disease
Parkinson S	1998	12428	2390	selegiline	Parkinsons Disease
Parkinson S	1998	12431	2390	vitamin e	Parkinsons Disease
Parkinson S	2000	12065	2093	remacemid	Parkinsons Disease
Parkinson S	2001	12055	1881	remacemid	Parkinsons Disease
Parkinson S	2004	12030	1498	cep-1347	Parkinsons Disease
Parkinson S	2006	11992	1031	altinicine (s	Parkinsons Disease
Parnetti	1993	12763	6854	l-alpha glyc	Alzheimers Disease
Parry	2010	11945	7720	ursodeoxyc	MND
Pascuzzi	2010	11944	7719	talampanel	MND
Pashenkov	1997	13205	28616	lycopid	Multiple Sclerosis
Pasqualetti	2009	12565	4094	ibuprofen	Alzheimers Disease
Passeri	1985	12087	7157	minaprine	Alzheimers Disease
Pattee	2003	12267	7920	pramipexol	MND
Paty	1978	13207	28627	linoleic acic	Multiple Sclerosis
Paty	1983	12515	10799	linoleic acic	Multiple Sclerosis
Patzold	1980	12525	10841	azathioprin	Multiple Sclerosis
Patzold	1980	12527	10841	azathioprin	Multiple Sclerosis
Patzold	1982	12521	10821	azathioprin	Multiple Sclerosis
Patzold	1982	12524	10821	azathioprin	Multiple Sclerosis
Pendefunda	1975	13891	3504	levodopa/a	Parkinsons Disease
Percy	1971	16969	13088	isoprinosin	MND
Perry	2003	12134	5443	salvia	Alzheimers Disease
Perry	1979	12592	7636	isoniazid	Huntingtons Disease
Perry	1980	12587	7632	aminooxya	Huntingtons Disease
Perry	1982	12584	7624	isoniazid	Huntingtons Disease
Perryman	1991	12415	6953	tha + lecith	Alzheimers Disease
Peskind	2006	11115	4711	memantine	Alzheimers Disease
petro	2001	13208	28656	cannabidiol	Multiple Sclerosis
Pettegrew	1995	12169	6737	acetyl-l-car	Alzheimers Disease

For peer review only

Peyser	1995	12369	7553	vitamin e	Huntingtons Disease
Pezzoli	1991	13044	19203	pergolide	Parkinsons Disease
Pfeiffer	1991	12437	2989	cqa 206-29	Parkinsons Disease
Piccinin	1977	12727	7660	sodium dip	Huntingtons Disease
Piccinin	1977	12728	7660	fluphenazir	Huntingtons Disease
Piccirilli	1985	12731	3263	bornaprine	Parkinsons Disease
Piepers	2008	16906	3808	valproate	MND
Piepers	2009	11828	7728	valproate	MND
Pierlovisi-La	1991	12444	6940	piracetam	Alzheimers Disease
Pierrot-Des	2012	19033	1829	vitamin d3	Multiple Sclerosis
Pilipovich	2005	12730	1218	piribedil	Parkinsons Disease
Pilipovich	2006	13170	13829	piribedil	Parkinsons Disease
Pilling	1975	12000	3500	amantadine	Parkinsons Disease
Piolti	1991	12710	2984	proglumide	Parkinsons Disease
Pioro	2010	11943	7696	dextrometh	MND
Plaitakis	1988	11806	8239	l-leucine, is	MND
Plaut	1987	12512	10713	amantadine	Multiple Sclerosis
Poewe	2006	11993	1106	rivastigmin	Parkinsons Disease
Poewe	2011	16761	841	pramipexol	Parkinsons Disease
Pollock	1972	12686	3617	amantadine	Parkinsons Disease
Polman	1994	12506	10450	4-aminopyr	Multiple Sclerosis
Polman	1994	12508	10450	3,4-diamino	Multiple Sclerosis
Polman	1994	12852	19510	4-aminopyr	Multiple Sclerosis
Pomara	1983	12969	11092	hydergine	Alzheimers Disease
Pomara	1985	12086	7162	naltrexone	Alzheimers Disease
Pomara	2002	12188	5677	mifepristor	Alzheimers Disease
Pomara	2006	12159	4740	mifepristor	Alzheimers Disease
Pomara	2006	12160	4740	dexametha	Alzheimers Disease
Pomara	2006	12162	4740	mifepristor	Alzheimers Disease
Pomara	2007	11101	4571	memantine	Alzheimers Disease
Pomara	1983	12970	11092	hydergine/l	Alzheimers Disease
Pompidou	1986	13183	27940	ciclosporin	Multiple Sclerosis
Pompidou	1986	13234	16025	isoprinosine	Multiple Sclerosis
Pondal	1996	12010	2570	trihexypher	Parkinsons Disease
Pongratz	2000	11906	8013	riluzole	MND
Pontieri	2005	11908	7860	minocycline	MND
Pontieri	2005	12341	7860	riluzole	MND
Popova	2010	13244	18864	piracetam/	Multiple Sclerosis
Popova	2011	16953	1154	emhps	Multiple Sclerosis
Porsteinsso	2008	12569	4386	memantine	Alzheimers Disease
Postuma	2006	12304	924	entacapone	Parkinsons Disease
Postuma	2006	12309	924	vitamin b a	Parkinsons Disease
Postuma	2012	16762	346	caffeine	Parkinsons Disease
Potkin	2002	12292	5616	neotrofin	Alzheimers Disease
Pourcher	1992	12342	2949	ethosuximi	Parkinsons Disease
Pourcher	2012	16763	2474	istradefyllir	Parkinsons Disease
Prentice	1996	13050	19771	tacrine	Alzheimers Disease
Prineas	1967	13385	10959	clofibrate	Multiple Sclerosis
Przuntek	1987	13185	27948	azathioprin	Multiple Sclerosis
Przuntek	2002	12048	1795	budipine	Parkinsons Disease

For peer review only

Puntoni	1983	13248	19093	biperiden	Parkinsons Disease
Puri	2002	11836	7459	ethyl-epa	Huntingtons Disease
Puri	2005	11815	7373	ethyl-epa	Huntingtons Disease
Puri	2008	11793	7276	ethyl-epa	Huntingtons Disease
Putzki	2002	12043	1766	flupirtine	Parkinsons Disease
Qin	2002	13102	21582	levodopa/b	Parkinsons Disease
Qin	2002	13103	21582	levodopa/p	Parkinsons Disease
Quinn	1984	11880	7614	sulpiride	Huntingtons Disease
Quinn	2010	12222	3820	docosahexa	Alzheimers Disease
Quinn	2003	12396	5369	vitamin c	Alzheimers Disease
Rabkin	2009	11825	7745	modafinil	MND
Radue	2012	16764	295	fingolimod	Multiple Sclerosis
Raffi	2011	13247	19041	huperzine-a	Alzheimers Disease
Rai	1989	12971	11096	acetyl-l-car	Alzheimers Disease
Rai	1990	12416	7033	acetyl-l-car	Alzheimers Disease
Rainer	2011	16890	2487	memantine	Alzheimers Disease
Rainer	2013	16853	2487	memantine	Alzheimers Disease
Rajput	1997	12329	2431	tolcapone	Parkinsons Disease
Rammohan	2002	12456	9748	modafinil	Multiple Sclerosis
Rampello	2002	12262	1838	citalopram	Parkinsons Disease
Randolph	1994	12397	6824	cycloserine	Alzheimers Disease
Randot	1987	13200	15493	levodopa/b	Parkinsons Disease
Ranen	1996	11857	7546	idebenone	Parkinsons Disease
Rao	1971	13357	3690	amantadine	Parkinsons Disease
Rascol	1990	12863	15358	cqa 206-29	Parkinsons Disease
Rascol	1994	12339	2822	naltrexone	Parkinsons Disease
Rascol	2001	12056	1911	idazoxan	Parkinsons Disease
Rascol	2011	16883	948	rasagiline	Parkinsons Disease
Rascol	2012	16766	352	naftazone	Parkinsons Disease
Rascol	2012	16778	680	perampane	Parkinsons Disease
Ravina	2005	11998	1184	donepezil	Parkinsons Disease
Reading	2001	12054	1865	rivastigmin	Parkinsons Disease
Reardon	2000	12063	2080	perindopril	Parkinsons Disease
Regland	2001	12548	5784	clioquinol	Alzheimers Disease
Reichmann	2003	12875	22058	pramipexol	Parkinsons Disease
Reilly	1983	12594	3295	ascorbic aci	Parkinsons Disease
Reines	2004	12117	5324	rofecoxib	Alzheimers Disease
Reisberg	2003	11102	5514	memantine	Alzheimers Disease
Reisberg	2006	11107	4836	memantine	Alzheimers Disease
Rektorova	2004	13116	22381	donepezil	Alzheimers Disease
Rektorova	2004	13288	22381	donepezil	Parkinsons Disease
Remington	2009	12440	4190	vitamin/nu	Alzheimers Disease
Remington	2009	12769	4190	nutritional	Alzheimers Disease
Ren	2008	12318	326	benserazide	Parkinsons Disease
Reynolds	2007	11797	7332	minocycline	Huntingtons Disease
Rice	1998	13209	28739	cladribine	Multiple Sclerosis
Rieckmann	1995	12993	28752	pentoxifylli	Multiple Sclerosis
Riekkinen	1999	12071	2163	clonidine	Parkinsons Disease
Riekkinen	1999	12942	20602	clonidine	Parkinsons Disease
Riepe	1997	11892	8115	riluzole	MND

For peer review only

Riepe	2007	11497	4560	memantine	Alzheimers Disease
Rigaud	2003	12155	5563	hrt	Alzheimers Disease
Rigaud	2005	13152	11712	rivastigmin	Alzheimers Disease
Rinne	1972	13894	3642	amantadin	Parkinsons Disease
Rinne	1980	13213	28763	tizanidine	Multiple Sclerosis
Riopelle	1988	13073	15448	levodopa/b	Parkinsons Disease
Risner	2006	12164	4818	rosiglitazon	Alzheimers Disease
Ritchie	2003	12120	5355	clioquinol	Alzheimers Disease
Rive	2004	11504	5254	memantine	Alzheimers Disease
Riviere	1998	16907	4121	riluzole	MND
Rix	1977	12612	3421	elantrine	Parkinsons Disease
Rocca	2002	13101	21553	donepezil	Alzheimers Disease
Roch-Torre	2000	13383	8020	riluzole	MND
Rockwood	1997	12240	6445	linopirdine	Alzheimers Disease
Rogers	1993	13360	6841	indometha	Alzheimers Disease
Rohrbach	1988	12996	28783	triamcinolo	Multiple Sclerosis
Rosas	1999	11848	7511	riluzole	Huntingtons Disease
Rose	1979	13000	28792	azathioprin	Multiple Sclerosis
Rosenberg	1988	12526	10649	amantadin	Multiple Sclerosis
Rosenberg	2013	16768	2516	methylphe	Alzheimers Disease
Rosenfeld	2000	12268	7995	oxandrolon	MND
Rosenfeld	2008	11847	7760	creatine m	MND
Rosler	1998	12854	20464	rivastigmin	Alzheimers Disease
Rossini	1996	13238	29374	4-aminopyr	Multiple Sclerosis
Rossini	2001	12455	9743	4-aminopyr	Multiple Sclerosis
Rowan	2007	12319	789	donepezil	Parkinsons Disease
Rudge	1989	13029	16143	ciclosporin	Multiple Sclerosis
Rudge	1989	13081	28816	ciclosporin	Multiple Sclerosis
Rudnicki	2013	16849	190	dexpramipe	MND
Ruggieri	1976	13389	3432	cytidine-5-c	Parkinsons Disease
Russman	2003	11916	7911	riluzole	MND
Sabbagh	2011	16769	2527	pf-0449470	Alzheimers Disease
Safarinejad	2009	13083	28842	omega 3	Parkinsons Disease
Saft	2006	12303	7350	valproate	Huntingtons Disease
Saida	1998	13214	28846	mizoribine	Multiple Sclerosis
Saida	2012	16770	2533	fingolimod	Multiple Sclerosis
Sailer	1996	13215	28848	amantadin	Multiple Sclerosis
Sailer	2000	12698	9964	amantadin	Multiple Sclerosis
Sakurai	2013	16771	187	cilostazol	Alzheimers Disease
Salas-Salva	2005	12336	5010	vegenat (ly	Alzheimers Disease
Saletu	1995	12170	6720	nicergoline	Alzheimers Disease
Salloway	2011	16772	806	elnd005	Alzheimers Disease
Samorajski	1985	12080	7167	lecithin & p	Alzheimers Disease
Sampaio	2011	13133	26682	pardoprun	Parkinsons Disease
Sancesario	1984	12439	3270	bornaprine	Parkinsons Disease
Sandler	1972	12284	3607	metatyrosi	Parkinsons Disease
Sandyk	1981	12601	3330	carbidopa	Parkinsons Disease
Sandyk	1981	12602	3330	amantadin	Parkinsons Disease
Sano	1990	12079	3043	piracetam	Parkinsons Disease
Sano	1992	12147	6889	acetyl levo	Alzheimers Disease

For peer review only

Sano	1997	12241	6450	selegiline	Alzheimers Disease
Sano	1997	12243	6450	vitamin e	Alzheimers Disease
Sano	1997	13307	6450	selegiline/c	Alzheimers Disease
Sano	2011	16774	851	simvastatin	Alzheimers Disease
Sarycheva	2011	13162	13070	ladasten	Parkinsons Disease
Sato	2002	12045	1776	menatetrer	Parkinsons Disease
Sato	2006	11882	7832	etidronate	MND
Sato	2006	11988	1016	alendronat	Parkinsons Disease
Sato	2006	11989	1016	vitamin d2	Parkinsons Disease
Sato	1999	12005	2274	vitamin d3	Parkinsons Disease
Sato	2011	16880	733	risedronate	Parkinsons Disease
sato	2007	12168	4723	tandospiroı	Alzheimers Disease
Savery	1976	13356	3452	levodopa/c	Parkinsons Disease
Savery	1977	12607	3410	amantadin	Parkinsons Disease
Sawada	2010	12905	13077	amantadin	Parkinsons Disease
Saxton	2012	16775	681	memantine	Alzheimers Disease
Scelsa	2005	16931	6234	indinavir	MND
Schapira	2013	16776	158	safinamide	Parkinsons Disease
Schapira	2013	16779	197	pramipexol	Parkinsons Disease
Schapira	2013	16780	198	pramipexol	Parkinsons Disease
Schapira	2013	16781	2563	pramipexol	Parkinsons Disease
Scharf	1999	12219	6123	diclofenac ,	Alzheimers Disease
Schellenbei	1997	12245	6504	cyclandelat	Alzheimers Disease
Scheltens	2012	16782	652	souvenaid	Alzheimers Disease
Schlegel	1989	12109	7054	guanfacine	Alzheimers Disease
Schluep	1991	13253	19207	ciclosporin	Multiple Sclerosis
Schluep	1991	13254	19207	azathioprin	Multiple Sclerosis
Schmidt	2008	11100	4292	memantine	Alzheimers Disease
Schmidt	2010	12736	3854	memantine	Alzheimers Disease
Schmidt	1975	13216	28871	dantrolene	Multiple Sclerosis
Schmidt	1975	13217	28871	diazepam	Multiple Sclerosis
Schmitt	2006	11098	4626	memantine	Alzheimers Disease
Schmitt	2010	12876	18963	rivastigmin	Parkinsons Disease
Schneider	2005	12579	4850	ginkgo bilol	Alzheimers Disease
Schneider	1997	12302	6401	tacrine & e:	Alzheimers Disease
Schneider	1997	12615	6403	estrogen	Alzheimers Disease
Schneider	2011	12393	3814	cholinester	Alzheimers Disease
Schneider	2011	12394	3814	memantine	Alzheimers Disease
Schneider	1982	13001	15543	bromocript	Parkinsons Disease
Schneider	1984	12732	3272	memantine	Parkinsons Disease
Schubert	1975	12758	3495	amantadin	Parkinsons Disease
Schultz	1984	13908	28877	cranberry ji	Multiple Sclerosis
Schulz	2011	13154	26633	memantine	Alzheimers Disease
Schwab	1969	12699	3773	amantadin	Parkinsons Disease
Schwartz	1996	12362	6603	cycloserine	Alzheimers Disease
Schwartz	1973	12681	3594	mk-486	Parkinsons Disease
Schwarz	1992	12588	2938	folinic acid	Parkinsons Disease
Scigliano	1984	12580	7619	gamma-vin	Huntingtons Disease
SCLEROSIS .	1988	12557	10655	azathioprin	Multiple Sclerosis
SCLEROSIS .	1988	13090	29079	azathioprin	Multiple Sclerosis

For peer review only

Sclesa	2005	11909	7876	indinavir	MND
Scott	1997	13218	28895	gabapentin	Multiple Sclerosis
Scripnikov	2007	12174	4478	ginkgo bilol	Alzheimers Disease
Selmaj	2013	16861	2583	siponimod	Multiple Sclerosis
Senin	1991	12135	6939	aniracetam	Alzheimers Disease
Senin	1992	12149	6888	idebenone	Alzheimers Disease
Seppi	2001	11838	7466	riluzole	Huntingtons Disease
Serby	1986	12093	7137	naltrexone	Alzheimers Disease
Serrano-Du	2003	13112	22118	clonidine	Parkinsons Disease
Sevigny	2008	12194	4199	mk-677	Alzheimers Disease
Sharafaddir	2010	12460	8351	naltrexone	Multiple Sclerosis
Shaw	1973	12537	3585	melatonin	Parkinsons Disease
Shaw	1976	13921	3458	melatonin	Parkinsons Disease
Shaw	1978	13220	28925	fatty acids	Multiple Sclerosis
Shaygannej	2007	12923	28927	rivastigmin	Multiple Sclerosis
Shaygannej	2010	12570	8390	glucosamin	Multiple Sclerosis
Shaygannej	2012	16858	274	aspirin	Multiple Sclerosis
Shaygannej	2012	16859	274	amantadin	Multiple Sclerosis
Shaygannej	2012	19150	2554	calcitriol	Multiple Sclerosis
Shefner	2004	11912	7889	creatine	MND
Shefner	2012	16785	331	ck-017357	MND
Sheplan	1975	13084	28929	dantrolene	Multiple Sclerosis
Shinotoh	1997	11082	2480	lamotrigine	Parkinsons Disease
Shinto	2009	12480	8597	omega 3 fa	Multiple Sclerosis
Shiozawa	1981	13393	3331	amantadin	Parkinsons Disease
Shotbolt	2009	12956	25441	quetiapine	Parkinsons Disease
Shoulson	1975	12613	7667	imidazole 4	Huntingtons Disease
Shoulson	1976	12621	7666	dipropylace	Huntingtons Disease
Shoulson	1978	11895	7643	muscimol	Huntingtons Disease
Shoulson	1989	11870	7599	baclofen	Huntingtons Disease
Shoulson	1992	13076	19225	selegiline	Parkinsons Disease
Shoulson	1992	13079	19225	tocopherol	Parkinsons Disease
Shoulson	2001	12059	1971	remacemid	Parkinsons Disease
Shrotriya	1996	12629	6525	bmy 21,50	Alzheimers Disease
Shults	2002	12041	1735	coenzyme c	Parkinsons Disease
Shults	2002	12042	1735	vitamin e	Parkinsons Disease
Shults	2004	12316	1408	coenzyme c	Parkinsons Disease
Shults	1986	12628	7608	cystamine	Huntingtons Disease
Siemers	2006	12165	4804	ly450139 di	Alzheimers Disease
Signoret	1978	12064	7206	choline citr	Alzheimers Disease
Sigwald	1970	12762	3749	amantadin	Parkinsons Disease
Sigwald	1972	12752	3605	amantadin	Parkinsons Disease
Silberberg	1965	12782	10968	tranylcypro	Multiple Sclerosis
Silver	1972	12687	3618	amantadin	Parkinsons Disease
Silver	2007	11128	8857	lamotrigine	Multiple Sclerosis
Silver	1971	12693	3695	amantadin	Parkinsons Disease
Simon	2008	12364	15452	caffeine	Parkinsons Disease
Simons	1996	12332	2561	fluoxetine	Parkinsons Disease
Simons	2002	12163	5625	simvastatin	Alzheimers Disease
Simpson	1985	13221	28956	efamol	Multiple Sclerosis

For peer review only

Simuni	2010	13906	13079	isradipine	Parkinsons Disease
Singer	2003	12124	5360	melatonin	Alzheimers Disease
Sipe	1994	13163	26862	cladribine	Multiple Sclerosis
Sivori	2007	12256	7786	riluzole	MND
Sjogren	2003	12142	5506	simvastatin	Alzheimers Disease
Smith	1984	12638	7175	lecithin	Alzheimers Disease
Smith	1984	12685	7175	lecithin & p	Alzheimers Disease
Smith	1994	13085	28968	tizanidine	Multiple Sclerosis
Smith	1986	16928	2634	bromocript	MND
Smolders	2010	19025	2138	vitamin d3	Multiple Sclerosis
Smolders	2011	16844	710	vitamin d3	Multiple Sclerosis
Smythies	1984	12448	3265	l-methionir	Parkinsons Disease
Snider	1984	12381	3264	octacosano	Parkinsons Disease
Snow	2000	12259	2078	amantadine	Parkinsons Disease
Snow	2010	11525	62	mitoq	Parkinsons Disease
Sofuoglu	2008	16904	3490	riluzole	MND
Sofuoglu	2008	16905	3490	d-ampheta	MND
Soilu-Hanni	2012	16850	451	vitamin d3	Multiple Sclerosis
Soininen	1985	12636	7150	org 2766	Alzheimers Disease
Soininen	2007	12176	4647	celecoxib	Alzheimers Disease
Sojka	1997	12272	8120	riluzole	MND
Solaro	1999	13223	28992	gabapentin	Multiple Sclerosis
Solaro	1999	13224	28992	vigabatrin	Multiple Sclerosis
Solaro	2000	12467	9921	gabapentin	Multiple Sclerosis
Solaro	2007	12496	8882	oxcarbazep	Multiple Sclerosis
Sommer	1999	11976	8040	riluzole	MND
Sommer	2003	13279	22156	tacrine	Huntingtons Disease
Soraru	2006	12263	7809	riluzole / cl	MND
Sorensen	1995	13225	28995	azathioprin	Multiple Sclerosis
Sorensen	2011	16843	2633	simvastatin	Multiple Sclerosis
Sotirchos	2013	19026	2157	vitamin d3	Multiple Sclerosis
Sourander	1987	12097	7119	aniracetam	Alzheimers Disease
Soysal	2008	12313	487	antiparkins	Parkinsons Disease
Spagnoli	1991	13362	6943	acetyl-l-car	Alzheimers Disease
Sparks	1998	12266	4708	atorvastati	Alzheimers Disease
Sparks	2005	12145	5019	atorvastati	Alzheimers Disease
Sparks	2005	12573	4987	atorvastati	Alzheimers Disease
Spieker	1999	12085	2199	budipine	Parkinsons Disease
Spieker	1999	12310	2205	budipine	Parkinsons Disease
Squitieri	2008	12871	24921	riluzole	Huntingtons Disease
Squitieri	2009	11932	7262	riluzole	Huntingtons Disease
Squitti	2002	12189	5734	penicillami	Alzheimers Disease
Sramek	1995	12406	6672	xanomeline	Alzheimers Disease
Sramek	1995	12541	6740	ci-979 (mila	Alzheimers Disease
Sramek	1996	12249	6638	rivastigmin	Alzheimers Disease
Sramek	1998	12235	6354	alvameline	Alzheimers Disease
Sramek	1999	12228	6168	nxx-066	Alzheimers Disease
Stacy	2008	11676	505	istradefyllir	Parkinsons Disease
Stahl	2004	12889	1331	galantamin	Alzheimers Disease
Stankoff	2005	12469	9276	modafinil	Multiple Sclerosis

For peer review only

Stathis	2011	12901	13059	levetiraceta	Parkinsons Disease
Steffensen	2011	16845	814	vitamin d3/	Multiple Sclerosis
Stefoski	1991	13366	10563	4-aminopyr	Multiple Sclerosis
Stein	2011	16790	779	vitamin d2	Multiple Sclerosis
Stelmasiak	1997	13226	29032	cladribine	Multiple Sclerosis
Stocchi	1989	11866	7594	terguride	Huntingtons Disease
Stocchi	2011	16792	915	ropinirole	Parkinsons Disease
Stocchi	2011	16869	728	rasagiline	Parkinsons Disease
Stocchi	2011	16870	728	rasagiline	Parkinsons Disease
Stocchi	2012	16791	2649	safinamide	Parkinsons Disease
Stommel	2009	11948	7722	thalidomide	MND
Stone	1989	13228	29037	ciclosporin	Multiple Sclerosis
Storch	2007	12544	710	coenzyme q	Parkinsons Disease
Strang	1965	12701	3808	ponalid	Parkinsons Disease
Strang	1966	12028	3790	ucb 1549	Parkinsons Disease
Strang	1966	12702	3804	rigidyl	Parkinsons Disease
Strang	1966	12703	3795	lysivane	Parkinsons Disease
Strang	1966	12704	3789	sd25	Parkinsons Disease
Strang	1966	12755	3793	ucb 1549	Parkinsons Disease
Strang	1967	12359	3783	caffeine/ch	Parkinsons Disease
Strang	1967	12700	3785	biperiden	Parkinsons Disease
Streim	2008	12357	4289	aripiprazole	Alzheimers Disease
Striano	2006	13126	23886	levetiraceta	Multiple Sclerosis
Strijks	1997	12067	2528	coenzyme q	Parkinsons Disease
Strijks	1999	12257	2230	estradiol	Parkinsons Disease
Strijks	1999	12258	2230	progesteron	Parkinsons Disease
Su	2004	13283	22528	selegiline	Parkinsons Disease
Sudilovsky	1993	12136	6869	ceranapril	Alzheimers Disease
Suh	2008	12940	24575	galantamin	Alzheimers Disease
Summers	1986	12977	12983	tetrahydroa	Alzheimers Disease
Sumowski	2011	16876	2661	l-amphetan	Multiple Sclerosis
Sun	2007	12346	4431	donepezil +	Alzheimers Disease
Sun	1988	13239	29376	tripterygiur	Multiple Sclerosis
Suzuki	2013	16793	63	vitamin d	Parkinsons Disease
Suzumura	2000	12470	9969	vinpocetin ,	Multiple Sclerosis
Swinburn	1974	13087	29057	azathioprin	Multiple Sclerosis
Swoboda	2009	11813	7740	valproate	MND
Swoboda	2010	11809	7699	l-carnitine/	MND
Symington	1978	12375	7649	valproate	Huntingtons Disease
Szczepanik	1988	12742	8070	pimozide	MND
Szulc-Kuber	1990	11965	8234	bromocript	MND
Tabrizi	2005	11816	7378	creatine	Huntingtons Disease
Tabrizi	2003	11827	7428	creatine	Huntingtons Disease
Tan	1998	13089	29062	linomide	Multiple Sclerosis
Tan	1998	13229	29064	linomide	Multiple Sclerosis
Tan	1976	12307	7665	valproate	Huntingtons Disease
Tandan	1996	11887	8129	l-leucine, is	MND
Tandan	1996	11888	8129	l-threonine	MND
Tariot	1988	12102	7094	tranylcypro	Alzheimers Disease
Tariot	2004	11104	5317	memantine	Alzheimers Disease

For peer review only

Tariot	2012	16864	634	donepezil	Alzheimers Disease
Tariot	2011	16794	2670	valproate	Alzheimers Disease
Tavakoli	1999	16927	2432	riluzole	MND
Tekin	1998	11081	6285	lamotrigine	Alzheimers Disease
Tell	1981	12586	7630	gamma-vin	Huntingtons Disease
Teri	1991	12445	6944	imipramine	Alzheimers Disease
Testa	1989	16970	1638	amino acid	MND
Testa	1992	11840	8222	l-threonine	MND
Testa	1992	11842	8222	vitamin b a	MND
teychenne	1976	12020	3426	nomifensin	Parkinsons Disease
Thal	1996	12247	6553	acetyl-l-car	Alzheimers Disease
Thal	2000	12207	5923	acetyl-l-car	Alzheimers Disease
Thal	2003	12121	5359	idebenone	Alzheimers Disease
The Canadi	1987	13367	10699	amantadin	Multiple Sclerosis
The Huntin	1998	11851	7519	opc-14117	Huntingtons Disease
The Italian	1993	13364	8208	l-leucine, is	MND
The Multipl	1990	12781	10587	ciclosporin	Multiple Sclerosis
The Multipl	1988	13230	29085	ciclosporin	Multiple Sclerosis
The Parkins	2011	16862	719	estrogen	Parkinsons Disease
The Parkins	1994	13920	2806	tocopherol	Parkinsons Disease
The Parkins	2011	16718	1136	pramipexol	Parkinsons Disease
The Parkins	2007	11708	631	cep-1347	Parkinsons Disease
The UK Tiza	1994	13031	16301	tizanidine	Multiple Sclerosis
Thobois	2013	16814	61	piribedil	Parkinsons Disease
Thomas	2001	12202	5851	donepezil	Alzheimers Disease
Thomas	2001	12203	5851	rivastigmin	Alzheimers Disease
Thomas	2001	12204	5851	vitamin e	Alzheimers Disease
Thomas	2004	12032	1510	amantadin	Parkinsons Disease
Thomas	2010	12539	29	amantadin	Parkinsons Disease
Thomas	2004	11820	7408	minocyclin	Huntingtons Disease
Thomas	2005	12921	23027	donepezil	Parkinsons Disease
Thompson	1990	12119	7000	hydergine	Alzheimers Disease
Timberlake	1978	12382	3391	levodopa	Parkinsons Disease
Timberlake	1978	12387	3391	amantadin	Parkinsons Disease
Tison	2013	16915	91	simvastatin	Parkinsons Disease
Tohgi	1995	12936	19637	tolcapone/	Parkinsons Disease
Tohgi	1977	13394	3416	mk-486	Parkinsons Disease
Tolosa	2012	16795	2689	rasagiline	Parkinsons Disease
Torkildsen	2012	16851	2693	fatty acids	Multiple Sclerosis
Torun	2010	13153	26125	sertraline	Parkinsons Disease
Tourian	1972	12624	7684	5-hydroxy-l	Huntingtons Disease
Tourtellott	1982	12918	15540	gogentin &	Parkinsons Disease
Tourtellott	1982	12999	15540	benztropin	Parkinsons Disease
Trappler	1998	13268	20355	sertraline	Parkinsons Disease
Trappler	1998	13269	20355	fluoxetine	Parkinsons Disease
Trappler	1998	13270	20355	paroxetine	Parkinsons Disease
Traynor	2004	16901	3068	topiramate	MND
Traynor	2004	16974	3068	topiramate	MND
Trejo	2005	12305	7370	fresubin (2	Huntingtons Disease
Treves	1999	12403	6077	denbufyllin	Alzheimers Disease

For peer review only

Tribanek	2012	16709	491	ginkgo bilol	Alzheimers Disease
Trzepacz	2013	16797	2698	ly451395	Alzheimers Disease
Tsai	1998	12233	6299	cycloserine	Alzheimers Disease
Tsang	2000	12061	2047	estrogen	Parkinsons Disease
Tsubaki	1974	13395	3520	citicoline	Parkinsons Disease
Tsui	1989	13384	3095	cy208-243	Parkinsons Disease
Tweedy	1982	12597	3305	lecithin	Parkinsons Disease
Tyne	2010	11570	197	modafinil	Parkinsons Disease
Tzimopoulc	2010	13035	18962	rosiglitazon	Alzheimers Disease
Uitti	1997	13264	20086	amantadin	Parkinsons Disease
Uney	1992	12140	6912	lecithin	Alzheimers Disease
Utsumi	2013	16937	185	pramipexol	Parkinsons Disease
Vaddadi	2002	12368	7458	hufa therap	Huntingtons Disease
Valen-Send	2010	12347	3953	estradiol/n	Alzheimers Disease
Valiquette	1993	13094	29159	desmopres	Multiple Sclerosis
van Amerо	2012	19028	2324	vitamin d3	Multiple Sclerosis
van Diemer	1992	12780	10543	4-aminopyr	Multiple Sclerosis
van Donger	2000	12590	5913	gingko bilol	Alzheimers Disease
Van Dyck	1997	12238	6419	linopirdine	Alzheimers Disease
Van Dyck	2007	11108	4516	memantine	Alzheimers Disease
van Gool	2001	12250	5799	hydroxychl	Alzheimers Disease
Van Kan	2008	12328	7780	riluzole	MND
Van Puyvel	2011	16798	893	galantamin	Alzheimers Disease
van Vugt	1997	11853	7532	clozapine	Huntingtons Disease
Van Woert	1972	12684	3602	p-cpa (para	Parkinsons Disease
VanItallie	2005	12325	1264	hyperketog	Parkinsons Disease
Vellas	1998	13266	20246	rivastigmin	Alzheimers Disease
Vellas	2011	13161	26672	eht0202	Alzheimers Disease
Verbessem	2003	11824	7423	creatine	Huntingtons Disease
Verhagen	1998	12051	2356	dextromet	Parkinsons Disease
Verhagen N	2002	11834	7450	amantadin	Huntingtons Disease
Vermersch	1991	12987	15307	lisuride	Parkinsons Disease
Vermersch	2012	16800	2732	masitinib	Multiple Sclerosis
Veroff	1998	12230	6213	xanomeline	Alzheimers Disease
Verstraete	2012	16875	2733	lithium	MND
Vestergaar	1977	12618	7657	lithium	Huntingtons Disease
Viallet	2013	16821	218	rasagiline	Parkinsons Disease
Viallet	2013	16822	218	rasagiline	Parkinsons Disease
Vida	1989	12678	7051	lecithin/tet	Alzheimers Disease
Vida	1989	12680	7051	lecithin	Alzheimers Disease
Viglietta	2010	13909	29201	cladribine	Multiple Sclerosis
Villardita	1992	12654	6923	oxiracetam	Alzheimers Disease
Villoslada	2009	11091	8609	memantine	Parkinsons Disease
Vojinovic	2005	19079	2384	alfacalcidol	Multiple Sclerosis
Wailke	2011	12926	13048	levodopa/c	Parkinsons Disease
Wajgt	1999	13233	29230	cladrybine	Multiple Sclerosis
Waldemar	2008	11112	4398	memantine	Alzheimers Disease
Waldemar	2010	12896	19048	donepezil	Alzheimers Disease
Waldemar	2011	16801	1058	donepezil	Alzheimers Disease
Walker	1989	11868	7597	dextromet	Huntingtons Disease

For peer review only

Walker	1972	12021	3631	amantadine	Parkinsons Disease
Walker	1972	12689	3647	amantadine	Parkinsons Disease
Walker	1972	12934	15679	amantadine	Parkinsons Disease
Walker	2001	12458	9767	pirfenidone	Multiple Sclerosis
Walker	2005	12494	9290	pirfenidone	Multiple Sclerosis
Wallin	2009	12925	25366	donepezil	Alzheimers Disease
Wallin	2009	12929	25366	galantamin	Alzheimers Disease
Wandinger	1999	12077	2185	amantadine	Parkinsons Disease
Wang	2000	12209	5984	estrogen	Alzheimers Disease
Wang	2003	13277	21869	nilestrol/hu	Alzheimers Disease
Weber	2010	16908	4296	tetrahydroc	MND
Weeth	1969	13902	3764	levodopa/a	Parkinsons Disease
Weeth	1969	13903	3764	amantadine	Parkinsons Disease
Weilbach	1995	13242	16387	methotrexa	Multiple Sclerosis
Weiner	1993	13235	29268	myelin	Multiple Sclerosis
Weiner	2011	12836	26634	memantine	Alzheimers Disease
Weiner	2001	12947	21264	pramipexol	Parkinsons Disease
Weinshenk	1992	13030	10542	pemoline	Multiple Sclerosis
Weinstein	1991	12128	6964	lecithin/tet	Alzheimers Disease
Weintraub	1983	12078	7179	lecithin	Alzheimers Disease
Weiss	1994	12660	6811	phosphatid	Alzheimers Disease
Weiss	1994	12661	6811	pyritinol	Alzheimers Disease
Weiss	1987	12677	7113	estrogen/n	Alzheimers Disease
Wender	1987	13368	10702	prednisolon	Multiple Sclerosis
Wender	1991	13251	19206	prednisolon	Multiple Sclerosis
Wender	1991	13252	19206	prednisolon	Multiple Sclerosis
Wermuth	1998	12865	20361	pramipexol	Parkinsons Disease
Wermuth	1998	12939	20353	citalopram	Parkinsons Disease
Wesnes	2005	12321	1083	rivastigmin	Parkinsons Disease
Wesseling	1984	12620	7173	4-aminopyr	Alzheimers Disease
Weyer	1996	12883	11139	idebenone	Alzheimers Disease
Weyer	1997	12244	6496	idebenone	Alzheimers Disease
Weyer	2000	12705	5979	cyclandelat	Alzheimers Disease
Wilcock	1993	12834	19241	tacrine	Alzheimers Disease
Wilcock	2008	12195	4331	tarenflurbil	Alzheimers Disease
Wilkinson	2007	12904	24048	memantine	Alzheimers Disease
Wilkinson	2012	16802	2768	memantine	Alzheimers Disease
Winblad	1999	11117	5957	memantine	Alzheimers Disease
Winblad	2001	13095	21135	nicergoline	Alzheimers Disease
Winblad	2010	13036	18977	phenserine	Alzheimers Disease
Wingerchul	2005	12465	9274	aspirin	Multiple Sclerosis
Wingerchul	2005	19034	2464	calcitriol	Multiple Sclerosis
Wissel	2006	11877	7817	nabilone	upper motor neurone syndrome
Wolf	2010	11952	125	amantadine	Parkinsons Disease
Wolinsky	1990	13231	29086	ciclosporin	Multiple Sclerosis
Wolinsky	2000	13098	29313	linomide	Multiple Sclerosis
Wolkowitz	2003	12144	5510	dhea	Alzheimers Disease
Wolters	1996	13053	19829	olanzapine	Parkinsons Disease
Wolters	1996	13057	19868	levodopa/c	Parkinsons Disease
Wolters	1996	13056	19868	levodopa/c	Parkinsons Disease

For peer review only

Wolz	2010	12980	13088	levetiraceta	Parkinsons Disease
Wolz	2012	16805	272	chocolate	Parkinsons Disease
Wong	2011	13134	26683	levetiraceta	Parkinsons Disease
Wood	1994	12899	19299	tacrine	Alzheimers Disease
Wouters	2010	13147	11144	galantamin	Alzheimers Disease
Wu	2012	16935	314	pioglitazon	Alzheimers Disease
Wurster	1993	13100	29327	15+- dexox	Multiple Sclerosis
Xu	1995	12647	6665	huperzine-a	Alzheimers Disease
Xu	1999	12595	6006	huperzine-a	Alzheimers Disease
Yadav	2005	12491	9289	lipoic acid	Multiple Sclerosis
Yamagami	1991	12669	6974	bifemelane	Alzheimers Disease
Yanagisawa	1997	16964	2422	riluzole	MND
Yanagisawa	1998	13271	20376	l-threo-dihy	Parkinsons Disease
Yancheva	2009	12388	4113	ginkgo bilol	Alzheimers Disease
Yancheva	2009	12389	4113	ginkgo bilol	Alzheimers Disease
Yancheva	2009	12390	4113	donepezil/g	Alzheimers Disease
Yang	2002	13240	29378	di huang co	Multiple Sclerosis
Yang	2003	13276	21868	huperzine-a	Alzheimers Disease
yarosh	1996	13236	29336	pyrogenalu	Multiple Sclerosis
Yavuz	2011	16891	3085	aripiprazole	MND
Ye	2005	13237	29337	paroxetine	Multiple Sclerosis
Yehuda	1996	12246	6531	bmy 21,502	Alzheimers Disease
Yoon	2003	12151	5552	hrt	Alzheimers Disease
Yoon	2003	12152	5552	hrt	Alzheimers Disease
Yoshida	1989	12016	3122	l-threo-3,4-	Parkinsons Disease
Yoshida	1989	12380	3122	l-threo-3,4-	Parkinsons Disease
Yoshikuni	2003	13107	22035	pramipexol	Parkinsons Disease
Yoshikuni	2003	13108	22035	pramipexol	Parkinsons Disease
Zabad	2007	12501	8916	minocycline	Multiple Sclerosis
Zajicek	2003	12499	9488	delta-9-tetr	Multiple Sclerosis
Zajicek	2003	12504	9488	cannabis ex	Multiple Sclerosis
Zajicek	2005	12520	9167	cannabis ex	Multiple Sclerosis
Zajicek	2005	12522	9167	tetrahydro	Multiple Sclerosis
Zajicek	2012	16808	281	cannabis ex	Multiple Sclerosis
Zajicek	2013	16807	11	dronabinol	Multiple Sclerosis
Zajicek	2013	16921	2806	dronabinol	Multiple Sclerosis
Zarbin	2013	16914	30	fingolimod	Multiple Sclerosis
Zeldowicz	1973	12001	3562	amantadine	Parkinsons Disease
Zesiewicz	2005	13120	23054	levetiraceta	Parkinsons Disease
Zesiewicz	2006	13125	23730	levetiraceta	Huntingtons Disease
Zhang	2013	16809	33	xifeng ding	Parkinsons Disease
Zhang	2008	12487	8693	minocycline	Multiple Sclerosis
Zhang	2002	13322	5633	huperzine-a	Alzheimers Disease
Zhang	2003	13106	22028	entacapone	Parkinsons Disease
Zhao	2007	12767	606	traditional	Parkinsons Disease
Zhou	2007	13329	4567	reinhardt a	Alzheimers Disease
Zhou	2007	13330	4567	donepezil/r	Alzheimers Disease
Zhou	2004	13281	22412	aspirin/harl	Alzheimers Disease
Zhou	2004	13280	22412	huperzine-a	Alzheimers Disease
Zhou	2007	13328	4567	donepezil	Alzheimers Disease

For peer review only

Ziegler	1994	13256	19376	levodopa/c	Parkinsons Disease
Ziegler	1999	13184	14652	piribedil	Parkinsons Disease
Zifko	2002	13105	21788	modafinil	Multiple Sclerosis
Zipp	1995	11490	2754	lamotrigine	Parkinsons Disease
Ziv	1994	11971	8188	nimodipine	MND
Zoing	2006	11876	7852	riluzole	MND
Zuardi	2009	12957	25481	cannabidiol	Parkinsons Disease
Zwanikken	1976	13354	3447	dexetimide	Parkinsons Disease

For peer review only

Publication id	Drug	Disease	Number.of.	EfficacySco	SafetyScore	StudySizeSc	SumQuality
11884 8138 Aisen,	3,4-Diamin	MND	9	2	2	1	13
16925 1041 Aisen,	3,4-Diamin	MND	9	2	3	1	12.5
13037 19034 Bert	3,4-Diamin	MND	24	2.75	2	2	18
12486 10299 Beve	3,4-Diamin	Multiple Sc	34	3.333333	2	2	11.75
13188 28066 Mair	3,4-Diamin	Multiple Sc	7	3.333333	1	1	6.75
12508 10450 Polm	3,4-Diamin	Multiple Sc	10	1	3	1	13.25
12664 10479 Beve	4-Aminopy	Multiple Sc	8	2.75	2	1	13.75
12103 7091 David	4-Aminopy	Alzheimers	14	2	3	2	9.5
12558 10596 Davi	4-Aminopy	Multiple Sc	20	3.25	3	2	10.5
12505 8927 Good	4-Aminopy	Multiple Sc	36	2.333333	2	2	17.5
12485 8674 Good	4-Aminopy	Multiple Sc	206	2.428571	2	3	14.75
12479 8582 Good	4-Aminopy	Multiple Sc	301	3	2	3	18
12453 8298 Good	4-Aminopy	Multiple Sc	239	3.333333	2	3	17
12506 10450 Polm	4-Aminopy	Multiple Sc	10	3	3	1	14.25
12455 9743 Rossir	4-Aminopy	Multiple Sc	54	2.5	3	2	12.75
12620 7173 Wess	4-Aminopy	Alzheimers	14	3	3	2	9
12743 7181 Acierr	acetyl-L-car	Alzheimers	25	2	4	2	12.25
12578 5460 Bianc	acetyl-L-car	Alzheimers	21	3	1	2	9.5
12232 6278 Brook	acetyl-L-car	Alzheimers	334	3	1	3	9.75
12373 7593 Goety	acetyl-L-car	Huntington	10	2	4	1	13
12169 6737 Pette	acetyl-L-car	Alzheimers	12	4	1	2	8
12971 11096 Rai,(	acetyl-L-car	Alzheimers	18	2.25	3	2	11.25
12416 7033 Rai,G.	acetyl-L-car	Alzheimers	36	2.833333	3	2	14.25
13362 6943 Spagr	acetyl-L-car	Alzheimers	130	3.142857	3	3	12.75
12247 6553 Thal,L	acetyl-L-car	Alzheimers	419	2	3	3	16.25
12207 5923 Thal,L	acetyl-L-car	Alzheimers	229	2.166667	2	3	16.25
12724 8078 Apost	Alsamin	MND	35	3.5	1	2	7
12053 1852 Bandi	Amantadin	Parkinsons	23	4	3	2	9
12022 3668 Barbe	Amantadin	Parkinsons	60	4	3	2	13.75
12707 3527 Bauer	Amantadin	Parkinsons	48	4	2	2	11.75
12694 3755 Boma	Amantadin	Parkinsons	36	3	1	2	7.25
12713 3490 Butze	Amantadin	Parkinsons	30	4	3	2	12.25
13336 3541 Callag	Amantadin	Parkinsons	21	3	3	2	6.75
12608 7679 Cande	Amantadin	Huntington	6	3	1	1	12.25
12519 10620 Cohe	Amantadin	Multiple Sc	29	2.5	3	2	13.25
12024 3712 Dallos	Amantadin	Parkinsons	62	2.5	3	2	11.25
12003 3621 Dallos	Amantadin	Parkinsons	69	3	2	2	10
12714 3486 Fahn,:	Amantadin	Parkinsons	23	2	3	2	13
12690 3649 Fehlir	Amantadin	Parkinsons	30	4	3	2	12.5
12683 3595 Fehlir	Amantadin	Parkinsons	21	4	3	2	13.25
12279 3725 Fiescl	Amantadin	Parkinsons	31	3	4	2	9.75
12361 3678 Freed	Amantadin	Parkinsons	23	3.666667	3	2	12.5
12497 10366 Geis	Amantadin	Multiple Sc	45	2	1	2	14.25
12025 3722 Godw	Amantadin	Parkinsons	24	2	4	2	13.5
12691 3657 Griffit	Amantadin	Parkinsons	4	3	1	1	2.75
11821 7400 Heckr	Amantadin	Huntington	8	1.5	1	1	9.5
12282 3716 Hunte	Amantadin	Parkinsons	13	2	4	2	11.25
12754 3591 Kisele	Amantadin	Parkinsons	53	4	3	2	8.25
12280 3517 Kravit	Amantadin	Parkinsons	27	1	1	2	5

For peer review only

12500	10393	Krup	Amantadin	Multiple Sc	119	3.666667	3	3	12.25
11830	7441	Lucet	Amantadin	Huntington	8	3.5	1	1	8.25
11933	7429	Lucet	Amantadin	Huntington	9	4	1	1	9.25
12060	2024	Lugin	Amantadin	Parkinsons	11	4	3	2	13.25
12983	14501	Mals	Amantadin	Parkinsons	53	4	2	2	13
12692	3665	Mann	Amantadin	Parkinsons	13	4	3	2	10.5
12068	2145	Metr	Amantadin	Parkinsons	17	4	1	2	10.25
12283	3713	Millac	Amantadin	Parkinsons	32	2	1	2	10.5
12556	8273	Muns	Amantadin	MND	10	2	3	1	11.5
13344	10759	Mur	Amantadin	Multiple Sc	32	3	3	2	11.25
11826	7426	O'Suil	Amantadin	Huntington	24	3	3	2	17
12312	1906	Paci,C	Amantadin	Parkinsons	20	2	1	2	5.5
12027	3730	Parke	Amantadin	Parkinsons	43	4	3	2	12.5
12269	3747	Parke	Amantadin	Parkinsons	37	3.666667	3	2	12.5
13021	15696	Park	Amantadin	Parkinsons	38	4	4	2	12.25
12000	3500	Pilling	Amantadin	Parkinsons	6	2	1	1	9.5
12512	10713	Plau	Amantadin	Multiple Sc	53	3.5	1	2	8.75
12686	3617	Polloc	Amantadin	Parkinsons	38	1.5	4	2	11
12526	10649	Rose	Amantadin	Multiple Sc	10	3	3	1	13
12698	9964	Sailer,	Amantadin	Multiple Sc	24	2	1	2	11.75
12602	3330	Sandy	Amantadin	Parkinsons	15	3	1	2	6
12607	3410	Saver	Amantadin	Parkinsons	42	4	3	2	14.5
12758	3495	Schub	Amantadin	Parkinsons	56	3	1	2	5.5
12699	3773	Schwa	Amantadin	Parkinsons	163	3	2	3	3.75
12762	3749	Sigwa	Amantadin	Parkinsons	45	1	3	2	4.75
12752	3605	Sigwa	Amantadin	Parkinsons	144	3	2	3	4
12693	3695	Silver,	Amantadin	Parkinsons	50	3.5	3	2	9.75
12687	3618	Silver,	Amantadin	Parkinsons	50	0	2	2	5
12259	2078	Snow,	Amantadin	Parkinsons	24	3.75	4	2	15
12032	1510	Thom	Amantadin	Parkinsons	40	2.5	2	2	16.25
12387	3391	Timbe	Amantadin	Parkinsons	94	1	2	2	6.5
11834	7450	Verha	Amantadin	Huntington	24	3	3	2	17.25
12021	3631	Walke	Amantadin	Parkinsons	42	4	3	2	12.75
12689	3647	Walke	Amantadin	Parkinsons	28	4	3	2	12
12077	2185	Wand	Amantadin	Parkinsons	10	3	1	1	4.25
11952	125	Wolf,E.	Amantadin	Parkinsons	32	3	3	2	17.5
12001	3562	Zeldo	Amantadin	Parkinsons	77	4	1	2	6.25
16941	958	Baston	Amino acid	MND	39	2	1	2	5.25
16971	2268	Brom	Amino acid	MND	88	2	1	2	6.75
16945	2047	Gil,R.	Amino acid	MND	20	2	1	2	8.5
12407	239	Mace,J	Amino acid	Parkinsons	20	2	1	2	12
16970	1638	Testa,	Amino acid	MND	32	2	1	2	5.5
12738	7877	Levits	Amitriptylir	MND	10	1	3	1	6.25
12181	4427	benth	Aspirin	Alzheimers	310	2	2	3	17.25
12972	28169	Mille	Aspirin	Multiple Sc	86	2	1	2	10.5
12857	28172	Mille	Aspirin	Multiple Sc	57	2	1	2	7.75
16858	274	Shayga	Aspirin	Multiple Sc	52	2	3	2	14
11789	7244	Beglir	Atomoxetir	Huntington	20	2	3	2	19
11607	299	Jankov	Atomoxetir	Parkinsons	5	2	4	1	13.75
11623	375	Marsh,	Atomoxetir	Parkinsons	12	4	3	2	9

For peer review only

12351	4022 Mohs	Atomoxetir	Alzheimers	92	2	3	2	19
12509	10456 Bissa	Bromocript	Multiple Sc	18	2	2	2	6.75
12289	7633 Carac	Bromocript	Huntington	2	2	3	1	9.5
11901	7663 Kartzi	Bromocript	Huntington	6	1	3	1	14.5
13204	15580 Mac	Bromocript	Parkinsons	15	2	3	2	14.25
12988	15310 Man	Bromocript	Parkinsons	243	2.333333	3	3	16.25
12988	15310 Man	Bromocript	Parkinsons	243	2.333333	3	3	15.75
12989	15310 Man	Bromocript	Parkinsons	243	2.333333	3	3	16.25
12989	15310 Man	Bromocript	Parkinsons	243	2.333333	3	3	15.75
12988	15310 Man	Bromocript	Parkinsons	243	2	3	3	16.25
12988	15310 Man	Bromocript	Parkinsons	243	2	3	3	15.75
12989	15310 Man	Bromocript	Parkinsons	243	2	3	3	16.25
12989	15310 Man	Bromocript	Parkinsons	243	2	3	3	15.75
12991	15410 Mon	Bromocript	Parkinsons	28	2	3	2	15.25
13195	15387 Mon	Bromocript	Parkinsons	36	2.5	3	2	13.5
12986	15188 Mon	Bromocript	Parkinsons	60	4	2	2	17
13196	15433 Mon	Bromocript	Parkinsons	60	3	3	2	12
13069	15296 Naka	Bromocript	Parkinsons	416	2.5	3	3	13.5
13001	15543 Schn	Bromocript	Parkinsons	40	4	2	2	11.75
16928	2634 Smith	Bromocript	MND	10	2	1	1	10.25
11965	8234 Szulc-	Bromocript	MND	19	2	2	2	4.75
11903	7829 Cudkc	Celecoxib	MND	300	2	2	3	20
12176	4647 Soinir	Celecoxib	Alzheimers	425	2	1	3	19
11810	8241 Appel	Ciclosporin	MND	74	2	1	2	18
16893	451 Ballant	Ciclosporin	MND	36	1.333333	1	2	10
13074	27598 Haas	Ciclosporin	Multiple Sc	73	1	3	2	8.5
13012	27801 Kapp	Ciclosporin	Multiple Sc	89	2	1	2	10.75
13015	27802 Kapp	Ciclosporin	Multiple Sc	194	2	3	3	18
12860	27895 Koet	Ciclosporin	Multiple Sc	81	1.5	2	2	9.75
13081	28816 Rudg	Ciclosporin	Multiple Sc	82	2.285714	2	2	13
13253	19207 Schl	Ciclosporin	Multiple Sc	26	2	3	2	14
13230	29085 The	Ciclosporin	Multiple Sc	547	3.25	3	3	12.75
12374	7637 Caine	Clozapine	Huntington	12	2	2	2	12.25
13048	19616 Chac	Clozapine	Parkinsons	12	3	3	2	9.25
13117	15217 Fact	Clozapine	Parkinsons	19	2.666667	3	2	13.25
12944	20625 Fried	Clozapine	Parkinsons	60	3.5	2	2	20
13080	19384 Jans	Clozapine	Parkinsons	23	4	3	2	11
11853	7532 van V	Clozapine	Huntington	33	2.25	2	2	15
11911	7855 Ferrai	Coenzyme (	MND	31	1	2	2	9.5
11839	7474 Hunti	Coenzyme (	Huntington	347	2	2	3	18.25
11949	7729 Kaufn	Coenzyme (	MND	185	2	2	3	19.75
12040	1646 Mulle	Coenzyme (	Parkinsons	28	3	1	2	14.5
11730	788 NINDS	Coenzyme (	Parkinsons	142	2	2	3	18
12041	1735 Shults	Coenzyme (	Parkinsons	80	3	3	2	19.25
12316	1408 Shults	Coenzyme (	Parkinsons	17	2	2	2	9.5
12544	710 Storch,	Coenzyme (	Parkinsons	131	2.4	2	3	20.5
12067	2528 Strijks	Coenzyme (	Parkinsons	10	2	1	1	8.75
11817	7386 Bende	Creatine	Huntington	20	2	1	2	5.5
11984	7953 Drory	Creatine	MND	14	2	4	2	5.5
11917	7933 Groer	Creatine	MND	175	2.2	3	3	18

For peer review only

12552	7879 Groer	Creatine	MND	175	2	3	3	16
12411	504 Kieburt	Creatine	Parkinsons	200	2	2	3	16
12964	28069 Mali	Creatine	Multiple Sc	12	2	1	2	14.75
12550	7974 Mazzi	Creatine	MND	53	2.5	3	2	6.25
11990	1028 NINDS	Creatine	Parkinsons	200	3	2	3	17
11912	7889 Shefn	Creatine	MND	104	2	2	3	18.5
11816	7378 Tabriz	Creatine	Huntington	13	2	3	2	5
11827	7428 Tabriz	Creatine	Huntington	13	2	3	2	6
11824	7423 Verbe	Creatine	Huntington	41	2	1	2	13.25
11844	8213 Askm	Dextrometl	MND	14	2	2	2	10.75
11972	8144 Blin,C	Dextrometl	MND	49	2	3	2	13.5
12433	2923 Bonu	Dextrometl	Parkinsons	12	4	3	2	5.5
11893	8107 Greda	Dextrometl	MND	45	2	2	2	18.25
11858	8183 Hollar	Dextrometl	MND	13	2	3	2	5.5
12051	2356 Verha	Dextrometl	Parkinsons	6	4	3	1	15.5
11868	7597 Walke	Dextrometl	Huntington	11	0.8	3	2	6.5
11915	7895 Brook	Dextrometl	MND	140	2	3	3	16.25
12513	9083 Panit	Dextrometl	Multiple Sc	150	4	2	3	19.25
11943	7696 Pioro,	Dextrometl	MND	326	4	2	3	17.25
12047	1791 Aarsla	Donepezil	Parkinsons	14	3.333333	3	2	17
13158	26641 Alva	Donepezil	Alzheimers	197	1.777778	2	3	18.75
16663	532 Anders	Donepezil	Alzheimers	90	2	3	2	17
12847	21145 Bara	Donepezil	Alzheimers	10	2	3	1	10
13924	26655 Burn	Donepezil	Alzheimers	114	2	2	3	17.5
11801	7337 Cubo,	Donepezil	Huntington	30	2.333333	2	2	21
13286	22867 Cum	Donepezil	Alzheimers	31	3	3	2	10.75
16677	1875 Dood	Donepezil	Alzheimers	153	2	3	3	17.25
16678	313 Dubois	Donepezil	Parkinsons	550	2.75	1	3	16.5
12911	20830 Evan	Donepezil	Alzheimers	282	4	3	3	8.75
11843	7500 Ferna	Donepezil	Huntington	8	2	3	1	11
13039	19054 Froli	Donepezil	Alzheimers	160	2.75	3	3	15.75
12848	21150 Fusc	Donepezil	Alzheimers	27	2	3	2	16.5
13282	22439 Gaut	Donepezil	Alzheimers	219	4	1	3	12.75
13325	4737 Hu,H.	Donepezil	Alzheimers	100	2.5	4	2	12.5
13272	20763 Iavar	Donepezil	Alzheimers	40	2	1	2	10.5
12909	24616 Jelic,	Donepezil	Alzheimers	248	3.4	3	3	18
13181	27933 Krup	Donepezil	Multiple Sc	69	4	3	2	16.75
13246	18994 Krup	Donepezil	Multiple Sc	120	2.25	3	3	18.75
12031	1508 Leroi,	Donepezil	Parkinsons	16	2.333333	3	2	16.5
13323	5400 Lin,S.	Donepezil	Alzheimers	60	4	4	2	9
12420	1111 Linaza	Donepezil	Parkinsons	10	2	3	1	8.75
11997	1151 Linaza	Donepezil	Parkinsons	10	2.75	1	1	10.5
16734	955 maher-	Donepezil	Alzheimers	198	1.375	3	3	14.5
12261	1762 Mard	Donepezil	Parkinsons	14	3.333333	3	2	17.25
12156	4755 Maru	Donepezil	Alzheimers	38	4	1	2	14.75
12033	1536 Minet	Donepezil	Parkinsons	19	3	1	2	10
12846	19059 Moli	Donepezil	Alzheimers	403	1.333333	1	3	16.25
11720	720 Muller,	Donepezil	Parkinsons	24	3.5	3	2	3
12879	19057 Na,H	Donepezil	Alzheimers	114	3.2	3	3	13
13273	21112 Niko	Donepezil	Alzheimers	14	3.333333	3	2	9.25

For peer review only

12311	1342 Okere	Donepezil	Parkinsons	25	1.5	3	2	15.25
11998	1184 Ravin	Donepezil	Parkinsons	22	2.6	3	2	19
13288	22381 Rekt	Donepezil	Parkinsons	14	2	3	2	11.25
13101	21553 Rocc	Donepezil	Alzheimers	25	1.666667	3	2	11.25
12319	789 Rowan	Donepezil	Parkinsons	45	2.6	1	2	10.75
12202	5851 Thom	Donepezil	Alzheimers	40	3	3	2	12.25
12921	23027 Thor	Donepezil	Parkinsons	70	2	3	2	14.5
12896	19048 Walc	Donepezil	Alzheimers	576	4	1	3	18
16801	1058 Walde	Donepezil	Alzheimers	576	4	1	3	9.75
13328	4567 Zhou,	Donepezil	Alzheimers	68	3.75	4	2	9
12851	19292 Hend	Estrogen	Alzheimers	10	4	1	1	9.25
12248	6581 Hende	Estrogen	Alzheimers	62	3.75	1	2	10.5
12215	6050 Hende	Estrogen	Alzheimers	42	2	1	2	17
13386	11235 Kolle	Estrogen	Huntington	10	2	1	1	5.5
12211	6039 Mulna	Estrogen	Alzheimers	120	1.5	1	3	17.25
12615	6403 Schne	Estrogen	Alzheimers	343	3.25	1	3	9.75
16862	719 The Pa	Estrogen	Parkinsons	23	2.333333	3	2	15.5
12061	2047 Tsang	Estrogen	Parkinsons	40	2.75	3	2	14.5
12209	5984 Wang	Estrogen	Alzheimers	50	2.333333	3	2	16
12036	1564 Avila,.	Fluoxetine	Parkinsons	9	2	3	1	15
11856	7535 Como	Fluoxetine	Huntington	30	2.25	3	2	17.25
16720	234 Kostic,'	Fluoxetine	Parkinsons	18	2.5	3	2	10.25
12546	9079 Moste	Fluoxetine	Multiple Sc	11	3	1	2	6.75
12489	8730 Moste	Fluoxetine	Multiple Sc	40	2.75	3	2	17.75
12332	2561 Simor	Fluoxetine	Parkinsons	5	2	1	1	5.25
16820	130 Albayra	Fluvoxamin	Huntington	1	3	1	1	5.75
11973	8127 Lanna	Fluvoxamin	MND	10	3	1	1	2.25
11904	8039 Caran	Gabapentir	MND	16	4	3	2	6
12331	2461 Chana	Gabapentir	Parkinsons	7	3.5	3	1	5
11897	8073 Mazzi	Gabapentir	MND	110	1.571429	1	3	11.25
11958	7922 Merlii	Gabapentir	MND	120	3	2	3	17
11890	8125 Miller	Gabapentir	MND	152	2.5	3	3	16
16926	1902 Miller	Gabapentir	MND	84	1	3	2	9.75
12549	7976 Miller	Gabapentir	MND	84	2	3	2	18
11983	8006 Miller	Gabapentir	MND	204	2	3	3	18
16977	2563 Palma	Gabapentir	MND	50	2	1	2	7.75
12035	1562 Aarsla	Galantamin	Parkinsons	16	3.25	3	2	10.25
13148	24581 Creti	Galantamin	Alzheimers	138	4	1	3	16.25
13285	22867 Cum	Galantamin	Alzheimers	33	3	3	2	10.75
13149	25210 Gavr	Galantamin	Alzheimers	25	3	3	2	7.75
11642	409 Grace,J	Galantamin	Parkinsons	69	2	3	2	16.75
13122	23474 Hon	Galantamin	Alzheimers	233	2	2	3	14.5
16716	1127 Keller	Galantamin	Alzheimers	18	4	4	2	16.75
13387	21470 Lang	Galantamin	Alzheimers	245	1	1	3	10
11693	525 Litvine	Galantamin	Parkinsons	41	4	3	2	9.25
11640	397 Litvine	Galantamin	Parkinsons	41	4	3	2	14.25
12889	1331 Stahl,	Galantamin	Alzheimers	1419	2	3	4	5.25
16798	893 Van Pu	Galantamin	Alzheimers	128	2	3	3	7.75
12388	4113 Yanch	ginkgo bilol	Alzheimers	121	2.5	3	3	13.5
12389	4113 Yanch	ginkgo bilol	Alzheimers	96	2.5	3	2	13.5

For peer review only

#N/A	468 Diamo	ginkgo bilol	Multiple Sc	1	3	4	1	#N/A
#N/A	319 Lovera	ginkgo bilol	Multiple Sc	1	2	4	1	#N/A
12579	4850 Schne	ginkgo bilol	Alzheimers	1	2	4	1	19.25
12273	4687 Mazz	ginkgo bilol	Alzheimers	1	3.33	4	1	14.25
12273	4687 Mazz	ginkgo bilol	Alzheimers	1	3.33	4	1	16.75
12278	4687 Mazz	ginkgo bilol	Alzheimers	76	3.33	4	2	14.25
12278	4687 Mazz	ginkgo bilol	Alzheimers	76	3.33	4	2	16.75
12838	19779 Cant	Haloperido	Alzheimers	14	2.666667	1	2	13.5
12376	7669 Leona	Haloperido	Huntington	6	2	1	1	12.5
16960	1589 Murp	IGF-1	MND	266	1	1	3	10.75
12534	10946 Dear	Imipramine	Multiple Sc	40	2	3	2	11.75
12445	6944 Teri,L	Imipramine	Alzheimers	61	2	1	2	16
16931	6234 Scelsa	Indinavir	MND	154	1.6	1	3	19
11909	7876 Sclesa	Indinavir	MND	46	2	2	2	13.5
12567	8282 Faree	Isoprinosin	MND	25	2	2	2	19
13070	27536 Gon	Isoprinosin	Multiple Sc	97	2	1	2	10
12962	28055 Mac	Isoprinosin	Multiple Sc	65	1	1	2	8.5
16969	13088 Perc	Isoprinosin	MND	14	2	1	2	14.25
11968	8209 Eisen,	Lamotrigine	MND	67	2	2	2	18
16702	2049 Hayto	Lamotrigine	Multiple Sc	117	2	1	3	17.75
11083	8338 Kapoc	Lamotrigine	Multiple Sc	120	2	3	3	17.5
11083	8338 Kapoc	Lamotrigine	Multiple Sc	120	2	3	3	20.25
12459	8338 Kapoc	Lamotrigine	Multiple Sc	120	2	3	3	17.5
12459	8338 Kapoc	Lamotrigine	Multiple Sc	120	2	3	3	20.25
11082	2480 Shino	Lamotrigine	Parkinsons	14	2	2	2	15.25
11081	6285 Tekin,	Lamotrigine	Alzheimers	11	3	3	2	14.25
11490	2754 Zipp,F	Lamotrigine	Parkinsons	20	2.5	3	2	15.5
12517	10580 Mas	Levamisole	Multiple Sc	41	3.333333	2	2	14.5
12574	8271 Olarte	Levamisole	MND	55	2	3	2	6.25
12571	8259 Olarte	Levamisole	MND	57	3	2	2	12.5
11808	7358 de To	Levetiracet	Huntington	22	2.666667	3	2	10.5
12402	4494 Kyom	Levetiracet	Alzheimers	19	4	1	2	4.5
12901	13059 Statl	Levetiracet	Parkinsons	76	3.333333	2	2	19.75
13126	23886 Stria	Levetiracet	Multiple Sc	14	4	3	2	12.75
12980	13088 Wol	Levetiracet	Parkinsons	32	2	1	2	21
13125	23730 Zesie	Levetiracet	Huntington	9	4	3	1	14.25
12576	4443 Hager	lipoic acid	Alzheimers	43	3	1	2	7
12491	9289 Yadav	lipoic acid	Multiple Sc	37	1	3	2	12
11798	7712 Aggar	Lithium	MND	84	2	2	2	20
11085	7677 Amin	Lithium	Huntington	9	1.333333	1	1	10.75
11919	7674 Carm	Lithium	Huntington	6	2	1	1	9
12326	7702 Chio,/	Lithium	MND	171	2	2	3	19.25
12330	4060 Hamp	Lithium	Alzheimers	71	3	2	2	14.5
13023	15818 Leor	Lithium	Huntington	6	2	1	1	11.5
12335	4133 Leyhe	Lithium	Alzheimers	27	3	1	2	10.75
12187	4412 Macd	Lithium	Alzheimers	10	2	2	1	10.5
11087	7681 Many	Lithium	Huntington	1	3	1	1	2.5
11086	7680 Matts	Lithium	Huntington	2	2	1	1	3.25
13011	15643 McC	Lithium	Parkinsons	21	2	3	2	3.5
12618	7657 Veste	Lithium	Huntington	6	2	1	1	13.75

For peer review only

12130	5424 Asaya	Melatonin	Alzheimers	20	3	3	2	11
12404	6183 Brusc	Melatonin	Alzheimers	55	2	4	2	3.5
11996	1144 Dowli	Melatonin	Parkinsons	40	1.666667	3	2	12
12205	4165 Gehrr	Melatonin	Alzheimers	41	2	1	2	7.5
13104	21712 Jaco	Melatonin	MND	3	2	4	1	1.75
11722	732 Medeir	Melatonin	Parkinsons	20	2.25	1	2	11
12537	3585 Shaw,	Melatonin	Parkinsons	4	2	3	1	2
12124	5360 Singer	Melatonin	Alzheimers	157	2	3	3	18
11096	261 Aarslar	Memantine	Parkinsons	72	3.166667	3	2	19.25
12734	3821 Aguer	Memantine	Alzheimers	539	3	2	3	7.5
12741	5914 Andrc	Memantine	Alzheimers	16	4	1	2	4.75
12741	5914 Andrc	Memantine	Alzheimers	16	4	1	2	8
12800	5914 Andrc	Memantine	Alzheimers	24	4	1	2	4.75
12800	5914 Andrc	Memantine	Alzheimers	24	4	1	2	8
11116	4491 Bakch	Memantine	Alzheimers	470	2.25	2	3	19
11491	7397 Beiste	Memantine	Huntington	27	2	1	2	5.75
11589	617 Borgha	Memantine	Parkinsons	10	2	3	1	8.25
11113	4498 calabı	Memantine	Alzheimers	2000	4	2	4	6.5
11110	4747 Dautz	Memantine	Alzheimers	47	3	3	2	5.5
16909	4353 De Ca	Memantine	MND	63	2	2	2	21
11097	23 Emre,M	Memantine	Parkinsons	199	2.833333	2	3	16
11118	4625 Feldm	Memantine	Alzheimers	404	4	2	3	16
11597	7141 Fleisc	Memantine	Alzheimers	20	2	3	2	13
16691	1942 Fox,C.	Memantine	Alzheimers	153	2.6	3	3	21
16704	170 Holtho	Memantine	Alzheimers	451	4	2	3	9.75
16712	1059 Johan	Memantine	Parkinsons	56	0	3	2	17.5
11105	4594 Jones,	Memantine	Alzheimers	78	2	2	2	11.5
12891	13101 Lars:	Memantine	Parkinsons	42	3	3	2	18.25
16884	2202 Larssc	Memantine	Parkinsons	70	4	3	2	18.25
11093	293 Leroi,I.	Memantine	Parkinsons	25	2	3	2	14.5
12729	344 Levin,C	Memantine	Parkinsons	26	3.4	3	2	11.25
16951	1055 Li,W.	Memantine	Parkinsons	55	4	3	2	10.5
11094	150 Litviner	Memantine	Parkinsons	62	3.375	1	2	7.75
11090	8365 Lover	Memantine	Multiple Sc	116	1.846154	3	3	15.75
11089	2160 Merel	Memantine	Parkinsons	12	4	3	2	12.75
16968	2312 Mielk	Memantine	Alzheimers	327	4	1	3	11.25
11106	3995 Modr	Memantine	Alzheimers	67	3	1	2	15
16747	75 Moreau,	Memantine	Parkinsons	13	3.5	2	2	15.75
11493	230 Olin,J. :	Memantine	Alzheimers	117	2	2	3	8
11088	7334 Ondo,	Memantine	Huntington	12	2.4	3	2	7.5
12900	13054 Ond	Memantine	Parkinsons	40	2	3	2	19.75
11111	4566 Ott,B.	Memantine	Alzheimers	314	1	2	3	4.5
11101	4571 Poma	Memantine	Alzheimers	403	3.75	1	3	15
12569	4386 Porste	Memantine	Alzheimers	433	1.8	2	3	17.25
16853	2487 Raine	Memantine	Alzheimers	377	4	2	3	11.25
16853	2487 Raine	Memantine	Alzheimers	377	4	2	3	13.5
16890	2487 Raine	Memantine	Alzheimers	377	4	2	3	11.25
16890	2487 Raine	Memantine	Alzheimers	377	4	2	3	13.5
11102	5514 Reisb	Memantine	Alzheimers	252	3.25	2	3	18
11107	4836 Reisb	Memantine	Alzheimers	181	3	2	3	9

For peer review only

11497	4560 Riepe	Memantine	Alzheimers	95	3.6	2	2	7.5
16775	681 Saxton,	Memantine	Alzheimers	265	3.5	2	3	17.75
11100	4292 Schmi	Memantine	Alzheimers	36	2.666667	2	2	18
12736	3854 Schmi	Memantine	Alzheimers	53	2.666667	2	2	6.5
11098	4626 Schmi	Memantine	Alzheimers	404	4	1	3	17.25
12732	3272 Schne	Memantine	Parkinsons	67	3	1	2	8.5
13154	26633 Schu	Memantine	Alzheimers	97	3.5	2	2	11.75
11104	5317 Tariot	Memantine	Alzheimers	404	3.5	3	3	18
11108	4516 Van D	Memantine	Alzheimers	350	2.285714	2	3	17
11091	8609 Villosl	Memantine	Parkinsons	60	1	1	2	10.25
11112	4398 Walde	Memantine	Alzheimers	46	2	2	2	14
12836	26634 Weir	Memantine	Alzheimers	47	2.5	3	2	14.75
12904	24048 Wilk	Memantine	Alzheimers	1826	4	3	4	19
16802	2768 Wilkir	Memantine	Alzheimers	278	2	3	3	21
11117	5957 Winbl	Memantine	Alzheimers	167	4	2	3	17
12414	6848 Cutler	Milacemide	Alzheimers	148	2.5	2	3	13.75
12650	6907 Dyske	Milacemide	Alzheimers	228	2	2	3	12.25
11862	7583 Giuffr	Milacemide	Huntington	7	2.5	1	1	14.5
12340	2898 Giuffr	Milacemide	Parkinsons	6	0	4	1	7
11829	7437 Bonel	Minocyclin	Huntington	19	4	3	2	6.25
11818	7394 Bonel	Minocyclin	Huntington	14	2	3	2	6.5
11875	7783 Gordc	Minocyclin	MND	412	1.6	2	3	20.5
11783	7214 Hunti	Minocyclin	Huntington	114	2	3	3	17.5
11822	7401 Hunti	Minocyclin	Huntington	60	2	2	2	14.25
12412	504 Kieburt	Minocyclin	Parkinsons	200	2	2	3	12.5
11991	1028 NINDS	Minocyclin	Parkinsons	200	2	2	3	16.25
11797	7332 Reync	Minocyclin	Huntington	34	1	3	2	2.5
11820	7408 Thom	Minocyclin	Huntington	30	2	4	2	8.75
12501	8916 Zabac	Minocyclin	Multiple Sc	10	3.333333	3	1	6
12487	8693 Zhang	Minocyclin	Multiple Sc	10	3	2	1	8.75
11794	7288 Black	Modafinil	Huntington	20	2.666667	1	2	16.25
12477	8558 Lange	Modafinil	Multiple Sc	21	4	1	2	15.5
12088	5778 Adair,	N-acetyl cy	Alzheimers	47	3	3	2	13.75
12457	8325 Cree,I	Naltrexone	Multiple Sc	80	3	3	2	14.5
12484	8666 Giron	Naltrexone	Multiple Sc	40	2.4	2	2	5.25
12092	7148 Hyma	Naltrexone	Alzheimers	17	2	4	2	12.75
11894	7639 Nutt,J	Naltrexone	Huntington	9	2	4	1	9.75
12086	7162 Poma	Naltrexone	Alzheimers	10	2.333333	1	1	5.5
12339	2822 Rasco	Naltrexone	Parkinsons	18	2	3	2	11.5
12093	7137 Serby	Naltrexone	Alzheimers	9	2	1	1	11
12460	8351 Shara	Naltrexone	Multiple Sc	106	2	3	3	14.5
12720	8078 Apost	Nimodipine	MND	35	3.5	1	2	6.5
16898	2053 Miller	Nimodipine	MND	87	2	3	2	17.25
11867	8148 Miller	Nimodipine	MND	87	2	2	2	15.25
13051	19796 Mor	Nimodipine	Alzheimers	1648	2.25	1	4	17.5
11971	8188 Ziv,I. :	Nimodipine	MND	2	2	4	1	8.75
11963	8249 Norris	Octacosanc	MND	12	2	4	2	12.75
12381	3264 Snide	Octacosanc	Parkinsons	10	2	3	1	10.25
13086	20586 Aars	Olanzapine	Parkinsons	21	4	3	2	7.25
11837	7460 Bonel	Olanzapine	Huntington	1	4	3	1	9.25

For peer review only

11833	7447 Bonel	Olanzapine	Huntington	9	4	3	1	10
11835	7456 Palea	Olanzapine	Huntington	11	2.666667	1	2	9.75
13053	19829 Wolt	Olanzapine	Parkinsons	15	2.666667	3	2	14.5
12547	4660 Freun	Omega 3 Fa	Alzheimers	204	2	3	3	14.25
12480	8597 Shint	Omega 3 Fa	Multiple Sc	10	1.5	3	1	6.5
11881	7853 Meini	Pentoxifylli	MND	400	1.333333	1	3	18.25
12473	10113 Mye	Pentoxifylli	Multiple Sc	14	2	1	2	6.5
12993	28752 Riecl	Pentoxifylli	Multiple Sc	8	1.5	1	1	6.75
12910	13082 Haus	Pramipexol	Parkinsons	259	4	2	3	19.25
12951	24822 Kanc	Pramipexol	Parkinsons	36	4	3	2	12.5
13164	13220 Levir	Pramipexol	Parkinsons	98	4	4	2	8.75
12267	7920 Patte	Pramipexol	MND	17	2.5	1	2	8
16761	841 Poewe	Pramipexol	Parkinsons	539	2.818182	2	3	16
12875	22058 Reicl	Pramipexol	Parkinsons	657	4	2	3	16.25
16779	197 Schapi	Pramipexol	Parkinsons	902	2	2	3	7.75
16780	198 Schapi	Pramipexol	Parkinsons	391	1	2	3	10.75
16781	2563 Schap	Pramipexol	Parkinsons	535	2.125	2	3	21
16718	1136 The P	Pramipexol	Parkinsons	311	2.571429	2	3	17
16937	185 Utsumi	Pramipexol	Parkinsons	34	4	3	2	10.75
12947	21264 Weir	Pramipexol	Parkinsons	306	3	3	3	11.25
12865	20361 Wer	Pramipexol	Parkinsons	69	3.5	3	2	16.75
13107	22035 Yosh	Pramipexol	Parkinsons	325	3	3	3	20
13108	22035 Yosh	Pramipexol	Parkinsons	113	3	3	3	20
11886	8136 Newa	Propranolo	MND	16	3	1	2	5
11987	944 Bara-Ji	Riluzole	Parkinsons	15	2	3	2	14.75
11854	8199 Bensi	Riluzole	MND	155	3.666667	2	3	18.25
12715	7958 Bensi	Riluzole	MND	168	2.2	2	3	16.25
11905	8039 Caran	Riluzole	MND	16	2	1	2	6.25
11823	7416 Hunti	Riluzole	Huntington	63	3.333333	2	2	18.25
12046	1788 Janko	Riluzole	Parkinsons	20	2	3	2	15
12462	9253 Killest	Riluzole	Multiple Sc	16	3.666667	1	2	5.75
11889	8126 Lacon	Riluzole	MND	959	1.625	2	3	17.25
11985	7956 Lacon	Riluzole	MND	516	1	2	3	6
11795	7315 Landv	Riluzole	Huntington	537	2	2	3	17.5
12560	7782 Mors	Riluzole	MND	10	1.5	1	1	5
11937	7950 Niebr	Riluzole	MND	17	1	2	2	4.25
11848	7511 Rosas	Riluzole	Huntington	8	3	3	1	8.75
11838	7466 Seppi,	Riluzole	Huntington	9	2	1	1	8.75
12256	7786 Sivori,	Riluzole	MND	97	2	1	2	4.75
12272	8120 Sojka,	Riluzole	MND	5	4	1	1	5.75
11932	7262 Squiti	Riluzole	Huntington	23	3	1	2	7.5
13243	18411 Ague	Rivastigmin	Alzheimers	429	2	3	3	12.5
16662	981 Alva,G.	Rivastigmin	Alzheimers	782	4	1	3	16.25
13088	20795 Anar	Rivastigmin	Alzheimers	2791	4	3	4	17.75
13118	22521 Bon	Rivastigmin	Parkinsons	541	4	3	3	17
11758	879 Burn,D	Rivastigmin	Parkinsons	541	4	3	3	15.75
12954	25277 Chiu	Rivastigmin	Alzheimers	264	2.666667	1	3	15
13267	20283 Core	Rivastigmin	Alzheimers	699	3.333333	3	3	17.25
13284	22867 Cum	Rivastigmin	Alzheimers	37	4	3	2	10.75
11819	7396 de To	Rivastigmin	Huntington	21	2.333333	2	2	16.75

For peer review only

11796	7328 de To	Rivastigmin	Huntington	21	2.666667	1	2	15
11967	867 Dujard	Rivastigmin	Parkinsons	28	2.6	1	2	12.75
13119	22941 Edw	Rivastigmin	Alzheimers	173	4	2	3	16.75
13128	24619 Figie	Rivastigmin	Alzheimers	146	2	2	3	13
12949	24652 Figie	Rivastigmin	Alzheimers	270	4	2	3	10.75
12315	1655 Fogel	Rivastigmin	Parkinsons	19	4	1	2	9.75
12849	21150 Fusc	Rivastigmin	Alzheimers	11	2.666667	3	2	14.75
12034	1539 Giladi	Rivastigmin	Parkinsons	28	4	3	2	10.75
16693	1057 Gross	Rivastigmin	Alzheimers	1052	4	1	4	9
16708	759 Huolm	Rivastigmin	Multiple Sc	28	4	1	2	15.75
12895	24005 Met	Rivastigmin	Alzheimers	175	3.142857	1	3	14.5
12317	1170 Morg	Rivastigmin	Parkinsons	541	3.75	3	3	19
11993	1106 Poew	Rivastigmin	Parkinsons	273	3	3	3	17.5
12054	1865 Readi	Rivastigmin	Parkinsons	15	3.333333	3	2	9.75
12854	20464 Rosl	Rivastigmin	Alzheimers	34	4	3	2	12.75
12876	18963 Schn	Rivastigmin	Parkinsons	541	4	1	3	15.5
12923	28927 Shay	Rivastigmin	Multiple Sc	60	2	1	2	11.5
12249	6638 Srame	Rivastigmin	Alzheimers	50	2	1	2	16.75
12203	5851 Thom	Rivastigmin	Alzheimers	20	3	3	2	10.25
12321	1083 Wesn	Rivastigmin	Parkinsons	487	4	3	3	18.75
12476	8489 Bielek	Rolipram	Multiple Sc	8	2	3	1	6.25
12450	3291 Casac	Rolipram	Parkinsons	10	2	3	1	10.25
12137	6913 Agnol	Selegiline	Alzheimers	10	4	1	1	12
12673	7004 Camp	Selegiline	Alzheimers	40	4	4	2	13.5
13097	21265 Cara	Selegiline	Parkinsons	501	3	3	3	14.75
16944	1555 Janik,	Selegiline	MND	67	2	1	2	8.25
12566	8206 Jossa	Selegiline	MND	10	2	1	1	7.75
11981	7971 Kwiec	Selegiline	MND	67	2	2	2	14.25
16895	1230 Lange	Selegiline	MND	133	2	3	3	18.5
11896	8094 Lange	Selegiline	MND	133	2	3	3	16
13091	20903 Lyyti	Selegiline	Parkinsons	16	4	3	2	13
13047	19610 Mall	Selegiline	Parkinsons	20	4	1	2	12.75
11845	8204 Mazzi	Selegiline	MND	111	2	2	3	13
16894	758 Mitche	Selegiline	MND	19	1	1	2	7.25
16965	3373 Mitch	Selegiline	MND	52	1	1	2	8.25
12276	8163 Mitch	Selegiline	MND	56	2	2	2	8.5
16878	1056 Ondo	Selegiline	Parkinsons	48	2.333333	3	2	8.25
12013	2892 Parkir	Selegiline	Parkinsons	800	3.5	2	3	15.5
12338	2671 Parkir	Selegiline	Parkinsons	310	2	1	3	9
12428	2390 Parkir	Selegiline	Parkinsons	800	2	2	3	14.75
12241	6450 Sano,	Selegiline	Alzheimers	341	2.5	3	3	14.5
13076	19225 Shou	Selegiline	Parkinsons	800	4	2	3	16
13283	22528 Su,W	Selegiline	Parkinsons	143	3	3	3	9.25
16774	851 Sano,M	Simvastatin	Alzheimers	406	2.2	2	3	19.75
12163	5625 Simor	Simvastatin	Alzheimers	44	2.75	2	2	20
12142	5506 Sjogre	Simvastatin	Alzheimers	19	2.4	3	2	12.75
16843	2633 Soren	Simvastatin	Multiple Sc	307	2	3	3	18.75
16915	91 Tison,F.	Simvastatin	Parkinsons	12	2	4	2	12.75
11950	7758 Cudkc	Sodium phe	MND	40	2	2	2	16
13043	19192 Eagg	Tacrine	Alzheimers	73	4	3	2	14.75

For peer review only

12886	11013 Eagg	Tacrine	Alzheimers	41	2	2	2	15
12974	12410 Esta	Tacrine	Alzheimers	100	2	2	2	14.25
12630	6551 Foster	Tacrine	Alzheimers	1	2.571429	3	1	16.25
12930	12624 Hark	Tacrine	Alzheimers	12	2	1	2	13
12008	2564 Hutch	Tacrine	Parkinsons	7	4	1	1	9
13260	19803 Kauf	Tacrine	Alzheimers	36	2.5	1	2	5.75
12967	11076 Norc	Tacrine	Alzheimers	5	4	1	1	13.25
12834	19241 Wilc	Tacrine	Alzheimers	79	2.25	2	2	17.25
12899	19299 Woc	Tacrine	Alzheimers	154	2.75	2	3	19.75
12945	27869 Kille:	Tetrahydro	Multiple Sc	16	1	1	2	11.5
16908	4296 Webe	Tetrahydro	MND	27	2	2	2	20
16946	6290 Aares	Theophyllir	MND	16	4	2	2	16
12265	1837 Kulise	Theophyllir	Parkinsons	10	2.5	3	1	15.25
11961	8277 Olson	Tilorone	MND	16	2	2	2	14.75
13068	27426 Fost	Tolbutamid	Multiple Sc	40	2	1	2	16.75
12679	3806 Hanse	Tolbutamid	Parkinsons	20	3	3	2	11.25
11928	7925 Cudkc	Topiramate	MND	296	1	2	3	19.25
12782	10968 Silbe	Tranylcypro	Multiple Sc	39	3	4	2	11.5
12102	7094 Tariot	Tranylcypro	Alzheimers	7	2	3	1	8.25
11945	7720 Parry,	Ursodeoxy	MND	18	2	3	2	14.75
11907	7840 Bricht	Valproate	MND	40	2	1	2	6.25
12604	3360 Nutt,J	Valproate	Parkinsons	8	2	3	1	11
16906	3808 Piepe	Valproate	MND	163	2	1	3	21
11828	7728 Piepe	Valproate	MND	163	2	2	3	21
12303	7350 Saft,C	Valproate	Huntington	8	4	1	1	10.25
11813	7740 Swob	Valproate	MND	42	3	3	2	9.75
12375	7649 Symir	Valproate	Huntington	3	2	4	1	8.25
12307	7665 Tan,B	Valproate	Huntington	1	2	1	1	9.5
16794	2670 Tariot	Valproate	Alzheimers	313	2	2	3	19
11871	8145 Miller	Verapamil	MND	72	2	1	2	9.25
12464	8374 Burto	Vitamin D3	Multiple Sc	49	3	4	2	16.75
19019	561 Deraks	Vitamin D3	Multiple Sc	24	3.4	4	2	16.75
19038	676 Etemac	Vitamin D3	Multiple Sc	15	3	4	2	13.25
19031	819 Golan,I	Vitamin D3	Multiple Sc	30	2	4	2	16.75
16846	351 Kampn	Vitamin D3	Multiple Sc	68	2.166667	2	2	19.75
19021	1200 Kimba	Vitamin D3	Multiple Sc	12	2.571429	4	2	9.25
19032	1575 Mosa	Vitamin D3	Multiple Sc	59	2	1	2	17
19033	1829 Pierrc	Vitamin D3	Multiple Sc	156	4	3	3	6.25
19025	2138 Smolc	Vitamin D3	Multiple Sc	15	4	4	2	10.5
16850	451 Soilu-H	Vitamin D3	Multiple Sc	62	3	3	2	17.5
16793	63 Suzuki V	Vitamin D3	Parkinsons	153	4	4	3	18.5
11934	7651 Caro,/	Vitamin E	Huntington	10	2	2	1	11.25
11979	7996 Desnu	Vitamin E	MND	289	3	2	3	17
12589	3055 Facto	Vitamin E	Parkinsons	28	3	1	2	7
11910	7891 Graf,M	Vitamin E	MND	160	1.833333	2	3	14
12014	2892 Parkir	Vitamin E	Parkinsons	800	1.666667	2	3	16.25
12431	2390 Parkir	Vitamin E	Parkinsons	800	2	2	3	15.75
12369	7553 Peyse	Vitamin E	Huntington	81	2	1	2	17
12243	6450 Sano,	Vitamin E	Alzheimers	341	2.75	2	3	14.5
12204	5851 Thom	Vitamin E	Alzheimers	60	0.666667	4	2	12.5

For peer review only

QualityScor	ProductSco	PeerReview	ConflictOfIr	SampleSize	RandomAll	AllocationC	BlindedAss	SimilarBase
3	12	1	0	0	1	1	1	0.5
3	18	1	0	0	0	1	1	0.5
4	44	1	0	1	1	1	1	1
3	40	1	0	0	1	0	1	0.5
1	3.333333	0	0	0	0	0	1	0.5
3	9	1	1	0	1	0	1	1
3	16.5	1	0	0	1	1	1	0.5
2	24	1	0	0	1	0	1	0.5
2	39	1	0	0	0	0	1	0.5
4	37.33333	1	1	0	1	1	1	1
4	58.28571	1	1	1	1	0	1	1
4	72	1	1	1	1	0	1	1
4	80	1	1	1	1	1	1	0
4	36	1	1	0	1	0	1	1
3	45	1	0	1	1	1	1	0.5
2	36	1	0	0	1	0	1	0.5
3	48	1	0	0	1	1	0	0.5
2	12	1	0	0	0	0	0	1
2	18	1	0	0	0	0	0	0.5
3	24	1	0	0	1	0	0	1
2	16	1	0	0	0	0	0	0
2	27	1	0	0	0	0	0	0.5
4	68	0	0	0	1	1	1	1
3	84.85714	1	0	0	1	1	1	0.5
4	72	1	0	0	1	1	1	1
4	52	1	1	1	1	1	1	1
2	14	1	0	0	0	0	0	0.5
2	48	1	0	0	0	0	0	0
3	72	1	0	0	1	0	1	1
3	48	1	0	0	1	0	1	1
2	12	1	0	0	0	0	1	0.5
3	72	1	0	0	1	0	1	1
1	18	1	0	0	0	0	0	0
3	9	1	0	0	1	0	1	1
3	45	1	0	0	1	0	1	1
2	30	1	0	0	1	0	1	0.5
2	24	1	0	0	1	0	0	1
3	36	1	0	0	1	0	1	1
3	72	1	0	0	1	0	1	1
3	72	1	0	0	1	0	1	1
2	48	1	0	0	0	0	0	1
3	66	1	0	0	1	1	1	1
4	16	1	0	1	1	0	1	1
3	48	1	0	0	1	1	1	1
1	3	1	0	0	0	0	0	0
2	3	1	0	0	1	0	1	0.5
2	32	1	0	0	1	0	1	1
2	48	1	0	0	0	0	0	0
1	2	1	0	0	0	0	0	0

For peer review only

3	99	1	0	0	1	0	1	1
2	7	1	0	0	0	0	0	0
2	8	1	0	0	0	0	0	0
3	72	1	0	0	0	0	1	1
3	48	1	0	0	1	0	1	1
2	48	1	0	0	1	0	1	1
2	16	1	0	0	0	0	1	0
2	8	1	0	0	0	1	1	1
3	18	1	0	0	1	0	1	0.5
2	36	1	0	0	0	0	1	1
4	72	1	0	1	1	1	1	1
1	4	1	0	0	0	0	0	0
3	72	1	0	0	1	0	1	1
3	66	1	0	0	1	0	1	1
3	96	1	0	0	1	0	1	1
2	4	1	0	0	0	0	1	1
2	14	1	0	0	0	0	0	1
2	24	1	0	0	1	0	1	1
3	27	1	0	0	1	0	1	1
3	12	1	0	0	1	0	0	1
1	6	1	0	0	0	0	0	0
4	96	1	0	0	1	0	1	1
1	6	1	0	0	0	0	0	0
1	18	1	0	0	0	0	0	0
1	6	1	0	0	0	0	0	0
1	18	1	0	0	0	0	0	0
2	42	1	0	0	1	0	1	0.5
1	0	1	0	0	0	0	1	0
4	120	1	0	0	1	0	1	1
4	40	1	1	1	1	0	1	1
1	4	1	0	0	1	0	0	0
4	72	1	0	1	1	0	1	1
3	72	1	0	0	1	0	1	0.5
3	72	1	0	0	1	0	1	1
1	3	1	0	0	0	0	0	0
4	72	1	1	1	1	0	1	1
1	8	1	0	0	0	0	0	0
1	4	1	0	0	0	0	0	0.5
1	4	1	0	0	0	0	0	0.5
2	8	1	0	0	0	0	1	0.5
3	12	1	1	0	0	0	1	0
1	4	1	0	0	1	0	0	0.5
1	3	1	0	0	0	0	0	0
4	48	1	1	0	1	0	0	1
2	8	1	0	0	1	0	0	1
2	8	1	0	0	1	0	1	0.5
3	36	1	0	1	1	1	1	1
4	48	1	1	0	1	1	1	1
3	24	1	0	0	1	0	1	0.5
2	48	1	0	0	0	0	0	0

4	48	1	0	1	1	1	1	1
1	8	1	0	0	0	0	0	0
2	12	1	0	0	0	0	0	0
4	12	1	0	0	0	0	1	1
4	48	1	0	0	1	1	0	1
4	84	1	0	0	1	0	1	1
4	84	1	0	0	1	0	1	1
4	84	1	0	0	1	0	1	1
4	84	1	0	0	1	0	1	1
4	72	1	0	0	1	0	1	1
4	72	1	0	0	1	0	1	1
4	72	1	0	0	1	0	1	1
4	72	1	0	0	1	0	1	1
4	48	1	0	0	1	0	0	1
3	45	1	0	0	0	0	1	1
4	64	1	0	0	1	0	0	1
3	54	1	0	0	1	0	0	1
3	67.5	1	0	0	1	0	1	1
3	48	1	0	0	1	0	1	0.5
2	4	0	0	0	0	0	1	0.5
1	8	1	0	0	0	0	0	0
4	48	1	1	0	1	1	1	1
4	24	1	1	0	1	0	1	1
4	16	1	0	0	1	0	1	1
2	5.333333	1	0	0	1	1	0	1
2	12	0	0	0	0	0	0	0.5
2	8	1	0	0	0	0	1	0.5
4	72	1	0	1	1	1	1	1
2	12	0	0	0	0	0	1	0.5
3	27.42857	1	0	0	1	1	1	0
3	36	1	0	0	1	0	1	1
3	87.75	1	0	1	1	1	1	1
3	24	1	0	0	1	0	1	0
2	36	1	0	0	0	0	0	0
3	48	1	0	0	0	0	0	0.5
4	56	1	1	0	1	1	1	1
2	48	1	0	0	0	0	0	0
4	36	1	0	0	1	0	0	1
2	8	1	1	1	0	0	0	0
4	48	1	0	1	1	0	1	1
4	48	1	1	1	1	1	0	1
4	24	1	0	0	1	0	1	1
4	48	1	0	1	1	0	1	1
4	72	1	1	1	1	0	1	1
2	16	1	1	0	0	0	1	0
4	57.6	1	1	1	1	1	1	1
2	4	1	0	0	0	0	0	0
1	4	1	0	0	0	0	0	0
1	16	1	0	0	0	0	0	1
4	79.2	1	0	1	1	1	1	1

For peer review only

4	72	1	0	0	1	1	1	1
4	48	1	0	0	1	0	1	1
4	16	1	0	0	1	0	1	1
1	15	1	0	0	0	0	0	0
4	72	1	0	1	1	0	1	1
4	48	1	0	1	1	1	1	1
1	12	1	0	0	0	0	0	0
1	12	1	0	0	0	0	0	0
3	12	1	0	0	0	0	1	1
2	16	1	0	0	0	0	0	1
3	36	1	0	0	1	0	1	1
1	24	1	0	0	0	0	0	0
4	32	1	0	0	1	1	1	1
1	12	1	0	0	0	0	0	0
4	48	1	0	0	1	0	1	1
1	4.8	1	0	0	0	0	1	0
4	72	1	0	0	1	1	1	1
4	96	1	0	1	1	1	1	1
4	96	1	0	1	1	1	1	1
4	80	1	1	0	1	0	1	1
4	42.66667	1	1	1	1	1	1	1
4	48	1	1	0	1	1	1	1
2	12	1	0	0	0	0	0	1
4	48	1	1	1	0	0	1	1
4	37.33333	1	1	1	1	1	1	1
2	36	1	0	0	1	0	0	0.5
4	72	1	1	1	1	1	0	1
4	33	1	1	1	1	1	1	1
2	72	1	0	0	0	0	0	0
2	12	1	0	0	0	0	0	0
4	99	1	1	1	0	0	1	1
4	48	1	0	0	1	0	1	1
3	36	1	0	0	1	0	0	0.5
3	60	1	0	0	0	0	1	1
2	8	1	0	0	0	0	0	1
4	122.4	1	0	0	1	0	1	1
4	96	1	0	1	1	1	1	0
4	81	1	1	1	1	1	1	1
4	56	1	1	0	1	1	1	1
2	64	1	0	0	1	0	0	0.5
2	12	1	0	0	0	0	0	0.5
2	5.5	1	0	0	0	0	0	0
4	49.5	1	1	1	1	1	1	1
4	80	1	0	0	1	0	1	1
4	32	1	0	0	0	0	1	1
2	12	1	0	0	0	0	0	1
4	16	1	1	0	0	0	0	1
1	21	1	0	0	0	0	0	0
3	86.4	1	0	0	0	0	0	0.5
2	40	1	0	0	0	0	0	0.5

4	36	1	1	0	1	1	1	0.5
4	62.4	1	1	0	1	0	1	1
2	24	1	0	0	0	0	0	0.5
2	20	1	0	0	0	0	0	1
2	10.4	1	1	0	0	0	0	1
3	54	1	0	0	1	0	0	1
4	48	1	0	0	0	0	0	0.5
4	48	1	1	0	1	0	0	1
2	24	1	1	0	0	0	0	0
2	60	1	0	0	1	0	0	0.5
2	8	1	0	0	0	0	0	0.5
2	15	1	0	0	0	0	0	1
4	16	1	0	1	1	1	1	1
1	2	1	0	0	0	0	1	0.5
4	18	1	0	1	1	1	0	1
2	19.5	1	0	0	1	0	0	0
4	56	1	0	0	0	0	1	1
4	66	1	0	0	0	1	0	0
4	56	1	0	0	1	1	0	1
4	24	1	0	0	1	0	1	1
4	54	1	0	0	1	1	1	1
2	30	1	0	0	0	0	0	0
1	6	1	0	0	0	0	0	0
4	66	1	1	0	1	1	1	1
1	2	1	0	0	0	0	0	0
1	3	1	0	0	0	0	0	0.5
1	3	1	0	0	0	0	0	0
1	24	1	0	0	0	0	0	0.5
1	10.5	1	0	0	0	0	0	0
2	9.428571	1	0	0	1	0	0	1
4	72	1	0	1	1	1	0	1
4	90	1	0	1	1	0	1	1
2	12	1	0	0	1	0	1	0.5
4	48	1	0	1	1	1	1	1
4	72	1	0	1	1	1	1	1
2	8	1	0	0	1	0	0	0.5
2	39	1	0	0	0	0	0	0
4	48	1	0	0	1	1	0	1
2	36	1	0	0	1	0	0	0.5
2	36	1	0	0	0	0	0	0.5
4	48	1	1	0	1	0	0	1
4	48	1	0	0	1	0	0	1
4	128	1	1	0	1	0	0	1
2	6	1	0	0	0	0	0	0.5
2	48	1	0	0	0	0	0	0.5
4	96	1	0	0	1	0	0	1
1	24	1	0	0	0	0	0	0.5
2	36	1	0	0	0	0	0	0.5
3	67.5	1	0	0	1	1	1	1
3	45	1	0	0	1	1	1	1

For peer review only

#N/A	#N/A	#N/A	#N/A	#N/A	#N/A	#N/A	#N/A	#N/A
#N/A	#N/A	#N/A	#N/A	#N/A	#N/A	#N/A	#N/A	#N/A
4	32	1	1	1	1	1	1	1
4	53.28	1	0	0	1	0	0	1
4	53.28	1	0	0	1	1	1	1
4	106.56	1	0	0	1	0	0	1
4	106.56	1	0	0	1	1	1	1
3	16	1	0	0	1	0	1	0.5
3	6	1	0	0	1	0	0	0.5
2	6	1	1	0	0	0	1	0.5
3	36	1	0	0	1	1	1	0.5
4	16	1	0	0	1	1	1	1
4	19.2	1	1	0	1	1	1	1
3	24	1	1	0	1	1	1	1
4	32	1	0	0	1	1	1	1
2	8	0	0	0	1	0	0	1
2	4	0	0	0	0	0	0	0.5
4	16	1	0	0	1	0	1	1
4	32	1	0	0	1	0	1	1
4	24	1	1	1	1	1	1	0.5
4	72	1	1	1	1	1	1	1
4	72	1	1	1	1	1	1	1
4	72	1	1	1	1	1	1	1
4	72	1	1	1	1	1	1	1
4	32	1	0	0	0	0	1	1
4	72	1	0	0	1	1	1	1
4	60	1	0	0	1	0	0	1
4	53.33333	1	0	0	1	0	1	1
1	12	1	0	0	1	0	1	0
3	36	1	0	1	1	1	1	0.5
2	32	1	0	0	0	0	1	1
1	8	1	1	0	0	0	0	0
4	53.33333	1	1	1	1	1	1	1
3	72	1	0	0	0	1	1	0
4	16	1	1	1	1	1	1	1
4	48	1	0	0	0	0	0	1
2	12	1	0	0	0	0	0	0
3	18	1	0	0	1	0	1	1
4	32	1	1	1	1	1	1	1
2	2.666667	1	0	0	0	1	1	1
2	4	1	0	0	0	0	1	1
4	48	1	1	1	1	0	1	1
4	48	1	1	1	1	0	0	1
3	6	1	0	0	1	0	1	1
2	12	1	0	0	1	0	0	1
2	8	1	0	0	0	0	0	1
1	3	1	0	0	0	0	0	0
1	2	1	0	0	0	0	0	0
1	12	1	0	0	0	0	1	0
3	6	1	0	0	1	0	1	1

For peer review only

2	36	1	0	0	1	0	1	0.5
1	16	1	0	0	0	0	0	0
3	30	1	0	0	1	0	1	1
2	8	1	0	0	1	0	1	0
1	8	1	0	0	0	0	0	0
2	9	1	0	0	1	1	1	1
1	6	1	0	0	0	0	0	0
4	72	1	1	1	1	0	1	1
4	76	1	1	1	1	1	1	1
2	36	1	1	0	0	0	0	0
1	8	1	0	0	0	0	0	0
2	16	1	0	0	0	0	0	1
1	8	1	0	0	0	0	0	0
2	16	1	0	0	0	0	0	1
4	54	1	0	1	1	1	1	1
1	4	1	0	0	0	0	0	0.5
2	12	1	1	0	0	0	0	1
1	32	1	0	0	0	0	0	0
1	18	1	0	0	0	0	0	0
4	32	1	1	1	1	1	1	1
4	68	1	1	0	1	0	1	1
4	96	1	0	0	1	0	1	1
3	36	1	0	0	0	0	0	1
4	93.6	1	1	1	1	1	1	1
2	48	1	1	0	0	0	0	0
4	0	1	1	0	1	0	1	1
3	24	1	0	0	1	0	1	1
4	72	1	1	0	1	1	1	1
4	96	1	1	0	1	0	1	1
4	48	1	1	0	1	0	0	1
2	40.8	1	0	0	0	0	0	1
2	48	1	0	0	0	0	0	0.5
2	13.5	1	0	0	0	0	0	0.5
4	66.46154	1	0	1	1	1	1	0
3	72	1	0	0	1	0	0	0.5
2	24	1	0	0	0	0	0	1
4	24	1	0	1	1	1	1	0.5
4	56	1	1	0	1	0	0	1
2	24	1	1	0	0	0	0	0
2	28.8	1	0	0	0	0	0	0.5
4	48	1	1	0	1	1	1	1
1	6	1	0	0	0	0	0	0.5
4	45	1	0	0	1	0	1	1
4	43.2	1	0	1	1	1	1	1
2	48	1	0	0	0	0	0	0
3	72	1	0	0	0	0	0	1
2	48	1	0	0	0	0	0	0
3	72	1	0	0	0	0	0	1
4	78	1	1	0	1	1	1	1
2	36	1	1	0	0	0	0	0

For peer review only

2	28.8	1	0	0	0	0	0	0
4	84	1	1	1	1	0	1	1
4	42.66667	1	1	0	1	0	1	1
1	10.66667	1	1	0	0	0	0	0
4	48	1	1	0	1	0	1	1
2	12	1	0	0	0	0	1	0.5
3	42	1	0	0	0	0	0	1
4	126	1	1	1	1	1	1	1
4	54.85714	1	1	1	1	0	0	1
2	4	1	1	0	1	0	1	0.5
3	24	1	1	0	1	0	1	1
4	60	1	1	0	0	0	0	1
4	192	1	1	0	1	0	1	1
4	72	1	1	1	1	1	1	1
4	96	1	0	1	1	0	1	1
3	45	1	0	1	1	0	1	1
3	36	1	0	0	1	0	1	1
4	10	1	0	0	1	0	1	1
2	0	1	0	0	0	0	0	0
1	24	1	0	0	0	0	0	0.5
1	12	1	0	0	0	0	0	0
4	38.4	1	1	1	1	1	1	1
4	72	1	1	1	1	0	1	1
4	32	1	0	1	1	0	1	1
3	36	1	0	0	1	0	1	0.5
4	48	1	0	0	1	0	1	1
1	6	1	0	0	0	0	0	0
2	32	1	0	0	0	0	0	0
1	10	1	0	0	0	0	0	0
2	12	1	0	0	0	0	0	0
4	21.33333	1	1	0	1	0	1	1
4	32	1	1	0	1	0	1	1
3	54	1	0	0	1	0	0	1
4	72	1	0	0	1	0	1	1
1	9.6	1	0	0	0	0	0	0
3	48	1	0	0	0	0	1	1
2	16	1	0	0	0	0	1	1
1	2.333333	1	0	0	0	0	0	0
3	36	1	0	0	1	0	1	1
2	4	1	0	0	0	0	1	1
4	72	1	0	0	1	0	1	1
1	7	1	0	0	0	0	0	0.5
4	48	1	0	0	1	0	1	1
4	32	1	0	0	1	0	0	1
4	36	1	0	0	1	0	1	1
2	16	1	0	0	0	0	1	0.5
3	48	1	0	0	1	1	1	0.5
2	12	1	0	0	0	0	1	0.5
2	48	1	0	0	0	0	0	0
2	24	1	0	0	0	0	0	0

For peer review only

2	24	1	0	0	0	0	0	0
2	10.66667	1	0	0	0	0	0	0
4	64	1	1	0	0	0	0	1
4	72	1	1	0	1	0	1	1
1	4.5	1	0	0	0	0	0	0
4	16	1	1	0	1	0	1	1
1	4	1	0	0	0	0	0	0
1	1.5	0	0	0	0	0	0	0.5
4	96	1	1	0	1	1	1	1
3	72	1	0	0	0	0	0	0
2	64	1	0	0	0	0	0	0.5
2	10	1	1	0	0	0	0	0.5
4	67.63636	1	1	1	1	1	1	1
4	96	1	1	0	0	0	0	1
2	24	1	1	0	0	0	0	0
2	12	1	1	0	1	1	1	1
4	51	1	1	1	1	1	1	1
4	61.71429	1	1	1	1	1	1	1
2	48	1	1	0	1	0	0	0
2	54	1	0	0	0	0	0	1
4	84	1	0	0	1	0	0	1
4	108	1	0	1	1	1	1	1
4	108	1	0	1	1	1	1	1
1	6	1	0	0	0	0	0	0.5
4	48	1	0	1	1	1	1	1
4	88	1	0	1	1	0	1	1
4	52.8	1	0	1	1	0	1	0
1	4	1	0	0	0	0	0	0.5
4	53.33333	1	0	1	1	1	1	1
4	48	1	0	0	1	0	1	1
1	7.333333	1	0	0	0	0	0	0
4	39	1	0	0	1	0	1	1
1	6	1	0	0	0	0	0	0
4	48	1	0	1	1	1	1	1
1	1.5	1	0	0	0	0	0	0
1	4	1	0	0	0	0	0	0
2	18	1	0	1	0	0	0	0
2	4	1	0	0	0	0	0	0
1	4	1	0	0	0	0	0	0
1	4	1	0	0	0	0	0	0
2	12	1	0	0	0	0	0	1
3	54	1	1	1	0	0	0	1
4	48	1	1	0	1	0	1	1
4	192	1	0	0	1	0	1	1
4	144	1	0	0	1	0	0	1
4	144	1	0	0	1	0	1	1
4	32	1	1	0	0	0	0	1
4	120	1	0	0	1	1	1	1
2	48	1	0	0	1	0	0	0.5
4	37.33333	1	0	0	1	0	0	1

For peer review only

4	21.33333	1	0	0	0	0	0	1
3	15.6	1	1	0	1	0	0	1
4	96	1	1	0	0	0	0	1
3	36	1	0	0	0	0	0	1
2	48	1	0	0	0	0	0	1
2	16	1	0	0	0	0	0	0
4	64	1	0	0	1	0	0	1
2	48	1	0	0	0	0	0	0
2	32	1	1	0	0	0	0	1
4	32	1	1	0	1	0	1	1
4	37.71429	1	1	1	0	0	0	1
4	135	1	0	1	1	0	1	1
4	108	1	1	0	1	0	1	1
2	40	1	0	0	0	0	0	0
3	72	1	0	0	0	0	0	1
4	48	1	1	0	1	0	1	1
3	12	0	0	0	1	0	1	0.5
4	16	1	0	0	0	0	1	1
2	36	1	0	0	0	0	0	1
4	144	1	1	0	1	0	1	1
1	6	1	0	0	0	0	0	0
2	12	1	0	0	1	0	1	0
3	12	1	0	0	1	0	1	1
3	96	1	0	0	1	0	1	1
4	108	1	0	1	1	0	0	0.5
2	8	0	0	0	1	0	0	0.5
2	4	1	0	0	1	0	1	0.5
4	32	1	0	0	1	0	0	1
4	72	1	0	1	1	1	1	1
4	72	1	0	1	1	0	1	1
3	72	1	0	0	1	0	1	1
3	24	1	0	0	1	0	0	1
3	36	1	0	0	1	0	0	1
2	4	1	0	0	0	0	0	0.5
2	4	1	0	0	1	0	0	0.5
2	16	1	0	0	0	0	1	1
2	28	1	1	0	0	0	0	0.5
4	84	1	0	0	1	1	1	1
2	12	1	0	0	0	0	0	0
4	48	1	0	0	1	0	1	0.5
4	90	1	1	0	1	0	1	0
4	96	1	0	1	1	1	1	1
2	54	1	0	0	0	0	0	0.5
4	52.8	1	1	0	1	1	1	1
4	44	1	1	0	1	1	1	1
3	43.2	1	0	0	0	0	0	1
4	72	1	1	1	1	1	1	1
3	48	1	1	0	1	0	0	1
4	32	1	1	0	0	0	0	1
4	96	1	0	0	1	0	1	0

For peer review only

4	32	1	0	0	0	0	0	1
4	32	1	0	0	0	0	0	1
4	30.85714	1	1	1	1	1	1	1
3	12	1	0	0	0	0	1	1
2	8	1	0	0	0	0	0	1
1	5	1	0	0	0	0	0	0
3	12	1	0	0	0	0	0	0.5
4	36	1	0	0	1	0	1	1
4	66	1	0	1	1	1	1	1
3	6	0	0	0	1	0	1	1
4	32	1	1	1	1	1	1	1
4	64	1	0	0	0	0	1	1
4	30	1	0	0	1	1	0	1
4	32	1	0	0	1	0	1	1
4	16	1	0	0	1	0	1	1
2	36	1	0	0	1	0	1	1
4	24	1	1	1	1	1	1	1
3	72	1	0	0	0	1	1	1
2	12	1	0	0	0	0	1	1
4	48	1	1	0	1	1	1	0.5
1	4	1	0	0	0	0	0	0.5
2	12	1	0	0	1	0	0	0
4	24	1	1	1	1	1	1	1
4	48	1	1	1	1	1	1	1
2	8	1	1	0	0	0	0	1
2	36	1	1	0	0	0	0	0
2	16	1	0	0	0	0	0	0
2	4	1	0	0	0	0	0	0
4	48	1	1	1	1	0	1	1
2	8	1	0	1	0	0	0	0.5
4	96	1	1	1	1	0	0	1
4	108.8	1	1	0	1	0	1	0.5
3	72	1	1	0	1	0	0	0
4	64	1	1	1	1	0	1	1
4	34.66667	1	1	0	1	1	1	1
2	41.14286	1	1	0	0	0	0	0
4	16	1	1	0	1	0	1	1
1	36	1	1	0	0	0	0	0
2	64	1	1	0	0	0	0	0
4	72	1	1	1	1	0	1	1
4	192	1	0	0	1	1	1	1
2	8	1	0	0	0	0	1	0.5
4	72	1	0	1	1	0	1	1
2	12	1	0	0	0	0	0	1
3	33	1	0	1	1	0	1	1
4	40	1	0	0	1	0	1	1
4	48	1	0	0	1	0	1	1
4	16	1	0	1	1	1	1	1
4	66	1	0	0	1	0	0	1
3	16	1	0	0	1	0	1	1

For peer review only

EligibilityCr	PrimaryOut	IntentionTc	CompleteA	NonSelectiv	NoOtherLir	ConfidentIr	CohortsFro	ConfidentP
1	1	0	0	0	1	1	0.75	0.75
0	1	0.5	1	1	0.5	1	0.75	0.75
0.5	1	0.5	1	1	1	1	1	0.75
1	1	0	0.5	0.5	1	1	0.75	0.75
0	0	0.5	0.5	0.5	0.5	0.5	0.75	0.75
1	0	0	0	0.5	1	1	1	1
1	1	0	0	1	1	1	0.75	1
0	0	0	1	0.5	1	1	0.75	0
1	0	0.5	0.5	0.5	1	1	0.75	0.75
1	1	1	1	0	1	1	0.75	1
0	1	1	0	0	1	1	0.75	0.75
1	1	1	1	0	1	1	1	1
1	1	1	1	0	1	1	1	1
1	0	0	0	1	1	1	1	1
1	0	0	0	0	1	1	0.75	0.75
0	0	0	0	0	1	1	1	1
0	0.5	0.5	1	1	0.5	0.5	0.75	0.75
1	1	0	0	0	0	1	0.75	0.75
1	1	0	0	0	1	1	0.75	0.75
1	1	0	1	1	0	1	1	0.75
0	1	0	1	0	0	1	0.25	0.75
1	0	0	0	1	1	1	1	0.75
1	1	0	1	0	0.5	1	1	0.75
1	1	0.5	0	0	0	0	1	0.75
1	1	1	1	0	1	1	0.75	0.75
1	1	0	0	1	1	1	0	0.75
1	1	0.5	0	0	0.5	0.5	0.25	0.25
1	1	0.5	0	0.5	0	1	0	0.75
0	1	0	0.5	1	1	1	1	0.75
0	0	0	0	0.5	1	1	1	0.75
0	0	0	0	0	0	1	0.75	0.75
0	0	0	0	1	1	1	1	0.75
0	1	0	0	1	0	1	0.25	0.25
0	0	0	1	1	0	1	1	0.75
1	0	0	0	1	1	1	1	0.75
0	0	0	0	0.5	1	1	1	0.75
0	0	0	0	0.5	1	1	1	0.75
1	0	0	0	0	1	1	1	0.75
0	0	0	0	0.5	1	1	1	0.75
1	0	0	0	1	1	1	1	0.75
0	0	0	0	0.5	1	1	1	0.75
0	0	0	0	0.5	1	1	1	0.75
1	1	0	0	1	1	1	1	0.75
0	0	0	0	1	1	1	1	1
0	0	0	0	0	0	0	0	0.75
0	0	0	0	0	0	1	1	0.75
0	0	0	0	1	0	1	1	0.75
0	0	0	0	1	1	1	0	0.75
0	0	0	0	0.5	0	1	0	0.75

For peer review only

1	0	0	0	1	1	1	1	0.75
1	1	0	0	0	1	1	0	0.75
1	0	0	1	1	1	1	0	0.75
1	1	0	1	1	0	1	1	0.75
0.5	1	0.5	0.5	0.5	0.5	1	1	0.75
0	0	0	0	0.5	0	1	1	0.75
1	1	0	1	1	1	1	0	0.75
0	0	0	0	0.5	0	1	0.75	0.75
1	0	0	0	0	0	1	1	0.75
0	0	0	0	1	0	1	1	0.75
1	1	0	1	1	1	1	1	0.75
0	1	0	0	0.5	0	1	0	0.75
0	0	0	0	1	1	1	1	1
0	0	0	1	1	0	1	1	1
0	0	0	0	1	1	1	1	0.75
0	0	0	0	0.5	0	1	1	0.75
0	0	0	0	1	0	1	0.75	0.75
0	0	0	0	0	1	1	1	0.75
1	1	0	0	0	1	1	1	0.75
1	0	0	0	0.5	1	1	1	0.75
0	1	0	0	1	0	1	0	0.75
1	0	0	1	1	1	1	1	0.75
0	0	0	0	0.5	0	1	0	0.75
0	0	0	0	0	0	1	0	0.75
0	0	0	0	0	0	1	0	0.75
0	0	0	0	0.5	0	0	0	0.75
0	0	0	0	0.5	0	1	1	0.75
0	0	0	0	0	0	1	0	0.75
1	1	0	1	1	1	1	1	0.75
0	1	1	0.5	1	1	1	1	0.75
0	0	0	0	0.5	0	1	0.25	0.75
1	1	1	1	1	1	1	1	1
1	0	0	0	1	1	1	1	0.75
0	0	0	0	0	1	1	1	0.75
0	0	0	0	0	0	1	0	0.75
1	1	1	1	1	1	1	1	0.75
0	0	0	0	0.5	1	1	0	0.75
0	1	0	0	0	0	0.5	0.25	0.25
0	0	0.5	0	0	0.5	0.5	0.75	0.75
0	0	0.5	0	0.5	0.5	0.5	0.75	0.75
1	1	0.5	1	1	0	0.5	0.75	1
0	0	0	0	0	0	0.5	0.25	0.25
0	0	0.5	0	0.5	0	0.5	0.75	0.75
1	1	1	1	1	1	1	1	1
0	0	0	0	0.5	1	1	1	0.75
0	0	0	0.5	0	0.5	0	1	0.25
1	0	1	1	0	1	0.5	1	0.25
1	1	1	1	1	1	1	1	0.75
1	1	1	1	1	0	1	1	0.75
1	1	0	1	0.5	0	1	0	0.75

For peer review only

1	1	1	1	1	1	1	1	0.75
1	0	0	0.5	0.5	0	0.5	0	0.75
0	1	1	0.5	0.5	0	1	0.75	1
1	1	1	1	1	1	1	1	0.75
1	0	0	1	1	1	1	0.75	0.75
1	1	0.5	1	1	1	1	0.75	0.75
1	1	0.5	1	1	1	1	0.75	0.75
1	1	0.5	1	1	1	1	0.75	0.75
1	1	0.5	1	1	1	1	0.75	0.75
1	1	0.5	1	1	1	1	0.75	0.75
1	1	0.5	1	1	1	1	0.75	0.75
1	1	0.5	1	1	1	1	0.75	0.75
1	1	0.5	1	1	1	1	0.75	0.75
1	1	0.5	1	1	1	1	0.75	0.75
1	1	0.5	0.5	0.5	1	1	0.75	0.75
1	1	1	1	1	1	1	1	1
1	1	0	1	0	1	1	0.75	0.75
1	1	0	0.5	1	0	0.5	0.75	0.75
0.5	0	0.5	0.5	0.5	0.5	0.5	0.75	0.75
0.5	0.5	0.5	0.5	0.5	0.5	0.5	0.75	0.75
0	0	0	0.5	0.5	0	0.5	0	0.75
1	1	1	1	1	1	1	1	1
1	1	1	1	1	1	1	1	1
1	1	1	1	1	1	1	1	1
0	1	0	0	1	0	1	0.25	0.75
0.5	0.5	0	0.5	0.5	0.5	0.5	0.75	0.75
0.5	0.5	0.5	0.5	0.5	0.5	0.5	0.75	0.75
1	1	0	1	1	1	1	0.75	1
0.5	0	0.5	0.5	0.5	0.5	0.5	0.75	0.75
1	1	1	1	1	0.5	0.5	0	0.25
1	0	0	1	0.5	1	1	1	0.75
1	0	0.5	0	0	0	0	0.75	0.75
0.5	0.5	0.5	0.5	1	1	0.5	0.25	0.75
0	0.5	0.5	1	1	1	0.5	0	0.75
1	1	1	1	1	1	1	0	1
1	1	1	1	1	1	1	1	1
1	1	0.5	1	1	1	0.5	0.25	0.75
1	1	0.5	1	1	1	1	0.75	0.75
1	1	1	0	0	0	1	0	1
1	1	1	1	1	1	1	1	0.75
1	1	1	1	1	1	1	1	1
1	1	0.5	0.5	0.5	0	1	1	0.75
1	1	1	1	1	1	1	1	0.75
1	1	1	1	1	1	1	1	0.75
1	1	0	0	0	0	1	0	0.75
1	1	1	1	1	1	1	1	0.75
1	1	1	0	0.5	0	1	0	0.75
0	1	0	0	0.5	0	1	0	0.75
0	0	0	0	0.5	0	1	0	0.75
1	0	1	1	1	1	1	1	0.75

For peer review only

1	0	1	1	1	1	1	1	0.75
1	1	1	1	1	1	1	1	0.75
0	1	1	1	1	1	1	1	0.75
1	1	0	0	0.5	0	1	0	0.75
1	1	1	1	1	1	1	1	0.75
1	1	1	1	1	1	1	1	0.75
0	0	0	0	0.5	0	1	0	0.75
1	0	0	0	0.5	0	1	0	0.75
1	1	1	1	0.5	0	1	0.25	0.75
0	1	0	0	0.5	0	1	1	0.75
1	0	1	0	0.5	1	1	1	0.75
0	1	0	0	0.5	0	1	0	0.75
1	1	1	1	1	1	1	1	0.75
1	0	0	0	0	0	1	0	0.75
0	1	1	1	1	1	1	1	0.75
0	1	0	0	0.5	0	1	0	0.75
1	1	1	1	1	0	1	1	0.75
1	1	1	1	1	1	1	1	0.75
1	0	1	0	1	1	1	1	0.75
1	0	0.5	1	1	1	1	1	1
1	1	1	1	1	0	1	1	1
1	1	1	1	1	1	1	0.25	0.75
1	0	1	1	1	1	0	0	0.75
1	1	1	1	1	0.5	1	1	1
1	1	1	1	1	1	1	1	1
1	1	0.5	1	0	0.5	0.5	0.75	0.75
1	1	1	1	1	0.5	1	0.75	0.75
1	0	1	1	1	1	1	0	0.25
0.5	0.5	1	0	0.5	1	0.5	0	0.75
1	1	0.5	1	1	1	0.5	0	1
1	1	0	0.5	1	1	1	1	0.75
1	1	1	1	1	1	1	1	0.75
0.5	0.5	0.5	0.5	1	0.5	0.5	0.75	0.75
1	1	0.5	1	0.5	0	0.5	1	0.75
1	0	0.5	0.5	1	0	0	1	0.75
1	1	1	1	1	1	1	1	1
1	1	1	1	0	1	1	0.75	0.75
1	1	1	1	1	0.5	1	0.75	0.75
1	1	1	1	1	1	1	1	0.75
1	1	0	0.5	0.5	0.5	0.5	0.75	0.25
1	1	1	1	1	0	0.5	0.25	0.25
1	1	0.5	1	1	1	0.5	0	1
1	1	1	1	1	0	0.5	0	0.25
1	1	0.5	1	1	1	1	1	1
1	0	1	1	1	1	1	1	0.75
1	1	0	1	1	1	0.5	1	0.25
1	1	1	1	1	1	1	1	1
0	0	0	0	0	0	0	0	0.25
1	1	1	1	1	1	0.5	0	1
0	0	1	1	1	1	0	0	0.75

For peer review only

For peer review only - http://bmjopen.bmj.com/site/about/guidelines.xhtml

1	1	1	1	1	1	0.5	0.25	0.75
1	1	1	1	1	1	1	1	1
0.5	0.5	1	0.5	0.5	0.5	0.5	1	0.75
1	1	1	0	0	0.5	1	0.75	0.75
1	0	0.5	0.5	0.5	1	0.5	0	0.75
0.5	1	0	1	1	0	1	0.75	0.75
1	1	1	1	1	1	1	0.75	1
1	1	1	1	1	1	1	1	1
1	0	1	1	1	1	0	0	0.25
1	1	0.5	0.5	0.5	0.5	0.5	0.75	0.25
0.5	1	0	0.5	0.5	0.5	0.5	0.75	0.75
1	1	0.5	0	0	0.5	1	0.75	0.75
1	1	0	1	1	1	1	0.75	1
0	0	0.5	0	0	0	0.5	0.25	0.75
1	1	1	1	1	1	1	0.75	1
1	0	0.5	0	0.5	1	0.5	0.75	0.75
1	0.5	1	1	1	1	1	1	1
1	0.5	1	1	1	1	1	1	0.75
1	1	1	1	1	1	1	0.75	0.75
1	1	1	1	1	0	1	1	0.75
1	1	0	1	1	1	1	1	1
1	0.5	0.5	1	1	1	0.5	0	0.75
0	1	1	0	0.5	0	1	0	0.75
1	1	0	1	1	1	1	1	0.75
0	0	0	0	0	1	1	0	0.75
0.5	0.5	0.5	0.5	0.5	0	0	0	1
0	0	0	0	0	0	0	0	0.25
1	0	0.5	0	0.5	0	0.5	0.25	0.25
0	0	0	0	0.5	0	1	0	0.75
1	0	1	0.5	0	1	1	0	0.75
1	1	1	1	1	1	1	1	0.75
1	1	1	0.5	0.5	1	1	1	0.75
0	0	0.5	0	0.5	0	0.5	0.75	0.75
1	1	1	1	1	1	1	1	0.75
1	1	1	1	1	1	1	1	0.75
0	0	0.5	0	0	0	0.5	0.75	0.75
1	1	0.5	1	1	1	0.5	0	0.75
1	1	1	1	1	1	1	0.75	0.75
1	1	0.5	1	0	0.5	0.5	0.75	0.75
1	0	0.5	0.5	0.5	0	0.5	0.75	0.75
1	1	1	1	1	1	1	1	0.75
1	0.5	1	1	1	0.5	1	0.75	0.75
1	1	0.5	1	1	0.5	1	1	1
0.5	0.5	0.5	0.5	0.5	0.5	0.5	0.75	0.75
0.5	0.5	0.5	0.5	0.5	0.5	0.5	0.75	0.75
1	0.5	1	1	1	1	1	1	0.75
1	0	0.5	0.5	0.5	0	0	0	0.25
0.5	0	0.5	1	0	0	0	0.75	0.75
1	1	0	0	0	0.5	1	0.75	0.75
1	1	0	0	0	0.5	1	0.75	0.75

For peer review only

#N/A	#N/A	#N/A	#N/A	#N/A	#N/A	#N/A	#N/A	#N/A
#N/A	#N/A	#N/A	#N/A	#N/A	#N/A	#N/A	#N/A	#N/A
1	0.5	1	0.5	1	1	1	1	0.75
1	1	1	0.5	0	1	0.5	1	0.75
1	1	1	1	1	0.5	1	0.75	0.75
1	1	1	0.5	0	1	0.5	1	0.75
1	1	1	1	1	0.5	1	0.75	0.75
1	1	0	0.5	0	1	1	1	1
0.5	0.5	0.5	1	1	1	0.5	0.75	1
1	0	0.5	0	0.5	0	0.5	0.75	0.75
0	0	0	0.5	0.5	1	1	1	0.75
1	1	0	0	1	1	1	1	0.75
1	1	1	0	1	1	1	1	1
1	0	1	1	1	0	1	0.75	0.25
1	1	1	1	1	1	1	1	1
1	0.5	0.5	0.5	0.5	0.5	0.5	0	0.75
1	0	0.5	0.5	0.5	0.5	0.5	0.75	0.75
0	0.5	0.5	1	1	1	1	0.75	0.75
1	1	1	1	1	1	1	1	1
1	1	1	1	1	0	1	1	0.75
1	1	1	1	1	1	1	1	0
1	1	1	1	1	1	1	1	0.75
1	1	1	1	1	1	1	1	0
1	1	1	1	1	1	1	1	0.75
0.5	1	1	1	1	1	1	0.75	0.75
1	0	0.5	0.5	0.5	0	1	1	0.75
0.5	1	0.5	1	1	1	1	1	1
1	0	1	1	1	1	1	1	0.75
1	0	0	0	0	0	0	0.75	0.25
1	0	0	0	0.5	1	0	1	0.25
0	0	0	1	1	0	1	1	0.75
0	1	0	0	0	0	0	0	0.75
1	1	1	0	1	1	1	1	1
1	1	0.5	1	1	1	0.5	0	0.75
1	1	1	1	1	1	1	1	1
1	1	1	1	1	1	0.5	0.75	1
1	1	0	0	0.5	0	1	0	0.75
1	0	0	1	1	0	1	1	0.75
1	1	1	1	1	1	1	1	0.75
0	0	0	0	0.5	0	1	1	0.75
0	0	0	0	0.5	0	1	1	0.75
1	1	1	1	1	1	1	1	0.75
1	1	0	0	1	0	1	1	0.75
0	1	0	0	0.5	0	1	1	0.75
1	1	0	0	0.5	0	1	1	0.75
1	1	0	1	1	0	1	0	0.75
0	0	0	0	0	0	0	0	0.75
0	0	0	0	0	0	0	0	0.75
0	0	0	0	0	0	0	0	0.75
1	0	1	1	0.5	0	1	1	0.75

For peer review only

0	1	0	0	0.5	1	1	1	0.75
0	0	0	0	0	0	1	0	0.75
0	1	0	0	0	1	1	1	0.75
0	0	0	0	0	0	1	1	0.75
0	0	0	0	0	0	0	0	0.75
0	0	0	0	0	1	1	1	0.75
0	0	0	0	0	0	0	0	0.75
1	1	1	1	1	1	1	1	0.75
1	1	1	1	1	0	1	1	0.75
0	1	0	0.5	0.5	0	1	0	0.75
0	0	0	0.5	0.5	0	0.5	0	0.75
0	0	0.5	0	0	1	0.5	0.75	0.75
0	0	0	0.5	0.5	0	0.5	0	0.75
0	0	0.5	0	0	1	0.5	0.75	0.75
1	1	1	1	1	1	1	1	0.75
0	0	0	0.5	0.5	0	1	0	0.75
0	0	0	1	0	0	1	0.25	0.75
0	1	0	0.5	0.5	0	0	0	0.75
0	0	0	0.5	0.5	0	1	0	0.75
1	1	1	1	1	1	1	1	1
1	1	1	1	0.5	0	1	1	1
1	1	0.5	1	1	1	1	0.75	0.75
0.5	1	0.5	0	1	1	1	0.75	1
1	1	1	1	1	1	1	1	1
1	1	1	1	1	0	0	0	0.75
1	1	0.5	1	1	1	1	1	1
1	0	1	0	0.5	0	1	0.75	0.75
1	1	1	0	1	1	1	1	1
1	1	0.5	1	1	1	1	1	1
1	1	1	1	1	0.5	0	1	0.75
1	1	0.5	0.5	0.5	1	1	0.25	0.75
0.5	0.5	0.5	0.5	0.5	0.5	0.5	0.75	0.75
0	1	0.5	0.5	0.5	0.5	0	0.25	0.75
1	1	0	1	1	1	1	0.75	0.75
1	1	1	0.5	0.5	0.5	1	1	1
1	1	0.5	0	0	0	1	0.75	0.75
1	1	0	0	0.5	1	1	1	0.75
1	1	1	1	1	0.5	1	1	1
1	1	0	0	0.5	0	1	0	0.75
0	1	0.5	0.5	0.5	0	1	0	0.75
1	1	1	1	1	1	1	1	1
1	0	0	0	0	0	0	0	0.75
1	1	0	1	1	1	1	1	0.75
1	1	1	1	0	1	1	1	1
1	1	0.5	1	1	1	1	0	0.75
1	1	1	1	1	1	1	0	0.75
1	1	0.5	1	1	1	1	0	0.75
1	1	1	1	1	1	1	0	0.75
1	1	1	1	1	1	1	1	0.75
1	1	0	0.5	0.5	0	1	0	0.75

For peer review only

1	1	0	0.5	0.5	0	1	0	0.75
1	1	1	0	1	1	1	0.75	0.75
1	1	1	1	1	1	1	1	0.75
0	0	0	0.5	0.5	0	1	0	0.75
1	1	1	1	1	1	1	1	0.75
0	0	0	0	0.5	1	1	0.25	0.75
1	0.5	1	1	1	1	0.5	0.25	0.75
1	1	1	1	0	1	1	1	0.75
1	1	1	1	1	1	1	1	0.75
0.5	0	0	0	1	0	0.5	0.75	0
1	0	0.5	0.5	1	0	1	1	0.75
1	0.5	1	1	1	1	1	0.75	0.75
1	1	1	1	1	1	1	1	1
1	1	1	1	1	1	1	1	1
1	1	1	1	1	1	1	1	0.75
1	1	0	0	0.5	1	1	1	0.75
1	1	0	0	0.5	1	1	1	0.75
0	1	0	1	1	1	1	1	1
0	0	0	0.5	0.5	0	1	0	0.75
0	1	0	0	0.5	0	1	0.25	0.75
1	1	0	0	0.5	0	1	0	0.75
1	1	1	1	1	1	1	1	1
1	1	1	1	1	0	1	1	0.75
1	0	1	1	0	1	1	1	1
0.5	1	0.5	0	0	1	1	1	0.75
1	1	1	1	1	1	1	1	0.75
0	0	0	0	0	0	0	0	0.75
1	1	0	0	1	0	1	0	0.75
1	0	0	0	0.5	0	1	0	0.75
1	1	0	1	1	0	1	0	0.75
0	1	1	1	1	1	1	1	0.75
1	1	1	1	1	0	1	1	0.75
1	1	1	1	0	1	1	0.75	0.75
1	0	0	1	1	1	1	1	1
0	0	1	0	0.5	0	1	0	0.75
1	1	0	0	0.5	1	1	1	0.75
0	0	0	0	0.5	0	1	1	0.75
0	1	0	0	0.5	0	1	0	1
0	1	0	0	0.5	0	1	1	0.75
1	0	0	0	1	0	1	1	0.75
0	1	0	1	1	1	1	1	0.75
1	1	0.5	0	0	0.5	0	0.25	0.25
1	1	0.5	1	1	1	1	1	1
1	1	1	1	1	0.5	1	0.75	0.75
1	1	1	1	1	1	1	1	0.75
1	0	0	1	1	0	0.5	0.75	0.25
1	1	0	1	1	0.5	0.5	0.75	0.75
0.5	0.5	0.5	0	0	1	0.5	0.75	0.75
1	0	0	1	0	1	0.5	0	0.75
0	1	0.5	1	1	1	0	0	0.75

For peer review only

0.5	1	0.5	1	1	1	0.5	0	0.75
0.5	0.5	0.5	1	1	1	0.5	0	0.75
1	1	1	1	1	1	1	0	0.75
1	1	0	1	0.5	0	1	1	0.75
1	0	0	0	0	0	1	0	0.75
1	1	1	1	1	1	1	1	0.75
1	0	0	0	0.5	0	1	0	0.75
0	0	0.5	0.5	0.5	0	0.5	0.75	0.75
1	1	1	1	1	1	1	1	1
1	1	1	1	1	1	1	0	0.75
0.5	1	0	0.5	0.5	0	0.5	1	0.75
0	1	0	0	0	0.5	1	0.75	0.25
1	1	1	1	0	1	0.5	0	0.25
1	1	1	1	1	1	1	0.75	0.75
1	1	0	0.5	0	0.5	0.5	0	0.25
1	1	0	0	0	0	0.5	0	0.25
1	1	1	1	1	1	1	1	1
1	1	0	1	1	1	1	1	0.75
1	0	1	0	1	0	0.5	0.75	0.75
0.5	0	0	1	1	0	1	1	0.75
1	1	1	1	1	1	1	1	1
1	1	1	1	1	1	1	1	1
1	1	1	1	1	1	1	1	1
1	0	0	0	0	0	0	1	0.25
0	1	0.5	1	1	0	1	1	0.75
1	1	1	1	1	1	1	1	0.75
1	1	1	1	0	1	1	1	0.75
0	0	0	0	0	0	1	0.75	0.75
1	1	1	1	1	1	1	1	0.75
1	1	0	1	1	1	1	1	0.75
0	0	0	0	1	0	1	0	0.75
1	1	1	1	1	1	1	1	0.75
1	0	0	0	0	0	1	0	0.75
1	1	1	1	1	0	1	1	0.75
0	1	0	0	0	0	1	0	0.75
0	0	0	0	0	0	1	0	0.75
1	1	0	1	1	0	1	0	0.75
1	1	0	1	1	0	1	0	0.75
0	0	0	0	0	0	1	0	0.75
0	0	0	0	0.5	0	1	0	0.75
0	0	0	0	0	0	0.5	0.75	0.75
1	0.5	0	0.5	0.5	0	1	1	0.25
1	0.5	1	1	1	1	0.5	1	0.75
1	1	1	1	1	1	1	1	1
1	1	1	1	1	1	1	1	1
1	1	1	1	1	0	1	1	0.75
1	1	1	1	1	1	1	0	1
1	1	1	1	1	1	1	0.75	0.75
1	1	0.5	1	0	0.5	0.5	0.75	0.75
1	1	1	1	1	1	1	1	1

For peer review only

1	1	1	1	1	1	0.5	1	1
0.5	0.5	1	1	1	1	0	0.75	0.75
1	1	1	1	1	1	1	1	0.75
1	1	1	1	1	1	1	0	1
1	0.5	0.5	1	1	1	0.5	0	0.75
1	0.5	0.5	1	1	1	0.5	0	0.75
1	1	0.5	1	1	1	1	1	0.75
1	1	0.5	1	1	1	0.5	0	0.75
1	1	1	0.5	0	0	0.5	0	0.25
1	0.5	0.5	1	1	1	0.5	0.75	1
1	1	0.5	1	1	1	1	0	1
1	1	1	1	1	1	1	1	1
1	1	1	1	1	1	1	0.75	0.75
0.5	0.5	0.5	1	1	1	0.5	0	0.75
1	1	1	1	1	1	0.5	0	0.75
1	1	0.5	0	0.5	1	1	1	0.75
0	1	0.5	0.5	0.5	0.5	0.5	1	0.75
1	1	1	1	1	1	1	1	1
1	1	0	1	1	0	1	0	0.75
1	1	1	1	1	1	1	0.75	1
0	1	0	0	0.5	0	1	0	0.75
1	0	1	0	0.5	0	1	0	0.75
0	1	0	0.5	0.5	0	1	1	0.75
1	1	0.5	0	0.5	1	1	1	0.75
1	1	0.5	0	0.5	1	1	1	0.75
0	0	0.5	0.5	0.5	0	1	0.75	0.75
0	0	0	0	0	0	0.5	0.75	0.75
1	0	1	1	1	1	1	1	0.75
1	1	1	1	1	1	1	0.75	0.75
1	1	1	0	1	1	1	1	0.75
0	1	0	1	0	1	1	1	0.75
1	1	0.5	0	1	1	1	1	0.75
1	0	0	1	1	1	1	1	0.75
0	0	0.5	0	0	0	0.5	0.75	0.75
0	0	0	0	0	0	0.5	1	0.75
0	0	0	0	0	0	1	1	0.75
1	1	0	1	0	0	0.5	1	0.25
1	1	1	1	0	0	1	1	1
1	1	0	0	0.5	0	1	0	0.75
0	0	1	1	1	1	1	1	0.75
0	0	1	0	1	1	1	1	1
1	1	0.5	0	0.5	0	1	1	1
0.5	0.5	0.5	0.5	0.5	0	0.5	0.75	0.75
1	1	1	1	1	1	1	1	1
1	1	1	1	1	1	1	1	1
1	1	1	1	1	1	0.5	0	1
1	1	1	1	1	0.5	1	1	0.75
1	0	0	1	1	0.5	1	1	0.75
1	1	1	1	1	1	1	0.25	1
1	1	0.5	0.5	1	1	1	0.75	1

For peer review only

1	1	1	1	1	1	1	1	0.75
1	0.5	1	1	1	1	1	1	0.75
1	1	1	0	0.5	0.5	0.5	0.75	0.75
0	0.5	0.5	1	1	1	0.5	0.75	0.75
1	0	0	0	0.5	0	0	1	0.75
0.5	0	0	0	0	0.5	0	0	0.75
0.5	0.5	1	1	1	1	0.5	1	0.75
1	1	1	1	1	1	1	1	1
1	1	1	1	1	1	1	1	1
1	0.5	0.5	0.5	0.5	0.5	0.5	1	0.75
1	1	1	0	1	1	1	1	1
1	1	0.5	1	1	1	1	1	1
0	1	0.5	1	1	0.5	1	1	0.75
1	0.5	1	0.5	1	1	0.5	0.75	0.75
1	1	0.5	1	1	1	1	1	1
0	0	1	0	0	0	1	1	0.75
1	1	1	1	1	1	1	0.75	0.75
1	0	0	0	0	0	1	1	0.75
1	0	0	0	0	0	0.5	0	0.75
1	1	0.5	0.5	1	0.5	1	1	0.75
0	1	0.5	0	0	0.5	0.5	0.25	0.75
0.5	0.5	0.5	1	1	1	0	1	0.75
1	1	1	1	1	1	1	1	1
1	1	1	1	1	1	1	1	1
0	0.5	1	0	1	1	0.5	0	0.75
1	1	0	1	1	0	0.5	0.75	0.75
0	0	0.5	1	1	1	0	0	0.75
0	0	1	1	1	1	0.5	0	1
1	1	1	1	1	0	1	1	1
1	1	0.5	0	0.5	0	0.5	1	0.25
1	1	1	1	1	1	0	1	0.75
1	1	0	1	1	1	1	1	0.75
1	1	1	1	1	1	0	1	0.75
1	1	0	1	1	1	1	1	0.25
1	1	1	1	1	1	1	0.75	1
1	1	1	1	1	1	0	0	0.25
1	1	0	1	1	1	1	1	0.75
1	0	0	1	1	0	0	0	0.25
1	1	1	1	1	1	0	0	0.75
1	1	0	1	1	1	1	0.75	0.25
1	1	1	1	1	1	1	1	1
1	0	0	1	1	0	1	1	0.75
1	1	1	1	1	1	1	1	0.75
0	1	0	0	0	0	1	0	0.75
0	0	1	0	1	1	1	1	0.75
1	1	1	1	1	1	1	1	1
1	1	1	1	1	1	1	1	0.75
1	1	0	0	1	1	1	1	0.75
1	1	1	0	0.5	1	1	1	0.75
1	0	0	1	1	0	1	1	0.75

For peer review only

OutcomeO	StratifiedVa	Assessment	AdequateF	SimilarCointerventions
1	0.25	0.25	0.75	0.75
1	0.25	0.25	0.75	0.25
1	0.75	0.75	0.75	1
0.75	0.25	0.25	0.25	0.25
0.75	0.25	0.25	0	0
0.75	0.25	0.25	0.75	0.75
0.75	0.25	0.75	0	0.75
0.25	0	0.25	1	0.25
0.25	0	0.25	0.75	0.75
0	1	1	1	0.75
0.75	0.75	0.25	0.75	0.75
1	0.75	0.75	0.75	0.75
1	0.25	0.25	0.75	0.75
0.75	0.25	0.75	0.75	0.75
0.75	0.25	0.25	0.75	0.75
0.25	0.25	0	0.25	0.75
0.75	0.25	0.75	0.75	0.75
0.75	0.75	0.75	0.75	0
0.75	0.25	0.25	0.75	0.75
0.75	0.75	0.75	0.25	0.75
0.75	0	0.75	0.75	0.75
0.75	0.75	0.75	0.75	1
1	0.75	0.75	0.75	0.75
1	0.75	0.75	0.75	0.75
0.75	0.75	0.75	0.75	0.75
0.25	0.75	0.75	0.75	1
0.75	0	0.25	0.25	0.25
1	0	0.75	0.75	0.75
1	1	0.75	0	0.75
0.75	1	0.75	0.25	0.75
0.75	0	0.75	0	0.75
1	1	0.75	0	0.75
1	0	0.25	0.75	0.25
1	1	0.75	0	0.75
1	1	0.75	0	0.75
1	1	0.75	0	0.75
1	0	0.75	0.25	0.75
1	1	0.75	0.75	0.75
1	1	0.75	0.75	0.75
1	1	0.75	0	0.75
1	1	0.75	0	0.75
0.75	1	0.75	0	0.75
1	0	0.75	0	0.75
1	1	0.75	0	0.75
0.75	0	0.25	0	0
0.75	1	0.75	0	0.75
1	1	0.75	0	0.75
1	0	0.75	1	0.75
0.75	0	0.25	0	0.75

For peer review only

1	0	0.75	0	0.75
1	0	0.75	0.75	0
1	0	0.75	0.75	0
1	1	0.75	0	0.75
1	0.25	0.75	0	0.75
0.75	1	0.75	0	0.75
0.75	0	0.75	0	0
0.75	1	0.75	0.25	0.75
0.75	1	0.75	1	0.75
1	1	0.75	1	0.75
0.75	1	0.75	0	0.75
1	0	0.25	0	0
1	1	0.75	0	0.75
1	1	0.75	0	0.75
1	1	0.75	0	0.75
0.75	1	0.75	0	0.75
0.75	0	0.75	1	0.75
0.75	1	0.75	0	0.75
0.75	1	0.75	0	0.75
1	1	0.75	0	0.75
1	0	0.25	0	0
1	1	0.75	0.25	0.75
0.75	0	0.75	0	0.75
0.75	0	0.25	0	0
0.75	0	0.25	0.25	0.75
0.75	0	0.25	0.75	0
0.75	0	0.75	0.75	0.75
0.75	0	0.25	0.25	0
0.75	1	0.75	0	0.75
0.75	0	0.75	0.75	0.75
0.25	0	0.25	0.75	0.75
0.75	1	0.75	0	0.75
0.75	1	0.75	0.25	0.75
0.75	1	0.75	1	0.75
1	0	0.25	0.25	0
1	0	0.75	0.25	0.75
1	0	0.75	0.25	0
1	0.25	0	0.25	0.25
0.75	0.25	0.75	0.25	0.25
0.75	0.25	0.75	0	0.75
0.75	0.75	0.25	0.25	0.25
0.75	0	0.25	0.75	0.25
0.75	0.25	0.25	0.75	0.25
1	1	0.75	0.75	0.75
0.75	0	0.75	1	0.75
0.75	0	0.25	0.75	0.25
0.75	0.25	0.25	0.25	0.75
1	1	0.75	0.75	0.75
0.75	0	0.25	0.75	0.75
1	0	0.25	0.75	0.75

For peer review only

1	1	0.75	0.75	0.75
1	0	0	0.75	0.75
1	0	0.25	0.75	0.75
1	0	0.25	0.75	0.75
0.75	0.75	0.75	0.75	0.75
0.75	1	0.75	0.75	1
0.75	0.75	0.75	0.75	0.75
0.75	1	0.75	0.75	1
0.75	0.75	0.75	0.75	0.75
0.75	1	0.75	0.75	1
0.75	0.75	0.75	0.75	0.75
0.75	1	0.75	0.75	1
0.75	0.75	0.75	0.75	0.75
0.75	0.75	0.75	1	1
0.75	0.75	0.75	1	0.25
1	1	1	1	1
0.75	0.25	0.75	0.75	0
0.75	0.75	0.75	1	0.75
0.75	0.75	0.75	0.75	0.75
0.75	0.75	0.75	0.75	0.75
0.75	0	0	0.75	0
1	1	1	1	1
1	1	1	1	1
1	1	1	1	1
1	0.75	0	0	0.25
0.75	0.25	0.75	1	0.75
0.75	0.25	0.75	0.75	0.75
0.75	1	0.75	0.75	1
0.75	0.75	0.75	0.75	0.75
0.75	0.75	0.25	1	0
0.75	0.75	0.75	0.75	0.75
0.75	0.75	0.75	0.75	0.75
0.75	0.75	0.75	0.75	0.75
0.75	0.75	0.75	0.75	0
1	1	0.75	1	0
1	1	1	1	1
0.75	0.75	0.75	0.75	0
0.75	0.75	0.75	0.75	1
0	0	0	0.75	0.75
1	1	1	0.75	0.75
1	1	0.75	1	1
1	1	0.75	0.75	0.75
1	1	0.75	0.75	0.75
1	1	1	0.75	0.75
1	0	0.25	0.75	0.75
1	1	1	0.75	1
1	0	0	0.75	0.75
1	0	0.25	0	0
0.75	0	0	0.25	0.25
1	1	0.75	0.75	0.75

For peer review only

1	0	0.75	0.75	0.75
1	0	0.75	0.75	0.75
1	1	0.75	0	0.25
0.75	0	0	0.25	0
1	0	0.75	0.75	0.75
1	1	0.75	0.25	0.75
0.75	0	0	1	0
1	0	0	0.75	0
1	1	0.25	0.75	0.75
1	1	0.75	0.75	1
1	0	0.75	0.75	0.75
1	0	0.25	0	0
1	1	0.75	1	0.75
1	0	0	0.75	0
1	1	1	0	0.75
1	0.25	0	0	0
1	1	0.75	0	0.75
1	1	0.75	1	0.75
1	1	0.75	1	0.75
1	1	0.75	0.75	1
1	1	0.75	0.25	0.75
0.25	0.25	0.75	0.75	1
1	0.25	0.25	0.75	0
0.75	0.75	0.75	0.75	1
1	1	1	1	1
0.75	0.25	0.25	0.75	0.25
0.75	0.25	0.75	0.75	0.75
1	1	0.25	0.75	0.25
0.75	0.75	0.75	0.75	0
0.75	0.75	0.75	0.75	0
0.75	0.25	0.75	0.75	1
0.75	0.75	0.75	0.75	0.75
1	1	0.75	1	1
0.75	0.75	0.25	0.75	0.75
0.75	0.75	0.75	0.75	0.75
1	1	1	1	1
0.75	1	0.75	0.75	1
0.75	0.75	0.75	0.75	0.75
0.75	0.25	0.25	0.25	0.25
0	0.25	0.25	0.25	0.75
0.75	0	0	0.25	0.25
0.75	0.75	0.25	0.75	0
0.75	0.25	0.25	0.25	0.25
1	1	1	0.75	1
0.75	0.75	0.75	0.75	1
0.25	0	0	0.75	0.25
0.75	0.75	0.75	1	1
0.25	0.25	0.25	0.75	0.25
1	1	1	1	0
0.75	0.25	0.25	0.75	1

For peer review only

0.75	0.25	0.25	0.25	0.75
1	1	1	1	1
0.75	0.75	0.75	0.75	1
0.75	0.25	0.75	0.75	0.75
0.75	0.75	0.75	0.75	0
0.75	0.25	0.75	0.75	0.75
1	0.75	0.75	0.75	1
1	1	1	1	1
0.75	1	0.25	0.25	0.25
0	0.25	0	0	0.75
0.25	0.75	0.75	1	0
0.75	0.75	0.75	0.75	0
0.75	0.25	0.75	0.75	0.75
0.75	0.25	0	0	0
0.75	0.25	0.75	0.75	1
0.25	0.75	0.25	0.75	0.75
1	0.75	0.75	0.75	0.75
1	0.75	0.75	0.75	1
0.75	0.25	0.75	0.75	1
1	0	0.75	0.75	0.75
1	1	0.75	0.75	0.75
0.75	0.75	0.75	0.75	0
0.75	0	0	0	0.75
1	0.75	0.75	0.75	0.75
1	0	0.25	0.25	0
0.75	0	0	0	0
0.25	0	0	0.75	0
0.75	0	0.25	0.25	0.25
1	0	0	0.75	0
0.25	1	0.25	0.75	0.75
1	0	0.75	0.75	0.75
1	0	0.75	0.75	0.75
0.75	0.25	0.75	0.75	0.75
1	0	0.75	0.75	0.75
1	0	0.75	0.75	0.75
0.75	0.25	0.75	0.75	0.25
0.75	0.75	0.75	0.25	0
0.75	0.75	0.75	0.75	0.75
0.75	0.25	0.25	0.75	0.25
0.75	0	0	0.25	0.75
0.75	0.75	0.75	0.75	1
0.75	0.75	0.75	0.75	1
1	1	0.75	1	1
0.75	0.25	0.75	0.75	1
0.75	0.25	0.25	0.75	0.75
0.25	0.25	0.75	0.75	1
0	0.75	0	0.25	0
0.75	0.25	0.25	0.75	0.75
0.75	0.25	0.75	0.75	1
0.75	0.25	0.75	0.75	1

For peer review only

#N/A	#N/A	#N/A	#N/A	#N/A
#N/A	#N/A	#N/A	#N/A	#N/A
1	1	1	0.75	0.75
1	1	1	0.75	0.75
0.75	0.75	0.75	0.75	0.75
1	1	1	0.75	0.75
0.75	0.75	0.75	0.75	0.75
0.75	0.25	0.75	0.75	1
0.75	0.75	0.75	0.25	0.75
0.75	0.25	0.75	0.75	0.75
0.75	0	0.25	0.75	0.75
1	1	0.75	0.75	0.75
1	1	1	1	1
0.75	0.25	0.25	0.25	0
1	1	1	1	1
0.75	0.25	0.75	0.75	0.75
0.75	0.25	0.75	1	0.25
0.75	0.75	0.75	0.75	0.75
1	1	1	1	1
1	0.75	0.25	0.75	0.75
1	0.25	0.25	0.75	0.25
1	0.75	0.75	1	1
1	0.25	0.25	0.75	0.25
1	0.75	0.75	1	1
1	0.75	0.75	0.75	1
1	1	1	0.25	0.75
1	0.75	0.75	1	1
1	0	0.25	0.75	0.75
0.75	0	0	0.25	0.25
1	0	0.75	0.75	0.75
1	0	0.25	0.75	0.75
0.75	0	0	0	0
1	1	1	0.75	1
0.75	0.75	0.75	0.75	0
1	1	1	1	1
1	1	1	0.75	0.25
1	0	0	0.75	0
0.75	0	0.75	0	0.75
1	0.75	0.75	1	0.75
1	1	0.75	0	0.75
0.75	1	0.25	0	0.75
1	1	0.75	1	0.75
1	1	0.75	0.25	0.75
0.75	1	0.75	0	0.75
1	0	0.75	0	0.75
1	0	0.25	0.75	0.75
0.75	0	0	0	0
0.75	0	0	0	0.75
0	0	0	0	0.75
1	1	0.75	0	0.75

For peer review only

0.75	0	0.75	0	0.75
0.25	0	0.25	0.25	0
0.75	1	0.75	0	0.75
0.25	0	0.75	0	0.75
0	0	0	0	0
0.75	0	0.75	0	0.75
0	0	0	0	0.25
0.75	1	0.75	0	0.75
0.75	1	1	0.75	1
0.75	0	0.25	0.75	0
0.75	0	0	0.75	0
0.75	0.25	0.25	0.25	1
0.75	0	0	0.75	0
0.75	0.25	0.25	0.25	1
1	1	0.75	0.75	0.75
0.75	0	0	0.75	0
0.75	0.25	0.25	0.75	0.25
1	0	0.25	0.75	0.75
0.75	0	0.25	0.75	0
1	1	1	1	1
1	0.25	0.75	0.75	0.75
0.75	0.75	0.75	0.75	1
1	0.75	0.75	0.75	1
1	1	1	1	1
0.25	0.75	0.25	0.75	0
0.75	0.75	0.75	0.75	1
0.75	0	0.25	0.75	0.75
0.75	0.75	1	0.75	1
1	1	1	0.75	1
1	0	0.75	0.75	0.75
1	0	0.25	0.75	0.75
0.75	0.75	0.75	0.75	1
0.25	0.25	0.25	0.75	0.75
0.75	0.25	0.75	0.75	0.75
1	0	0.25	0.75	0.75
1	0.75	0.75	1	0.75
1	0	0.75	0.75	0.75
0.75	0.75	0.25	0.75	0.75
0.75	0	0.25	0.75	0
1	0	0	0.75	0
1	1	1	0.75	1
0.75	0	0.25	0.25	0
1	0	0.75	0.75	0.75
1	0	0.75	0.75	0.75
0.75	0.75	0.75	0.75	0
0.75	1	1	1	0
0.75	0.75	0.75	0.75	0
0.75	1	1	1	0
1	0	0.75	0.75	0.75
0.75	0	0.75	0.75	0

1	0	0	0.75	0
0.75	0.75	0.75	1	1
1	1	0.75	0.75	0.75
1	0	0	0.75	0
1	0.25	0.75	0.75	0.75
0.75	0	0.25	0.75	0.75
0.75	0.25	0.25	0.75	0.75
1	0	0.75	0.75	0.75
1	0	0.75	0.75	0.75
1	0.25	0.25	0.75	0.75
1	0	0.75	0.75	0.75
1	0.75	0.75	0.25	1
1	1	1	1	1
1	1	1	1	1
1	0	0.75	0.75	0.75
1	0	0.75	0	0.75
1	0	0.25	0	0.75
0.75	1	1	0	0.75
1	0	0.75	0.75	0.75
0.75	0	0	0.25	0.25
1	0	0.25	0	0
1	1	1	0.75	0.75
1	0.25	0.75	1	0.75
0.75	0	0.75	0	0.75
1	0	0.75	0.75	0.75
1	0	0.75	1	0.75
0.75	0	0	0	0
0.75	0	0.75	0.75	0.75
0.75	0	0.25	0.75	0
0.75	0	0.25	1	0
1	1	0.75	0	0.75
1	0	0.75	0.25	0.75
0.75	0.25	0.75	0.75	0.75
1	1	0.75	0	0.75
1	0	0	0	0
1	1	0.75	0	0.75
1	1	0.75	0	0.75
0.75	0	0	0	0.25
0.75	1	0.75	0	0.75
0.75	1	0.75	0	0.75
1	1	0.75	0.25	0.75
0.75	0	0.25	0.25	0.25
0.75	1	1	1	1
1	0.75	0.75	0.75	1
0.75	1	1	1	1
0.75	0	0	0.75	0.25
1	0	0.25	0.25	0.25
0.75	0.25	0.75	0.75	0.75
0.75	0.25	0.25	0.75	0
0.75	0.75	0.75	0.75	0

For peer review only

1	0.75	0.75	0.25	0
0.75	0.75	0.75	0.75	0
1	1	1	0.75	0
0.75	0	0.75	0.75	0.75
1	0	0.25	0.75	0.75
1	1	1	0.75	0.75
0.75	0	0	0.75	0.75
0.75	0.25	0.75	0.75	0.25
1	0.75	0.75	0.75	1
1	1	1	0.75	0
0.75	0	0.75	0.75	0.25
0.75	0	0.25	0.75	0.25
1	1	0.25	0.75	0.25
1	1	1	0.75	1
0.75	0.25	0.25	0.75	0
0.75	0	0.25	0.75	0.25
1	1	1	1	1
0.75	0.75	0.25	0.25	0.25
0.75	0.25	0.25	0.75	0.75
0.75	0.75	0.75	0.75	1
1	1	1	0.75	1
1	1	1	1	1
1	1	1	1	1
0.75	0	0	0.25	0.25
1	0	0.75	0	0.75
1	1	0.75	1	0.75
1	1	0.75	1	0.75
0.75	0	0.75	0	0.75
1	1	0.75	0	0.75
1	0	0.75	0.75	0.75
1	0	0.25	0.75	0
1	1	0.75	1	0.75
1	0	0.25	1	0
1	0.25	0.75	1	0.75
1	0	0	0.25	0
0.75	0	0	0.75	0
1	0	0	0	0
1	0	0.25	0.75	0
1	0	0	1	0
1	0	0.75	0.75	0
1	0.75	0.25	0.75	0.75
0.75	0.75	0.75	0.75	0.75
0.75	0.25	0.75	0.75	1
1	1	1	0.75	1
1	1	1	1	1
0.75	0.75	0.75	0.75	1
1	1	1	1	0
0.75	0.75	0.75	0.75	0.75
0.75	0.25	0.25	0.75	0.25
1	1	1	0.75	1

For peer review only

1	1	0.75	0.75	1
0.75	0.25	0.25	0.25	0.75
1	1	1	1	1
0.75	0.75	0.75	0.75	0
0.25	0.75	0.75	0.75	0
0.75	0.75	0.75	0.25	0
0.75	0.25	0.75	0.75	1
0.75	0.75	0.75	0.75	0
0.75	0.25	0.25	0.25	0.25
0.75	0.75	0.75	0.25	1
0.75	0.75	0.75	0.75	0
1	1	1	1	1
0.75	0.75	0.75	0.75	1
0.75	0.75	0.75	0.75	0
1	0.75	0.75	1	0
0.75	0.75	0.75	0.75	0.75
0.75	0.75	0.75	0.75	0.75
1	1	1	0.75	1
0.75	0	0.75	0.75	0.25
1	1	1	1	1
0.25	0	0.25	0.75	0.75
0.75	0	0.75	0.75	0.75
1	0	0.75	0.75	0.75
1	0	0.75	0.25	0.75
1	1	0.75	1	0.75
0.75	0.25	0.75	0.75	0.25
1	0	0.75	0.25	0.25
1	0.25	0.75	0.75	0.75
1	0.75	0.75	0.75	0.75
1	0	0.75	0.75	0.75
0.75	1	0.75	0	0.75
1	0	0.75	0	0.75
1	0	0.75	0.75	0.75
0.75	0.75	0.75	0.25	0.75
0.75	0.25	0.75	0.75	1
1	0	0.75	0.25	0.75
0.25	0	0.25	0.25	0.25
1	0	0.75	1	0.75
1	1	0.75	1	0
1	1	0.75	1	0.75
1	1	0.75	1	0.75
1	0.75	0.75	0.75	0.75
0.75	0.25	0.75	0.75	0.75
1	1	1	0.75	1
1	1	1	1	1
1	0.75	0.75	0.75	0
0.75	0.25	0.75	0.75	1
0.75	0.25	0.25	0.25	1
1	1	1	1	0.75
0.75	0.75	0.75	0.75	1

For peer review only

0.75	0.75	0.75	1	1
0.75	0.75	0.75	0.75	1
0.75	0.75	0.75	0.25	0.75
1	0.75	0.75	0.75	0.75
1	1	0	0.75	1
0.75	0.75	0.75	0.75	0
1	1	0.75	0.75	1
0.75	0.75	1	0.75	1
0.75	1	1	1	1
0.25	0.75	0.75	0.25	0.75
1	1	1	1	1
1	1	0.75	0.75	1
1	1	0.75	0.75	1
0.75	0.75	0.75	0.75	0.75
1	1	1	0.25	1
1	1	0.75	0	0.75
1	1	0.75	0.75	0.25
1	1	0.25	0.75	0.75
0.75	0	0.75	0.75	0.75
1	0.25	0.25	0.25	0.25
0.75	0	0	0.25	0.25
0.75	0.25	0.25	0.75	0.75
1	1	1	1	1
1	1	1	1	1
0.75	0.75	0.75	0.25	0
0.75	0.25	0.25	0.25	0.25
0.75	0.75	0.75	0.75	0
0.75	0.75	0.75	0.75	0
1	1	1	1	1
0.75	0	0.25	0.75	0.25
0.75	1	0.25	1	1
1	1	1	0.75	0.75
0.75	0.75	0.75	0	0.25
0	1	0.75	0.75	1
1	1	1	1	1
0.75	0	0	0.25	0
1	1	1	0.25	1
0.75	0	0	0.25	0
0.75	0	0	1	0
1	1	1	0.75	0.75
1	1	1	0.75	0.75
0.75	0	0.75	0.75	0.75
1	0	0.75	0.75	0.75
0.25	0.75	0.25	0.25	0.75
1	0	0.75	0.75	0.75
1	0	0.75	0.75	0.75
1	0	0.25	1	0.75
1	1	0.75	0.75	0.75
1	1	0.75	0.75	0.75
1	0	0.25	0.75	0.75

For peer review only

PublicationID	Author-Year	Drug	Animal	Number of animals in treatment group
21329	Bordet 2007	Cholest-4-en-3-one Oxime	Mouse	7
21846	Bordet 2001	AAV-CT-1	Mouse	46
21790	Koh 2007	GSK-3beta inhibitor	Mouse	14
21372	Koh 2006	Epigallocatechin gallate	Mouse	11
21391	Koh 2007	EPO	Mouse	24
21447	Mancuso 2014	Resveratrol	Mouse	23
21449	Mancuso 2012	PRE-084	Mouse	9
21705	Mancuso 2014	Resveratrol	Mouse	11
21444	Wagner 2015	anle138b	Mouse	37
21897	Hashimoto 2011	Metallothionein-III	Mouse	7
21857	Keller 2011	Minocycline	Mouse	16
21514	Kelly 2015	JAK4D	Mouse	13
21621	Azzouz 2000	AAV-Bcl2	Mouse	7
21626	Battaglia 2015	LY379268	Mouse	10
21854	Fornai 2008	Lithium	Mouse	10
21471	Raman 2004	THC	Mouse	9
21569	Kira 2006	acetyl-L-carnitine	Mouse	10
21763	Gill 2009	Lithium	Mouse	28
21489	Azari 2003	LIF	Mouse	9
21529	Azari 2005	Rofecoxib	Mouse	6
21767	Ohta 2008	TAT-BH4	Mouse	21
21523	Teng 2006	Clenbuterol	Mouse	12
21605	Butovsky 2015	anti-miR155-LNA	Mouse	11
21794	Butovsky 2012	Anti-Ly6C	Mouse	11
21689	Huang 2006	Bone marrow containing all cell types	Mouse	25
21738	Roberts 2014	Cu (atsm)	Mouse	8
21507	Jung 2001	EUK-8	Mouse	11
21859	Borel 2016	AAV9-SOD1	Mouse	15
21456	Uccelli 2012	MSCs	Mouse	12
21762	Guo 2015	Trehalose	Mouse	15
21877	Guo 2011	Diallyl trisulphide	Mouse	20
21326	Angelov 2003	Copaxone	Mouse	15

For peer review only

21955	Snyder 2014	Ferritin	Mouse	6
21915	Gerber 2013	Gacyclidine	Mouse	15
21520	Zhou 2013	Bone marrow mononuclear cells	Mouse	27
21966	Zhou 2013	MSCs	Mouse	15
21478	Esposito 2007	Pyruvate	Mouse	8
21335	Takata 2013	Fasudil	Mouse	13
21415	Podulso 2000	Polyamine-modified Catalase	Mouse	24
21355	Zheng 2007	VEGF	Mouse	4
21787	Zheng 2004	VEGF	Mouse	5
21345	Patel 2010	Caloric restriction	Mouse	31
21624	Patel 2015	Withaferin A	Mouse	12
21589	Patel 2014	AAV9-SOD1	Mouse	16
21542	Solomon 2011	Vitamin D deficiency	Mouse	47
21490	Jeong 2009	Salicylaldehyde isonicotinoyl hydrazone	Mouse	12
21392	Kong 2014	LDN/OSU-0212320	Mouse	23
21454	Takeuchi 2011	INI-0602	Mouse	24
21745	Takeuchi 2010	apo-SOD1 immunization	Mouse	9
21847	Ng 2015	KW-6002	Mouse	18
21585	Weishaupt 2006	Melatonin	Mouse	25
21650	Hwang 2009	NSCs	Mouse	10
21736	Kim 2013	Progesterone	Mouse	15
21377	Kim 2014	Cortical stimulation	Mouse	6
21369	Kim 2010	MSCs	Mouse	28
21796	Kim 2006	Cannabidiol	Mouse	6
21931	Kim 2014	Stem cells	Mouse	9
21575	Zhang 2008	Folic Acid	Mouse	9
21965	Zhang 2011	Rapamycin	Mouse	10
21779	Zhang 2003	Minocycline	Mouse	10
21852	Zhang 2013	Melatonin	Mouse	15
21811	Zhang 2009	MSCs	Mouse	21
21800	Trieu 1999	genistein	Mouse	13
21613	Trieu 2000	JAK3 inhibitor	Mouse	28
21322	Bruce 2004	Vincristine	Mouse	6

For peer review only

21577	Han 2012	Resveratrol	Mouse	16
21588	Ryu 2005	Sodium phenylbutyrate	Mouse	20
21552	Andreassen 2000	N-acetyl cystine	Mouse	12
21448	Andreassen 2001	Trientine	Mouse	21
21494	Andreassen 2001	Creatine	Mouse	12
21357	Klivenyi 2004	Creatine	Mouse	11
21550	Woodruff 2008	PMX205	Rat	5
21515	Yang 2010	Bee venom	Mouse	11
21339	Yang 2013	Kenpaullone	iPSC	384
21411	Yang 2011	Bee venom	Mouse	11
21346	Jiang 2000	Ginseng	Mouse	12
21625	Jiang 2014	Guanabenz	Mouse	15
21833	Ono 2014	SA 4503	Mouse	13
21872	Liu 2012	SOD1 Immunization	Mouse	10
21548	Liu 2002	DMPO	Mouse	16
21606	Thomsen 2014	AAV9-SOD1	Rat	14
21747	Chen 2015	AK-7	Mouse	27
21704	Chen 2012	Compound 13 (aryloxanyl pyrazolone)	Mouse	20
21879	Chen 2015	HDAC 6	Mouse	10
21403	Seo 2011	SK-PC-B70M	Mouse	9
21914	Ferrante 2001	Ginkgo biloba extract EGb 761	Mouse	10
21917	Groeneveld 2004	CGP 3466B	Mouse	7
21600	Groeneveld 2003	Zinc	Mouse	11
21361	Nolan 2014	miR-29a Antagomir	Mouse	20
21699	Martin 2007	NSCs	Mouse	15
21938	Martin 2014	GNX-4728	Mouse	12
21754	Kondo 2014	NSCs	Mouse	21
21807	Zhao 2007	MSCs	Mouse	29
21892	Zhao 2012	Caprylic triglyceride	Mouse	18
21429	Zhao 2015	High Fat diet	Mouse	10
21393	Chiba 2006	Colivelin	Mouse	9
21620	Chiba 2004	ADNF	Mouse	12
21909	Cheng 2015	Rapamycin	Drosophila	200

For peer review only

21672	West 2004	NDGA	Mouse	16
21388	Rabinowitz 2012	BBS2	Mouse	16
21484	Choi 2008	Estrogen	Mouse	35
21570	Yip 2013	Omega 3	Mouse	16
21488	Park 2007	Pyruvate	Mouse	19
21566	Ahn 2012	GSK-3beta inhibitor	Mouse	20
21748	Ahn 2014	GSK-3beta inhibitor	Mouse	31
21756	Wang 2008	siRNA SOD-1	Mouse	14
21652	Wang 2011	M30	Mouse	8
21635	Wang 2002	AAV-GDNF	Mouse	8
21557	Wang 2007	Nortriptyline	Mouse	12
21497	Wang 2014	Guanabenz	Mouse	22
21474	Wang 2005	Memantine	Mouse	10
21717	Morita 2008	MSCs	Mouse	13
21906	Castillo 2013	Trehalose	Mouse	17
21880	Sun 2014	MSCs	Mouse	12
21364	Morrison 2009	ActRIIB.mFc	Mouse	18
21900	Gurney 1996	Gabapentin	Mouse	9
21900	Gurney 1996	Gabapentin	Mouse	13
21380	Keep 2001	Ciclosporin	Mouse	6
21457	Kirkinezos 2003	Exercise	Mouse	30
21376	Sugai 2004	Valproate	Mouse	9
21841	CROCHEMORE 20(	Valproate	Mouse	6
21813	Rouaux 2007	Valproate	Mouse	15
21701	Weydt 2005	Cannabidiol	Mouse	8
21618	Kriz 2002	Minocycline	Mouse	17
21659	Kiaei 2006	Thalidomide	Mouse	12
21579	Kiaei 2005	Sulindac Sulfide	Mouse	12
21539	Kiaei 2005	Pioglitazone	Mouse	18
21546	Kiaei 2005	Celastrol	Mouse	14
21316	Dodge 2010	IGF-1	Mouse	25
21867	Dodge 2015	GENZ-667161	Mouse	9
21776	Dodge 2008	IGF-1	Mouse	10

For peer review only

21330	Corti 2007	NSCs	Mouse	6
21374	Corti 2004	Bone marrow containing all cell types	Mouse	12
21814	Corti 2010	bone marrow derived cells	Mouse	24
21510	Nagano 2005	IGF-1	Mouse	6
21684	Saenger 2012	IGF-1	Mouse	15
21684	Saenger 2012	IGF-1	Mouse	7
21318	Yoo 2011	Trichostatin A	Mouse	18
21399	Yoo 2012	Dihydrotestosterone	Mouse	18
21395	Schutz 2005	Pioglitazone	Mouse	7
21591	Williams 2016	Cu (atsm)	Mouse	20
21669	Tanaka 2008	L-745,870	Mouse	17
21400	Tanaka 2014	WN1316	Mouse	26
21426	Tanaka 2011	Bromocriptine	Mouse	38
21505	Suzuki 2008	MSCs	Rat	9
21927	Karlsson 2004	Ciclosporin	Mouse	7
21967	Zhu 2002	Minocycline	Mouse	10
21602	Xu 2006	NSCs	Rat	16
21389	Xu 2006	Epigallocatechin gallate	Mouse	8
21375	Xu 2011	NSCs	Rat	11
21525	Mahoney 2004	Exercise	Mouse	14
21646	Wu 2009	siRNA SOD-1	Mouse	6
21387	Wu 2003	Iron porphyrin	Mouse	19
21455	Feng 2012	DL-3n-butylphthalide	Mouse	11
21871	Frantz 2009	IGF-1	Rat	8
21674	Grignaschi 2007	EPO	Mouse	11
21368	Lee 2014	NSCs	Mouse	8
21885	Lee 2009	Arginine	Mouse	20
21802	Lepore 2007	IGF-1	Mouse	9
21528	Lepore 2008	Astrocytes	Rat	10
21710	Li 2012	Exendin-4 (Ex4)	Mouse	10
21382	Li 2015	N-acetyl-L-tryptophan	Mouse	30
21310	Li 2015	GLT1 overexpression	Mouse	7
21861	Li 2011	OECs only	Rat	20

For peer review only

21935	Li 2013	OECs only	Rat	24
21479	Matthews 1998	Coenzyme Q10	Mouse	16
21655	Smith 2006	siRNA SOD-1	Mouse	12
21670	Song 2013	Galactooligosaccharide	Mouse	12
21421	Song 2014	Resveratrol	Mouse	10
21341	Thompson 2012	D-serine	Mouse	7
21341	Thompson 2012	D-serine	Mouse	10
21438	Turner 2003	PNA	Mouse	6
21464	Turner 2004	p75NTR	Mouse	6
21960	Turner 2003	5-Hydroxy-L-Tryptophan (5-HTP)	Mouse	6
21752	Turner 2003	Clozapine	Mouse	8
21466	Bhattacharya 2012	Rapamycin	Mouse	6
21503	Chang 2005	Galectin-1	Mouse	14
21918	Gunther 2014	Y - 27632	Mouse	13
21918	Gunther 2014	Y - 27632	Mouse	13
21898	Aoki 2011	Edavarone	Rat	7
21592	Kanno 2012	CPN-9	Mouse	35
21698	Nanou 2013	NRF2 upregulation	Mouse	15
21584	Neymotin 2011	CDDO-TFEA	Mouse	18
21442	Neymotin 2009	Lenalidomide	Mouse	18
21311	Coughlan 2015	Latrepirdine	Mouse	24
21317	Beers 2011	Activated regulatory T lymphocytes	Mouse	10
21730	Van Den Bosch 20[illegible]	Minocycline	Mouse	7
21313	Van Damme 2003	NBQX	Mouse	15
21314	Saxena 2013	Rapamycin	Mouse	3
21331	Kaspar 2003	IGF-1	Mouse	25
21331	Kaspar 2003	IGF-1	Mouse	25
21422	Kaspar 2005	IGF-1	Mouse	18
21320	Nicaise 2008	Gemals	Rat	11
21321	Ruban 2009	OxAc	Mouse	9
21323	Bame 2012	Methionine	Mouse	50
21333	Staats 2012	Dantrolene Sodium	Mouse	19
21334	Dardiotis 2013	Melatonin	Mouse	14

For peer review only

21336	Vaknin 2011	IL-4	Mouse	13
21336	Vaknin 2011	IL-4	Mouse	14
21338	Barbeito 2010	Lead acetate	Mouse	7
21342	Locatelli 2007	siRNA Fas	Mouse	12
21351	Moreno-Igoa 2010	Fragment C tetanus toxin	Mouse	20
21352	Amante 2010	Uridine	Mouse	10
21354	Kieran 2008	Angiogenin	Mouse	12
21928	Kieran 2004	Acrimolol	Mouse	5
21356	Shimazawa 2010	SUN N8075	Mouse	6
21358	Hayes-Punzo 2012	DHEA	Rat	11
21366	Caraganis 2008	Thrombopoietin	Mouse	18
21379	Feeney 2003	LIF	Mouse	7
21381	McAllum 2015	Zinc	Mouse	16
21709	McAllum 2013	Cu (atsm)	Mouse	12
21385	Bronovitsky 2015	Dimebon	Mouse	18
21385	Bronovitsky 2015	Dimebon	Mouse	27
21396	Kupershmidt 2009	M30	Mouse	13
21397	Suchy 2010	S-adenosylmethionine	Mouse	12
21402	Knippenberg 2012	Umbilical Cord Blood Cells	Mouse	18
21476	Knippenberg 2013	Choline	Mouse	17
21506	Petri 2006	SS-31	Mouse	30
21695	Petri 2007	DP-109	Mouse	30
21769	Petri 2006	Sodium phenylbutyrate	Mouse	14
21404	Jaarsma 1998	N-acetyl cystine	Mouse	14
21405	Apolloni 2016	Clemastine	Mouse	16
21642	Apolloni 2014	BBG	Mouse	16
21406	Foust 2013	AAV9-SOD1	Mouse	6
21406	Foust 2013	AAV9-SOD1	Mouse	9
21406	Foust 2013	AAV9-SOD1	Mouse	5
21413	Garbuzova-Davis 2	hNT cells	Mouse	7
21458	Garbuzova-Davis 2	Umbilical Cord Blood Cells	Mouse	28
21458	Garbuzova-Davis 2	Umbilical Cord Blood Cells	Mouse	27
21761	Garbuzova-Davis 2	Umbilical Cord Blood Cells	Mouse	12

For peer review only

21417	Towne 2011	AAV9-SOD1	Mouse	8
21601	Towne 2008	AAV9-SOD1	Mouse	10
21423	Crow 2005	Manganese	Mouse	11
21439	Salado 2014	CK-1 inhibitor	Drosophila	150
21441	Tada 2014	JAK2 inhibitor	Mouse	25
21443	Ermilova 2005	Zinc	Mouse	9
21445	Habisch 2007	Glatiramer acetate	Mouse	9
21432	Dupuis 2004	High Fat diet	Mouse	6
21434	Forostyak 2014	MSC	Rat	6
21683	Forostyak 2011	MSCs	Rat	11
21451	Gifondorwa 2007	Hsp70	Mouse	20
21461	Lougheed 2011	Methylene Blue	Mouse	16
21864	Turnbull 2012	Apocynin	Mouse	14
21467	Ari 2014	ketonogenic diet	Mouse	11
21469	Potenza 2013	Caffeine	Mouse	10
21470	Kunis 2015	MOG	Mouse	19
21472	Vieira 2014	Dexpramipexole	Mouse	31
21783	Vieira 2015	Guanabenz	Mouse	32
21475	Shimojo 2010	Rosmaric acid	Mouse	14
21480	Lincecum 2010	anti-CD40L	Mouse	44
21491	Barneoud 1999	Aspirin	Mouse	20
21492	Joardar 2015	Pioglitazone	Drosophila	30
21495	Gros-Louis 2010	mAb SOD1	Mouse	8
21496	Fontanilla 2012	Caffeine	Mouse	39
21500	Olivan 2014	Olive oil	Mouse	10
21501	Shoemaker 2007	AM1241	Mouse	14
21509	Raoul 2005	LV-SOD1 knockdown	Mouse	6
21526	Moges 2009	Riboflavin	Mouse	11
21536	Hamadeh 2005	Caloric restriction	Mouse	7
21544	Ohnishi 2009	Bone marrow containing all cell types	Mouse	7
21549	Cervetto 2013	BBG	Mouse	33
21564	Martorana 2012	TAT-BH4	Mouse	14
21572	Jouroukhin 2013	NAP	Mouse	24

For peer review only

21573	Mar-13	mEN101	Mouse	35
21582	Getter 2015	Compound 14 (chaperone)	Mouse	15
21586	Miquel 2014	MitoQ	Mouse	15
21678	Miquel 2012	Dichloroacetate	Mouse	9
21587	Kaddurah-Daouk 2	Creatine	Mouse	7
21598	Gianfocaro 2013	vitamin D	Mouse	46
21599	Kalmar 2008	Acrimolol	Mouse	21
21604	Drachman 2002	celecoxib	Mouse	28
21611	Rembach 2004	asGluR3	Mouse	12
21616	Liebetanz 2004	Exercise	Mouse	12
21633	Koval 2013	anti-miR155-LNA	Mouse	22
21644	Glas 2007	WX-340	Mouse	18
21842	Lorenzl 2006	RO-28--2653	Mouse	19
21842	Lorenzl 2006	RO-28--2653	Mouse	14
21648	Pichon 2013	Erlotinib	Mouse	48
21657	Storkebaum 2005	VEGF	Rat	17
21666	Audet 2012	Cytosine arabinoside	Mouse	7
21882	Audet 2012	Methylene Blue	Mouse	10
21901	Tokuda 2015	Dexamethasone	Mouse	6
21667	Tokuda 2008	Ammonium tetrathiomolybdate	Mouse	17
21671	Kuo 2014	NH4Cl	Mouse	14
21675	Barmada 2014	Fluphenazine	iPSC	18
21679	Koschnitzky 2014	Fluoxetine	Mouse	14
21693	Hefferan 2012	NSCs	Rat	24
21694	Arrat 2015	ACTH	Mouse	14
21702	Pollari 2011	GCSF	Mouse	8
21712	Katsumata 2012	Dasatinib	Mouse	24
21715	Andries 2007	Ivermectin	Mouse	4
21716	Boston-Howes 20(	Nordihydroguaiaretic acid	Mouse	17
21718	Tonges 2014	Fasudil	Mouse	13
21723	Ralph 2005	siRNA SOD-1	Mouse	6
21729	Dibaj 2012	Methylene Blue	Mouse	16
21731	Poesen 2008	VEGF	Rat	8

For peer review only

21733	Pitzer 2008	GCSF	Mouse	18
21737	Ghoddoussi 2010	Methionine	Mouse	15
21739	Linares 2013	TEMPOL (4-hydroxy-TEMPO)	Rat	9
21744	Eitan 2012	AGS-499	Mouse	10
21868	Urushitani 2007	SOD1 Immunization	Mouse	8
21765	Veldink 2003	Exercise	Mouse	13
21765	Veldink 2003	Exercise	Mouse	13
21770	Henriques 2011	GCSF	Mouse	12
21771	Goursaud 2015	SNV	Rat	10
21772	Ayers 2015	AAV2-IL10	Mouse	14
21774	Ishigaki 2007	HGF	Mouse	8
21777	Moreno-Martet 2(	Cannabidiol	Mouse	8
21781	Dang 2013	Progesterone	Mouse	13
21791	Ganel 2006	GLT1 overexpression	Mouse	15
21795	Waibel 2004	Rasagiline	Mouse	15
21798	Deforges 2009	Exercise	Mouse	25
21808	Sabado 2015	SOD1 Immunization	Mouse	10
21810	Nizzardo 2014	NSCs	Mouse	16
21822	Reinholz 1999	PUT-CAT	Mouse	27
21822	Reinholz 1999	PUT-CAT	Mouse	18
21839	Soon 2011	Cu (atsm)	Mouse	14
21850	Lucchetti 2013	Ubiquinol	Mouse	15
21851	Kaneb 2011	Metformin	Mouse	29
21853	Pizzasegola 2009	Lithium	Mouse	10
21858	Hwee 2014	Tirasemtiv	Mouse	81
21862	Haenggeli 2007	Glatiramer acetate	Mouse	15
21863	Boucherie 2009	MSCs	Rat	12
21866	Golko-Perez 2016	M30	Mouse	12
21876	Hottinger 1997	Penicillamine	Mouse	9
21878	Hemendinger 200!	Sertoli cells	Mouse	11
21881	Tortarolo 2006	ZK 187638	Mouse	24
21889	Ghadge 2003	2-MPPA	Mouse	12
21910	Ciriza 2008	GDNF	Mouse	11

For peer review only

21951	Riancho 2015	Bexarotene	Mouse	15

For peer review only

True number of controls	Median survival in treatment group	Median survival in control group	log(Median	Quality Score
9	135	123	0.09309	2
25	180	165	0.087011	2
14	143	122	0.158824	3
11	147	127	0.146246	2
24	140	124	0.121361	3
23	150	130	0.143101	4
19	141	124	0.128478	4
10	149	134	0.106107	4
37	345	300	0.139762	4
20	175	160	0.089612	3
16	147	138	0.063179	3
13	133	126	0.054067	4
7	165	160	0.030772	2
10	110	105	0.04652	2
10	146	117	0.221433	4
15	132	127	0.038615	2
10	270	240	0.117783	2
NA	124	127	-0.02391	7
10	125	134	-0.06953	2
6	134	128	0.04581	2
12	143	133	0.072496	4
12	139.5	127	0.093878	3
10	148	137	0.077231	3
11	148	132	0.11441	1
15	182	140	0.262364	2
24	210	182	0.143101	3
11	240	215	0.110001	2
15	158	131	0.187398	3
12	137	119	0.140857	4
10	139	132	0.051672	3
19	150	144	0.040822	3
15	263	211	0.220296	3

For peer review only

10	136.5	128.5	0.060396	0
21	145	140	0.035091	3
29	155	140	0.101783	4
15	116	104	0.109199	2
8	170	170	0	4
15	127	122	0.040166	3
7	280	250	0.113329	2
4	134	132	0.015038	4
5	145	130	0.109199	3
23	76	82	-0.07599	3
12	153	144	0.060625	4
18	160	145	0.09844	4
54	132	129	0.02299	4
18	385	357	0.075508	3
18	139	126	0.098192	5
24	172	158	0.084899	3
12	288	274	0.049832	2
18	145	132	0.093932	3
25	137	131	0.044784	3
10	145	130	0.109199	2
12	130	123	0.05535	2
6	124.33	109.5	0.127015	7
27	129	125	0.031499	3
5	124	123	0.008097	2
9	148	124	0.176931	3
9	139	130	0.066939	4
10	108	126	-0.15415	3
10	140	130	0.074108	3
15	145	137	0.056753	3
20	157	140	0.114603	4
13	230	155	0.394654	2
24	230	130	0.570545	2
4	135	119	0.126151	5

For peer review only

14	139	130	0.066939	2
20	145	127	0.132547	5
18	134	129	0.038027	2
21	135	125	0.076961	2
12	155	135	0.13815	2
11	150	125	0.182322	2
14	148	127	0.153025	4
11	139	117	0.1723	6
384	1.8	1	0.587787	3
11	132	129	0.02299	3
13	140	130	0.074108	3
15	148	132	0.11441	3
10	146	144	0.013793	3
9	393	352	0.110178	3
16	156	140	0.108214	2
14	185	165	0.11441	2
23	167	175	-0.04679	4
20	147	127	0.146246	2
10	145	130	0.109199	4
9	112	105	0.064539	3
10	136	125	0.084341	2
7	145	142	0.020907	4
9	124	139	-0.11419	3
19	175	170	0.028988	4
11	145	125	0.14842	2
10	750	360	0.733969	3
21	162	147	0.097164	2
34	210	190	0.100083	4
17	135	132	0.022473	3
10	150	130	0.143101	3
11	158	145	0.085861	4
7	157	154	0.019293	3
200	31	23	0.298493	3

For peer review only

16	140	132	0.058841	3
20	130	130	0	1
35	135	127	0.061088	2
16	182	182	0	5
19	132	120	0.09531	3
20	136	122	0.108634	3
31	137	127	0.075794	4
16	134	127	0.053653	3
8	140	127	0.097455	4
8	142	124	0.135545	3
29	140	123	0.129458	3
15	172	152	0.123614	2
11	130	122	0.063513	2
13	161	161	0	2
15	175	150	0.154151	2
12	142	127	0.11164	3
18	134	142	-0.05799	3
8	140	139	0.007168	3
25	175	165	0.058841	3
5	24	12	0.693147	2
32	140	130	0.074108	1
8	295	265	0.107246	3
5	140	140	0	2
21	115	110	0.044452	2
9	125	125	0	4
12	364	336	0.080043	4
10	150	127	0.166448	3
12	130	120	0.080043	3
15	135	120	0.117783	3
10	144	126	0.133531	3
25	130	120	0.080043	3
7	123	112	0.093685	3
10	135	120	0.117783	3

For peer review only

6	168	142	0.168137	3
12	140	128	0.089612	2
24	162	145	0.110863	4
5	285	260	0.091808	3
15	133	126	0.054067	6
6	322	280	0.139762	6
16	159	152	0.045024	6
24	155	150	0.03279	3
10	135	123	0.09309	3
21	151	130	0.149745	6
12	175	165	0.058841	2
26	42	26	0.479573	5
31	40	35	0.133531	2
9	126	119	0.057158	3
6	144	130	0.102279	4
10	135	127	0.061088	3
11	150	140	0.068993	2
11	19.5	17.5	0.108214	3
11	155	140	0.101783	2
25	133	126	0.054067	3
6	141	134	0.05092	4
19	134	128	0.04581	4
11	167	135	0.212719	3
8	135	135	0	2
7	127	120	0.056695	3
8	147	134	0.092593	2
20	145	130	0.109199	3
9	133	127	0.046162	2
7	170	155	0.092373	3
10	125	125	0	6
31	130	120	0.080043	3
7	170	170	0	4
20	130	127	0.023347	2

For peer review only

20	156	143	0.087011	4
13	140	135	0.036368	2
11	132	124	0.06252	2
12	170	150	0.125163	4
10	140	120	0.154151	4
4.5	54	35	0.433636	7
4.5	56	35	0.470004	7
6	145	130	0.109199	3
10	136	132	0.029853	2
6	142	128	0.103797	3
8	140	132	0.058841	3
6	225	225	0	3
14	145	140	0.035091	4
18	105	105	0	3
18	135	130	0.03774	3
8	200	200	0	3
31	168	166	0.011976	3
15	165	170	-0.02985	8
18	149	129	0.144134	3
18	144	130	0.102279	2
22	174	160	0.083881	5
6	182	142	0.24818	3
7	155	130	0.175891	3
15	142	132	0.073025	1
3	295	260	0.126294	2
25	160	123	0.262989	1
25	146	124	0.163325	1
18	150	120	0.223144	2
8	240.5	212.5	0.123778	3
9	50	31	0.478036	0
50	135	125	0.076961	6
16	148.6	150.2	-0.01071	3
14	143	143	0	5

For peer review only

11	127	142	-0.11164	2
11	135	142	-0.05055	2
8	137	125	0.091667	4
12	160	142	0.119347	3
20	137	130	0.052446	3
10	146	126	0.147325	6
12	137	126	0.083699	7
18	151	126	0.180998	4
6	135	115	0.160343	4
11	180	175	0.028171	3
17	141.6	139.4	0.015659	3
7	82	72	0.130053	3
21	210	195	0.074108	3
14	250	196	0.243346	4
20	121	120	0.008299	2
20	125	120	0.040822	2
13	127	123	0.032003	3
12	140	133	0.051293	3
18	135	125	0.076961	4
17	135	133	0.014926	3
30	142	126	0.119545	3
30	150	135	0.105361	3
12	139	127	0.090287	3
14	251	239	0.048989	3
12	171	162	0.054067	5
34	161	163	-0.01235	3
5	185	130	0.352821	3
5	175	130	0.297252	3
5	165	130	0.238411	3
4	35	28	0.223144	2
12.5	135	127	0.061088	5
12.5	129	127	0.015625	5
10	133	127	0.046162	5

For peer review only

8	135	135	0	4
10	137	130	0.052446	3
11	127	102	0.219214	5
150	40	34	0.162519	4
28	150	150	0	3
9	135	120	0.117783	3
9	130	130	0	2
6	120	100	0.182322	3
6	210	200	0.04879	3
8	195	175	0.108214	5
28	130	126	0.031253	2
15	171	168	0.0177	4
13	134	126	0.061558	3
13	121	120	0.008299	5
10	125	119	0.04919	4
23	160	145	0.09844	3
29	126	129.5	-0.0274	5
32	121	126	-0.04049	6
22	165	155	0.06252	4
45	133	124	0.070068	3
18	150	155	-0.03279	2
30	14	14	0	3
8	151	142	0.061453	3
33	134	124	0.077558	3
10	140	125	0.113329	4
NA	210	168	0.223144	6
4	122	102	0.179048	4
12	190	185	0.026668	2
6	134	125	0.069526	3
10	140	128	0.089612	4
25	142	140	0.014185	5
14	137	123	0.107797	4
27	134	132	0.015038	2

For peer review only

35	140	130	0.074108	2
15	140	130	0.074108	3
15	132	125	0.054488	3
9	138	127	0.083067	4
6	169	144	0.160085	1
54	126	124	0.016	4
30	130	126	0.031253	3
27	139	119	0.15535	2
6	139	124	0.114192	3
12	133	131	0.015152	3
20	140	130	0.074108	4
8	135	125	0.076961	2
10	133	123	0.078165	2
10	139	127	0.090287	2
46	158	163	-0.03116	5
17	132	125	0.054488	3
9	141	134	0.05092	3
19	142	142	0	3
5	145	135	0.071459	4
12	149	125	0.175633	4
14	155	140	0.101783	2
18	120	40	1.098612	4
20	139	132	0.051672	3
16	145	142	0.020907	5
16	138	129	0.067441	4
8	183	171	0.067823	3
24	145	135	0.071459	4
4	152	140	0.082238	4
17	150	150	0	2
14	140	130	0.074108	3
7	230	130	0.570545	3
16	130	125	0.039221	3
8	147	137	0.070452	3

For peer review only

17	161	147	0.090972	4
15	154	137	0.116972	4
11	137.5	130	0.056089	4
10	142	127	0.11164	4
7	425	402	0.055637	3
13	240	240	0	2
13	120	112	0.068993	2
12	170	155	0.092373	4
10	220	170	0.257829	2
11	130	115	0.122602	2
8	150	140	0.068993	3
12	130	135	-0.03774	4
14	105	98	0.068993	3
14	250	210	0.174353	2
15	240	220	0.087011	3
50	153	127	0.186251	2
6	260	260	0	2
24	165	150	0.09531	4
33	125	125	0	4
13	265	255	0.038466	4
9	276	254	0.083067	3
15	127	122	0.040166	3
29	126	123	0.024098	3
10	119	129	-0.08069	4
81	119	115	0.034191	5
17	166	160	0.036814	4
12	140	127	0.097455	2
12	126	121	0.040491	4
9	150	139	0.076161	3
11	140	140	0	2
19	160	145	0.09844	2
11	237	190	0.221036	3
11	129	123	0.047628	2

For peer review only

12	139	132	0.051672	3

For peer review only

Peer Review Publication	Statement of potential conflicts of interests	Sample size calculation	Random Allocation to group	Blinded Ass	Allocation c
TRUE	FALSE	FALSE	FALSE	FALSE	FALSE
TRUE	FALSE	FALSE	FALSE	FALSE	FALSE
TRUE	FALSE	FALSE	FALSE	FALSE	TRUE
TRUE	FALSE	FALSE	FALSE	FALSE	FALSE
TRUE	FALSE	FALSE	FALSE	FALSE	TRUE
TRUE	TRUE	FALSE	FALSE	FALSE	FALSE
TRUE	TRUE	FALSE	FALSE	FALSE	FALSE
TRUE	TRUE	FALSE	FALSE	FALSE	TRUE
TRUE	FALSE	FALSE	TRUE	FALSE	FALSE
TRUE	FALSE	FALSE	TRUE	FALSE	FALSE
TRUE	FALSE	FALSE	TRUE	FALSE	FALSE
TRUE	FALSE	FALSE	TRUE	TRUE	FALSE
TRUE	FALSE	FALSE	FALSE	FALSE	FALSE
TRUE	FALSE	FALSE	FALSE	FALSE	FALSE
TRUE	TRUE	FALSE	FALSE	FALSE	TRUE
TRUE	FALSE	FALSE	FALSE	FALSE	FALSE
TRUE	FALSE	FALSE	FALSE	FALSE	FALSE
TRUE	TRUE	TRUE	TRUE	TRUE	TRUE
TRUE	FALSE	FALSE	FALSE	FALSE	FALSE
TRUE	FALSE	FALSE	FALSE	FALSE	FALSE
TRUE	FALSE	FALSE	TRUE	FALSE	TRUE
TRUE	FALSE	FALSE	TRUE	FALSE	FALSE
TRUE	FALSE	FALSE	FALSE	FALSE	FALSE
TRUE	FALSE	FALSE	FALSE	FALSE	FALSE
TRUE	FALSE	FALSE	FALSE	FALSE	FALSE
TRUE	FALSE	FALSE	FALSE	FALSE	TRUE
TRUE	FALSE	FALSE	FALSE	FALSE	FALSE
TRUE	FALSE	FALSE	FALSE	FALSE	TRUE
TRUE	FALSE	FALSE	TRUE	FALSE	TRUE
TRUE	FALSE	FALSE	FALSE	FALSE	TRUE
TRUE	FALSE	FALSE	TRUE	FALSE	FALSE
TRUE	FALSE	FALSE	FALSE	FALSE	TRUE

For peer review only

FALSE	FALSE	FALSE	FALSE	FALSE	FALSE
TRUE	FALSE	FALSE	FALSE	FALSE	FALSE
TRUE	TRUE	FALSE	TRUE	FALSE	FALSE
TRUE	FALSE	FALSE	FALSE	FALSE	FALSE
TRUE	FALSE	FALSE	TRUE	FALSE	FALSE
TRUE	TRUE	FALSE	FALSE	FALSE	FALSE
TRUE	FALSE	FALSE	FALSE	FALSE	FALSE
TRUE	FALSE	FALSE	TRUE	FALSE	TRUE
TRUE	FALSE	FALSE	TRUE	FALSE	FALSE
TRUE	TRUE	FALSE	FALSE	FALSE	FALSE
TRUE	FALSE	FALSE	TRUE	FALSE	TRUE
TRUE	FALSE	FALSE	TRUE	FALSE	TRUE
TRUE	TRUE	FALSE	FALSE	FALSE	TRUE
TRUE	FALSE	FALSE	FALSE	FALSE	TRUE
TRUE	TRUE	FALSE	TRUE	FALSE	TRUE
TRUE	TRUE	FALSE	FALSE	FALSE	FALSE
TRUE	FALSE	FALSE	FALSE	FALSE	FALSE
TRUE	FALSE	FALSE	FALSE	FALSE	TRUE
TRUE	FALSE	FALSE	FALSE	FALSE	TRUE
TRUE	FALSE	FALSE	FALSE	FALSE	FALSE
TRUE	FALSE	FALSE	FALSE	FALSE	FALSE
TRUE	TRUE	FALSE	TRUE	TRUE	TRUE
TRUE	FALSE	FALSE	TRUE	FALSE	FALSE
TRUE	FALSE	FALSE	FALSE	FALSE	FALSE
TRUE	TRUE	FALSE	FALSE	FALSE	FALSE
TRUE	FALSE	FALSE	TRUE	FALSE	TRUE
TRUE	FALSE	FALSE	FALSE	FALSE	TRUE
TRUE	FALSE	FALSE	FALSE	FALSE	TRUE
TRUE	FALSE	FALSE	FALSE	FALSE	TRUE
TRUE	FALSE	FALSE	TRUE	FALSE	TRUE
TRUE	FALSE	FALSE	FALSE	FALSE	FALSE
TRUE	FALSE	FALSE	FALSE	FALSE	FALSE
TRUE	FALSE	FALSE	TRUE	TRUE	TRUE

For peer review only

TRUE	FALSE	FALSE	FALSE	FALSE	FALSE
TRUE	FALSE	FALSE	TRUE	TRUE	TRUE
TRUE	FALSE	FALSE	FALSE	FALSE	FALSE
TRUE	FALSE	FALSE	FALSE	FALSE	FALSE
TRUE	FALSE	FALSE	FALSE	FALSE	FALSE
TRUE	FALSE	FALSE	FALSE	FALSE	FALSE
TRUE	TRUE	FALSE	FALSE	FALSE	TRUE
TRUE	TRUE	FALSE	TRUE	FALSE	TRUE
TRUE	FALSE	FALSE	FALSE	FALSE	FALSE
TRUE	TRUE	FALSE	FALSE	FALSE	FALSE
TRUE	FALSE	FALSE	TRUE	FALSE	FALSE
TRUE	FALSE	FALSE	FALSE	FALSE	TRUE
TRUE	FALSE	FALSE	FALSE	FALSE	FALSE
TRUE	TRUE	FALSE	FALSE	FALSE	FALSE
TRUE	FALSE	FALSE	FALSE	FALSE	FALSE
TRUE	FALSE	FALSE	FALSE	FALSE	FALSE
TRUE	TRUE	FALSE	TRUE	FALSE	FALSE
TRUE	FALSE	FALSE	FALSE	FALSE	FALSE
TRUE	FALSE	FALSE	TRUE	FALSE	TRUE
TRUE	FALSE	FALSE	FALSE	FALSE	FALSE
TRUE	FALSE	FALSE	FALSE	FALSE	FALSE
TRUE	FALSE	FALSE	TRUE	FALSE	TRUE
TRUE	FALSE	FALSE	FALSE	FALSE	TRUE
TRUE	TRUE	FALSE	FALSE	FALSE	TRUE
TRUE	FALSE	FALSE	FALSE	FALSE	FALSE
TRUE	TRUE	FALSE	FALSE	FALSE	FALSE
TRUE	FALSE	FALSE	FALSE	FALSE	FALSE
TRUE	FALSE	FALSE	TRUE	FALSE	TRUE
TRUE	TRUE	FALSE	FALSE	FALSE	FALSE
TRUE	TRUE	FALSE	FALSE	FALSE	FALSE
TRUE	FALSE	FALSE	TRUE	FALSE	FALSE
TRUE	FALSE	FALSE	TRUE	FALSE	FALSE
TRUE	FALSE	FALSE	FALSE	FALSE	FALSE

For peer review only

TRUE	FALSE	FALSE	FALSE	FALSE	TRUE
TRUE	FALSE	FALSE	FALSE	FALSE	FALSE
TRUE	FALSE	FALSE	FALSE	FALSE	FALSE
TRUE	TRUE	FALSE	FALSE	FALSE	TRUE
TRUE	FALSE	FALSE	TRUE	FALSE	FALSE
TRUE	FALSE	FALSE	FALSE	FALSE	TRUE
TRUE	FALSE	FALSE	TRUE	FALSE	TRUE
TRUE	FALSE	FALSE	TRUE	FALSE	FALSE
TRUE	FALSE	FALSE	TRUE	FALSE	TRUE
TRUE	FALSE	FALSE	TRUE	FALSE	FALSE
TRUE	FALSE	FALSE	FALSE	FALSE	TRUE
TRUE	FALSE	FALSE	FALSE	FALSE	FALSE
TRUE	FALSE	FALSE	FALSE	FALSE	FALSE
TRUE	FALSE	FALSE	FALSE	FALSE	FALSE
TRUE	FALSE	FALSE	FALSE	FALSE	FALSE
TRUE	FALSE	FALSE	FALSE	FALSE	FALSE
TRUE	FALSE	FALSE	FALSE	FALSE	TRUE
TRUE	FALSE	FALSE	TRUE	FALSE	FALSE
TRUE	FALSE	FALSE	TRUE	FALSE	FALSE
TRUE	FALSE	FALSE	FALSE	FALSE	FALSE
TRUE	FALSE	FALSE	FALSE	FALSE	FALSE
TRUE	FALSE	FALSE	TRUE	FALSE	FALSE
TRUE	FALSE	FALSE	FALSE	FALSE	FALSE
TRUE	FALSE	FALSE	FALSE	FALSE	FALSE
TRUE	FALSE	FALSE	TRUE	FALSE	TRUE
TRUE	FALSE	FALSE	TRUE	FALSE	FALSE
TRUE	FALSE	FALSE	TRUE	FALSE	FALSE
TRUE	FALSE	FALSE	TRUE	FALSE	FALSE
TRUE	FALSE	FALSE	TRUE	FALSE	FALSE
TRUE	FALSE	FALSE	TRUE	FALSE	FALSE
TRUE	FALSE	FALSE	FALSE	FALSE	TRUE
TRUE	TRUE	FALSE	FALSE	FALSE	FALSE
TRUE	FALSE	FALSE	FALSE	FALSE	TRUE

For peer review only

TRUE	FALSE	FALSE	FALSE	FALSE	FALSE
TRUE	FALSE	FALSE	FALSE	FALSE	FALSE
TRUE	FALSE	FALSE	TRUE	FALSE	TRUE
TRUE	FALSE	FALSE	FALSE	FALSE	TRUE
TRUE	TRUE	FALSE	TRUE	FALSE	TRUE
TRUE	TRUE	FALSE	TRUE	FALSE	TRUE
TRUE	FALSE	FALSE	TRUE	TRUE	TRUE
TRUE	TRUE	FALSE	FALSE	FALSE	FALSE
TRUE	FALSE	FALSE	FALSE	FALSE	TRUE
TRUE	TRUE	FALSE	TRUE	FALSE	TRUE
TRUE	FALSE	FALSE	FALSE	FALSE	FALSE
TRUE	TRUE	FALSE	FALSE	FALSE	TRUE
TRUE	FALSE	FALSE	FALSE	FALSE	FALSE
TRUE	FALSE	FALSE	FALSE	FALSE	FALSE
TRUE	TRUE	FALSE	FALSE	FALSE	TRUE
TRUE	TRUE	FALSE	FALSE	FALSE	FALSE
TRUE	FALSE	FALSE	FALSE	FALSE	FALSE
TRUE	FALSE	FALSE	TRUE	FALSE	FALSE
TRUE	FALSE	FALSE	FALSE	FALSE	FALSE
TRUE	FALSE	FALSE	TRUE	FALSE	FALSE
TRUE	TRUE	FALSE	FALSE	FALSE	FALSE
TRUE	FALSE	FALSE	TRUE	TRUE	FALSE
TRUE	FALSE	FALSE	FALSE	FALSE	TRUE
TRUE	FALSE	FALSE	FALSE	FALSE	FALSE
TRUE	FALSE	FALSE	FALSE	FALSE	FALSE
TRUE	TRUE	FALSE	FALSE	FALSE	FALSE
TRUE	FALSE	FALSE	TRUE	FALSE	FALSE
TRUE	FALSE	FALSE	FALSE	FALSE	FALSE
TRUE	TRUE	FALSE	FALSE	FALSE	FALSE
TRUE	TRUE	FALSE	TRUE	FALSE	TRUE
TRUE	FALSE	FALSE	FALSE	FALSE	TRUE
TRUE	TRUE	FALSE	FALSE	FALSE	FALSE
TRUE	FALSE	FALSE	FALSE	FALSE	FALSE

For peer review only

TRUE	TRUE	FALSE	FALSE	FALSE	TRUE
TRUE	FALSE	FALSE	FALSE	FALSE	FALSE
TRUE	FALSE	FALSE	FALSE	FALSE	FALSE
TRUE	FALSE	FALSE	TRUE	FALSE	FALSE
TRUE	FALSE	FALSE	TRUE	FALSE	TRUE
TRUE	TRUE	FALSE	TRUE	TRUE	TRUE
TRUE	TRUE	FALSE	TRUE	TRUE	TRUE
TRUE	FALSE	FALSE	FALSE	FALSE	TRUE
TRUE	FALSE	FALSE	FALSE	FALSE	FALSE
TRUE	FALSE	FALSE	FALSE	FALSE	TRUE
TRUE	FALSE	FALSE	FALSE	FALSE	TRUE
TRUE	TRUE	FALSE	FALSE	FALSE	FALSE
TRUE	FALSE	FALSE	TRUE	FALSE	TRUE
TRUE	FALSE	FALSE	FALSE	FALSE	TRUE
TRUE	FALSE	FALSE	FALSE	FALSE	TRUE
TRUE	FALSE	FALSE	FALSE	FALSE	TRUE
TRUE	FALSE	FALSE	FALSE	FALSE	FALSE
TRUE	TRUE	TRUE	TRUE	TRUE	TRUE
TRUE	FALSE	FALSE	TRUE	FALSE	FALSE
TRUE	FALSE	FALSE	FALSE	FALSE	FALSE
TRUE	TRUE	FALSE	FALSE	FALSE	TRUE
TRUE	TRUE	FALSE	FALSE	FALSE	FALSE
TRUE	FALSE	FALSE	FALSE	FALSE	TRUE
TRUE	FALSE	FALSE	FALSE	FALSE	FALSE
TRUE	FALSE	FALSE	FALSE	FALSE	FALSE
TRUE	FALSE	FALSE	FALSE	FALSE	FALSE
TRUE	FALSE	FALSE	FALSE	FALSE	FALSE
TRUE	FALSE	FALSE	TRUE	FALSE	FALSE
TRUE	FALSE	FALSE	TRUE	FALSE	FALSE
FALSE	FALSE	FALSE	FALSE	FALSE	FALSE
TRUE	TRUE	FALSE	TRUE	TRUE	TRUE
TRUE	FALSE	FALSE	FALSE	FALSE	FALSE
TRUE	TRUE	FALSE	FALSE	FALSE	TRUE

For peer review only

TRUE	TRUE	FALSE	FALSE	FALSE	FALSE
TRUE	TRUE	FALSE	FALSE	FALSE	FALSE
TRUE	FALSE	FALSE	TRUE	FALSE	FALSE
TRUE	FALSE	FALSE	TRUE	FALSE	FALSE
TRUE	TRUE	FALSE	FALSE	FALSE	FALSE
TRUE	FALSE	FALSE	TRUE	TRUE	TRUE
TRUE	TRUE	FALSE	TRUE	TRUE	TRUE
TRUE	TRUE	FALSE	FALSE	FALSE	TRUE
TRUE	TRUE	FALSE	FALSE	FALSE	TRUE
TRUE	TRUE	FALSE	FALSE	FALSE	FALSE
TRUE	FALSE	FALSE	FALSE	FALSE	TRUE
TRUE	FALSE	FALSE	TRUE	FALSE	FALSE
TRUE	FALSE	FALSE	FALSE	FALSE	TRUE
TRUE	FALSE	FALSE	TRUE	FALSE	TRUE
TRUE	FALSE	FALSE	FALSE	FALSE	FALSE
TRUE	FALSE	FALSE	FALSE	FALSE	FALSE
TRUE	FALSE	FALSE	TRUE	FALSE	FALSE
TRUE	FALSE	FALSE	FALSE	FALSE	TRUE
TRUE	FALSE	FALSE	TRUE	FALSE	TRUE
TRUE	TRUE	FALSE	FALSE	FALSE	FALSE
TRUE	FALSE	FALSE	TRUE	FALSE	FALSE
TRUE	FALSE	FALSE	TRUE	FALSE	FALSE
TRUE	FALSE	FALSE	TRUE	FALSE	FALSE
TRUE	FALSE	FALSE	TRUE	FALSE	FALSE
TRUE	TRUE	FALSE	TRUE	FALSE	FALSE
TRUE	FALSE	FALSE	TRUE	FALSE	FALSE
TRUE	FALSE	FALSE	FALSE	FALSE	TRUE
TRUE	FALSE	FALSE	FALSE	FALSE	TRUE
TRUE	FALSE	FALSE	FALSE	FALSE	TRUE
TRUE	FALSE	FALSE	FALSE	FALSE	FALSE
TRUE	TRUE	FALSE	TRUE	FALSE	TRUE
TRUE	TRUE	FALSE	TRUE	FALSE	TRUE
TRUE	TRUE	FALSE	TRUE	FALSE	TRUE

For peer review only

TRUE	TRUE	FALSE	FALSE	FALSE	TRUE
TRUE	FALSE	FALSE	FALSE	FALSE	TRUE
TRUE	FALSE	FALSE	TRUE	TRUE	TRUE
TRUE	TRUE	FALSE	FALSE	FALSE	FALSE
TRUE	TRUE	FALSE	FALSE	FALSE	FALSE
TRUE	FALSE	FALSE	TRUE	FALSE	FALSE
TRUE	FALSE	FALSE	FALSE	FALSE	FALSE
TRUE	FALSE	FALSE	FALSE	FALSE	FALSE
TRUE	TRUE	FALSE	FALSE	FALSE	FALSE
TRUE	TRUE	FALSE	TRUE	FALSE	FALSE
TRUE	FALSE	FALSE	FALSE	FALSE	FALSE
TRUE	TRUE	FALSE	TRUE	FALSE	FALSE
TRUE	FALSE	FALSE	TRUE	FALSE	FALSE
TRUE	TRUE	FALSE	TRUE	FALSE	FALSE
TRUE	FALSE	FALSE	FALSE	FALSE	TRUE
TRUE	FALSE	FALSE	FALSE	FALSE	TRUE
TRUE	TRUE	FALSE	TRUE	FALSE	TRUE
TRUE	TRUE	FALSE	TRUE	FALSE	TRUE
TRUE	FALSE	FALSE	FALSE	FALSE	TRUE
TRUE	TRUE	FALSE	FALSE	FALSE	FALSE
TRUE	FALSE	FALSE	FALSE	FALSE	FALSE
TRUE	FALSE	FALSE	FALSE	FALSE	FALSE
TRUE	TRUE	FALSE	FALSE	FALSE	FALSE
TRUE	FALSE	FALSE	TRUE	FALSE	FALSE
TRUE	FALSE	FALSE	TRUE	FALSE	TRUE
TRUE	FALSE	TRUE	TRUE	TRUE	TRUE
TRUE	TRUE	FALSE	FALSE	FALSE	TRUE
TRUE	FALSE	FALSE	FALSE	FALSE	FALSE
TRUE	FALSE	FALSE	TRUE	FALSE	FALSE
TRUE	FALSE	FALSE	TRUE	FALSE	TRUE
TRUE	FALSE	FALSE	TRUE	FALSE	TRUE
TRUE	TRUE	FALSE	FALSE	FALSE	TRUE
TRUE	FALSE	FALSE	FALSE	FALSE	FALSE

For peer review only

TRUE	FALSE	FALSE	FALSE	FALSE	FALSE
TRUE	FALSE	FALSE	FALSE	FALSE	TRUE
TRUE	FALSE	FALSE	TRUE	FALSE	FALSE
TRUE	TRUE	FALSE	TRUE	FALSE	FALSE
TRUE	FALSE	FALSE	FALSE	FALSE	FALSE
TRUE	TRUE	FALSE	TRUE	FALSE	FALSE
TRUE	FALSE	FALSE	TRUE	FALSE	FALSE
TRUE	FALSE	FALSE	FALSE	FALSE	FALSE
TRUE	FALSE	FALSE	FALSE	FALSE	TRUE
TRUE	FALSE	FALSE	TRUE	FALSE	FALSE
TRUE	FALSE	FALSE	FALSE	FALSE	TRUE
TRUE	FALSE	FALSE	FALSE	FALSE	FALSE
TRUE	FALSE	FALSE	FALSE	FALSE	FALSE
TRUE	FALSE	FALSE	FALSE	FALSE	FALSE
TRUE	TRUE	FALSE	TRUE	FALSE	TRUE
TRUE	FALSE	FALSE	FALSE	FALSE	TRUE
TRUE	TRUE	FALSE	FALSE	FALSE	FALSE
TRUE	FALSE	FALSE	FALSE	FALSE	TRUE
TRUE	FALSE	FALSE	TRUE	FALSE	TRUE
TRUE	FALSE	FALSE	TRUE	FALSE	TRUE
TRUE	FALSE	FALSE	FALSE	FALSE	FALSE
TRUE	TRUE	FALSE	FALSE	FALSE	FALSE
TRUE	TRUE	FALSE	FALSE	FALSE	FALSE
TRUE	TRUE	FALSE	TRUE	FALSE	TRUE
TRUE	TRUE	FALSE	TRUE	FALSE	TRUE
TRUE	FALSE	FALSE	TRUE	FALSE	FALSE
TRUE	TRUE	TRUE	FALSE	FALSE	FALSE
TRUE	FALSE	FALSE	TRUE	FALSE	TRUE
TRUE	FALSE	FALSE	FALSE	FALSE	FALSE
TRUE	FALSE	FALSE	FALSE	FALSE	TRUE
TRUE	TRUE	FALSE	FALSE	FALSE	FALSE
TRUE	TRUE	FALSE	FALSE	FALSE	FALSE
TRUE	FALSE	FALSE	FALSE	FALSE	TRUE

For peer review only

For peer review only

TRUE	FALSE	FALSE	TRUE	FALSE	TRUE
TRUE	FALSE	FALSE	TRUE	FALSE	TRUE
TRUE	TRUE	FALSE	TRUE	FALSE	FALSE
TRUE	TRUE	FALSE	FALSE	FALSE	TRUE
TRUE	TRUE	FALSE	FALSE	FALSE	FALSE
TRUE	FALSE	FALSE	FALSE	FALSE	FALSE
TRUE	FALSE	FALSE	FALSE	FALSE	FALSE
TRUE	FALSE	FALSE	TRUE	FALSE	TRUE
TRUE	FALSE	FALSE	FALSE	FALSE	FALSE
TRUE	FALSE	FALSE	FALSE	FALSE	FALSE
TRUE	FALSE	FALSE	FALSE	FALSE	TRUE
TRUE	TRUE	FALSE	FALSE	FALSE	FALSE
TRUE	TRUE	FALSE	FALSE	FALSE	FALSE
TRUE	FALSE	FALSE	FALSE	FALSE	FALSE
TRUE	FALSE	FALSE	FALSE	FALSE	TRUE
TRUE	FALSE	FALSE	FALSE	FALSE	FALSE
TRUE	FALSE	FALSE	FALSE	FALSE	FALSE
TRUE	FALSE	FALSE	TRUE	FALSE	TRUE
TRUE	FALSE	FALSE	TRUE	TRUE	FALSE
TRUE	FALSE	FALSE	TRUE	TRUE	FALSE
TRUE	FALSE	FALSE	FALSE	FALSE	TRUE
TRUE	FALSE	FALSE	FALSE	FALSE	TRUE
TRUE	FALSE	FALSE	FALSE	FALSE	TRUE
TRUE	TRUE	FALSE	FALSE	FALSE	TRUE
TRUE	TRUE	FALSE	TRUE	FALSE	TRUE
TRUE	FALSE	FALSE	TRUE	FALSE	FALSE
TRUE	FALSE	FALSE	FALSE	FALSE	FALSE
TRUE	FALSE	FALSE	FALSE	FALSE	TRUE
TRUE	FALSE	FALSE	TRUE	FALSE	FALSE
TRUE	FALSE	FALSE	FALSE	FALSE	FALSE
TRUE	FALSE	FALSE	FALSE	FALSE	FALSE
TRUE	FALSE	FALSE	FALSE	FALSE	TRUE
TRUE	FALSE	FALSE	FALSE	FALSE	FALSE

TRUE	TRUE	FALSE	FALSE	FALSE	FALSE

For peer review only

Appropriat	Compliance	Statement of temperature control
FALSE	TRUE	FALSE
FALSE	TRUE	FALSE
FALSE	TRUE	FALSE
FALSE	TRUE	FALSE
FALSE	TRUE	FALSE
FALSE	TRUE	TRUE
FALSE	TRUE	TRUE
FALSE	TRUE	FALSE
FALSE	TRUE	TRUE
FALSE	TRUE	FALSE
FALSE	TRUE	FALSE
FALSE	TRUE	FALSE
FALSE	TRUE	FALSE
FALSE	TRUE	FALSE
FALSE	TRUE	FALSE
FALSE	TRUE	FALSE
FALSE	TRUE	FALSE
FALSE	TRUE	FALSE
FALSE	TRUE	FALSE
FALSE	TRUE	FALSE
FALSE	TRUE	FALSE
FALSE	TRUE	FALSE
FALSE	TRUE	TRUE
FALSE	FALSE	FALSE
FALSE	TRUE	FALSE
FALSE	TRUE	FALSE
FALSE	TRUE	FALSE
FALSE	TRUE	FALSE
FALSE	TRUE	FALSE
FALSE	TRUE	FALSE
FALSE	TRUE	FALSE
FALSE	TRUE	FALSE

For peer review only

FALSE	FALSE	FALSE
FALSE	TRUE	TRUE
FALSE	TRUE	FALSE
FALSE	TRUE	FALSE
FALSE	TRUE	TRUE
FALSE	TRUE	FALSE
FALSE	TRUE	FALSE
FALSE	TRUE	FALSE
FALSE	TRUE	FALSE
FALSE	TRUE	FALSE
FALSE	TRUE	FALSE
FALSE	TRUE	FALSE
FALSE	TRUE	FALSE
FALSE	TRUE	FALSE
FALSE	TRUE	FALSE
FALSE	TRUE	FALSE
FALSE	TRUE	FALSE
FALSE	TRUE	FALSE
FALSE	TRUE	FALSE
FALSE	TRUE	FALSE
FALSE	TRUE	FALSE
FALSE	TRUE	TRUE
FALSE	TRUE	FALSE
FALSE	TRUE	FALSE
FALSE	TRUE	FALSE
FALSE	TRUE	FALSE
FALSE	TRUE	FALSE
FALSE	TRUE	FALSE
FALSE	TRUE	FALSE
FALSE	TRUE	FALSE
FALSE	TRUE	FALSE
FALSE	TRUE	FALSE
FALSE	TRUE	FALSE

For peer review only

FALSE	TRUE	FALSE
FALSE	TRUE	FALSE
FALSE	TRUE	FALSE
FALSE	TRUE	FALSE
FALSE	TRUE	FALSE
FALSE	TRUE	FALSE
FALSE	TRUE	FALSE
FALSE	TRUE	TRUE
FALSE	TRUE	TRUE
FALSE	TRUE	FALSE
FALSE	TRUE	FALSE
FALSE	TRUE	FALSE
FALSE	TRUE	TRUE
FALSE	TRUE	FALSE
FALSE	TRUE	FALSE
FALSE	TRUE	FALSE
FALSE	TRUE	FALSE
FALSE	TRUE	FALSE
FALSE	TRUE	FALSE
FALSE	TRUE	TRUE
FALSE	TRUE	FALSE
FALSE	TRUE	FALSE
FALSE	TRUE	FALSE
FALSE	TRUE	FALSE
FALSE	TRUE	FALSE
FALSE	TRUE	FALSE
FALSE	TRUE	FALSE
FALSE	TRUE	FALSE
FALSE	TRUE	FALSE
FALSE	TRUE	FALSE
FALSE	TRUE	TRUE
FALSE	TRUE	FALSE
FALSE	TRUE	TRUE

For peer review only

FALSE	TRUE	FALSE
FALSE	FALSE	FALSE
FALSE	TRUE	FALSE
FALSE	TRUE	TRUE
FALSE	TRUE	FALSE
FALSE	TRUE	FALSE
FALSE	TRUE	FALSE
FALSE	TRUE	FALSE
FALSE	TRUE	FALSE
FALSE	TRUE	FALSE
FALSE	TRUE	FALSE
FALSE	TRUE	FALSE
FALSE	TRUE	FALSE
FALSE	TRUE	FALSE
FALSE	TRUE	FALSE
FALSE	TRUE	TRUE
FALSE	TRUE	FALSE
FALSE	TRUE	FALSE
FALSE	TRUE	FALSE
FALSE	TRUE	FALSE
FALSE	FALSE	FALSE
FALSE	TRUE	FALSE
FALSE	TRUE	FALSE
FALSE	TRUE	FALSE
FALSE	TRUE	FALSE
FALSE	TRUE	TRUE
FALSE	TRUE	FALSE
FALSE	TRUE	FALSE
FALSE	TRUE	FALSE
FALSE	TRUE	FALSE
FALSE	TRUE	FALSE
FALSE	TRUE	FALSE
FALSE	TRUE	FALSE

For peer review only

FALSE	TRUE	TRUE
FALSE	TRUE	FALSE
FALSE	TRUE	FALSE
FALSE	TRUE	FALSE
FALSE	TRUE	TRUE
FALSE	TRUE	TRUE
FALSE	TRUE	TRUE
FALSE	TRUE	FALSE
FALSE	TRUE	FALSE
FALSE	TRUE	TRUE
FALSE	TRUE	FALSE
FALSE	TRUE	TRUE
FALSE	TRUE	FALSE
FALSE	TRUE	TRUE
FALSE	TRUE	FALSE
FALSE	TRUE	FALSE
FALSE	TRUE	FALSE
FALSE	TRUE	FALSE
FALSE	TRUE	FALSE
FALSE	TRUE	FALSE
FALSE	TRUE	TRUE
FALSE	TRUE	FALSE
FALSE	TRUE	FALSE
FALSE	TRUE	FALSE
FALSE	TRUE	TRUE
FALSE	FALSE	FALSE
FALSE	TRUE	FALSE
FALSE	TRUE	FALSE
FALSE	TRUE	FALSE
FALSE	TRUE	TRUE
FALSE	TRUE	FALSE
FALSE	TRUE	TRUE
FALSE	TRUE	FALSE

For peer review only

FALSE	TRUE	FALSE
FALSE	TRUE	FALSE
FALSE	TRUE	FALSE
FALSE	TRUE	TRUE
FALSE	TRUE	FALSE
FALSE	TRUE	TRUE
FALSE	TRUE	TRUE
FALSE	TRUE	FALSE
FALSE	TRUE	FALSE
FALSE	TRUE	FALSE
FALSE	TRUE	FALSE
FALSE	TRUE	FALSE
FALSE	TRUE	FALSE
FALSE	TRUE	FALSE
FALSE	TRUE	FALSE
FALSE	TRUE	FALSE
FALSE	TRUE	TRUE
FALSE	TRUE	TRUE
FALSE	TRUE	FALSE
FALSE	TRUE	FALSE
FALSE	TRUE	TRUE
FALSE	TRUE	FALSE
FALSE	TRUE	FALSE
FALSE	FALSE	FALSE
FALSE	TRUE	FALSE
FALSE	FALSE	FALSE
FALSE	FALSE	FALSE
FALSE	FALSE	FALSE
FALSE	TRUE	FALSE
FALSE	FALSE	FALSE
FALSE	TRUE	FALSE
FALSE	TRUE	TRUE
FALSE	TRUE	TRUE

For peer review only

FALSE	FALSE	FALSE
FALSE	FALSE	FALSE
FALSE	TRUE	TRUE
FALSE	TRUE	FALSE
FALSE	TRUE	FALSE
FALSE	TRUE	TRUE
FALSE	TRUE	TRUE
FALSE	TRUE	FALSE
FALSE	TRUE	FALSE
FALSE	TRUE	FALSE
FALSE	TRUE	FALSE
FALSE	TRUE	FALSE
FALSE	TRUE	FALSE
FALSE	TRUE	FALSE
FALSE	TRUE	FALSE
FALSE	TRUE	FALSE
FALSE	TRUE	FALSE
FALSE	TRUE	FALSE
FALSE	TRUE	FALSE
FALSE	TRUE	FALSE
FALSE	TRUE	FALSE
FALSE	TRUE	FALSE
FALSE	TRUE	FALSE
FALSE	TRUE	FALSE
FALSE	TRUE	TRUE
FALSE	TRUE	FALSE
FALSE	TRUE	FALSE
FALSE	TRUE	FALSE
FALSE	TRUE	FALSE
FALSE	TRUE	FALSE
FALSE	TRUE	FALSE
FALSE	TRUE	FALSE
FALSE	TRUE	FALSE

For peer review only

FALSE	TRUE	FALSE
FALSE	TRUE	FALSE
FALSE	TRUE	FALSE
FALSE	TRUE	TRUE
FALSE	TRUE	FALSE
FALSE	TRUE	FALSE
FALSE	TRUE	FALSE
FALSE	TRUE	TRUE
FALSE	TRUE	FALSE
FALSE	TRUE	TRUE
FALSE	TRUE	FALSE
FALSE	TRUE	FALSE
FALSE	TRUE	FALSE
FALSE	TRUE	TRUE
FALSE	TRUE	TRUE
FALSE	TRUE	FALSE
FALSE	TRUE	FALSE
FALSE	TRUE	TRUE
FALSE	TRUE	TRUE
FALSE	TRUE	FALSE
FALSE	TRUE	FALSE
FALSE	TRUE	TRUE
FALSE	TRUE	FALSE
FALSE	TRUE	FALSE
FALSE	TRUE	FALSE
FALSE	TRUE	FALSE
FALSE	TRUE	FALSE
FALSE	TRUE	FALSE
FALSE	TRUE	FALSE
FALSE	TRUE	FALSE
FALSE	TRUE	TRUE
FALSE	TRUE	FALSE
FALSE	TRUE	FALSE

For peer review only

FALSE	TRUE	FALSE
FALSE	TRUE	FALSE
FALSE	TRUE	FALSE
FALSE	TRUE	FALSE
FALSE	FALSE	FALSE
FALSE	TRUE	FALSE
FALSE	TRUE	FALSE
FALSE	TRUE	FALSE
FALSE	TRUE	FALSE
FALSE	TRUE	FALSE
FALSE	TRUE	TRUE
FALSE	TRUE	FALSE
FALSE	TRUE	FALSE
FALSE	TRUE	FALSE
FALSE	TRUE	FALSE
FALSE	TRUE	FALSE
FALSE	TRUE	FALSE
FALSE	TRUE	FALSE
FALSE	TRUE	FALSE
FALSE	TRUE	FALSE
FALSE	TRUE	FALSE
FALSE	TRUE	TRUE
FALSE	TRUE	FALSE
FALSE	TRUE	FALSE
FALSE	TRUE	FALSE
FALSE	TRUE	FALSE
FALSE	TRUE	FALSE
FALSE	TRUE	FALSE
FALSE	TRUE	FALSE
FALSE	TRUE	FALSE
FALSE	TRUE	FALSE
FALSE	TRUE	FALSE
FALSE	TRUE	FALSE

For peer review only

FALSE	TRUE	FALSE
FALSE	TRUE	FALSE
FALSE	TRUE	FALSE
FALSE	TRUE	FALSE
FALSE	TRUE	FALSE
FALSE	TRUE	FALSE
FALSE	TRUE	FALSE
FALSE	TRUE	FALSE
FALSE	TRUE	FALSE
FALSE	TRUE	FALSE
FALSE	TRUE	FALSE
FALSE	TRUE	TRUE
FALSE	TRUE	FALSE
FALSE	TRUE	FALSE
FALSE	TRUE	FALSE
FALSE	TRUE	FALSE
FALSE	TRUE	FALSE
FALSE	TRUE	FALSE
FALSE	TRUE	FALSE
FALSE	TRUE	FALSE
FALSE	TRUE	FALSE
FALSE	TRUE	FALSE
FALSE	TRUE	FALSE
FALSE	TRUE	FALSE
FALSE	TRUE	FALSE
FALSE	TRUE	FALSE
FALSE	TRUE	TRUE
FALSE	TRUE	FALSE
FALSE	TRUE	TRUE
FALSE	TRUE	FALSE
FALSE	TRUE	FALSE
FALSE	TRUE	FALSE
FALSE	TRUE	FALSE
FALSE	TRUE	FALSE

For peer review only

FALSE	TRUE	FALSE

For peer review only

PRISMA 2020 Checklist

Section and Topic	Item #	Checklist item	Location where item is reported
**TITLE**			
Title	1	Identify the report as a systematic review.	NA - the paper describes a process of selecting drugs for which systematic reviews are a part of rather than the only process involved.
**ABSTRACT**			
Abstract	2	See the PRISMA 2020 for Abstracts checklist.	3<br>The abstract does not include all items in the PRISMA 2020 Abstract checklist as the paper describes a process of selecting drugs for which systematic reviews are a part of rather than the only process
**INTRODUCTION**			
Rationale	3	Describe the rationale for the review in the context of existing knowledge.	5
Objectives	4	Provide an explicit statement of the objective(s) or question(s) the review addresses.	5, 6
**METHODS**			
Eligibility criteria	5	Specify the inclusion and exclusion criteria for the review and how studies were grouped for the syntheses.	6, 7
Information sources	6	Specify all databases, registers, websites, organisations, reference lists and other sources searched or consulted to identify studies. Specify the date when each source was last searched or consulted.	6, 7
Search strategy	7	Present the full search strategies for all databases, registers and websites, including any filters and limits used.	6
Selection process	8	Specify the methods used to decide whether a study met the inclusion criteria of the review, including how many reviewers screened each record and each report retrieved, whether they worked independently, and if applicable, details of automation tools used in the process.	7
Data collection process	9	Specify the methods used to collect data from reports, including how many reviewers collected data from each report, whether they worked independently, any processes for obtaining or confirming data from study investigators, and if applicable, details of automation tools used in the process.	7
Data items	10a	List and define all outcomes for which data were sought. Specify whether all results that were compatible with each outcome domain in	7, 8

For peer review only

PRISMA 2020 Checklist

Section and Topic	Item #	Checklist item	Location where item is reported
		each study were sought (e.g. for all measures, time points, analyses), and if not, the methods used to decide which results to collect.	
	10b	List and define all other variables for which data were sought (e.g. participant and intervention characteristics, funding sources). Describe any assumptions made about any missing or unclear information.	7
Study risk of bias assessment	11	Specify the methods used to assess risk of bias in the included studies, including details of the tool(s) used, how many reviewers assessed each study and whether they worked independently, and if applicable, details of automation tools used in the process.	8, 9
Effect measures	12	Specify for each outcome the effect measure(s) (e.g. risk ratio, mean difference) used in the synthesis or presentation of results.	8
Synthesis methods	13a	Describe the processes used to decide which studies were eligible for each synthesis (e.g. tabulating the study intervention characteristics and comparing against the planned groups for each synthesis (item #5)).	6-8
	13b	Describe any methods required to prepare the data for presentation or synthesis, such as handling of missing summary statistics, or data conversions.	6-9
	13c	Describe any methods used to tabulate or visually display results of individual studies and syntheses.	6-9
	13d	Describe any methods used to synthesize results and provide a rationale for the choice(s). If meta-analysis was performed, describe the model(s), method(s) to identify the presence and extent of statistical heterogeneity, and software package(s) used.	6-9
	13e	Describe any methods used to explore possible causes of heterogeneity among study results (e.g. subgroup analysis, meta-regression).	NA
	13f	Describe any sensitivity analyses conducted to assess robustness of the synthesized results.	NA
Reporting bias assessment	14	Describe any methods used to assess risk of bias due to missing results in a synthesis (arising from reporting biases).	NA
Certainty assessment	15	Describe any methods used to assess certainty (or confidence) in the body of evidence for an outcome.	9
**RESULTS**			
Study selection	16a	Describe the results of the search and selection process, from the number of records identified in the search to the number of studies included in the review, ideally using a flow diagram.	10
	16b	Cite studies that might appear to meet the inclusion criteria, but which were excluded, and explain why they were excluded.	10
Study characteristics	17	Cite each included study and present its characteristics.	Supplemental data; We did not cite all studies in main manuscript due to volume of studies.
Risk of bias in studies	18	Present assessments of risk of bias for each included study.	Supplemental data
Results of individual studies	19	For all outcomes, present, for each study: (a) summary statistics for each group (where appropriate) and (b) an effect estimate and its precision (e.g. confidence/credible interval), ideally using structured tables or plots.	Supplemental data for study-level results. We present the drug-level results in page 10-11
Results of	20a	For each synthesis, briefly summarise the characteristics and risk of bias among contributing studies.	Supplemental data for study-

For peer review only

PRISMA 2020 Checklist

Section and Topic	Item #	Checklist item	Location where item is reported
syntheses			level results. We present the drug-level results in page 10-11
	20b	Present results of all statistical syntheses conducted. If meta-analysis was done, present for each the summary estimate and its precision (e.g. confidence/credible interval) and measures of statistical heterogeneity. If comparing groups, describe the direction of the effect.	Supplemental data for study-level results. We present the drug-level results in page 10-11
	20c	Present results of all investigations of possible causes of heterogeneity among study results.	Not applicable here – the nature of our systematic review where we include publications on different neurodegenrative diseases, using different interventions and outcomes means that we expect results to be heterogenous.
	20d	Present results of all sensitivity analyses conducted to assess the robustness of the synthesized results.	NA – see above
Reporting biases	21	Present assessments of risk of bias due to missing results (arising from reporting biases) for each synthesis assessed.	NA – see above
Certainty of evidence	22	Present assessments of certainty (or confidence) in the body of evidence for each outcome assessed.	NA – see above
**DISCUSSION**			
Discussion	23a	Provide a general interpretation of the results in the context of other evidence.	16-19
	23b	Discuss any limitations of the evidence included in the review.	16-17
	23c	Discuss any limitations of the review processes used.	18
	23d	Discuss implications of the results for practice, policy, and future research.	16-19
**OTHER INFORMATION**			
Registration and protocol	24a	Provide registration information for the review, including register name and registration number, or state that the review was not registered.	6
	24b	Indicate where the review protocol can be accessed, or state that a protocol was not prepared.	6
	24c	Describe and explain any amendments to information provided at registration or in the protocol.	6
Support	25	Describe sources of financial or non-financial support for the review, and the role of the funders or sponsors in the review.	19
Competing	26	Declare any competing interests of review authors.	19

For peer review only

PRISMA 2020 Checklist

Section and Topic	Item #	Checklist item	Location where item is reported
interests			
Availability of data, code and other materials	27	Report which of the following are publicly available and where they can be found: template data collection forms; data extracted from included studies; data used for all analyses; analytic code; any other materials used in the review.	20

From: Page MJ, McKenzie JE, Bossuyt PM, Boutron I, Hoffmann TC, Mulrow CD, et al. The PRISMA 2020 statement: an updated guideline for reporting systematic reviews. BMJ 2021;372:n71. doi: 10.1136/bmj.n71

For more information, visit: http://www.prisma-statement.org/

For peer review only

PRISMA 2020 Checklist

Section and Topic	Item #	Checklist item	Location where item is reported
**TITLE**			
Title	1	Identify the report as a systematic review.	NA - the paper describes a process of selecting drugs for which the preclinical systematic review forms a part of rather than being the only process involved.
**ABSTRACT**			
Abstract	2	See the PRISMA 2020 for Abstracts checklist.	3 The abstract does not include all items in the PRISMA 2020 Abstract checklist as the paper describes a process of selecting drugs for which the preclinical systematic review forms a part of rather than being the only process involved.
**INTRODUCTION**			
Rationale	3	Describe the rationale for the review in the context of existing knowledge.	5, 6
Objectives	4	Provide an explicit statement of the objective(s) or question(s) the review addresses.	5, 6

For peer review only

PRISMA 2020 Checklist

Section and Topic	Item #	Checklist item	Location where item is reported
**METHODS**			
Eligibility criteria	5	Specify the inclusion and exclusion criteria for the review and how studies were grouped for the syntheses.	9; protocol in ref(23)
Information sources	6	Specify all databases, registers, websites, organisations, reference lists and other sources searched or consulted to identify studies. Specify the date when each source was last searched or consulted.	9; protocol in ref(23)
Search strategy	7	Present the full search strategies for all databases, registers and websites, including any filters and limits used.	9; protocol in ref(23)
Selection process	8	Specify the methods used to decide whether a study met the inclusion criteria of the review, including how many reviewers screened each record and each report retrieved, whether they worked independently, and if applicable, details of automation tools used in the process.	9; protocol in ref(23)
Data collection process	9	Specify the methods used to collect data from reports, including how many reviewers collected data from each report, whether they worked independently, any processes for obtaining or confirming data from study investigators, and if applicable, details of automation tools used in the process.	9; protocol in ref(23)
Data items	10a	List and define all outcomes for which data were sought. Specify whether all results that were compatible with each outcome domain in each study were sought (e.g. for all measures, time points, analyses), and if not, the methods used to decide which results to collect.	9; protocol in ref(23)
	10b	List and define all other variables for which data were sought (e.g. participant and intervention characteristics, funding sources). Describe any assumptions made about any missing or unclear information.	9; protocol in ref(23)
Study risk of bias assessment	11	Specify the methods used to assess risk of bias in the included studies, including details of the tool(s) used, how many reviewers assessed each study and whether they worked independently, and if applicable, details of automation tools used in the process.	9; protocol in ref(23)
Effect measures	12	Specify for each outcome the effect measure(s) (e.g. risk ratio, mean difference) used in the synthesis or presentation of results.	9; protocol in ref(23)
Synthesis methods	13a	Describe the processes used to decide which studies were eligible for each synthesis (e.g. tabulating the study intervention characteristics and comparing against the planned groups for each synthesis (item #5)).	9; protocol in ref(23)
	13b	Describe any methods required to prepare the data for presentation or synthesis, such as handling of missing summary statistics, or data conversions.	9; protocol in ref(23)
	13c	Describe any methods used to tabulate or visually display results of individual studies and syntheses.	9; protocol in ref(23)
	13d	Describe any methods used to synthesize results and provide a rationale for the choice(s). If meta-analysis was performed, describe the model(s), method(s) to identify the presence and extent of statistical heterogeneity, and software package(s) used.	9; protocol in ref(23)
	13e	Describe any methods used to explore possible causes of heterogeneity among study results (e.g. subgroup analysis, meta-regression).	9; protocol in ref(23)
	13f	Describe any sensitivity analyses conducted to assess robustness of the synthesized results.	9; protocol in ref(23)
Reporting bias assessment	14	Describe any methods used to assess risk of bias due to missing results in a synthesis (arising from reporting biases).	9; protocol in ref(23)
Certainty assessment	15	Describe any methods used to assess certainty (or confidence) in the body of evidence for an outcome.	9; protocol in ref(23)
**RESULTS**			
Study selection	16a	Describe the results of the search and selection process, from the number of records identified in the search to the number of studies included	12

For peer review only

For peer review only - http://bmjopen.bmj.com/site/about/guidelines.xhtml

PRISMA 2020 Checklist

Section and Topic	Item #	Checklist item	Location where item is reported
		in the review, ideally using a flow diagram.	
	16b	Cite studies that might appear to meet the inclusion criteria, but which were excluded, and explain why they were excluded.	Full results not reported here as the aim of this paper is to describe a process of selecting drug, which includes use of data from the preclinical systematic reviews, rather than the describing the full preclinical systematic review.
Study characteristics	17	Cite each included study and present its characteristics.	Supplemental data
Risk of bias in studies	18	Present assessments of risk of bias for each included study.	Supplemental data
Results of individual studies	19	For all outcomes, present, for each study: (a) summary statistics for each group (where appropriate) and (b) an effect estimate and its precision (e.g. confidence/credible interval), ideally using structured tables or plots.	13, Supplemental data
Results of syntheses	20a	For each synthesis, briefly summarise the characteristics and risk of bias among contributing studies.	Supplemental data
	20b	Present results of all statistical syntheses conducted. If meta-analysis was done, present for each the summary estimate and its precision (e.g. confidence/credible interval) and measures of statistical heterogeneity. If comparing groups, describe the direction of the effect.	Page 13; Supplemental data
	20c	Present results of all investigations of possible causes of heterogeneity among study results.	Because survival was reported in a single paper for 11 of these 20 interventions, we did not conduct a

For peer review only

PRISMA 2020 Checklist

Section and Topic	Item #	Checklist item	Location where item is reported
			meta-analysis.
	20d	Present results of all sensitivity analyses conducted to assess the robustness of the synthesized results.	Because survival was reported in a single paper for 11 of these 20 interventions, we did not conduct a meta-analysis.
Reporting biases	21	Present assessments of risk of bias due to missing results (arising from reporting biases) for each synthesis assessed.	Because survival was reported in a single paper for 11 of these 20 interventions, we did not conduct a meta-analysis.
Certainty of evidence	22	Present assessments of certainty (or confidence) in the body of evidence for each outcome assessed.	Because survival was reported in a single paper for 11 of these 20 interventions, we did not conduct a meta-analysis.
**DISCUSSION**			
Discussion	23a	Provide a general interpretation of the results in the context of other evidence.	16-18
	23b	Discuss any limitations of the evidence included in the review.	16, 17
	23c	Discuss any limitations of the review processes used.	18
	23d	Discuss implications of the results for practice, policy, and future research.	17-18
**OTHER INFORMATION**			
Registration and	24a	Provide registration information for the review, including register name and registration number, or state that the review was not registered.	9, ref 23

For peer review only

PRISMA 2020 Checklist

Section and Topic	Item #	Checklist item	Location where item is reported
protocol	24b	Indicate where the review protocol can be accessed, or state that a protocol was not prepared.	6, 9
	24c	Describe and explain any amendments to information provided at registration or in the protocol.	6
Support	25	Describe sources of financial or non-financial support for the review, and the role of the funders or sponsors in the review.	19
Competing interests	26	Declare any competing interests of review authors.	19
Availability of data, code and other materials	27	Report which of the following are publicly available and where they can be found: template data collection forms; data extracted from included studies; data used for all analyses; analytic code; any other materials used in the review.	20

From: Page MJ, McKenzie JE, Bossuyt PM, Boutron I, Hoffmann TC, Mulrow CD, et al. The PRISMA 2020 statement: an updated guideline for reporting systematic reviews. BMJ 2021;372:n71. doi: 10.1136/bmj.n71

For more information, visit: http://www.prisma-statement.org/

For peer review only
